# Deep Learning-Driven Library Design for the *De Novo* Discovery of Bioactive Thiopeptides

**DOI:** 10.1021/acscentsci.3c00957

**Published:** 2023-11-07

**Authors:** Jun Shi Chang, Alexander A. Vinogradov, Yue Zhang, Yuki Goto, Hiroaki Suga

**Affiliations:** † Department of Chemistry, Graduate School of Science, The University of Tokyo, Bunkyo-ku, Tokyo 113-0033, Japan

## Abstract

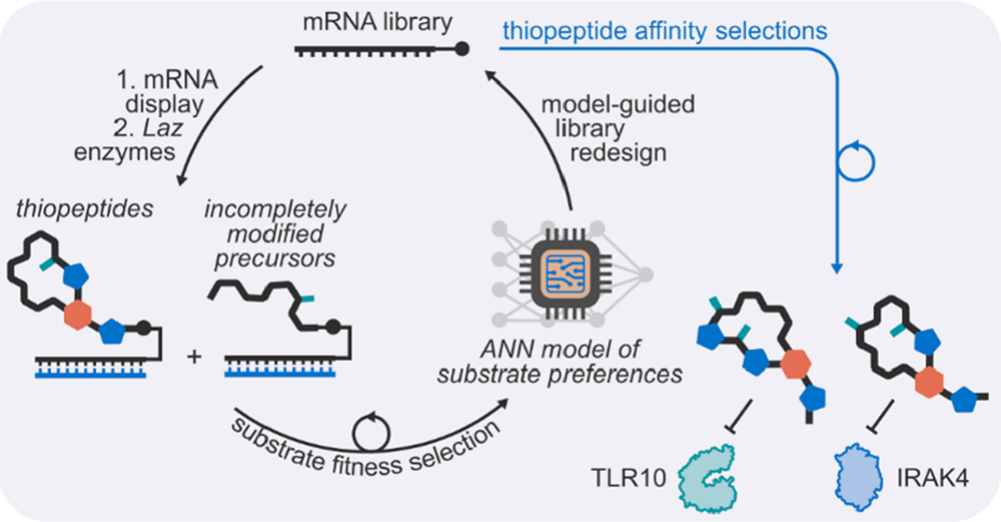

Broad substrate tolerance of ribosomally synthesized and post-translationally modified peptide (RiPP) biosynthetic enzymes has allowed numerous strategies for RiPP engineering. However, despite relaxed specificities, exact substrate preferences of RiPP enzymes are often difficult to pinpoint. Thus, when designing combinatorial libraries of RiPP precursors, balancing the compound diversity with the substrate fitness can be challenging. Here, we employed a deep learning model to streamline the design of mRNA display libraries. Using an *in vitro* reconstituted thiopeptide biosynthesis platform, we performed mRNA display-based profiling of substrate fitness for the biosynthetic pathway involving five enzymes to train an accurate deep learning model. We then utilized the model to design optimal mRNA libraries and demonstrated their utility in affinity selections against IRAK4 kinase and the TLR10 cell surface receptor. The selections led to the discovery of potent thiopeptide ligands against both target proteins (K_D_ up to 1.3 nM for the best compound against IRAK4 and 300 nM for TLR10). The IRAK4-targeting compounds also inhibited the kinase at single-digit μM concentrations *in vitro*, exhibited efficient internalization into HEK293H cells, and suppressed NF-kB-mediated signaling in cells. Altogether, the developed approach streamlines the discovery of pseudonatural RiPPs with *de novo* designed biological activities and favorable pharmacological properties.

## Abstract 2

A pipeline for the automated design of combinatorial libraries streamlines the de novo discovery of pseudonatural thiopeptides with designed biological activities.

## Introduction

Numerous recently developed strategies to improve, alter, or even completely reprogram the bioactivities of ribosomally synthesized and post-translationally modified peptides (RiPPs)^1,2^ have enabled the rapid exploration of pseudonatural product structures in drug discovery applications.^3−9^ Most RiPP engineering techniques focus on modifying the primary sequences of RiPP precursor peptides with no or minimal perturbation to the underlying enzymatic machinery, which offers a straightforward way to generate molecular diversity. Such strategies are enabled by the unique promiscuity of many RiPP biosynthetic enzymes capable of modifying a broad range of peptides.^10−12^ However, the exact substrate preferences of RiPP enzymes are difficult to explain, generalize, and predict in many cases.^13−18^ The interpretation of the substrate scope for the pathways composed of multiple enzymes which cooperate and compete with each other during biosynthesis can be particularly challenging.^19^ Therefore, when constructing combinatorial libraries of RiPP precursor peptides, library design becomes critically important. How do we design a library where individual peptides have the highest chance for enzymatic modification while keeping the compounds as diverse as possible? A good design should offer a compromise between these considerations. If the precursor peptides are poorly modified by the enzymes, then the resulting library mostly contains linear, unconstrained structures, whereas low-diversity libraries decrease the chances of finding compounds with bioactivities of interest.

**Figure 1:**
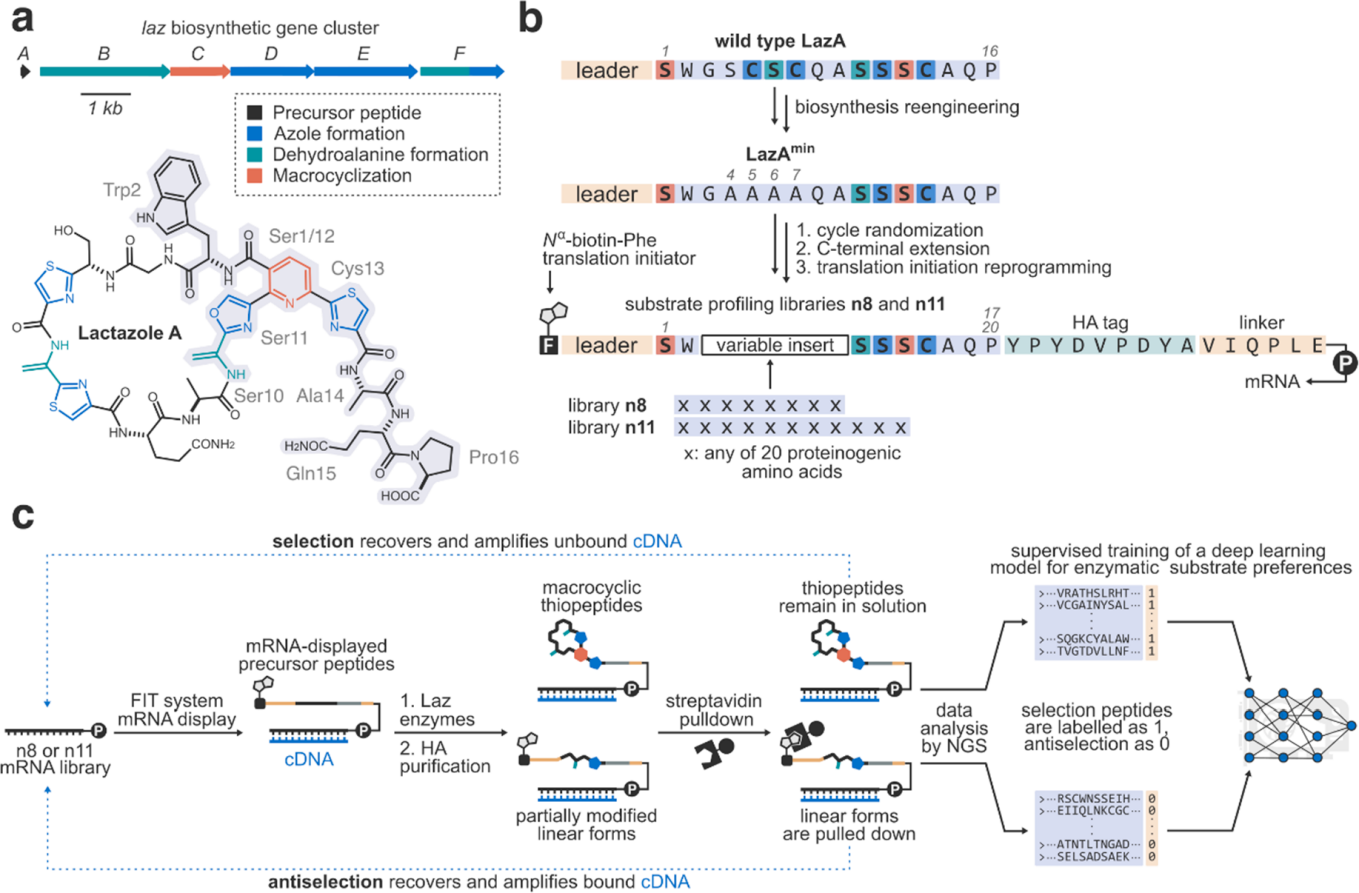
Design of the substrate specificity profiling pipeline. a) Lactazole A is a thiopeptide biosynthesized by five enzymes, LazBCDEF. Depicted are the *laz* biosynthetic gene cluster and the chemical structure of lactazole A; the structure highlighted in gray is shared between the natural product and library thiopeptides. b) Design of LazA-derived libraries n8 and n11, which feature 8- and 11-mer random inserts inside the core peptide region of precursor peptide LazA. Compared to the wild-type sequence, n8 and n11 also contain the HA-affinity tag, C-terminal linker, and *N*^α^-biotin-Phe as the translation initiator amino acid. c) Schematic overview of the pipeline to train an accurate model of the substrate preferences for the biosynthetic pathway. Randomized precursor peptides are first displayed on cognate mRNA and are then treated with Laz enzymes. Partially modified and shunt products maintain the N-terminal biotin tag and can be isolated by a pulldown with streptavidin beads, whereas thiopeptides remain in solution. Next-generation sequencing of cDNA barcodes produces large amounts of data to train a deep convolutional neural network-based classifier of enzymatic substrate preferences.

Previously, we reported the development of an mRNA display-based platform for the discovery of pseudonatural thiopeptide RiPPs with *de novo* designed biological activities.^20^ The platform leverages the five enzymes (LazBCDEF) from the lactazole biosynthesis pathway^21^ to access large libraries of lactazole-like thiopeptides for affinity selection against target proteins of interest (Figure 1a). mRNA-tagged partially randomized precursor peptides (LazA variants) are prepared using the flexible in vitro translation (FIT) system^22^ and the standard mRNA display techniques.^23^ Enzymatic treatment of such libraries with LazBCDEF affords lactazole-like thiopeptides for downstream affinity selection. As a proof of principle of the platform, we developed several potent inhibitors of a therapeutically relevant enzyme. This workflow relies on the remarkable catalytic promiscuity of LazBCDEF which require only seven amino acids (Ser1, Trp2, Ser10, Ser11, Ser12, Cys13, and Ala14) in the LazA core peptide for its maturation to macrocyclic thiopeptides and can operate on a variety of sequence-randomized LazA variants.^24^ Nevertheless, despite extensive efforts to understand the substrate preferences of Laz enzymes, the accurate prediction of whether a particular artificial precursor can be efficiently converted to the corresponding thiopeptide product remains challenging.^13,19,25^ As we argue above, addressing this challenge is crucial to designing combinatorial libraries with the highest average modification efficiency, which in turn should lead to a more reliable *de novo* identification of thiopeptide ligands. Incomplete maturation of precursor peptides not only decreases the library diversity but also contaminates it with unwanted linear species that can compete for binding with the designed thiopeptides. Analogous concerns also arise in other RiPP engineering approaches, thus representing a general problem in the field.^26−32^

Here, we report the application of a deep learning model to facilitate the optimal design of a LazA-based mRNA display library to address the aforementioned challenge. Specifically, we employed mRNA display for a comprehensive assessment of the substrate fitness of the entire lactazole pathway and then trained an accurate convolutional neural network classifier of enzymatic preferences using the obtained profiling data. The resulting model enabled us to design LazA libraries that offer an optimal balance between compound diversity and maturation efficiency. Finally, we demonstrated the success of this approach in affinity selections against two proteins involved in innate immunity processes, interleukin-1 receptor-associated kinase 4 (IRAK4) and toll-like receptor 10 (TLR10), both of which delivered multiple potent thiopeptide ligands against the selected targets. The IRAK4-targeting thiopeptides also inhibited the enzyme *in vitro* and in cell models. This work establishes a general approach to combinatorial library design, simplifies the development of similar engineering workflows for other RiPP biosynthetic pathways, and overall streamlines the discovery of pseudonatural products with favorable pharmacological properties for medicinal chemistry applications.

## Results and Discussion

### Data Acquisition and Model Training

At the outset, we reasoned that an accurate model of substrate specificity encompassing the entire pathway would facilitate the design of optimal precursor peptide libraries. Such a model could extrapolate substrate fitness across the available sequence space, allowing for a direct comparison between the maturation efficiencies of various library designs with minimal computational and experimental overhead. To obtain the requisite model, we sought to utilize mRNA display to dissect the fitness landscapes of the lactazole biosynthetic pathway and deep learning to analyze the resulting data. We recently developed deep learning models to understand the substrate fitness of some of the individual enzymes (Ser/Thr dehydratase LazBF and Ser/Thr/Cys cyclodehydratase LazDE) but not the entire pathway.^13^ Substrate preferences of the full pathway are obfuscated by the fact that the enzymes extensively cooperate and compete with each other during biosynthesis.^19^ Cyclodehydration of Ser and Cys residues, azoline dehydrogenation, and dehydroalanine formation events are intertwined during the maturation process to maintain the integrity of the biosynthesis and minimize the formation of shunt products. The substrate preferences of the whole pathway are composed of the preferences of the individual enzymes and numerous second-order effects stemming from the fact that the installations of post-translational modifications (PTMs) influence each other.

Here, we employed mRNA display techniques^13,23,25,33^ to construct a randomized LazA library and utilize it in a DADL (data acquisition for deep learning) applications experiment to acquire two data sets: one corresponding to LazA variants which are efficiently macrocyclized to thiopeptides and the other for poor substrates that either undergo shunt modification or remain partially processed/unmodified (linear forms). Discrimination of one substrate population from another is achieved by genetic code reprogramming during *in vitro* translation, which installs *N*^α^-biotinylated Phe as the N-terminal (translation initiator) amino acid (Figure 1b). Upon treating mRNA-displayed LazA variants with LazBCDEF (a two-step incubation protocol as detailed in S.I. 2.3), the substrates that undergo complete maturation lose the N-terminal biotin tag because the pyridine synthase LazC cleaves the first 38 amino acids of LazA (leader peptide, leader-NH_2_) during the last step of the maturation process. In contrast, all linear forms maintain the tag at their N-terminus. Because the remaining enzymes act upstream of LazC and their PTMs are required for LazC activity, leader peptide cleavage informs the success of the entire pathway. Therefore, following an indispensable HA-affinity purification step to denoise the library, the substrate populations can be separated by a streptavidin pulldown, where poor substrates are retained by magnetic beads while fully modified thiopeptides remain in solution. Upon recovering the respective DNA barcodes (**cDNA**), the process can be repeated to obtain populations of progressively higher or lower fitness as desired (Figure 1c).

**Figure 2:**
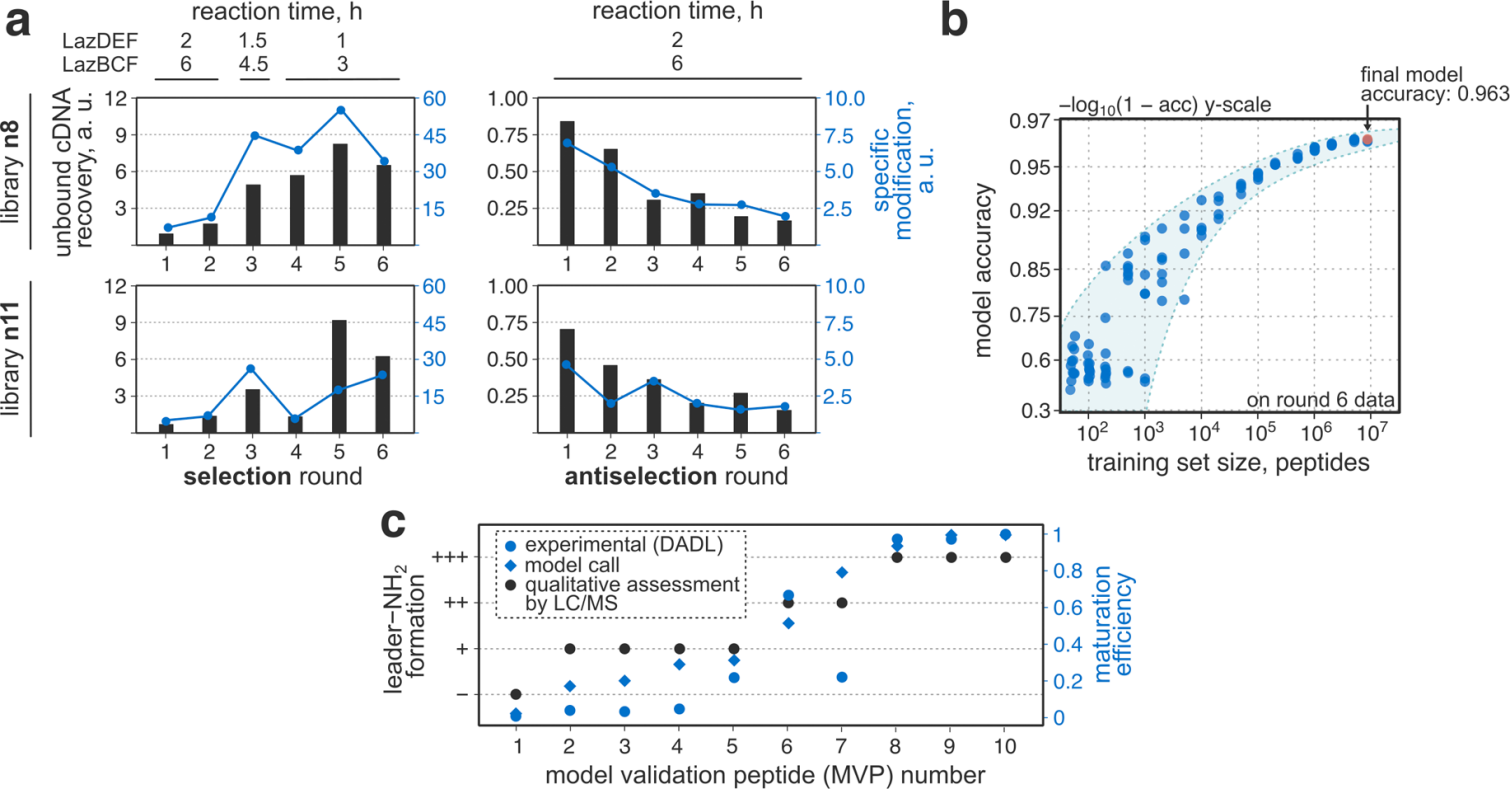
mRNA display profiling of the lactazole biosynthesis pathway and model training. a) Summary of the selection and antiselection experiments for libraries n8 and n11. Plotted are unbound cDNA recovery (thiopeptide fraction) and specific modification values (S.I. 2.1) as measured by qPCR after every round of mRNA display. b) Model accuracy as a function of the training data set size. The models were trained on round 6 data. The final model utilized for downstream applications is highlighted in orange. c) Validation of model predictions against experimental data (S.I. 2.5). Maturation of model validation peptides (MVP1–10; see also Table S4 and Figures S1–S10) was ascertained experimentally using a single-round DADL (data acquisition for deep learning applications) assay (S.I. 2.3) or by LC/MS (semiquantitative monitoring of leader-NH_2_ formation; S.I. 2.7). Model predictions showed good agreement with the experiments.

We conducted separate DADL experiments using two large LazA-based libraries, each consisting of initial 6 × 10^12^ mRNA molecules. Library n11 contained a fully randomized 11-mer insert sequence (theoretical diversity: 2 × 10^14^ peptides), and library n8 had an analogous 8-mer insert (2.6 × 10^10^ peptides) (Figure 1b). For each library, we conducted a “selection” experiment, where the unbound fraction representing fully modified thiopeptides was recovered and amplified at every round. In an “antiselection”, the bound fraction (linear forms) was enriched to produce a population consisting of progressively poorer substrates at every round. We performed six rounds of selection and antiselection for both libraries. In the selection experiments, the enzymatic reaction time was shortened from the original 8 to 4 h in rounds 4, 5, and 6. In both selections, the recovery of unbound cDNA and specific modification (for the technical definition, see S.I. 2.1; measured by qPCR) increased from round to round, suggesting that the experiments progressed as designed. For antiselections, the same metrics decreased over the course of six rounds, indicating that the final libraries were depleted of high-fitness peptides (Figure 2a).

The resulting DNA was analyzed by next-generation sequencing (NGS; S.I. 2.4). DNA reads were in-silico translated and preprocessed to remove noise, overly mutated or truncated sequences, and reads containing low-confidence base calls (Phred scores <30). The obtained peptide lists were further preprocessed by discarding the entries found in both selection and antiselection data sets, and the unique sequences from the n11 and n8 library lists were combined to produce two data sets: the selection peptide list with 4.8 × 10^6^ entries and the antiselection data set containing 3.9 × 10^6^ peptides. All sequences in the selection data set received a label of “1”, while all antiselection peptides were labeled as “0”. The data sets (8.47 × 10^6^ unique peptides) were then used to train a deep convolutional neural network classifier (12 convolutional layers, 1.2 × 10^7^ parameters) of substrate fitness for LazA variants. The final model had excellent accuracy (0.963) when evaluated against 1.7 × 10^5^ unseen peptides from the NGS data sets (Figure 2b).

To further validate that classifications were based on substrate fitness and not on mRNA display-related artifacts, we prepared 10 random library members (MVP1–10, Table S4). Because the exact quantification of thiopeptides produced from the FIT system-derived precursor peptides is challenging (see S.I. 2.5 for additional discussion), we studied these constructs in two ways. First, we employed an LC/MS assay (S.I. 2.7 and Figures S1–S10) to qualitatively evaluate the efficiency of the macrocyclization by following the production of leader-NH_2_. A single-round, single-substrate DADL experiment constituted the second analysis method (S.I. 2.3), which provided a semiquantitative metric of maturation yields. In 9 out of the 10 cases, the model predictions were consistent with the mRNA display and LC/MS outcomes (Figure 2c). For MVP7, the DADL assay indicated a low thiopeptide yield, but both the model and LC/MS results agreed that MVP7 is a competent substrate (Figure S7). Altogether, we concluded that the trained model is an accurate and reliable estimator of substrate fitness across the peptide space of the LazA variants.

### Model-Guided Library Design

**Figure 3:**
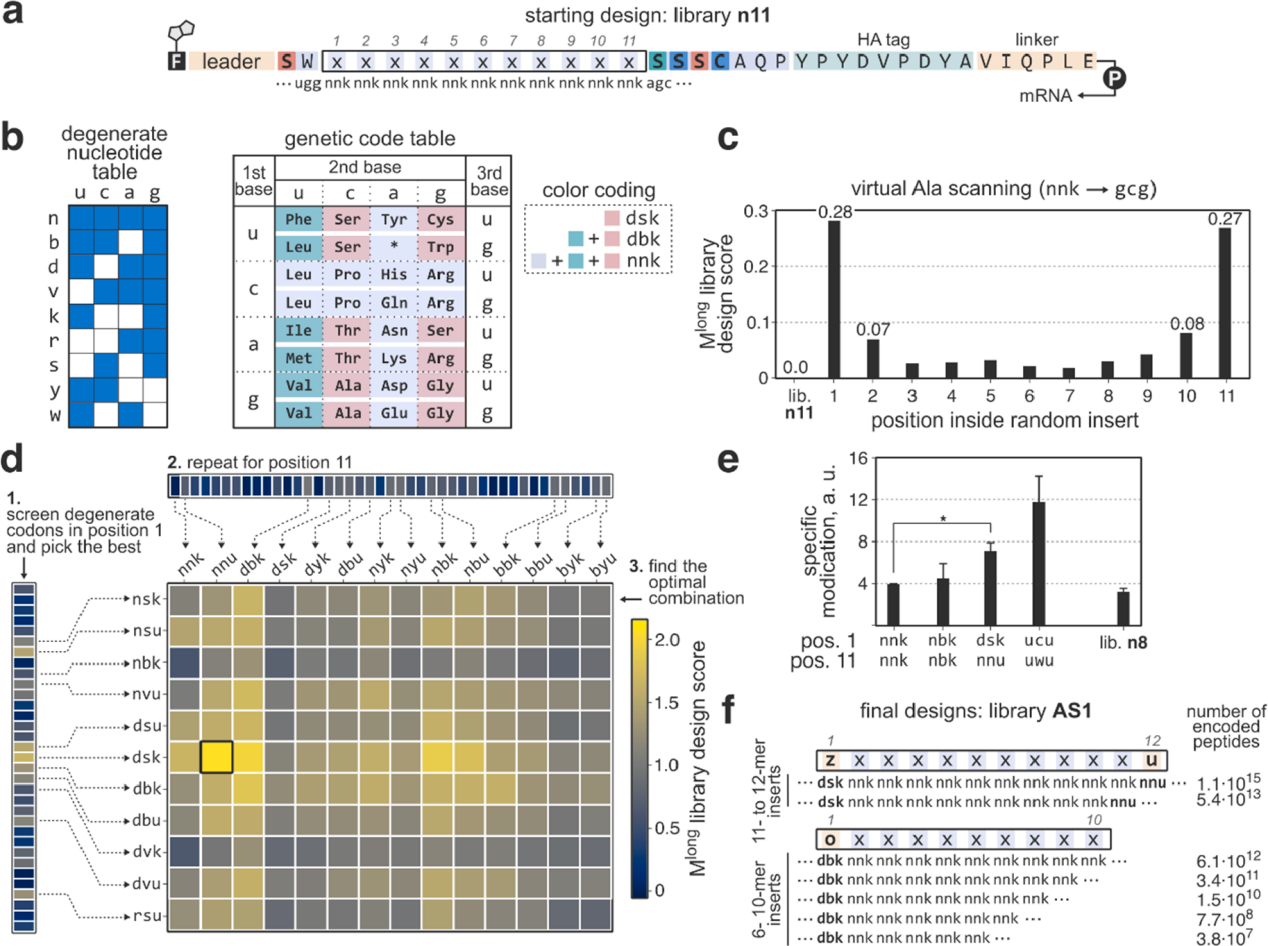
Model-guided library design. a) Initial library design featuring a fully randomized 11-mer insert. b) Degenerate nucleotide nomenclature and the genetic code table for reference. c) In silico Ala scan of library n11. For details regarding the calculation of library design scores using model predictions, see S.I. 2.1. The experiment points to positions 1 and 11, i.e., those adjacent to the modification sites, as critical for thiopeptide maturation. d) Identification of the optimal degenerate codon combinations in positions 1 and 11. e) Experimental measurement of maturation efficiencies for several library designs using a single-round DADL assay. Displayed are specific modification values (S.I. 2.1) as measured by qPCR. The identified design (dsk/nnu) undergoes more efficient maturation as compared to the fully random (nnk/nnk) and previously reported (nbk/nbk) libraries.*: *p* < 0.005 (two-tailed equal variance *t*-test). f) Final library designs. mRNA bearing 6–12-mer inserts were mixed to generate the final selection library AS1.

To assess the fitness of a particular mRNA library design, we in silico generated 10^4^ random peptides encoded by a combination of degenerate codons and computed their average modification efficiencies using the model. To gauge and compare the quality of different designs, we developed scoring functions M^long^ and M^short^ used for library designs with 11-mer or longer inserts and 10-mer or shorter inserts, respectively (S.I. 2.1). Both functions consider the average predicted modification efficiency and the theoretical diversity of a library. M^long^ prioritizes the former because libraries with longer inserts are inherently more diverse, while M^short^ favors the latter. In both cases, libraries that strike a balance between a higher diversity of precursor sequences and a higher thiopeptide maturation yield will have greater scores. We first optimized the design of the 11-mer insert library. Starting with a fully random (nnk)_11_ design (Figure 3a), which encodes all 20 proteinogenic amino acids in every position inside the insert, we first conducted a virtual Ala scan by replacing each nnk degenerate codon with Ala-encoding gcg (Figure 3b). Fixing an Ala residue in positions 1 and 11 and to a lesser extent in positions 2 and 10 (counting from the first insert position), the amino acids adjacent to the PTM sites had the largest effect on the predicted maturation efficiency, whereas restricting diversity in other positions had minimal impact (Figure 3c). Thus, we focused on finding the optimal combination of degenerate codons in positions 1 and 11, allowing for fully random nnk codons elsewhere inside the insert (Table S5, Figure 3d). In position 1, degenerate codons dsk, dsu, nsu, dbk, and rsu had the highest M^long^ scores. A similar search for the optimal degenerate codon in position 11 revealed dbk, nyu, nyk, nnu, and several others as high-scoring designs. We then evaluated the combinations of the identified codons in positions 1 and 11 (Figure 3d). The results pointed to dsk-(nnk)_9_-nnu as the best design, characterized by a 0.53 predicted modification efficiency [compare with 0.36 for a fully random (nnk)_11_ insert] and only a 3.8-fold decrease in peptide diversity compared to (nnk)_11_ [5.4 × 10^13^ vs 2.0 × 10^14^ peptides]. Further constraints in positions 2 and 10 did not lead to better designs according to our metrics, as the modification efficiency gains were insufficient to justify the loss of library diversity (Table S5). Therefore, dsk-(nnk)_9_-nnu was chosen as the final design for the 11-mer insert library.

Because the training data contained sequences of different lengths, the model can estimate the fitness of variable-length inserts, although in most cases such predictions are performed in the so-called “zero-shot” manner and may lose some accuracy. Thus, dsk-(nnk)_10_-nnu stood out as the best 12-mer insert design. For shorter libraries, similar optimization using M^short^ as the metric pointed to dbk-(nnk)_*n*_ (*n* = 5–9) as the highest-scoring designs (Figures S11 and S12 and Table S5).

To ascertain the accuracy of these predictions, we performed a single-round DADL assay to experimentally compare the maturation yields of several library designs. The assay indicated that the modification efficiency of dsk-(nnk)_9_-nnu was superior to those of (nnk)_11_ and our previously employed nbk-(nnk)_9_-nbk designs,^20^ validating the developed model-guided approach (Figure 3e). DNA recovery rates in the unbound fraction gauged in this assay also provide an estimate for the true library size; approximately 4% of the starting mRNA molecules display a thiopeptide after the enzymatic treatment. The use of 10^14^ mRNAs [nbk-(nnk)_9_-nbk design] for *in vitro* translation, which is the standard in many mRNA display selections,^34,35^ leads to a thiopeptide library containing 4 × 10^12^ compounds, much larger than achievable with other pseudonatural product engineering techniques. This result also indicates that during the first round of affinity selection 11- and 12-mer insert libraries are sparsely sampled, i.e., a small fraction of all encoded thiopeptides is produced, and to a first approximation, every compound is unique.

Our final library contained LazA variants of various lengths. Because we aimed to minimize the size of the displayed thiopeptides to improve their pharmacological properties (for instance, to increase their cellular uptake efficiency), we capped the insert length at 12 residues. Seven mRNA templates, each containing a fixed-length insert (6 to 12 random residues), were individually assembled and then mixed into a single library, library AS1, for downstream affinity selection (Figure 3f).

### Selections against IRAK4 and TLR10

To test the resulting library’s ability to yield high-affinity thiopeptide ligands, we performed mRNA display selection experiments against two proteins: IRAK4, a key kinase in innate inflammatory signaling pathways,^36,37^ and TLR10, an orphan receptor also implicated in innate immunity.^38−40^ These proteins, particularly IRAK4, are promising therapeutic targets to alleviate a variety of autoimmune disorders.^41−43^ The affinity selection protocol closely follows the DADL workflow (Figure S13). After streptavidin pulldown, which removes linear forms, the unbound fraction containing mature thiopeptides was first subjected to a counterselection (panning the library against magnetic beads with no immobilized protein to eliminate bead binders) and then to the pulldown against the target protein. The recovered cDNA was PCR-amplified and transcribed to mRNA for the following round. Unlike DADL, in these selections, we did not perform HA-affinity purification, and in round 1, the streptavidin pulldown step was also omitted. Given the high maturation efficiency of library AS1, we questioned whether a dedicated step to remove the linear forms was necessary. To this end, we additionally conducted selections without genetic code reprogramming (i.e, *N*^α^-biotinylated Phe was replaced with the standard *N*^α^-formyl Met as the N-terminal amino acid) and therefore without the dedicated streptavidin pulldown process [pulldown(−) selections]. This modification considerably simplifies the procedure by obviating the need for genetic code reprogramming (S.I. 2.8 and Figure S13).

**Figure 4:**
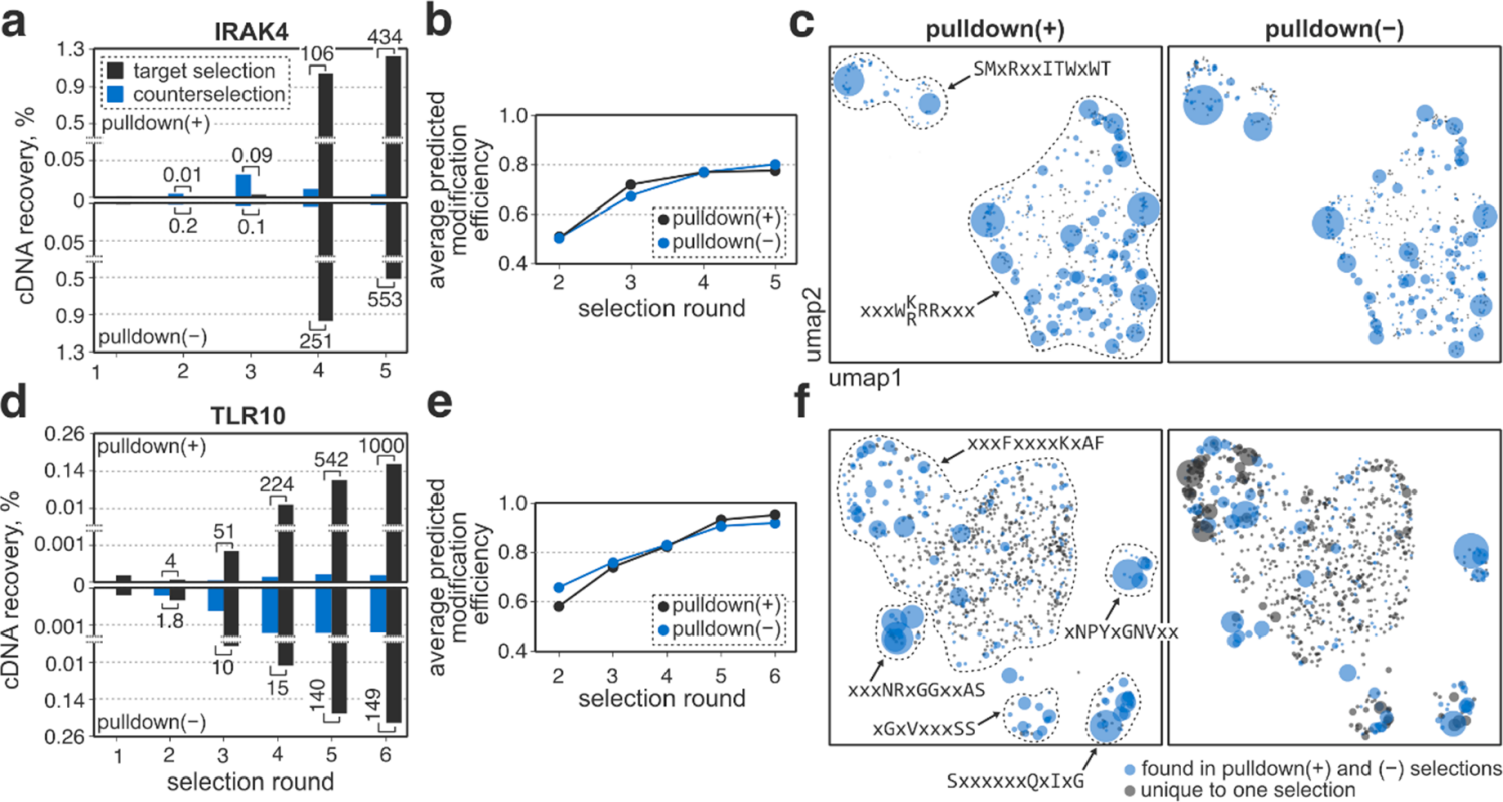
Results of the affinity selections of IRAK4 and TLR10. In each case, two experiments [pulldown(+) and pulldown(−) selections as described in the text] were performed. Panels a), b), and c) show data for IRAK4, and panels d), e), and f) show data for TLR10. a, d) Recovery of cDNA during the course of selection as measured by qPCR. Numbers above bars are the ratio of cDNA recovery between the target protein pulldown and counterselection. b, e) Library fitness (average modification efficiency of peptides in the library) as a function of selection round. In each case, the selections enriched for substrates which are efficiently converted to thiopeptides by the enzymes. c, f) Visualization of the top 1000 most abundant sequences obtained from each selection using the uniform manifold approximation and projection-based (umap) embedding. Each sequence is represented by a circle, where similar peptides appear closer to each other and more abundant entries appear larger. For both IRAK4 and TLR10, pulldown(+) and pulldown(−) selections resulted in similar peptide compositions.

All four selections progressed smoothly, and a significant increase in cDNA recovery occurred after round 4 in every case (qPCR; Figure 4a and d). In contrast, cDNA recovery in the counterselection fraction remained consistently low, suggesting the enrichment of target-binding species. For both TLR10 and IRAK4, cDNA recovery metrics pointed to only minor differences between pulldown(+) and pulldown(−) selections. An analysis of the libraries by NGS also revealed that for both target proteins the final populations contained several distinct sequence families (vide infra), with marginal differences between pulldown(+) and pulldown(−) selections. The model-assessed library fitness increased from round to round in all cases (Figure 4b and e). For example, in the TLR10 pulldown(+) selection, the average predicted modification efficiency increased from 0.59 in round 2 to 0.96 in round 6. The pulldown(−) selection experiments behaved similarly, and the final libraries consisted primarily of excellent substrates (0.80 for IRAK4 and 0.92 for TLR10). These results suggest that during the selection process macrocyclic thiopeptides outcompeted various linear forms, even without the dedicated step to remove the latter, highlighting the propensity of thiopeptides to act as protein ligands.

For both target proteins, the pulldown(+) and (−) data sets were also highly similar at the peptide level (Figure 4c and f). After five rounds of selection, the libraries in both IRAK4 selections converged to two major families, xxxW(K/R)RRxxx and SMxRxxITWxWT (insert sequences), with the former being more populous and diverse. Although the frequencies and relative ranks of peptides varied, most of the top sequences were present in both libraries. For instance, the most abundant pulldown(+) peptide absent from the pulldown(−) data set comprised only 0.008% of the library and was ranked 104th. The TLR10 selection libraries also converged, but the data sets remained more diverse compared to IRAK4. Four well-defined sequence clusters (SxxxxxxQxIxG, xxxNRxGGxxAS, xNPYxGNVxx, and xGxVxxxSS) as well as one large family with a loose consensus (xxxFxxxxKxAF) comprised the majority of both data sets. At the same time, the pulldown(−) library contained several high-ranking peptides absent from the pulldown(+) data set, most of which belonged to the latter family, including CISFWLQRKVAF, the rank 2 sequence comprising 8.1% of the pulldown(−) population (Table S6).

**Figure 5:**
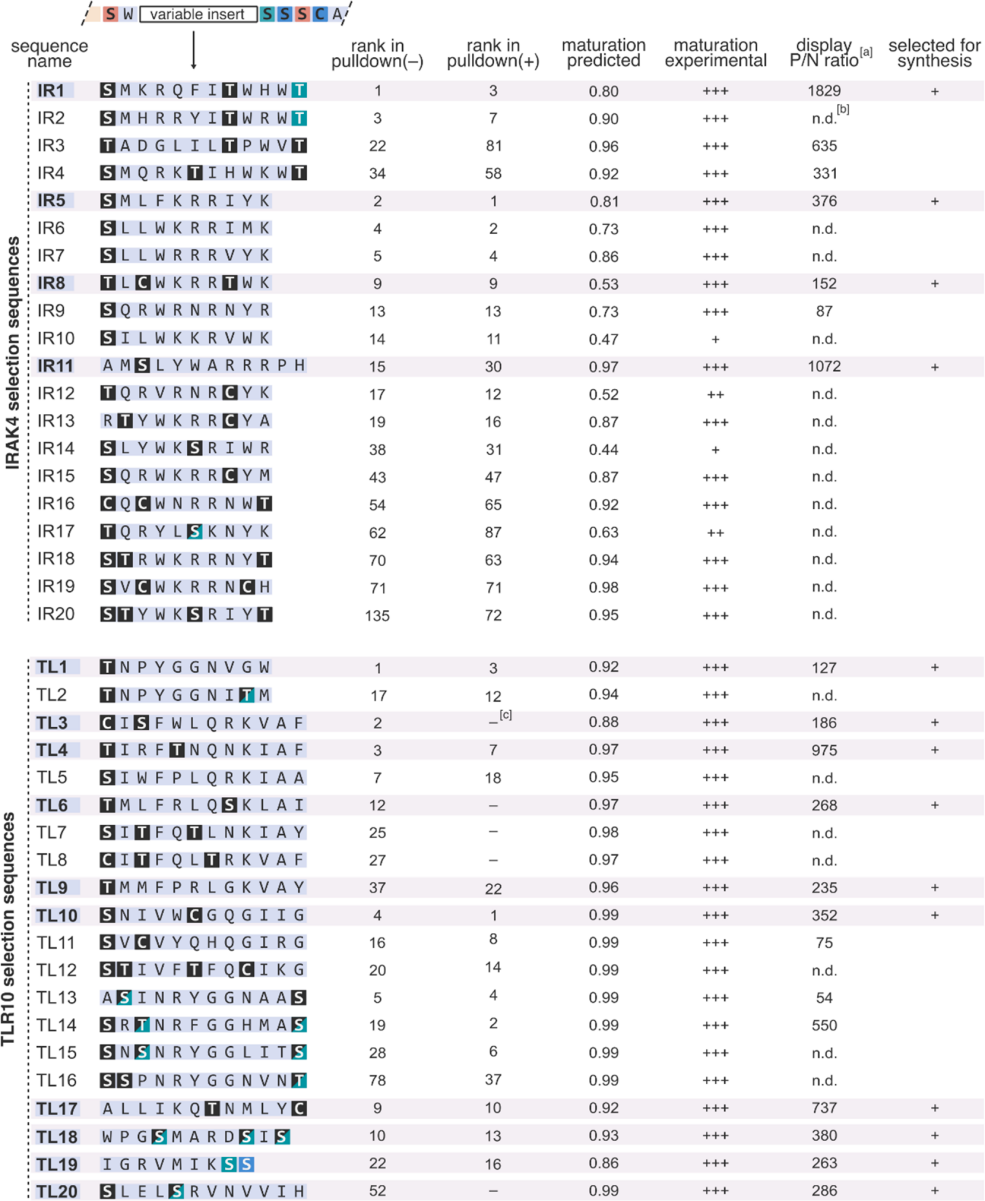
Initial analysis of the selected sequences. Indicated are random inserts (residues highlighted in black are Ser/Thr/Cys that remained intact after the enzymatic treatment; in blue, Ser/Thr/Cys modified to azoles; and in green Ser/Thr converted to dehydroalanine and dehydrobutyrine, respectively). The experimental maturation efficiency was semiquantitatively assayed by LC/MS (+++/++/+/– scale; S.I. 2.7; LC/MS chromatograms can be found in Figures S14 and S15). Annotations of individual thiopeptides in product mixtures (IR17 and TL2, 13–16, 18, 20) are summarized in Figures S16–S23. ^[a]^: the ratio of cDNA recoveries (qPCR) for the target protein pulldown over beads only in a single-round selection experiment (Figure S24). ^[b]^: n.d.: not determined. ^[c]^: sequences not found in the final pulldown(+) libraries at the sequenced depth.

For each target protein, we selected 20 sequences for further analysis, prioritizing both the peptide’s abundance in NGS data and the diversity of the resulting set. For TLR10, five selected peptides, TL3, 6, 7, 8, and 20, were found only in the pulldown(−) experiment. First, we analyzed maturation efficiencies of these precursor peptides. LazA variants (IR1–20 and TL1–20) were *in vitro* translated and treated with Laz enzymes under the selection conditions. Consistent with the model predictions, LC/MS analysis of the reaction mixtures indicated the formation of leader-NH_2_ and the corresponding thiopeptides as the major products in every case (Figure 5 and Figures S14–S23). Minor accumulation of linear forms was observed in two sequences (IR10 and IR14). These results indicate that the selected precursor peptides underwent efficient macrocyclization to thiopeptides during selection. Importantly, the five pulldown(−)-exclusive peptides were all competent substrates for Laz enzymes, suggesting that their disappearance from the pulldown(+) libraries was not due to their poor maturation. Sequences containing Ser, Thr, or Cys inside the random insert can undergo undesigned modifications inside the macrocycle, which can lead to thiopeptide product mixtures. Nevertheless, despite the presence of Cys, Ser, and/or Thr residues in the random inserts of every selected precursor, 32 out of the 40 sequences cleanly yielded a single major macrocyclic product. Notably, Ser10-Ser11 in TL19 gave rise to dehydroalanine10-oxazole11 in the corresponding thiopeptide, and Thr14 in IR1 and IR2 was also converted to dehydrobutyrine14. Finally, we semiquantitatively assayed the affinity of 7 IRAK4 and 13 TLR10 peptides toward their targets in an mRNA display experiment. As measured by qPCR, the thiopeptide-mRNA/cDNA constructs bound bead-immobilized protein targets but not the beads in every case. This result confirms the affinity of the discovered thiopeptides for their respective targets. When the enzymatic maturation of the linear precursors was omitted, no significant cDNA recovery took place, indicating the critical role of macrocyclization in binding (Figure S24).

### Thiopeptide Synthesis and Characterization

We selected 4 IRAK4- and 10 TLR10-targeting thiopeptides for chemical synthesis and biochemical characterization. To produce the requisite compounds on a multimilligram scale, we utilized our previously developed solid-phase-based strategy (synthetic schemes and procedures are in S.I. 5.1 and Figure S32).^44^ Briefly, the approach employs Fmoc/^*t*^Bu solid-phase peptide synthesis to assemble linear peptide precursors, where the pyridine-bisazole moiety is incorporated as an Fmoc-protected amino acid. After mild acidic cleavage of peptides from 2-chlorotrityl chloride resin, which leaves the side-chain protecting groups intact, the linear precursors are macrocyclized in solution. The resulting peptides are then subjected to full deprotection with trifluoroacetic acid. Oxidative elimination of the Se-alkyl selenocysteine derivatives (precursors to dehydroamino acids) furnishes the target compounds. As before, we made several modifications to the thiopeptide structures to simplify the synthesis.^44^ The changes included truncating the C-terminal tail outside the macrocycle, introducing an oxazole to thiazole mutation to simplify the synthesis of the pyridine-bisazole building block, and mutating sulfide-containing amino acids (primarily methionine) to their CH_2_-containing isosteres because the selective oxidation of selenocysteine residues in the presence of sulfides proved difficult in many cases. Thiopeptide TL19 contained a unique dehydroalanine10-oxazole11 motif which necessitated the custom synthesis of the appropriate precursor, Fmoc-^L^Sec(Ph)-Oxz-OH, accomplished in 30% yield over seven steps (S.I. 4.3). Overall, our synthetic effort produced 14 target compounds, most in milligram quantities and excellent (>95%) purity, as assayed by UPLC and LC/MS (S.I. 5.2).

**Figure 6:**
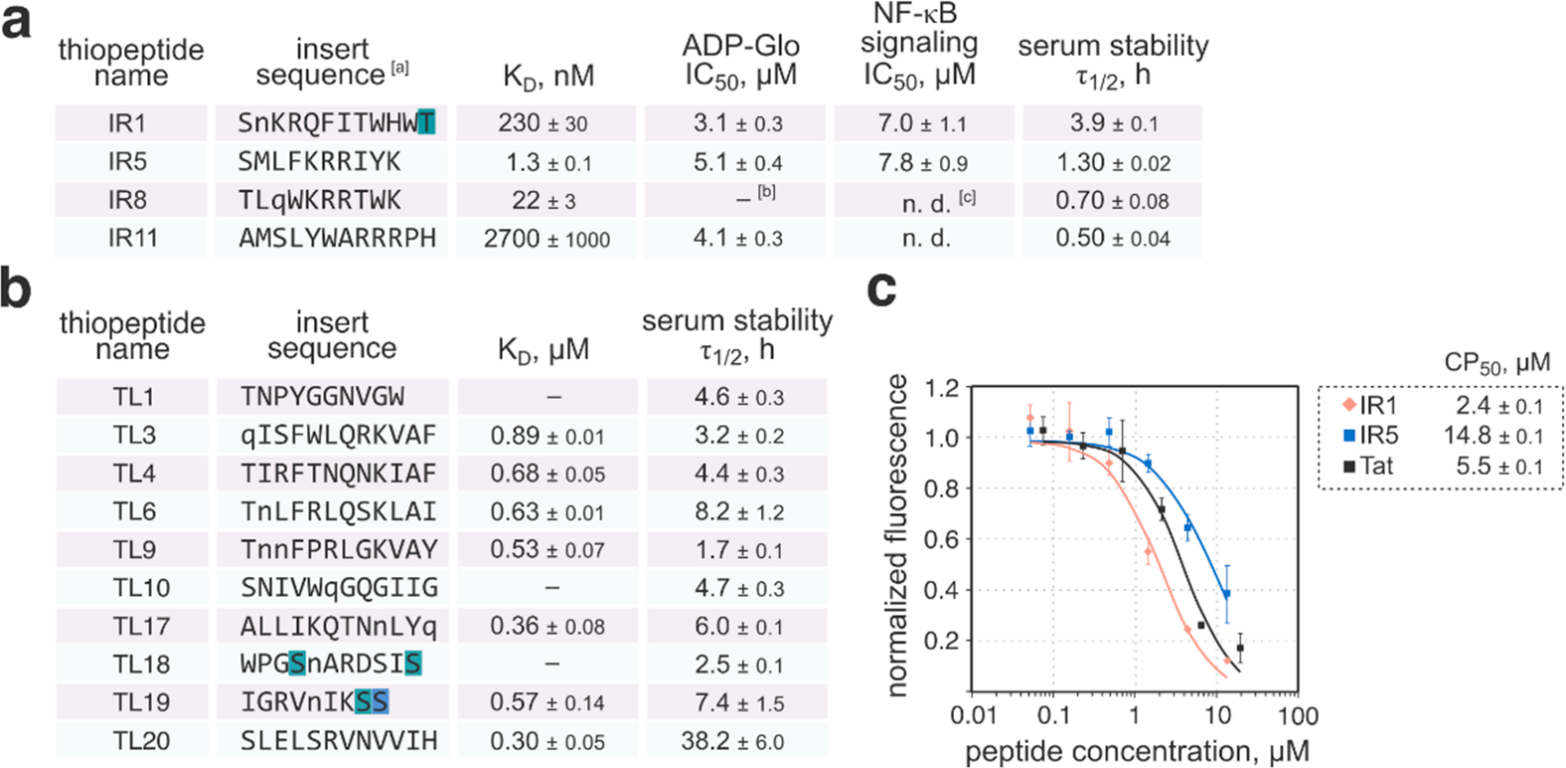
Biochemical characterization of discovered thiopeptides. a) Binding affinity (K_D_ values measured by surface plasmon resonance) to the target protein, IRAK4 ADP-Glo inhibition (IC_50_ values), cell-based NF-κB signaling inhibition (IC_50_ values), and serum stability (τ_1/2_ values) of IRAK4-targeting thiopeptides. b) Binding affinity to the target protein and serum stability of the discovered TLR10 ligands. ^[a]^: residues highlighted in green indicate dehydroalanine (S) or dehydrobutyrine (T); those highlighted in blue indicate Ser-derived oxazole; n stands for Lnorlecuine; and q stands for Lhomoglutamine (for full chemical structures, see S.I. 5.2). ^[b]^: no binding/inhibition. ^[c]^: n.d.: not determined. c) Results of the chloroalkane penetration assay for IR1 and IR5. The assay was performed in HEK293H cells as described in S.I. 2.14. IR1 internalized to the cytosol of HEK293H cells more efficiently than the known cell-penetrating peptide, Tat. CP_50_: midpoint internalization concentration.

With the compounds in hand, we quantified their affinity for the target proteins using surface plasmon resonance (Figure 6, S.I. 2.10, and Figures S25 and 26). The four synthesized IRAK4 thiopeptides (IR1, 5, 8, and 11) were all competent protein ligands. The best peptide, IR5, strongly bound to the kinase with a dissociation constant (K_D_) of 1.3 nM. For TLR10, out of the 10 synthesized thiopeptides, 7 bound to the extracellular domain of TLR10 with sub-μM affinities. The ligand association rates varied between 5 × 10^3^ and 5 × 10^4^ M^–1^ s^–1^, and in five out of seven cases, the dissociation rates were below 10^–2^ s^–1^ (Table S7). The thiopeptides selectively identified from pulldown(−) selection (TL3, 6, and 20) were among the active compounds, and TL20 was the best TLR10 ligand with a K_D_ of 0.3 μM. The three thiopeptides with no measurable association with TLR10, TL1, 10, and 18 were identified in both selections. As such, these results suggest that omitting the streptavidin pulldown step and thus allowing the linear forms to compete against thiopeptides does not compromise selection outcomes, at least for libraries with high maturation efficiencies such as library AS1. The fact that potent TLR10 ligands TL3, 6, and 20 were identified only in pulldown(−) selection also suggests that for library AS1, the streptavidin pulldown step was counterproductive because it might eliminate some true ligands.

Next, we tested the metabolic stability of the discovered thiopeptides. The compounds were incubated in human serum at 37 °C in the presence of an internal control, and the amounts of the remaining analytes were quantified by LC/MS (Figures S27 and 28). The assay revealed varying stability of the thiopeptides, where IR1, TL6, TL19, and, in particular, TL20 demonstrated high stability in serum (TL20 had a half-life, τ_1/2_, of 38 h). At the other extreme, IR11 rapidly degraded in human serum with τ_1/2_ = 0.5 h.

The role of IRAK4 as a kinase acting downstream of TLR and interleukin-1 receptors to phosphorylate IRAK1 and transduce immunological signals is well-established.^37^ IRAK4 is a confirmed therapeutic target evaluated for the treatment of several autoimmune disorders.^45−47^ Therefore, we investigated the inhibitory activity of thiopeptides IR1, 5, 8, and 11. In an ADP-Glo kinase activity assay, three compounds (IR1, 5, and 11) inhibited the enzyme with a single-digit μM IC_50_ values (3.1 to 5.1 μM; see S.I. 2.11 and Figure S29). The disparity between the binding affinity and inhibition potencies for IR1 and IR5 may suggest an allosteric mode of action, commonly observed in mRNA display-derived ligands,^48^ but the mode of inhibition was not further ascertained. We also explored the inhibition of IRAK4 in cell models using two thiopeptides with the strongest binding, inhibition, and serum stability: IR1 and IR5. We first prepared chloroalkane tag-containing derivatives, IR1-ct and IR5-ct (S.I. 5.3), and utilized them in a chloroalkane penetration assay^49^ to test their cellular uptake. Both compounds accessed the cytosol of HEK293H cells. IR1 internalized more efficiently than a known cell-penetrating peptide Tat (half-internalization concentration, CP_50_, of 2.4 vs 5.5 μM), whereas IR5 required a higher concentration for uptake (CP_50_ = 14.8 μM).^50,51^ In a cell culture model, IR1 and IR5 could also inhibit TLR signaling according to the NF-κB reporter assay (S.I. 2.13 and Figure S30). THP1-XBlue monocytes were incubated with the test compounds for 6 h, after which the TLR signaling and hence the IRAK4 function were stimulated by adding lipopolysaccharide from *Escherichia coli* K12. The activity of NF-κB-induced secreted alkaline phosphatase quantified 20 h after induction responded to the changes in the test compound concentration, indicating that IR1 and IR5 inhibited the lipopolysaccharide-induced NF-κB signaling with IC_50_ values of 7.0 and 7.8 μM, respectively. The compounds were not cytotoxic at these concentrations (assayed by Cell Counting Kit-8),^52^ suggesting that the effect was due to IRAK4 inhibition (Figure S31). Future in-depth studies will be required to decipher the mode of action of these thiopeptides.

## Conclusions

The nuanced substrate preference profiles of many RiPP enzymes necessitate the careful design of combinatorial RiPP precursor peptide libraries to maximize the chances for the successful production of RiPP analogs. In this work, we describe a methodology to optimize the design of mRNA display libraries for downstream affinity selections. Using the biosynthesis of lactazole A as a model, we developed a high-throughput platform for comprehensive profiling of the substrate preferences of the entire biosynthetic pathway. The resulting data enabled us to train an accurate neural network-based classifier capable of predicting the thiopeptide maturation efficiency from the primary sequences of a given substrate. Furthermore, we developed a methodology to leverage the resulting classifier for the design of mRNA libraries with an optimal average modification yield and high diversity. In principle, analogous pipelines can be constructed for other RiPP biosynthetic pathways so long as the enzymatic reaction products can be chemically differentiated from the starting materials and intermediates. Many RiPP pathways amenable to combinatorial workflows (lanthipeptides,^5,26^ lasso peptides,^3,53^ cyanobactins,^15,29^ graspetides,^54,55^ and spliceotides,^56,57^ among others) may benefit from comprehensive substrate profiling in an mRNA display format. Model-guided library design may be particularly useful for the pathways with enigmatic substrate preferences (e. g., prochlorosins^58,59^) or highly cooperative biosynthesis (thiopeptides^19,60^ and polytheonamides^61^), where the substrate fitness landscapes become too complex for the manual construction of optimal library designs.

For our platform, we show that the model-designed libraries not only yield high-affinity thiopeptide ligands against protein targets of choice, as demonstrated here for IRAK4 and TLR10, but also simplify our previously established thiopeptide-mRNA display pipeline. In total, our efforts produced 11 pseudonatural thiopeptides with affinities as high as K_D_ = 1.3 nM (IR5 against IRAK4). IR1 and IR5 also inhibited the target kinase, internalized into HEK293H cells, and inhibited IRAK4-mediated NF-κB signaling in monocyte cell models. This work takes a step toward streamlining the discovery of pseudonatural RiPPs with *de novo* designed biological activities and favorable pharmacological properties such as metabolic stability and cellular uptake.

## Data Availability

NGS DADL data were deposited in DDBJ (accession no. DRA016846). Source Python code for model training as well as the final model weights can be found at https://github.com/avngrdv/mRNA-display-deep-learning (accessed on September 27, 2023).

## Supporting Information Available

Experimental procedures, additional figures and tables, and the characterization of the synthesized compounds (PDF)
Assembly schemes; oligonucleotides; parser statistics; validation peptide summary; library design; top 100 selection hits; and SPR kinetic data (XLSX)
Transparent Peer Review report available (PDF)
