# Supplementary material for: Deep Learning-Driven Library Design for the De Novo Discovery of Bioactive Thiopeptides
Source: ACS Cent Sci. 2023 Nov 7;9(11):2150–60. doi: 10.1021/acscentsci.3c00957 (PMC10683472; doi:10.1021/acscentsci.3c00957)
Supplement: Supplementary file 1 — oc3c00957_si_001.pdf [file oc3c00957_si_001.pdf]

Supporting Information

Deep learning-driven library design for de novo discovery of bioactive thiopeptides

Jun Shi Chang[1], Alexander A. Vinogradov[1]*, Yue Zhang[1], Yuki Goto[1]* and Hiroaki Suga[1]*

[1]Department of Chemistry, Graduate School of Science, The University of Tokyo, Bunkyo-ku, Tokyo 113-0033, Japan

* Correspondence to hsuga@chem.s.u-tokyo.ac.jp, y-goto@chem.s.u-tokyo.ac.jp, and a_vin@chem.s.u-tokyo.ac.jp

Contents

1. General 2
2. Methods 3
   2.1. Definition 3
   2.2. mRNA library constructions 3
   2.3. DADL assay for the entire lactazole biosynthesis pathway 4
   2.4. NGS data processing and deep learning 6
   2.5. Model validation 7
   2.6. Preparation of synthetic DNA for in vitro translation 8
   2.7. Enzymatic reactions and LC/MS analysis of their outcomes 9
   2.8. Affinity selection 10
   2.9. Preparation of tRNA and aminoacylation 13
   2.10. Surface plasmon resonance 13
   2.11. Kinase inhibition assays with ADP-Glo 14
   2.12. Thiopeptide metabolic stability experiments 14
   2.13. NF-kB signaling pathway inhibition assay 14
   2.14. Chloroalkane penetration assay 15
3. Supplementary Figures 16
4. Chemical synthesis of Thiopeptides Building Blocks 43
   4.1. General remarks and Experimental Details 43
   4.2. Synthesis of dehydrobutyrine building block 45
   4.3. Synthesis of dehydroalanine-oxazole building block 48
5. Total synthesis of lactazole-like thiopeptides 52
   5.1. General procedures for thiopeptide synthesis 52
   5.2. Total synthesis of Lactazole-like thiopeptides 56
   5.3. Late-stage Lactazole-like Thiopeptide Functionalization 75
6. ^{1}H and ^{13}C NMR Spectra 79
7. Supplementary References 87

1. General

Reagents were purchased from Nacalai Tesque, Wako Pure Chemical Industries, Sigma-Aldrich Japan, or TCI Chemicals and were used as received. Biotinylated IRAK4 (kinase domain, amino acids 1-460; NP_057207.2) was purchased from Carna Biosciences (Kobe, Japan). Recombinant human TLR10 Fc Chimera protein (extracellular domain, amino acid 20-576; Q9BXR5) was purchased from R&D system (#6619-TR-050). Anti-HA magnetic beads were from Pierce Thermo Fisher Scientific (#88836). Magnetic beads with immobilized streptavidin were from Invitrogen (Dynabeads Streptavidin C1, # 65001). Magnetic beads with immobilized streptavidin were from Invitrogen (Dynabeads Streptavidin M280, #11206D). Dynabeads Protein G was purchased from Thermo Fisher.

Oligonucleotides for library assembly (HPLC and OPC purification grade for regular and randomized sequences, respectively) were purchased from GeneDesign Inc. (Osaka, Japan). Other primers were from Eurofins Genomics (OPC purification grade). All oligonucleotides were used as received. PCR amplifications were conducted in a BioER TC-96GHBC thermal cycler. qPCR analysis was performed using a LightCycler Nano instrument (Roche) running on LightCycler Nano Software v.1.0.

Protein purification and synthesis of *Streptomyces lactacystinaeus* $tRNA^{Glu}$ were done as previously described.[1–3] Protein concentration measurements were carried out on a Nanodrop 2000c spectrophotometer (Thermo Scientific) equipped with a 5 mm pathlength quartz cuvette using extinction coefficient values calculated with ExPASy ProtParam tool (https://web.expasy.org/protparam). For thiopeptides, concentrations were measured analogously using the empirically determined extinction coefficient of the central heterocyclic core (ε^{329} = 10100 $M^{-1}cm^{-1}$; measured in DMSO).[4]

THP1-Blue™NF-κB cells were purchased from InvivoGen and cultured in RPMI 1640 medium supplemented with 100 µg/mL penicillin-streptomycin, 100 µg/mL normocin, 10 µg/mL blasticidin, and 10% (v/v) fetal bovine serum (all from Thermo Fisher) in a 37 °C/5% CO_2 incubator. HEK293T cells grown analogously in DMEM (high glucose, Thermo Fisher) medium. Cell Counting Kit-8 were purchased from Dojindo Molecular Technologies.

Biochemical measurements (sections 2.10 – 2.14) were performed in triplicate or more. Most experiments were reproduced to an analogous outcome at least once. In the cases where the determination of biophysical parameters involved cell assay (CP_{50} and IC_{50}), reported are standard errors of the fit; otherwise, error bars are standard deviations.

2. Methods

2.1. Definitions

qPCR metrics: DNA recovery and specific modification. Here, and elsewhere in the text, cDNA refers to “complementary DNA”, which is the first strand product of reverse transcription for an mRNA library. cDNA recovery was defined as

$$\mathbf{r} = \frac{n_{\text{unbound}}}{n_{\text{bound}}} \tag{1}$$

where *n* is molar cDNA amount as quantified by qPCR. “Unbound” refers to the fraction of the library that was modified by the Laz enzymes. Specific modification of an mRNA display experiment is defined as

$$\text{specific modification} = \frac{\mathbf{r}_{\text{Laz enzymes}}}{\mathbf{r}_{\text{no enzyme}}} \tag{2}$$

Library design score. Various tested library designs were compared and optimized using the developed score, which factored in the loss of peptide diversity upon incomplete randomization of the precursor peptides, as well as the gains in maturation efficiency compared to the fully random library [$(nnk)_x$ insert]. For long library designs (11- and 12-mer inserts), the score was computed as follows:

$$M^{\text{long}} = 100 \cdot (\mathbf{f}_L \cdot \mathbf{m}_L - \mathbf{f}_{ref} \cdot \mathbf{m}_{ref})^{1.5} \cdot \left(\frac{\mathbf{f}_L \cdot \mathbf{d}_L}{\mathbf{f}_{ref} \cdot \mathbf{d}_{ref}}\right) \tag{3}$$

where $\mathbf{f}_L$ is the fraction of the successfully translated ORFs in library **L** (some libraries encode stop codons inside their random regions and thus produce non-substrates), $\mathbf{m}_L$ is the average model call of the library computed by the model and $\mathbf{d}_L$ is the number of peptide encoded by the design. Analogously, $\mathbf{f}_{ref}$, $\mathbf{m}_{ref}$, and $\mathbf{d}_{ref}$ are the values for the reference library (contains the fully random $(nnk)_x$ insert). For short library design (6- to 10-mer inserts) the score was modified as follows:

$$M^{\text{short}} = 100 \cdot (\mathbf{f}_L \cdot \mathbf{m}_L - \mathbf{f}_{ref} \cdot \mathbf{m}_{ref}) \cdot \left(\frac{\mathbf{f}_L \cdot \mathbf{d}_L}{\mathbf{f}_{ref} \cdot \mathbf{d}_{ref}}\right)^{1.5} \tag{4}$$

2.2. mRNA library construction

PCR assembly. DNA libraries were assembled by PCR from oligonucleotide primers (assembly schemes and primer sequences are summarized in Table S1 and S2, respectively). First, overlapping forward and reverse primers were annealed and extended in the primer extension step. To this end, 150 µL of Platinum SuperFi DNA Polymerase solution [PCR buffer (1x), SuperFi Pol (1x), 250 µM each dNTP; SuperFi PCR mix; buffer and enzyme from Thermo Fisher] containing 270 nM forward and 250 nM reverse primers was denatured at 95 °C for 60 s. Then,

three cycles of annealing (53.1 °C for 60 s) and extension (72 °C for 30 s, 0.5 °C/s) were carried out. In the next step, the extension product (150 µL) was added to SuperFi PCR mix (2850 µL) containing 500 nM forward and reverse primers, and five cycles of amplification ensued (three stage thermal cycling including a denaturing step at 95 °C for 20 s, annealing at 60 °C for 20 s, and extension at 72 °C for 30 s, 0.5 °C/s). After, the mixture was further incubated at 72 °C for 60 s, and cooled to 4 °C. The outcomes were evaluated by TapeStation (Agilent Technologies). DNA was first extracted by phenol/chloroform/isoamyl alcohol (25:24:1, saturated with 10 mM Tris (pH 8.0), 1 mM EDTA), and then by chloroform/isoamyl alcohol (24:1).

Transcription. Extracted DNA was precipitated with ethanol, washed with 70% ethanol in water (v/v), dissolved in water, and added to the transcription reaction mix [40 mM Tris buffer (pH 8.0) supplemented with 20 mM $MgCl_2$, 10 mM DTT, 1 mM spermidine, 0.01% Triton X-100, 240 nM T7 RNA polymerase, 0.04 U/µL RNasin RNase inhibitor (Promega), and 3.75 mM each NTP; 1 mL total volume]. Transcription was allowed to proceed at 37 °C for 14 h, after which 30 µL of 1 unit/µL RQ1 RNase-free DNase (Promega) was added, and the reactions were further incubated for 60 min at 37 °C. Reactions were quenched by the addition of EDTA (final concentration [f.c.]: 67 mM) and NaCl (f.c.: 270 mM). The transcripts were precipitated with isopropanol, washed with 70% ethanol (v/v), redissolved in water, and purified by 6% polyacrylamide gel electrophoresis (PAGE) containing 6 M urea. RNA extracted from the gel with 300 mM NaCl were collected by ethanol precipitation, dissolved in water, and frozen at –80 °C for storage.

Starting with round 2 of affinity selection, transcription was scaled down to a 25 µL reaction, and PAGE purification was not performed.

Puromycin ligation. For affinity selection, individual mRNA libraries (6-mer:7-mer:8-mer:9-mer:10-mer:11-mer:12-mer random inserts) were mixed in 1:1:8:8:8:8:8 molar ratios to give the resulting mRNA library AS1. All mRNA library was attached to puromycin via a Y-ligation performed with the use of T4 ligase. The reaction containing 1 µM mRNA, 1.5 µM puromycin linker and 1 µM T4 ligase in ligation buffer (40 mM Tris, pH 7.8, 10 mM $MgCl_2$, 10 mM DTT, 0.5 mM ATP in a water/DMSO mixture [8:2, v/v]) was incubated at 25 °C for 45 min. Ligated mRNA was extracted with phenol/chloroform as described above, precipitated with ethanol, washed with 70% ethanol, and redissolved in water. The outcome of puromycin ligation was judged by PAGE (6%, 6 M urea). Ligation product was diluted with water to 6 µM and frozen at –20 °C for storage. This product was used for DADL assay (section 2.3) and affinity selection (section 2.8).

2.3. DADL assay for the entire lactazole biosynthesis pathway

Translation and reverse transcription. An *in vitro* translation system was reconstituted by mixing purified ribosome, enzymes, and translation factors.[5, 6] The final reaction mixture contained 50 mM HEPES-KOH (pH 7.6), 100 mM KOAc, 2 mM GTP, 2 mM ATP, 1 mM CTP, 1 mM UTP, 20 mM creatine phosphate, 12 mM $Mg(OAc)_2$, 2 mM spermidine, 2 mM DTT, 1.5 mg/mL *E. coli* total tRNA (Roche), 1.2 µM ribosome, 0.6 µM MTF, 2.7 µM prokaryotic IF1, 0.4 µM IF2, 1.5 µM IF3, 10 µM EF-Tu, 10 µM EF-Ts, 0.26 µM EF-G, 0.25 µM RF2, 0.17 µM RF3, 0.5 µM RRF, 4 µ/mL

creatine kinase, 3 µg/mL myokinase, 0.1 µM pyrophosphatase, 0.1 µM nucleotide-diphosphatase kinase, 0.1 µM T7 RNA polymerase, 0.73 µM AlaRS, 0.03 µM ArgRS, 0.38 µM AsnRS, 0.13 µM AspRS, 0.02 µM CysRS, 0.06 µM GlnRS, 0.23 µM GluRS, 0.09 µM GlyRS, 0.02 µM HisRS, 0.4 µM IleRS, 0.04 µM LeuRS, 0.11 µM LysRS, 0.03 µM MetRS, 0.68 µM PheRS, 0.16 µM ProRS, 0.04 µM SerRS, 0.09 µM ThrRS, 0.03 µM TrpRS, 0.02 µM TyrRS, 0.02 µM ValRS, 500 µM each proteinogenic amino acid and 1.2 µM puromycin-conjugated mRNA library n8 or n11 . Biotin-Phe-$tRNA^{ini}$ (f.c.: 50 µM) was prepared as described in section 2.9 and was added to the mixture.

The resulting solutions were first incubated at 37 °C for 40 min, and then at 25 °C for 10 min before adding EDTA (pH 8.0) to a final concentration of 17 mM. Quenched translation reactions were further incubated at 37 °C for 10 min to ensure complete dissociation of mRNA from the ribosome. Reverse transcription was performed with MMLV (H–) reverse transcriptase (Promega) using v.t.3.l4.R44 primer (sequence in Table S2) at 42 °C for 60 min following manufacturer's protocol (Tris 50 mM, pH 8.3, 2 mM $MgCl_2$).

Maturation with Laz enzymes. To one volume of ice-cold reverse transcription product, 1 volume of LazDEF enzyme premix, which contained 3 µM LazD, 4 µM LazE and 4 µM LazF in 100 mM Tris buffer (pH 8.0) supplemented with 20 mM $MgCl_2$, 9 mM ATP, and 2 mM DTT, was added on ice. The mixture was transferred to a 25° C thermostat and incubated for 2 h (selection rounds 1-2, antiselection 1-6) or 1.5 h (selection round 3) or 1 h (selection round 4-6). The tubes were then placed on ice, and a solution of iodoacetamide in water was added (f.c.: 5 mM). After 5 min on ice, 1 volume of LazBCF enzyme premix [4 µM LazB, 8 µM LazC, 2.2 µM LazF, 2 µM *S. lividans* GluRS, and 40 µM *S. lactacystinaeus* $tRNA^{Glu}$ in 50 mM Tris buffer (pH 8.0) supplemented with 10 mM $MgCl_2$, 4.5 mM ATP, and 0.5 mM glutamic acid] was added. The mixture was further incubated at 25 °C for 6 h (selection rounds 1-2, antiselection 1-6) or 4.5 h (selection round 3) or 3 h (selection round 4-6).

Library purification. Prior to HA-affinity purification, one volume of 2x blocking buffer [TBS-T supplemented with 2 mg/mL bovine serum albumin and 2 mg/mL yeast RNA, where TBS-T contained 50 mM Tris, 150 mM NaCl, 0.2% (v/v) tween-20, pH 7.6] was added to one volume of the library mixture. Anti-HA beads (17 µl of 10 mg/mL bead slurry per pmol of library mRNA) were washed twice with TBS-T, once with 1x blocking buffer and added to the sample. Incubation at 4 °C for 60 min ensued, after which the supernatant was discarded, and the beads were washed thrice with TBS-T. Bound peptide-mRNA/cDNA chimeras were eluted from the beads with HA peptide (2 mg/mL in TBS-T; sequence: NH_2-YPYDVPDYA-$CONH_2$) by incubating the suspension at 37 °C for 15 min and collecting the supernatant. The elution step was repeated once more, and the supernatants were combined. HA-affinity purification was indispensable to remove peptide-unconjugated and puromycin-unligated mRNA/cDNA, chimeras displaying frameshifted peptides, and other translation side-products.

Streptavidin pulldown. Biotinylated peptide–mRNA/cDNA chimeras (i.e., the substrates that were partially modified) were separated from non-biotinylated ones (i.e., those which were fully

modified) with the streptavidin pulldown. To one volume of the sample from above, one volume of 2x blocking buffer was added. Streptavidin C1 Dynabeads (17 µl of 10 mg/mL bead slurry per pmol of library mRNA) were washed twice with TBS-T, once with 1x blocking buffer and added to the sample. Incubation at 4 °C for 10 min ensued, after which the supernatant was collected as “unbound” fraction, and the beads were washed twice with TBS-T containing 1 M urea and then once with TBS-T. Elution of the “bound” cDNA was carried out by heating the beads suspended in elution buffer [SuperFi PCR buffer (1x), 250 µM each dNTP, 250 nM T7g10M.F46 and v.t.3.l4.R44 primers] at 95 °C for 5 min.

PCR amplification. Concentrations of recovered cDNA, as well as the cDNA recovery rate as reported in Fig. 2a were determined by qPCR. Recovered sample aliquots were amplified [10 mM Tris (pH 8.4), 50 mM KCl, 0.1% (v/v) Triton X-100, 2.5 mM $MgCl_2$, 250 µM each dNTP supplemented with 250 nM T7g10M.F46/ v.t.3.l4.R44 primers, Taq DNA polymerase (1x) and Sybr Green I (1x; Thermo Fisher)] and the outcomes were analyzed against a six-point calibration curve generated with a naïve n8 or n11 library cDNA sample of known concentrations.

Thermal cycling to recover library DNA was performed based on the outcomes of qPCR to avoid cDNA overamplification. SuperFi Pol (f.c.: 1x) was added to the elution product, and three stage thermal cycling ensued (denaturing at 95° C for 15 s, annealing at 61° C for 15 s, and extension at 72° C for 15 s), after which DNA isolation, transcription and puromycin ligation steps were performed as described in section 2.2.

NGS sequencing. Tailed PCR was used to install Rd1 and Rd2 adapter sequences to library 5’ and 3’-ends, respectively. cDNA recovered as described above was PCR-amplified in the SF PCR mix with appropriate primers (primers lists can be found in Table S2). The product was carried forward to the second PCR step, which used SuperFi Polymerase and Nextera XT v3 Set primers (sequences from Illumina) to install sequencing barcodes on each sample. The success of PCR was evaluated by 3% agarose gel electrophoresis and TapeStation. After, PCR products were combined and column-purified with a NucleoSpin kit (TaKaRa) adhering to manufacturer’s protocol. Concentration of the sample was measured with Qubit (Thermo Fisher) using the dsDNA BR kit. cDNA was then appropriately diluted and denatured with 200 mM NaOH per Illumina’s protocol. Denatured library [10 pM containing 10–50% (mol/mol) PhiX Control v3 (Illumina)] was sequenced on Illumina’s MiSeq instrument in the single read 1x151 cycle mode using v3 chip, collecting data in the .fastq format. NGS results were deposited to DDBJ (Accession No. DRA016846).

2.4. NGS data processing and deep learning

.fastq parser. Original python code can be found at *https://github.com/avngrdv/mRNA-display-deep-learning* and *https://github.com/avngrdv/FastqProcessor*. Briefly, .fastq data files containing NGS base calls were parsed to retrieve DNA sequences, which were in silico translated. The resulting peptide lists were filtered to discard sequences of incorrect length, ORFs missing stop codons, peptides containing ambiguous symbols, or not conforming to the library design

criteria (i.e., those not having fixed sequences flanking the random region on either side). Additionally, the presence of the intact modification sites in LazA core peptide (Ser1 and the downstream Ser-Ser-Ser-Cys motif) was asserted, and constant region sequences were trimmed. Finally, for each remaining entry, sequencing Q scores corresponding to the variable region were inspected, and the reads containing any Q scores below 30 were discarded. The final datasets were obtained by concatenating peptide lists from several sequencing runs.

Data preprocessing. To prepare training and test datasets, selection and antiselection peptide lists for n8 and 11 library experiments were merged in the following way. First, the lists were demultiplexed by removing intraset duplicate sequences, and then the entries appearing in both selection and antiselection datasets were discarded altogether. The resulting peptide lists were compared against the validation peptide set (Table S4). Any entry within Hamming distance ≤ 2 from any of the respective validation peptides was discarded. The remaining datasets were labelled (all selection peptides labelled as 1, antiselection as 0), merged, shuffled, and split into train and test sets. To ensure that the model can operate on variable length peptides, the sequences were randomly padded (peptide list dimensions: [12, n], where n is the total number of peptides in the list).

Finally, the peptides were represented as matrices of extended-connectivity fingerprints (ECFPs) for model training.[7] To this end, an ECFP feature matrix was generated using chemical structures of component amino acids. ECFPs were generated with *max_radius* = 4 in both cases (rdkit 2021.03.3 implementation; http://www.rdkit.org), which resulted in the matrix of dimensions (20, 208). One-hot encoded peptide matrices [dimensions: (12, 20)] were then multiplied by the corresponding feature matrix to produce the final representations [dimensions: (12, 208)].

Model description and training. A neural network containing Conv1D layers with batch normalization and of different kernel sizes and dilation rates (overall model architecture as previously reported)[8] was constructed in tensorflow 2.4.1. The final models contained $1.19 \cdot 10^7$ parameters and utilized $8.47 \cdot 10^6$ peptides for training. The resulting model weights can be found at *https://github.com/avngrdv/mRNA-display-deep-learning*.

The Adam optimizer[9] with parameters $\beta_1 = 0.9$, $\beta_2 = 0.98$ and $\varepsilon = 10^{-9}$ was used for training. The learning rate was varied during the course of training following the Noam schedule with *warmup_steps* = 4000.[10] Dropout[11] (0.25) was applied to regularize the data, and batch size was set to 2048. Binary cross entropy was used as the loss function, and the training was allowed to proceed either for 300 epochs or until validation loss stopped improving (no decrease in 10 epochs). The model training took approximately 12 h on a single GeForce RTX3090 GPU (Nvidia).

2.5. Model validation

Design of validation set peptides. Like previously reported[8], we opted for a semi-random sampling to select a set that uniformly covers the expected fitness of the substrates. Briefly, 100 peptides were in silico generated. Overly hydrophobic (>5 hydrophobic amino acids) or positively charged (>3 positively charged amino acids) peptides as well as the substrates containing multiple

Cys residues were discarded for practical reasons. The remaining peptides were sorted by their statistically expected fitness (S scores; see ref. 8 for the technical definition), binned and sampled. Overly repetitive sequences were discarded, and MVP1-10 validation peptides were appended to their respective lists to give the final validation sets, which are summarized in Table S4.

Validation experiment. The absolute quantification of experimental modification efficiency using LC-MS is challenging for the following reasons:

i. Thiopeptide ionization efficiency is variable for different peptides sequences, and as such, the absolute quantification of the products in the absence of a calibration curve is unreliable.
ii. Quantification of the amounts of leader-NH_2 produced during maturation does not account for the peptide expression level, which is peptide-dependent and although can be quantified, the process is laborious and not scalable.

Thus, we studied the maturation efficiency of the precursor peptides using the following two methods: (i) LC/MS assay (described in section 2.7) to qualitatively evaluate the efficiency of macrocyclization and (ii) a single-round DADL assay to semi-quantitatively assess the maturation yields for individual clones/libraries (MVP1-10 and 5 library designs in Fig 3e). The single-round DADL assay procedures were as described in section 2.3; the amounts of pulled down cDNA were quantified by qPCR. The specific modification values (section 2.1) were used for the numerical comparison of modification efficiencies.

2.6. Preparation of synthetic DNA for in vitro translation

PCR. Linear double-stranded DNA containing a T7 promoter sequence and ORFs encoding individual precursor peptides were assembled by PCR from synthetic single-stranded DNA oligonucleotides using Taq polymerase. All PCR were performed in 10 mM Tris (pH 8.4), 50 mM KCl, 0.1% (v/v) Triton X-100, 2.5 mM $MgCl_2$, 250 µM each dNTP supplemented with 500 nM of appropriate primers and Taq DNA polymerase. Three stage thermal cycling included a denaturing step at 95 °C for 40 s, annealing at 57.5° C for 40 s, and extension at 72° C for 40 s. The list of all oligonucleotides and assembly schemes can be found in Tables S1 and S2.

In a one-step PCR assembly, template DNA (1:20000 dilution) was amplified with appropriate primers in Taq PCR solution for 13 cycles.

In a two-step PCR assembly, template DNA (1:2000 dilution) was first amplified in Taq PCR solution for 9 cycles, followed by a 13-cycle amplification of the PCR product from the first step (1 µL in 200 µL Taq PCR solution). Assembly outcomes were analyzed by 3% agarose gel electrophoresis stained with ethidium bromide. DNA was isolated following phenol/chloroform extraction and ethanol precipitation steps as in section 2.2. Templates prepared in this way were used for in vitro translation without concentration adjustment or further purification.

2.7. Enzymatic reactions and LC/MS analysis of their outcomes

In vitro translation and enzymatic reactions. Template DNA encoding individual library clones were added (20% v/v) to the transcription-coupled in vitro translation premix described in section 2.3; genetic code reprogramming was not performed in these experiments, and additionally, for *lazA* variants bearing an amber stop codon (TAG), the reaction mixture was supplemented with 1 µM release factor-1.

Translation was allowed to proceed at 37 °C for 30 min, after which the mixtures were transferred on ice. The LazDEF/BCF two-step enzymatic treatment was identical to that described in section 2.3. To stop the reactions, the tubes were transferred on ice, and one volume of 30 mM iodoacetamide in methanol was added. The mixtures were incubated on ice for 15 min, followed by a 25 °C incubation for another 10 min. The samples were then centrifuged (15300 g for 5 min), and 2–8 µL of the supernatant was analyzed by LC-MS.

LC-MS analysis. Reaction outcomes were analyzed using Waters Xevo G2-XS QTof instrument equipped with Acquity I-Class UPLC system. HPLC was done on an Acquity UPLC Peptide BEH C4 column [dimensions: 150 x 2.1 mm; pore size: 300Å ; particle size: 1.7 µm] or an analogous C18 column using 0.1% (v/v) formic acid (FA) in water (solvent A) and 0.1% (v/v) FA in acetonitrile (solvent B) as a mobile phase. Analysis was performed at 60 °C and 300 µL/min flow rate with the methods listed below.

Method 1

0 – 2 min	1% B
2 – 17 min	1 – 61% B, linear ramp
17 – 18.5 min	95% B
18.5 – 22 min	1% B

MS analysis was done in a positive polarity/high sensitivity mode with a 0.3 s scan time. Capillary voltage was set to 700 V; ESI source and desolvation temperatures were 120 and 400 °C, respectively. Manufacturer-supplied [Glu1]-Fibrinopeptide B or Leu-enkephalin were used as a lockspray standard for continuous mass axis referencing, and the lockspray setup procedure was performed according to the manufacturer's instructions prior to every run.

LC-MS data was analyzed with MassLynx v.4.1. To analyze the efficiency of macrocyclization and the formation of various linear forms broad range extracted ion current (^{br}EIC) chromatograms were generated as previously reported[1] with *m/z* ±100 (±500 Da) tolerance window. Briefly, despite methanol precipitation, significant interference by the FIT system-derived small molecules in total ion current chromatograms was frequently observed. However, in general, the interfering compounds had a low molecular weight (<1500 Da), and thus, little interference in the *m/z* region above 1000, where most studied peptides were detected (z=5 in most cases), was observed.

Generating ^{br}EIC chromatograms for translated peptides and their reaction products at *z*=5 with *m/z* ±100 (±500 Da) tolerance window enabled visualization of reaction outcomes with minimal interference from the translation components. To analyze the formation of thiopeptide products, a combination of ^{br}EIC, biggest peak intensity (BPI) and narrow range EIC (^{nr}EIC) chromatograms corresponding to plausible structures were utilized in tandem. Reported are combined ^{nr}EIC chromatograms for all observed thiopeptide products, each generated with target *m/z* ±2 value cutoff.

2.8. Affinity selection

Translation and reverse transcription. An *in vitro* translation system was reconstituted by mixing purified ribosome, enzymes, and translation factors.[5, 6] The final reaction mixture contained 50 mM HEPES-KOH (pH 7.6), 100 mM KOAc, 2 mM GTP, 2 mM ATP, 1 mM CTP, 1 mM UTP, 20 mM creatine phosphate, 12 mM $Mg(OAc)_2$, 2 mM spermidine, 2 mM DTT, 1.5 mg/mL *E. coli* total tRNA (Roche), 1.2 µM ribosome, 0.6 µM MTF, 2.7 µM prokaryotic IF1, 0.4 µM IF2, 1.5 µM IF3, 10 µM EF-Tu, 10 µM EF-Ts, 0.26 µM EF-G, 0.25 µM RF2, 0.17 µM RF3, 0.5 µM RRF, 4 µ/mL creatine kinase, 3 µg/mL myokinase, 0.1 µM pyrophosphatase, 0.1 µM nucleotide-diphosphatase kinase, 0.1 µM T7 RNA polymerase, 0.73 µM AlaRS, 0.03 µM ArgRS, 0.38 µM AsnRS, 0.13 µM AspRS, 0.02 µM CysRS, 0.06 µM GlnRS, 0.23 µM GluRS, 0.09 µM GlyRS, 0.02 µM HisRS, 0.4 µM IleRS, 0.04 µM LeuRS, 0.11 µM LysRS, 0.03 µM MetRS, 0.68 µM PheRS, 0.16 µM ProRS, 0.04 µM SerRS, 0.09 µM ThrRS, 0.03 µM TrpRS, 0.02 µM TyrRS, 0.02 µM ValRS, 500 µM each proteinogenic amino acid and 1.2 µM puromycin-conjugated library AS1.

In round 1, translation was carried out on a 150 µL scale (180 pmol of mRNA; 10^{14} molecules). The mixture was additionally supplemented with 100 µM 10-formyltetrahydrofolate (10-CHO-THF) and no genetic code reprogramming was performed. In round 2 and henceforth, translation was scaled down to a 5 µL scale. In addition, for selection with pulldown, from round 2 10-CHO-THF was omitted, and instead biotin-Phe-tRNA^{ini} (f.c.: 50 µM) prepared as described in section 2.9 was added to the mixture.

The resulting solutions were first incubated at 37 °C for 40 min, and then at 25 °C for 10 min before adding EDTA (pH 8.0) to a final concentration of 17 mM. Quenched translation reactions were further incubated at 37 °C for 10 min to ensure complete dissociation of mRNA from the ribosome. Reverse transcription was performed with MMLV (H–) reverse transcriptase (Promega) using v.t.3.l4.R44 primer (sequence in Table S2) at 42 °C or 60 min following manufacturer's protocol (Tris 50 mM, pH 8.3, 2 mM $MgCl_2$).

Maturation with Laz enzymes. To one volume of ice-cold reverse transcription product, 1 volume of LazDEF enzyme premix, which contained 3 µM LazD, 4 µM LazE and 4 µM LazF in 100 mM Tris buffer (pH 8.0) supplemented with 20 mM $MgCl_2$, 9 mM ATP, and 2 mM DTT, was added on ice. The mixture was transferred to a 25 °C thermostat and incubated for 2 h. The tubes were then placed on ice, and a solution of iodoacetamide in water was added (f.c.: 5 mM). After 5 min on ice, 1 volume of LazBCF enzyme premix [4 µM LazB, 8 µM LazC, 2.2 µM LazF, 2 µM *S.*

lividans GluRS, and 40 µM *S. lactacystinaeus* $tRNA^{Glu}$ in 50 mM Tris buffer (pH 8.0) supplemented with 10 mM $MgCl_2$, 4.5 mM ATP, and 0.5 mM glutamic acid] was added. The mixture was further incubated at 25 °C for 6 h.

IRAK4 affinity pulldown. The tubes were transferred back on ice, and 0.1 volume of 10x blocking buffer [TBS-T supplemented with 20 mg/mL bovine serum albumin and 20 mg/mL yeast total RNA, where TBS-T contained 50 mM Tris, 150 mM NaCl, 0.5% (v/v) tween-20, pH 7.6] was added. Next, in selection with pulldown, linear forms (shunt products and partially modified TPPs maintaining the LP, i.e., N-terminally biotinylated species) were eliminated prior to IRAK4 pulldown. To this end, M280 streptavidin Dynabeads (5 µL of 10 mg/mL bead slurry per pmol of library mRNA) were washed twice with TBS, once with 1x blocking buffer and added to the sample. After a brief 2 min incubation at room temperature, the supernatant was recovered, and the procedure was repeated twice more with fresh beads. During the first round of selection, this pulldown was not performed.

The resulting samples were then subjected to a counterselection (also not performed during the first round). A 1:1 mixture of M280 streptavidin Dynabeads and the same beads bound to D-biotin (prepared by incubating the beads with a saturating concentration of D-biotin at 4 °C for 10 min) was washed twice with TBS-T, once with 1x blocking buffer and added to the libraries (0.8 µL of 10 mg/mL bead slurry per pmol of library mRNA). Incubation at 4 °C for 30 min ensued, after which the supernatant was recovered and added to IRAK4-immobilized M280 streptavidin Dynabeads (0.5 µL of 10 mg/mL bead slurry per pmol of library mRNA; prepared by agitating the beads with 1 µM IRAK4 in TBS-T at 4 °C for 10 min and washing thrice with TBS-T). The mixtures were nutated at 4 °C for 30 min, after which the beads were washed thrice with TBS-T. Elution of captured cDNA was conducted by heating the beads suspended in elution buffer [SuperFi PCR buffer (1x), 250 µM each dNTP, 250 nM T7g10M.F46 and v.t.3.l4.R44 primers] at 95 °C for 5 min.

TLR10 affinity pulldown. Similar to IRAK4 pulldown, after maturation with Laz enzyme, the tubes were transferred back on ice, and 0.1 volume of 10x blocking buffer [PBS-T supplemented with 20 mg/mL bovine serum albumin and 20 mg/mL yeast total RNA, where PBS-T contained 2.7 mM KCl, 140 mM NaCl, 10mM Na_3PO_4, 0.5% (v/v) tween-20, pH 7.6] was added. Next, in selection with pulldown, linear forms (shunt products and partially modified TPPs maintaining the LP, i.e., N-terminally biotinylated species) were eliminated prior to TLR10 pulldown. To this end, M280 streptavidin Dynabeads (5 µL of 10 mg/mL bead slurry per pmol of library mRNA) were washed twice with PBS-T, once with 1x blocking buffer and added to the sample. After a brief 2 min incubation at room temperature, the supernatant was recovered, and the procedure was repeated twice more with fresh beads. During the first round of selection, this pulldown was not performed.

The resulting samples were then subjected to a counterselection (also not performed during the first round). A 1:1 mixture of Protein G Dynabeads and the same beads immobilized with human IgG1 Fc (prepared by incubating the beads with a saturating concentration of human IgG1 Fc at

4 °C for 30 min) was washed twice with PBS, once with 1x blocking buffer and added to the libraries (0.8 µL of 10 mg/mL bead slurry per pmol of library mRNA). Incubation at 4 °C for 30 min ensued, after which the supernatant was recovered and added to TLR10-immobilized Protein G Dynabeads (0.5 µL of 10 mg/mL bead slurry per pmol of library mRNA; prepared by agitating the beads with 0.5 µM TLR10 in PBS-T at 4 °C for 10 min and washing thrice with PBS-T). The mixtures were nutated at 4 °C for 30 min, after which the beads were washed thrice with PBS-T. Elution of captured cDNA was conducted by heating the beads suspended in elution buffer [SuperFi PCR buffer (1x), 250 µM each dNTP, 250 nM T7g10M.F46 and v.t.3.l4.R44 primers] at 95 °C for 5 min.

PCR amplification. Concentrations of recovered cDNA were determined by qPCR. Recovered sample aliquots were amplified [10 mM Tris (pH 8.4), 50 mM KCl, 0.1% (v/v) Triton X-100, 2.5 mM $MgCl_2$, 250 µM each dNTP supplemented with 250 nM T7g10M.F46/ v.t.3.l4.R44 primers, Taq DNA polymerase (1x) and Sybr Green I (1x; Thermo Fisher)] and the outcomes were analyzed against a six-point calibration curve generated with a naïve AS1 library cDNA sample of known concentrations.

Thermal cycling to recover library DNA was performed based on the outcomes of qPCR to avoid cDNA overamplification. SuperFi Pol (f.c.: 1x) was added to the elution product, and three stage thermal cycling ensued (denaturing at 95 °C for 15 s, annealing at 61 °C for 15 s, and extension at 72 °C for 15 s), after which DNA isolation, transcription and puromycin ligation steps were performed as described in section 2.2.

NGS. Tailed PCR was used to install Rd1 and Rd2 adapter sequences to library 5' and 3'-ends, respectively. cDNA recovered after the selection was PCR-amplified in the SF PCR mix with appropriate primers (primers lists can be found in Table S2). The product was carried forward to the second PCR step, which used SuperFi Polymerase and Nextera XT v3 Set primers (sequences from Illumina) to install sequencing barcodes. The success of PCR was evaluated by 3% agarose gel electrophoresis and TapeStation. After, PCR product was column-purified with a NucleoSpin kit (TaKaRa) adhering to manufacturer's protocol. The concentration of the sample was measured with Qubit (Thermo Fisher) using the dsDNA BR kit. cDNA was then appropriately diluted and denatured with 200 mM NaOH per Illumina's protocol. Denatured library [10 pM containing 10–50% (mol/mol) PhiX Control v3 (Illumina)] was sequenced on Illumina's MiSeq instrument in the single read 1x151 cycle mode using v3 chip, collecting data in the .fastq format.

.fastq parser. See also section 2.4. Briefly, .fastq data files containing NGS base calls were parsed to retrieve DNA sequences, which were in silico translated. The resulting peptide list was filtered to discard sequences of incorrect length, ORFs missing stop codons, peptides containing ambiguous symbols, and overly mutated sequences (those which had the constant regions [leader and the C-terminal fraction of core peptides] with more than 5 mutations). Finally, for each remaining entry, constant regions were trimmed (leaving only random insert sequences), and

sequencing Q scores corresponding to the variable region were inspected, discarding reads containing any Q scores below 20.

Data analysis. Sequences in the resulting peptide list were counted, and the top 100 most abundant entries (Table S6) were used for multiple sequence alignment. To visualize sequence convergence, the top 1000 peptides were represented as matrices of extended-connectivity fingerprints and embedded with umap.[7, 12]

2.9. Preparation of tRNA and aminoacylation

$tRNA^{ini}$ and eFx were prepared by in vitro transcription with T7 RNA polymerase from DNA templates assembled by PCR as previously described.[1] The list of all oligonucleotides and assembly schemes can be found in Tables S1 and S2. PCR products were extracted by phenol/chloroform/isoamyl alcohol (25: 24: 1, saturated with 10 mM Tris (pH 8.0), 1 mM EDTA), chloroform/isoamyl alcohol (24: 1), and precipitated with ethanol. DNA were redissolved in water and added to the transcription reaction mix (40 mM Tris buffer (pH 8.0) supplemented with 22.5 mM $MgCl_2$, 10 mM DTT, 1 mM spermidine, 0.01% Triton X-100, 120 nM T7 RNA polymerase, 0.04 U/µL RNasin RNase inhibitor, and 3.75 mM each NTP). For $tRNA^{ini}$ transcription, 5 mM GMP was additionally supplied to the reaction mixture. Transcriptions were conducted at 37 °C for 12–16 h on a 2 mL scale. After, 60 µL of 1 unit/µL RQ1 RNase-free DNase (Promega) was added, and the reactions were further incubated for 60 min at 37 °C. The transcripts were precipitated with isopropanol, redissolved in water, and purified by 8% ($tRNA^{ini}$) or 12% (eFx) polyacrylamide gel containing 6 M urea. RNA extracted from the gel with 300 mM NaCl were collected by ethanol precipitation followed by centrifugation (15300 g for 15 min) and dissolved in water for storage.

$tRNA^{ini}$ was aminoacylated with eFx using biotin-Phe-CME as a substrate. $tRNA^{ini}$ (25 µM) and eFx (25 µM) were incubated with biotin-Phe-CME (5 mM) in HEPES-KOH buffer (50 mM; pH 7.5) containing 600 mM $MgCl_2$ on ice for 2 h. The reactions were stopped with the addition of 300 mM NaOAc (pH 5.2), and precipitated with ethanol. Precipitated RNA was recovered by centrifugation (15300 g for 15 min). The pellet was washed with 70% ethanol in water (v/v) containing 100 mM NaOAc (pH 5.2) and used in translation.

2.10. Surface plasmon resonance

Binding affinities between the thiopeptides and IRAK4/TLR10 were measured with a Biacore 8K instrument (GE Healthcare) at 25 °C. The assay buffer for IRAK4 binding analysis contained 50 mM Tris (pH 7.5), 150 mM NaCl, 10 mM $MgCl_2$, 0.05% (v/v) tween-20 and 0.1% (v/v) DMSO. The assay buffer for TLR10 binding analysis contained 2.7 mM KCl, 140 mM NaCl, 10 mM Na_3PO_4, 0.5% (v/v) tween-20 (pH 7.6).

Biotinylated IRAK4 was immobilized on a Biacore CAP chip (GE Healthcare) to a surface density of 850-1400 response units following the standard immobilization protocols provided by the manufacturer. The interaction sensorgrams were measured as single-cycle kinetics performed with 8 concentrations of the ligand sequentially flown over the chip at a flow rate of 30 µL/min

allowing for 120 s association and 60 s for dissociation between the steps. The final dissociation step was allowed to proceed for 480 s. Kinetic parameters and affinities of the interaction were determined assuming the standard 1:1 binding model using Biacore Insight Evaluation Software v.3.0 (GE Healthcare).

Human TLR10 Fc Chimera protein was immobilized on a Biacore CM5 chip (GE Healthcare) to a surface density of 2300-6900 response units following the standard immobilization protocols provided by the manufacturer. The interaction sensorgrams were measured as single-cycle kinetics performed with 8 concentrations of the ligand sequentially flown over the chip at a flow rate of 30 μL/min allowing for 120 s association and 60 s for dissociation between the steps. The final dissociation step was allowed to proceed for 120 s. Kinetic parameters and affinities of the interaction were determined assuming the standard 1:1 binding model using Biacore Insight Evaluation Software v.3.0 (GE Healthcare).

2.11. Kinase inhibition assays with ADP-Glo

The ADP-Glo assay system was purchased from Promega. The kinase reaction buffer contained 40 mM Tris (pH 7.5), 20 mM $MgCl_2$, 0.1mM Na_3VO_4, and 0.01% (v/v) triton X-100. IRAK4 (25 nM) was incubated with various concentrations of analyte inhibitors in kinase reaction buffer at 25 °C for 10 min. After, the reactions were initiated by the addition of ATP and substrate peptide (ERM peptide, sequence: NH_2-AGAGRDKYKTLRQIRK-$CONH_2$;[ref 13] synthesized using the standard Fmoc SPPS techniques). The final mixtures containing 10 nM IRAK4, 30 μM ATP, 20 μM substrate peptide and variable concentration of the inhibitors were incubated at 25 °C for 120 min. The reactions were stopped by the addition of ADP-Glo reagent (1 volume), and the luminescent signal was developed following the manufacturer's instructions. Luminescence was recorded on a Tecan M1000 PRO microplate reader (Tecan Group) with 1 s acquisition time and no signal attenuation. IRAK4 kinase activity was normalized to a no inhibitor control, and IC_{50} values were obtained from non-linear regression (scipy)[14] of experimentally measured values to the standard 4-parameter logistic curve model.

2.12. Thiopeptide metabolic stability experiments

Serum stability assay. To a solution of analyte thiopeptide (13.3 μM) and the internal standard peptide (13.3 μM NH_2-PEG_5-wstndwdtnd-PEG_5-$CONH_2$;[ref 15] lower case encoding is for D-amino acids; synthesized using the standard Fmoc-SPPS techniques) in TBS [50 mM Tris (pH 7.6), 150 mM NaCl] human serum (0.33 volumes; Cosmo Bio) was added on ice. The mixtures were stirred well and transferred to a 37 °C thermostat. At various timepoints, aliquots were withdrawn and quenched with 1 volume of 96% ethanol containing 30 mM IAA. The mixtures were incubated on ice for 15 min. The samples were then centrifuged (18000 g for 2 min), and 0.5 μL of the supernatant was analyzed by LC-MS (section 2.7) using method 2 as detailed below.

Method 2

0 – 1 min	1% B
1 – 9 min	1 – 81% B, linear ramp
9 – 10 min	95% B
10 – 12 min	1% B

The ratio of areas under the peak for the intact analyte over the internal standard (^{nr}EIC chromatograms generated with *m/z* ±2 cutoff values) were used to calculate the extent of analyte degradation. Thiopeptide half-life values were obtained from non-linear regression (scipy)[14] of experimentally measured values to the standard first order kinetic decomposition model.

2.13. NF-kB signaling pathway inhibition assay

THP-1 Cellular Assay. THP1-XBlue cells containing an NF-kB-inducible secreted embryonic alkaline phosphatase (SEAP) reporter gene (InvivoGen) were seeded to 96-well assay plates (10^5 cells/well) with RPMI 1640 culture medium containing 10% fetal calf serum. The cells were pre-treated with various concentration of analyte thiopeptide inhibitor in 0.1% DMSO and incubated at 37 °C for 6 h. LPS-EK (30 ng/mL, InvivoGen) was subsequently added to activate the NF-kB signalling for 20 h. To quantitate SEAP activity, supernatants were harvested (300 g, 5min) and mixed with Quanti-Blue (InvivoGen) detection reagent following the manufacturer's instructions. Cells were assayed with cell counting kit-8 in parallel to rule out potential cytotoxicity. The plates were incubated at 37 °C for 2 h and absorbance at 650 nM was measured using a Tecan M1000 PRO microplate reader (Tecan Group). NF-kB signaling inhibition activity was normalized to a no inhibitor control, and IC_{50} values were obtained from non-linear regression (scipy)[14] of experimentally measured values to the standard 4-parameter logistic curve model.

2.14. Chloroalkane penetration assay

CAPA assay. A previously constructed cell line was used for the experiment.[4] HaloTag expressing HEK293H cells were seeded on a 96-well collagen-I coated plate ($6 \cdot 10^4$ cells/well) and incubated for 24 h. The following day, the medium was gently aspirated and analyte solutions were added [peptide stocks in DMSO were diluted in Opti-MEM containing 5% FBS ensuring that the final DMSO concentration is kept to 1% (v/v)]. The plates were incubated at 37 °C for 18 h, after which the medium was aspirated, the cells were washed with Opti-MEM containing 5% FBS, and ct-TAMRA[16] (5 µM in the same medium) was added. Following a 15 min incubation at 37 °C, the cells were washed twice with the same medium, lifted with trypsin, and resuspended in PBS. The samples were analyzed by flow cytometry (CytoFLEX S, Beckman coulter), and the resulting data was then processed with FlowJo (Becton Dickinson). TAMRA fluorescence intensity was normalized to the GFP signal, and CP_{50} values were obtained from non-linear regression (scipy)[14] of experimentally measured normalized mean fluorescence values to the standard 4-parameter logistic curve model.

3. Supplementary Figures

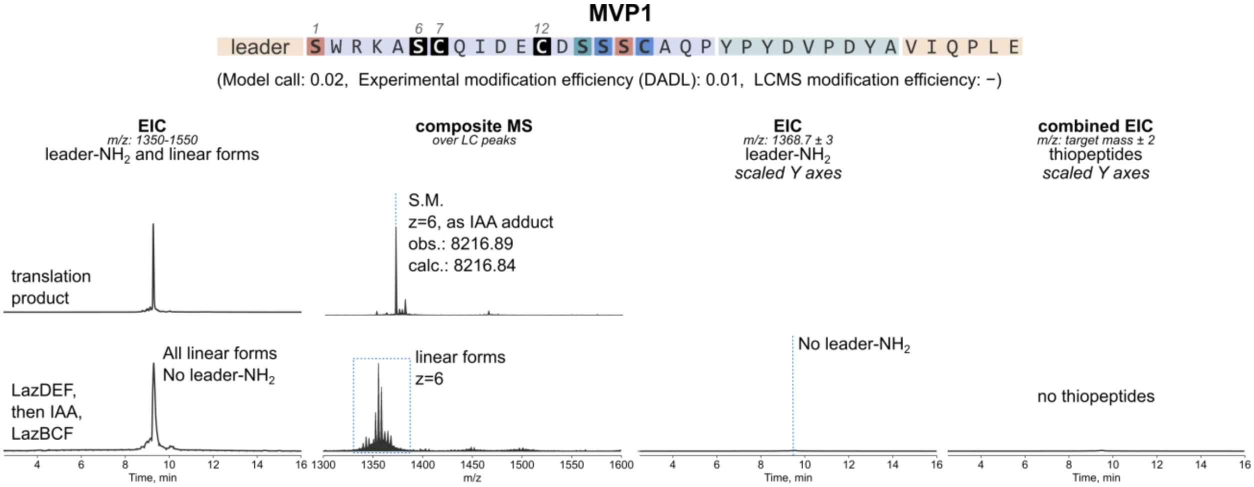

Figure S1. Analysis of maturation of MVP1 precursor peptides with LC/MS. Displayed are extracted ion current (EIC) chromatograms (*m/z*: 1350-1550; visualizes the accumulation of linear forms and leader-NH_2); composite MS spectra integrated over substrate-derived peaks (*m/z*: 1300-1600/1700, i.e., the region corresponding to the LC peaks in the chromatograms on the left); EIC chromatograms for leader-NH_2 (*m/z*: 1368.7 ± 3; scaled Y-axes to enable relative quantification of macrocyclization yields); and composite EICs for all detected thiopeptide products (scaled Y-axes). No thiopeptide was observed therefore the LCMS modification efficiency in this case is “-".

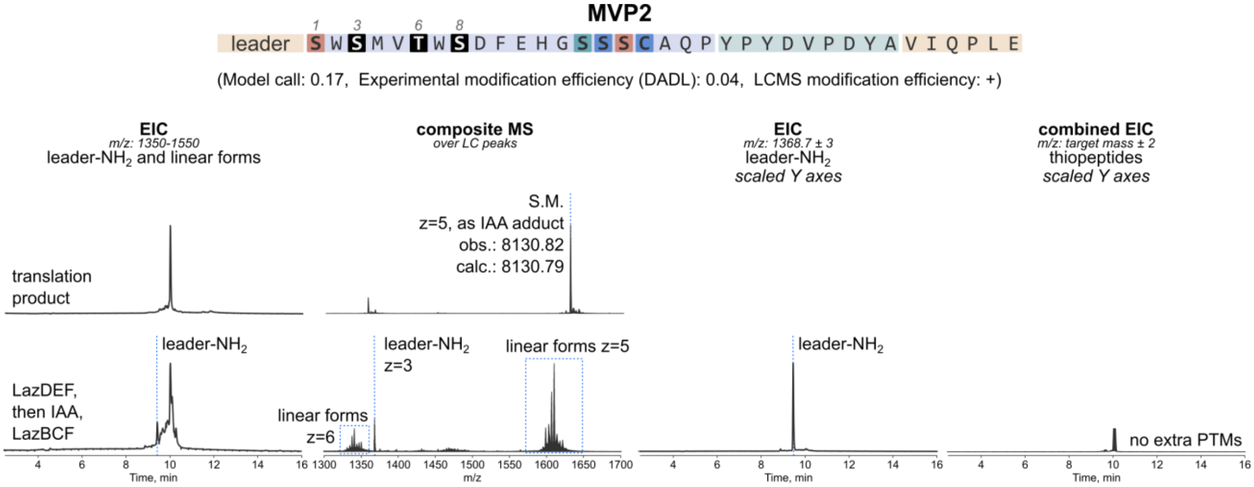

Figure S2. Analysis of maturation of MVP2 precursor peptides with LC/MS. Displayed are extracted ion current (EIC) chromatograms (*m/z*: 1350-1550; visualizes the accumulation of linear forms and leader-NH_2); composite MS spectra integrated over substrate-derived peaks (*m/z*: 1300-1600/1700, i.e., the region corresponding to the LC peaks in the chromatograms on the left); EIC chromatograms for leader-NH_2 (*m/z*: 1368.7 ± 3; scaled Y-axes to enable relative quantification of macrocyclization yields); and composite EICs for all detected thiopeptide products (scaled Y-axes). Although a tiny amount of thiopeptide was observed, the majority of the products are linear forms, therefore the LCMS modification efficiency in this case is “+".

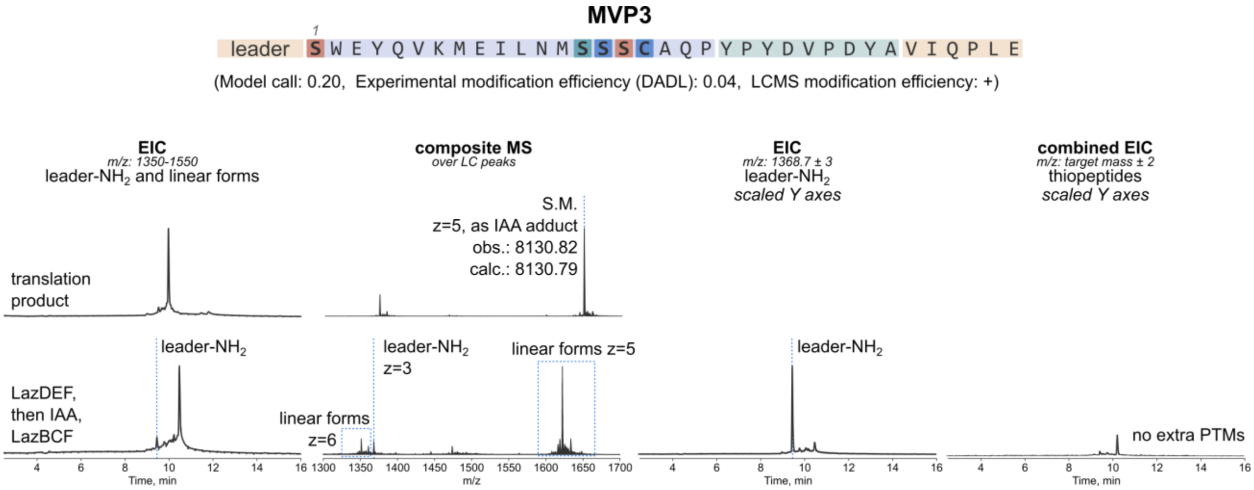

Figure S3. Analysis of maturation of MVP3 precursor peptides with LC/MS. Displayed are extracted ion current (EIC) chromatograms (*m/z*: 1350-1550; visualizes the accumulation of linear forms and leader-NH_2); composite MS spectra integrated over substrate-derived peaks (*m/z*: 1300-1600/1700, i.e., the region corresponding to the LC peaks in the chromatograms on the left); EIC chromatograms for leader-NH_2 (*m/z*: 1368.7 ± 3; scaled Y-axes to enable relative quantification of macrocyclization yields); and composite EICs for all detected thiopeptide products (scaled Y-axes). Although a tiny amount of thiopeptide was observed, the majority of the products are linear forms, therefore the LCMS modification efficiency in this case is "+".

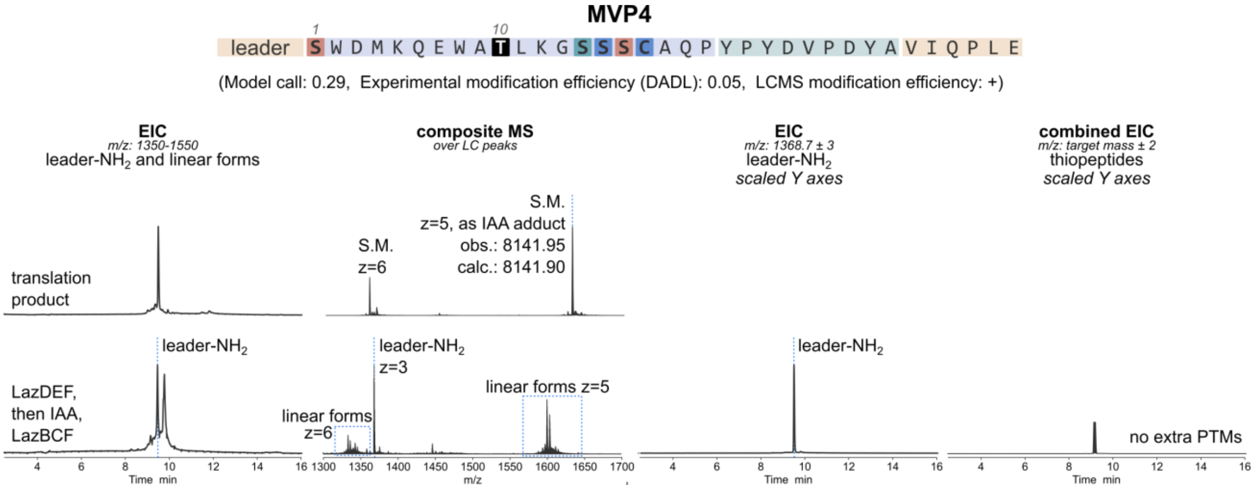

Figure S4. Analysis of maturation of MVP4 precursor peptides with LC/MS. Displayed are extracted ion current (EIC) chromatograms (*m/z*: 1350-1550; visualizes the accumulation of linear forms and leader-NH_2); composite MS spectra integrated over substrate-derived peaks (*m/z*: 1300-1600/1700, i.e., the region corresponding to the LC peaks in the chromatograms on the left); EIC chromatograms for leader-NH_2 (*m/z*: 1368.7 ± 3; scaled Y-axes to enable relative quantification of macrocyclization yields); and composite EICs for all detected thiopeptide products (scaled Y-axes). Although thiopeptide was observed, the majority of the products are linear forms, therefore the LCMS modification efficiency in this case is "+".

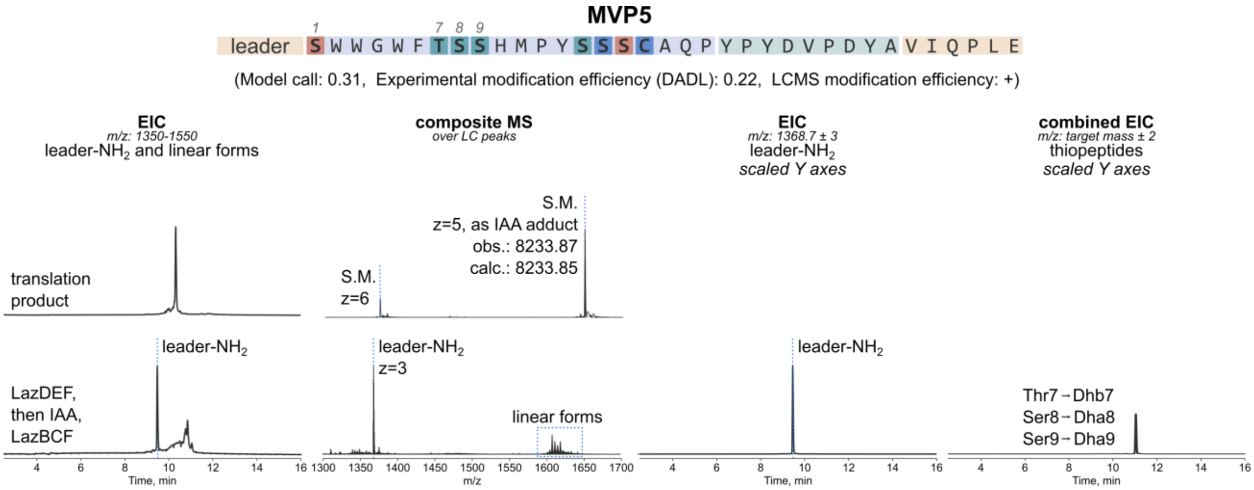

Figure S5. Analysis of maturation of MVP5 precursor peptides with LC/MS. Displayed are extracted ion current (EIC) chromatograms (*m/z*: 1350-1550; visualizes the accumulation of linear forms and leader-NH_2); composite MS spectra integrated over substrate-derived peaks (*m/z*: 1300-1600/1700, i.e., the region corresponding to the LC peaks in the chromatograms on the left); EIC chromatograms for leader-NH_2 (*m/z*: 1368.7 ± 3; scaled Y-axes to enable relative quantification of macrocyclization yields); and composite EICs for all detected thiopeptide products (scaled Y-axes). Although thiopeptide was observed, the linear forms were still very significant, therefore the LCMS modification efficiency in this case is "+".

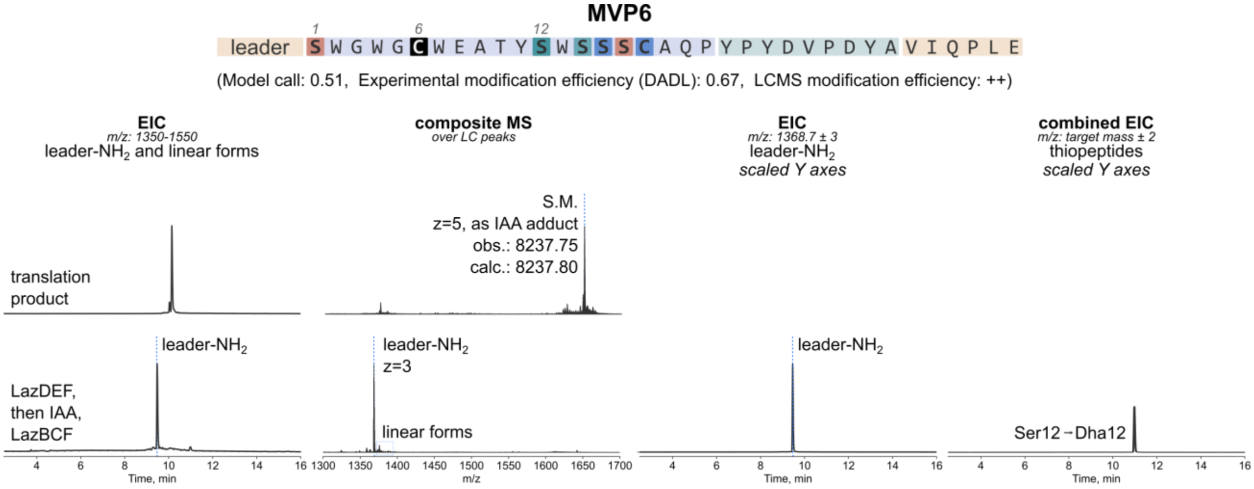

Figure S6. Analysis of maturation of MVP6 precursor peptides with LC/MS. Displayed are extracted ion current (EIC) chromatograms (*m/z*: 1350-1550; visualizes the accumulation of linear forms and leader-NH_2); composite MS spectra integrated over substrate-derived peaks (*m/z*: 1300-1600/1700, i.e., the region corresponding to the LC peaks in the chromatograms on the left); EIC chromatograms for leader-NH_2 (*m/z*: 1368.7 ± 3; scaled Y-axes to enable relative quantification of macrocyclization yields); and composite EICs for all detected thiopeptide products (scaled Y-axes). Clean reaction with very minor linear forms, therefore the LCMS modification efficiency in this case is "++".

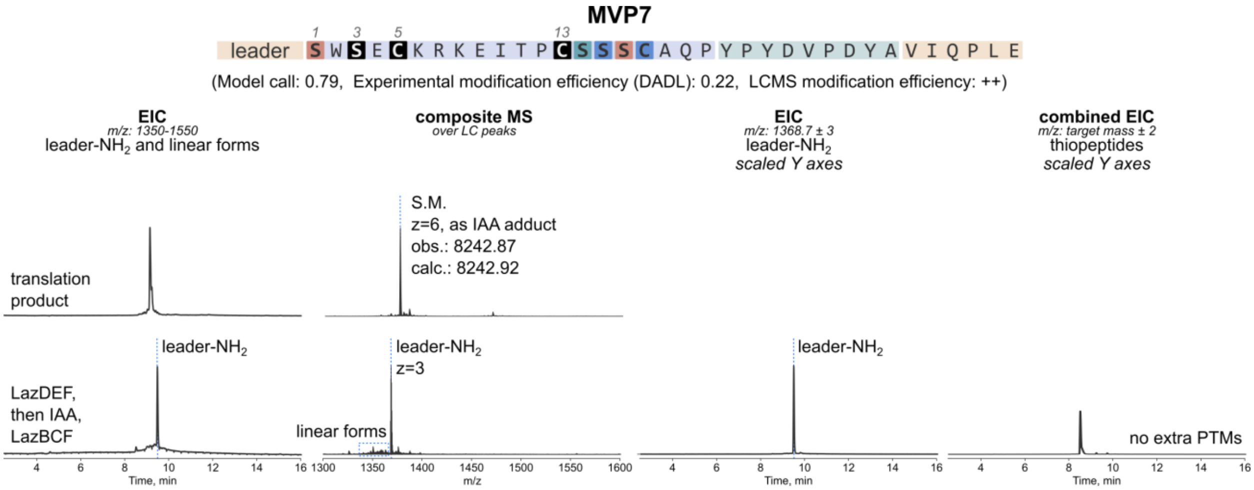

Figure S7. Analysis of maturation of MVP7 precursor peptides with LC/MS. Displayed are extracted ion current (EIC) chromatograms (*m/z*: 1350-1550; visualizes the accumulation of linear forms and leader-NH_2); composite MS spectra integrated over substrate-derived peaks (*m/z*: 1300-1600/1700, i.e., the region corresponding to the LC peaks in the chromatograms on the left); EIC chromatograms for leader-NH_2 (*m/z*: 1368.7 ± 3; scaled Y-axes to enable relative quantification of macrocyclization yields); and composite EICs for all detected thiopeptide products (scaled Y-axes). Clean reaction with very minor linear forms, therefore the LCMS modification efficiency in this case is "++".

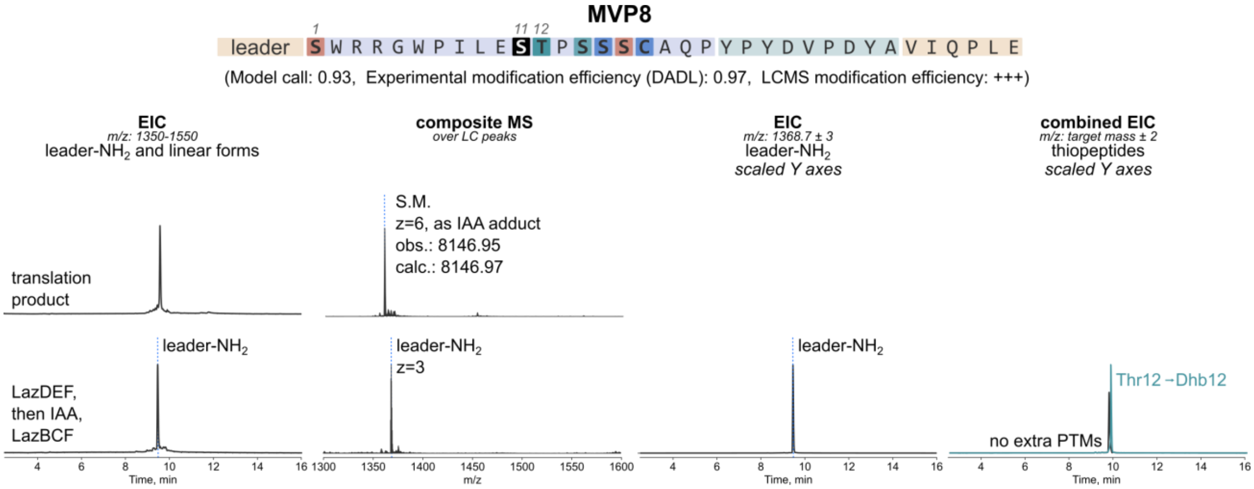

Figure S8. Analysis of maturation of MVP8 precursor peptides with LC/MS. Displayed are extracted ion current (EIC) chromatograms (*m/z*: 1350-1550; visualizes the accumulation of linear forms and leader-NH_2); composite MS spectra integrated over substrate-derived peaks (*m/z*: 1300-1600/1700, i.e., the region corresponding to the LC peaks in the chromatograms on the left); EIC chromatograms for leader-NH_2 (*m/z*: 1368.7 ± 3; scaled Y-axes to enable relative quantification of macrocyclization yields); and composite EICs for all detected thiopeptide products (scaled Y-axes). Very clean reaction, therefore the LCMS modification efficiency in this case is "+++".

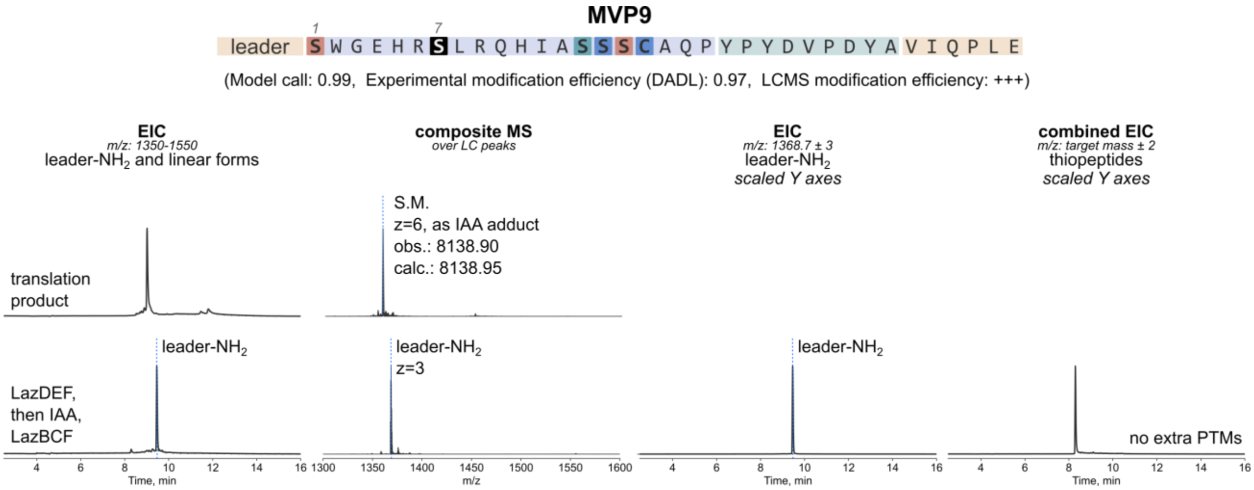

Figure S9. Analysis of maturation of MVP9 precursor peptides with LC/MS. Displayed are extracted ion current (EIC) chromatograms (*m/z*: 1350-1550; visualizes the accumulation of linear forms and leader-NH_2); composite MS spectra integrated over substrate-derived peaks (*m/z*: 1300-1600/1700, i.e., the region corresponding to the LC peaks in the chromatograms on the left); EIC chromatograms for leader-NH_2 (*m/z*: 1368.7 ± 3; scaled Y-axes to enable relative quantification of macrocyclization yields); and composite EICs for all detected thiopeptide products (scaled Y-axes). Very clean reaction, therefore the LCMS modification efficiency in this case is “+++”.

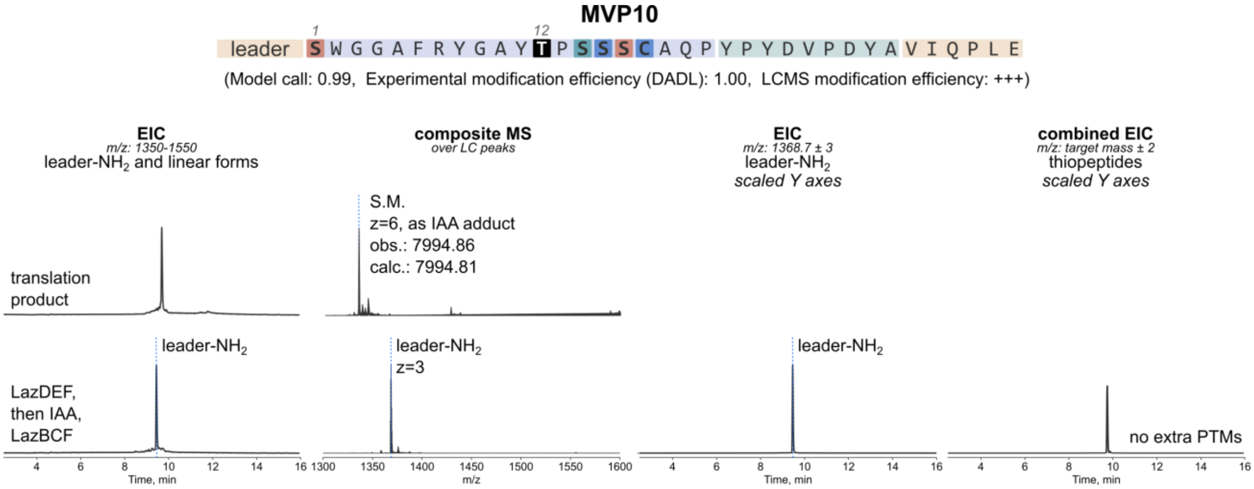

Figure S10. Analysis of maturation of MVP10 precursor peptides with LC/MS. Displayed are extracted ion current (EIC) chromatograms (*m/z*: 1350-1550; visualizes the accumulation of linear forms and leader-NH_2); composite MS spectra integrated over substrate-derived peaks (*m/z*: 1300-1600/1700, i.e., the region corresponding to the LC peaks in the chromatograms on the left); EIC chromatograms for leader-NH_2 (*m/z*: 1368.7 ± 3; scaled Y-axes to enable relative quantification of macrocyclization yields); and composite EICs for all detected thiopeptide products (scaled Y-axes). Very clean reaction, therefore the LCMS modification efficiency in this case is “+++”.

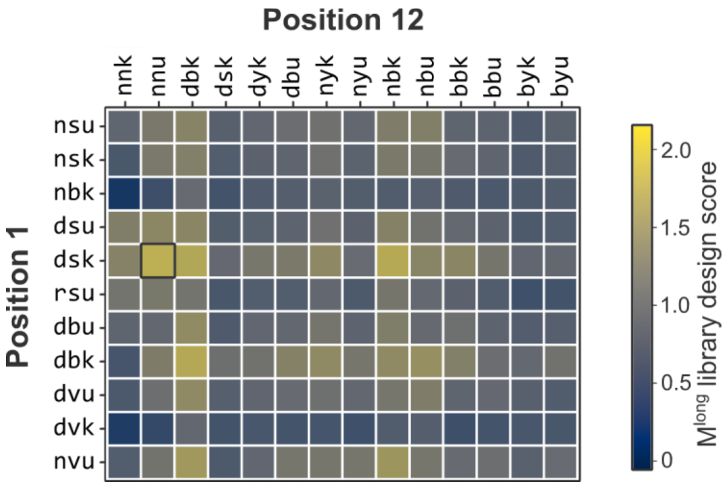

Figure S11. Degenerate codon combinations at position 1 and position 12. dsk-$(nnk)_{10}$-nnu had the highest M^{long} scores (Table S5).

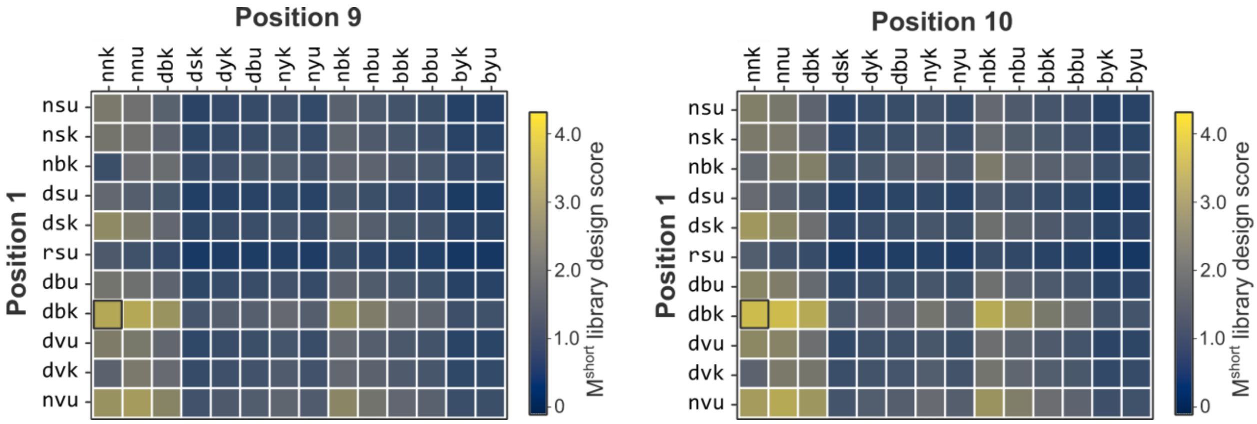

Figure S12. Degenerate codon combinations at position 1 and position9/10. For these shorter libraries, dbk-$(nnk)_n$ (n=8-9) had the highest M^{short} scores (Table S5).

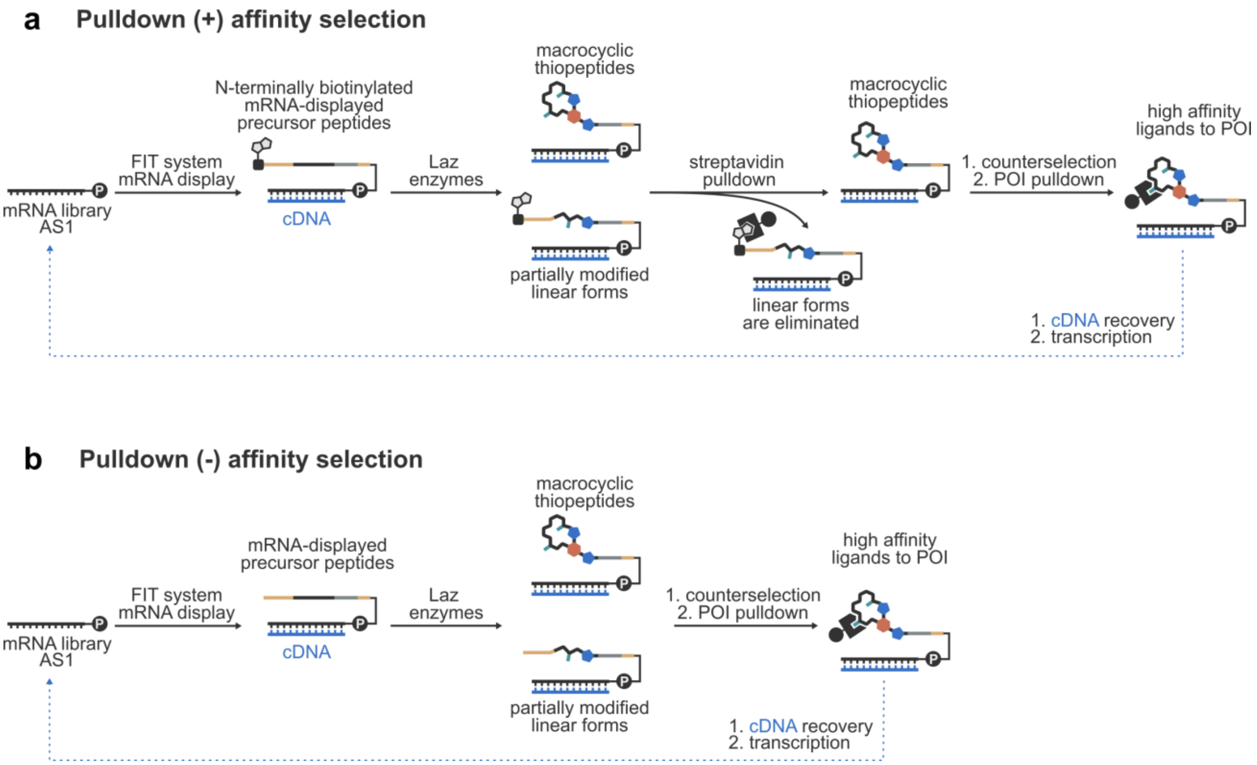

Figure S13. The schemes of thiopeptide affinity selections. mRNA library AS1, which features an expanded and randomized 6-12 insert in the core peptide region and a C-terminal linker, are first displayed on cognate mRNA and then converted to thiopeptides with Laz enzymes. In pulldown (+) selection, partially modified and shunt products maintain the N-terminal biotin tag during biosynthesis and are eliminated by a streptavidin pulldown. The resulting mRNA-displayed library of thiopeptides is panned against immobilized protein of interest (POI) to enrich for high affinity thiopeptide ligands. In pulldown (-) selection, both partially modified linear forms and macrocyclic thiopeptide are subjected to affinity selection against the POI. After the affinity selection, the process can be repeated by recovering DNA and converting it to mRNA for the following round of selection.

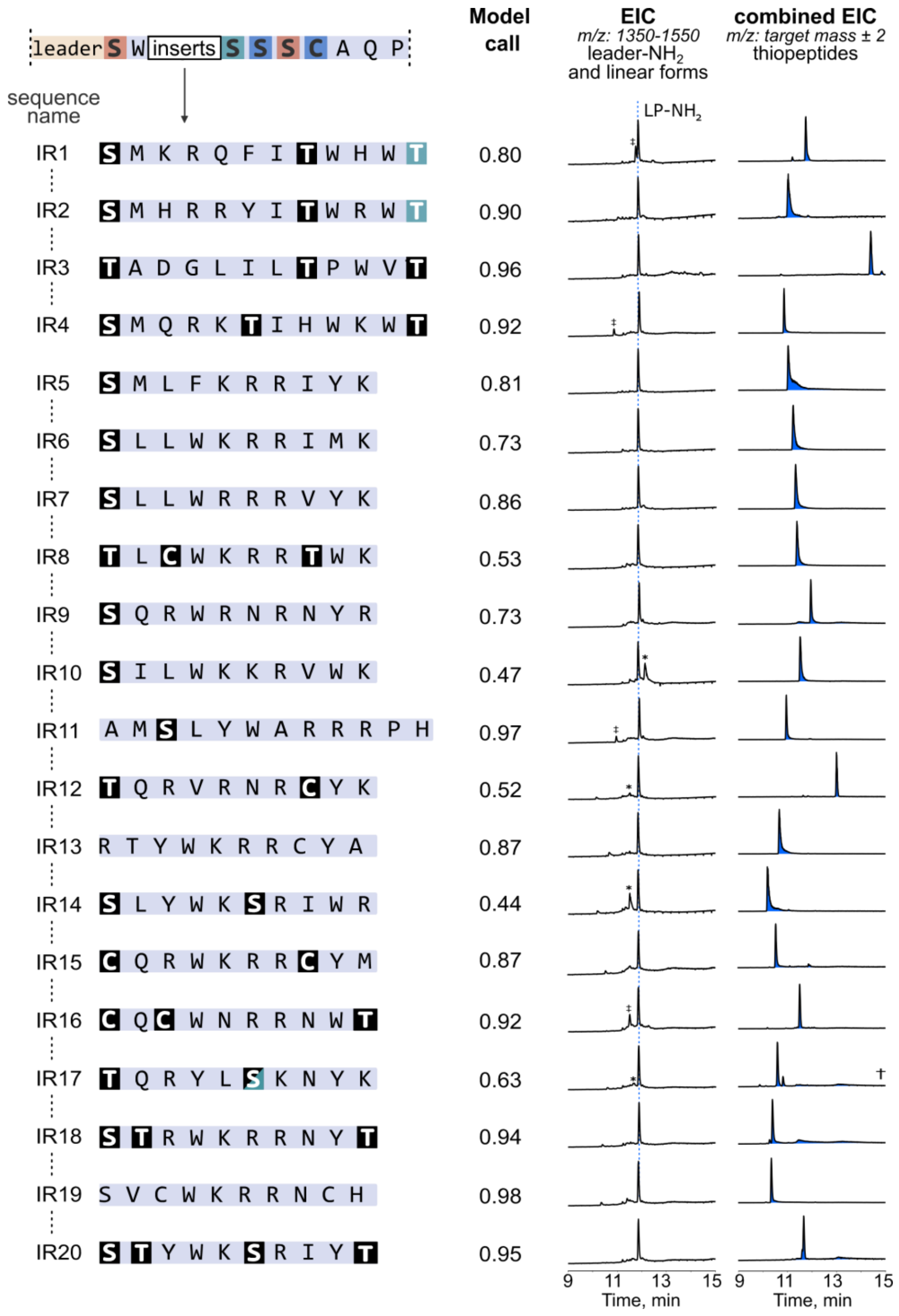

Figure S14. Maturation of 20 selected thiopeptide precursors without/without streptavidin pulldown against IRAK4 by Laz enzymes. In vitro expressed IR1-IR20 clones were treated with LazDEF/BCF under the selection conditions, and the outcomes were analyzed by LC/MS. Displayed are the model prediction, EIC chromatograms showing the formation of LP-NH_2 (one of the macrocyclization products) and linear forms, and the combined EIC chromatograms for the identified thiopeptides. Ser/Thr/Cys, i.e., the residues which can potentially be modified by LazDEF and LazBF, are highlighted in random insert sequences: unmodified Ser/Thr/Cys are in black, dehydroamino acids derived from Ser and Thr by LazBF – in green. Simultaneous green and black highlighting indicates the sites of partial dehydration of Ser/Thr residues which leads to the formation of product mixtures. *: peaks corresponding to linear forms. ‡: Thiopeptides. †: see Fig. S16 for the annotation of individual thiopeptides in product mixtures.

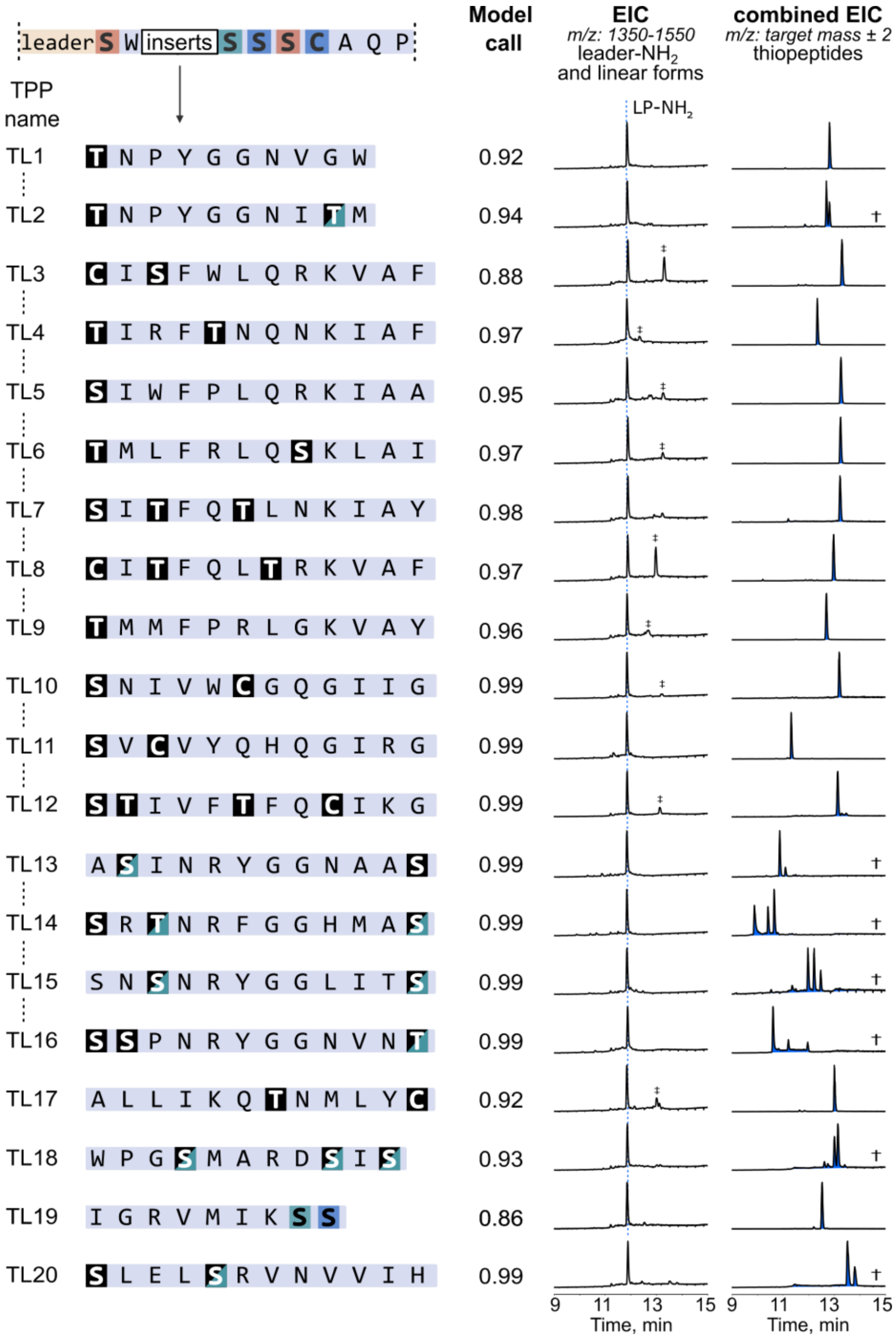

Figure S15. Maturation of 20 selected thiopeptide precursors without/without streptavidin pulldown against TLR10 by Laz enzymes. In vitro expressed TL1-TL20 clones were treated with LazDEF/BCF under the selection conditions, and the outcomes were analyzed by LC/MS. Displayed are the model prediction, EIC chromatograms showing the formation of LP-NH2 (one of the macrocyclization products) and linear forms, and the combined EIC chromatograms for the identified thiopeptides. Ser/Thr/Cys, i.e., the residues which can potentially be modified by LazDEF and LazBF, are highlighted in random insert sequences: unmodified Ser/Thr/Cys are in black, dehydroamino acids derived from Ser and Thr by LazBF – in green; Ser/Thr/Cys cyclodehydrated to azolines and azoles – in blue. Simultaneous green and black highlighting indicates the sites of partial dehydration of Ser/Thr residues which leads to the formation of product mixtures. *: peaks corresponding to linear TPP forms. ‡: Thiopeptides. †: see Fig. S17-S23 for the annotation of individual thiopeptides in product mixtures.

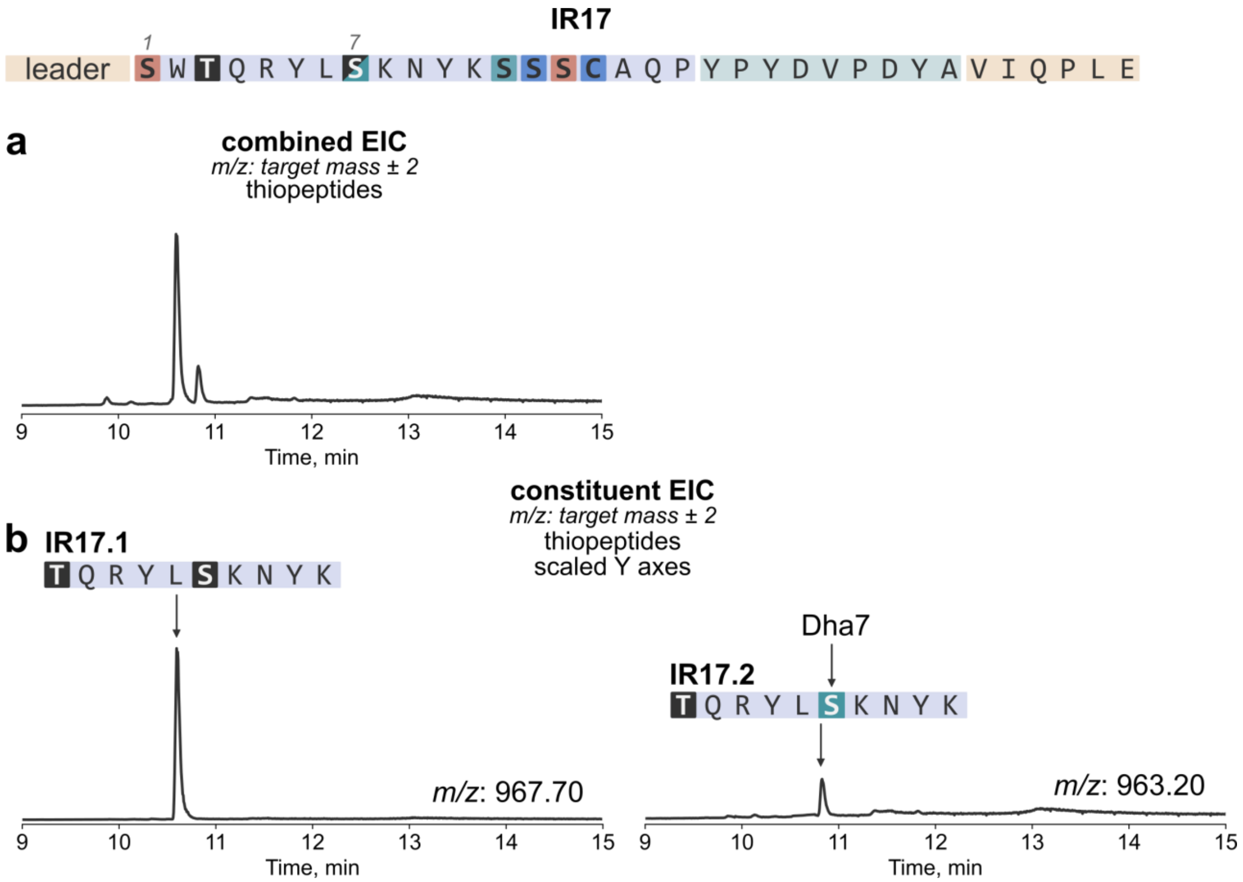

Figure S16. Formation of thiopeptide mixtures from IR17. FIT-system derived peptide was treated with Laz enzymes as described in section 2.8 and the outcomes were analyzed by LC/MS. a) A composite EIC for all detected TP products (as in Fig. S14). b) EIC for the individual products and their annotations.

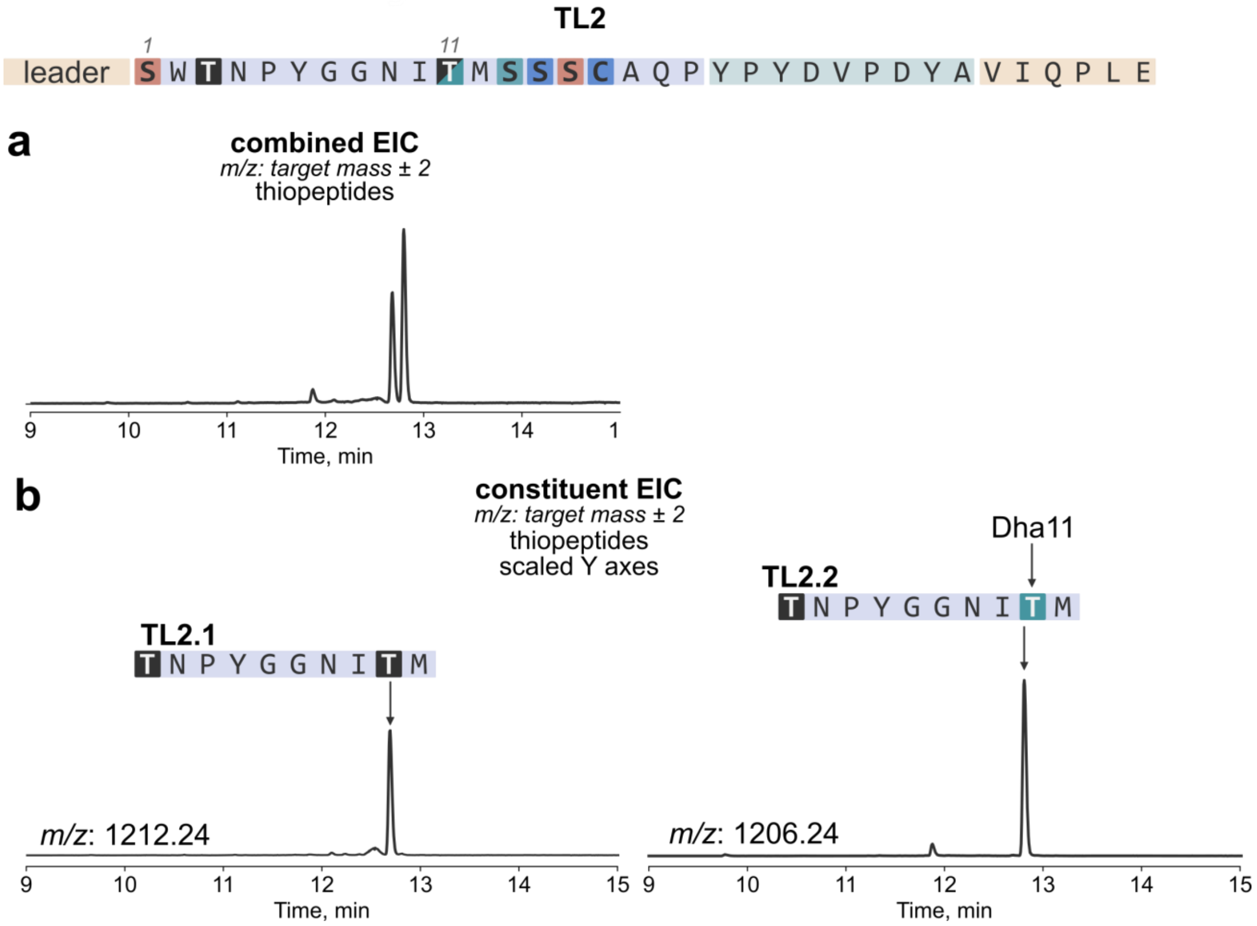

Figure S17. Formation of thiopeptide mixtures from TL2. FIT-system derived peptide was treated with Laz enzymes as described in section 2.8 and the outcomes were analyzed by LC/MS. a) A composite EIC for all detected TP products (as in Fig. S15). b) EIC for the individual products and their annotations.

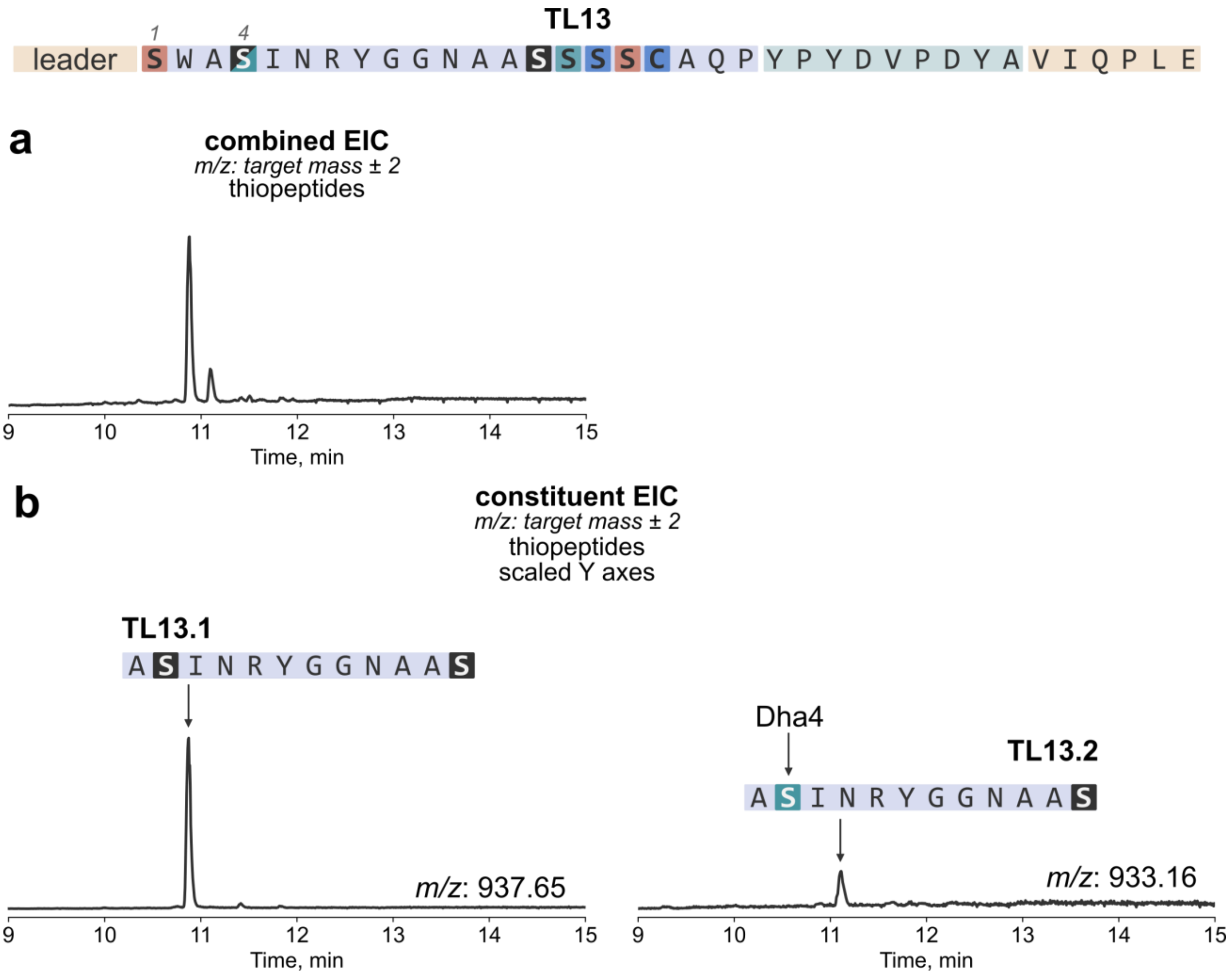

Figure S18. Formation of thiopeptide mixtures from TL13. FIT-system derived peptide was treated with Laz enzymes as described in section 2.8 and the outcomes were analyzed by LC/MS. a) A composite EIC for all detected TP products (as in Fig. S15). b) EIC for the individual products and their annotations.

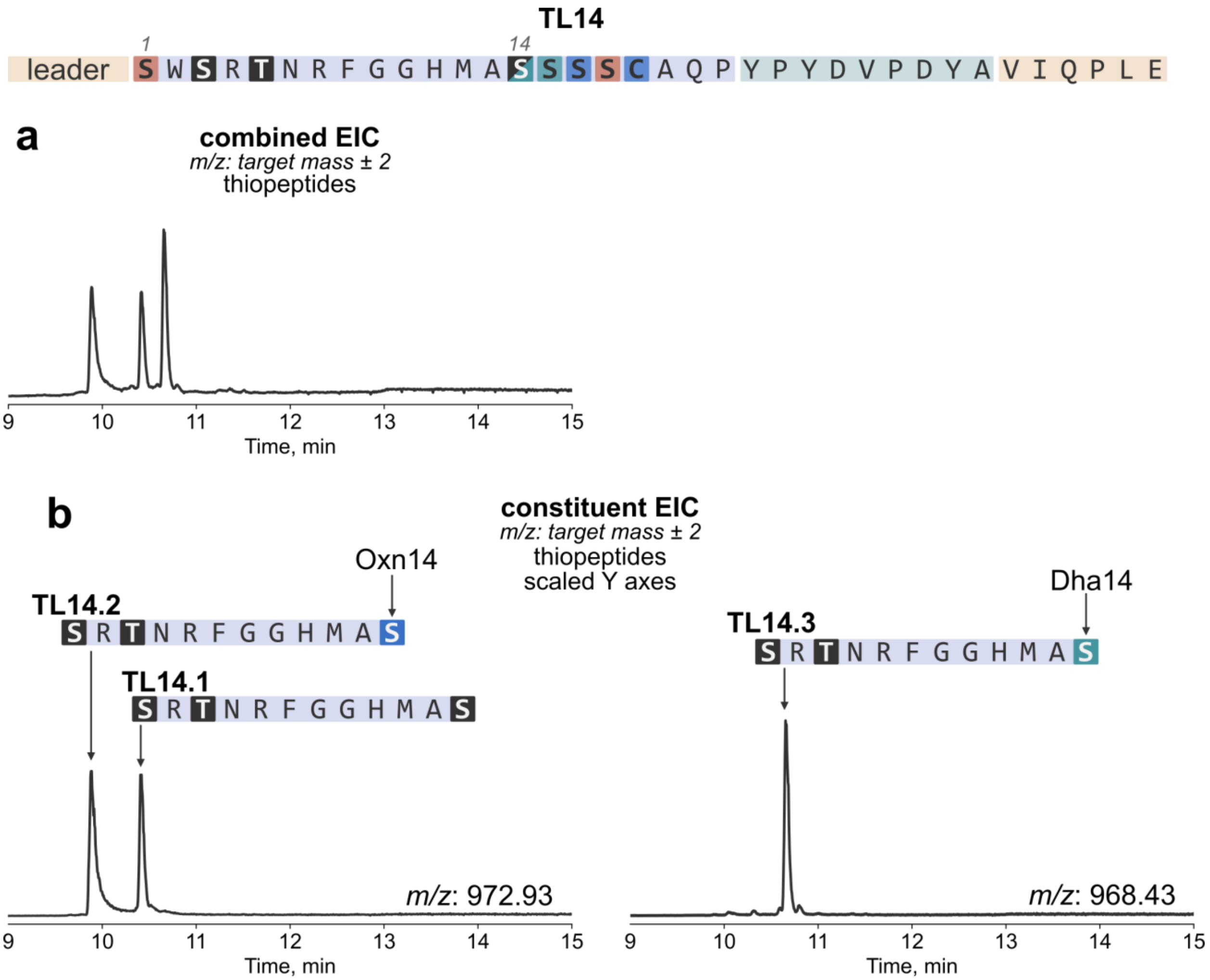

Figure S19. Formation of thiopeptide mixtures from TL14. FIT-system derived peptide was treated with Laz enzymes as described in section 2.8 and the outcomes were analyzed by LC/MS. a) A composite EIC for all detected TP products (as in Fig. S15). b) EIC for the individual products and their annotations.

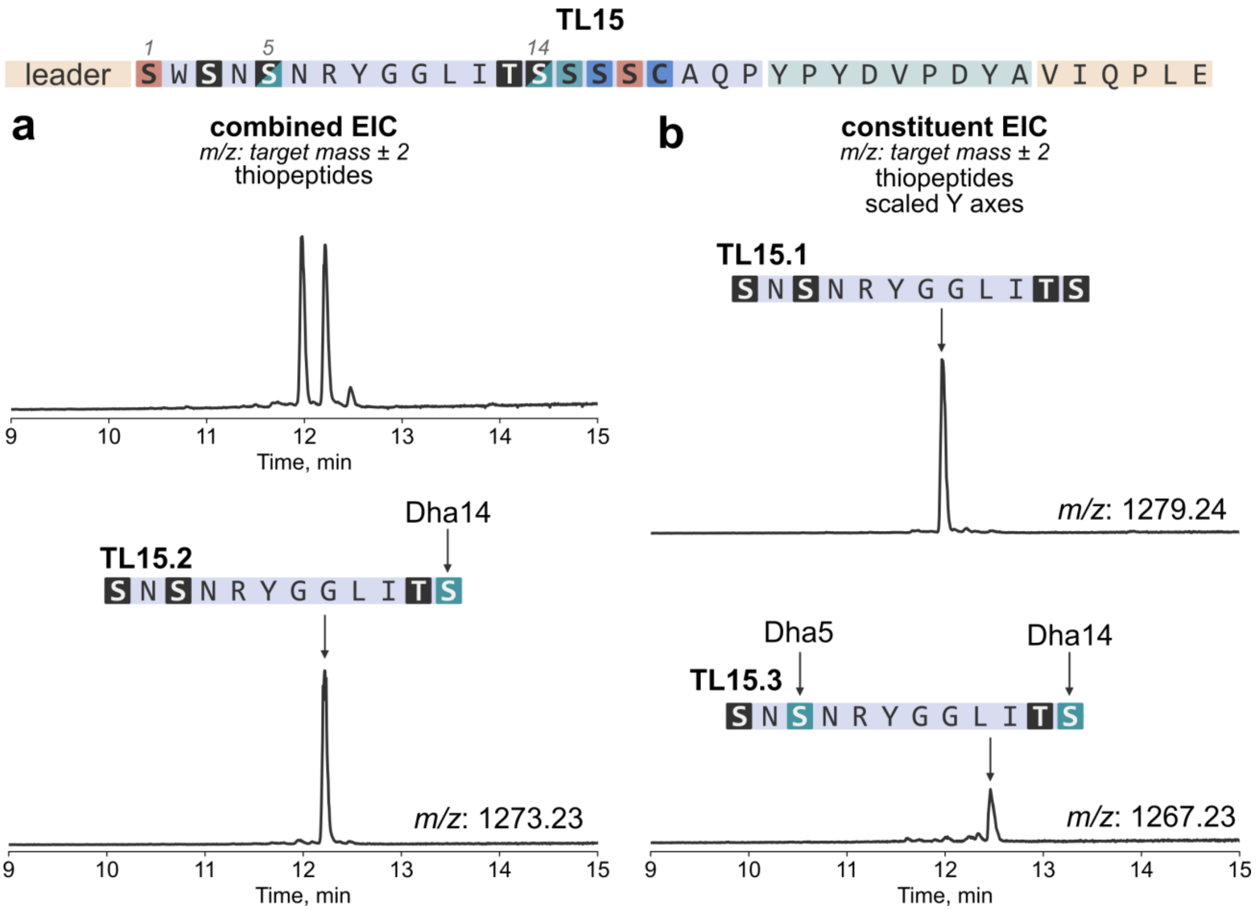

Figure S20. Formation of thiopeptide mixtures from TL15. FIT-system derived peptide was treated with Laz enzymes as described in section 2.8 and the outcomes were analyzed by LC/MS. a) A composite EIC for all detected TP products (as in Fig. S15). b) EIC for the individual products and their annotations.

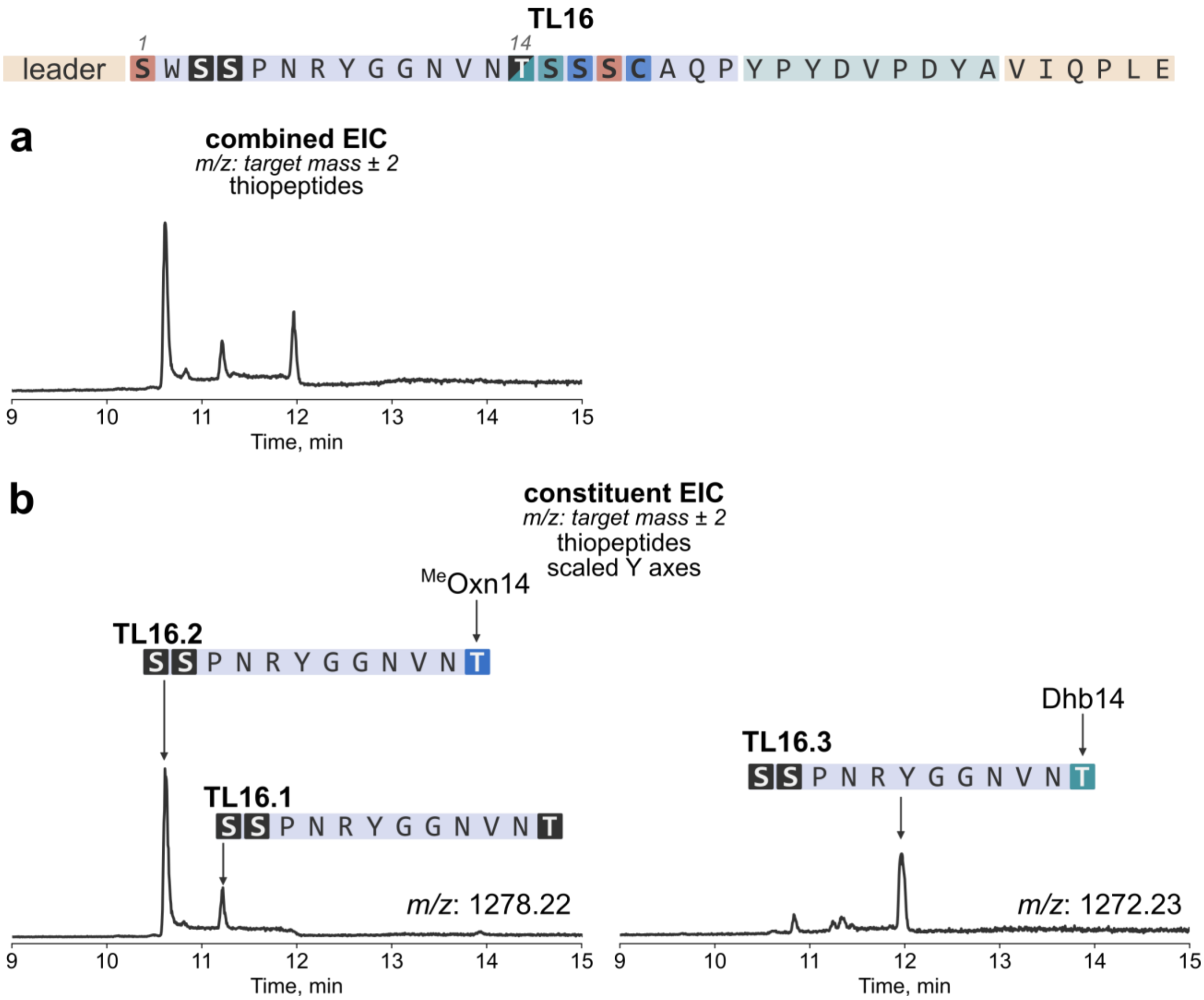

Figure S21. Formation of thiopeptide mixtures from TL16. FIT-system derived peptide was treated with Laz enzymes as described in section 2.8 and the outcomes were analyzed by LC/MS. a) A composite EIC for all detected TP products (as in Fig. S15). b) EIC for the individual products and their annotations.

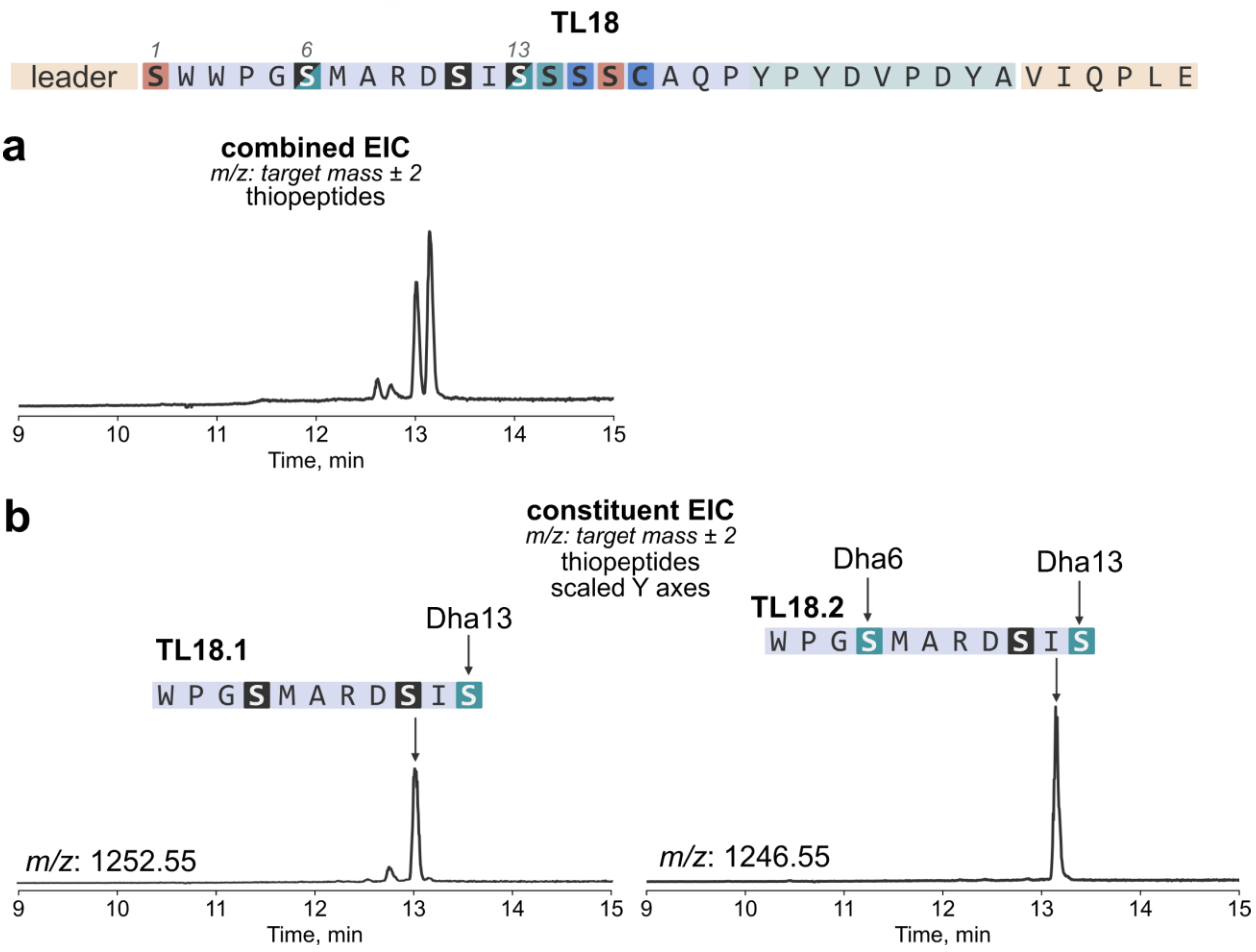

Figure S22. Formation of thiopeptide mixtures from TL18. FIT-system derived peptide was treated with Laz enzymes as described in section 2.8 and the outcomes were analyzed by LC/MS. a) A composite EIC for all detected TP products (as in Fig. S15). b) EIC for the individual products and their annotations.

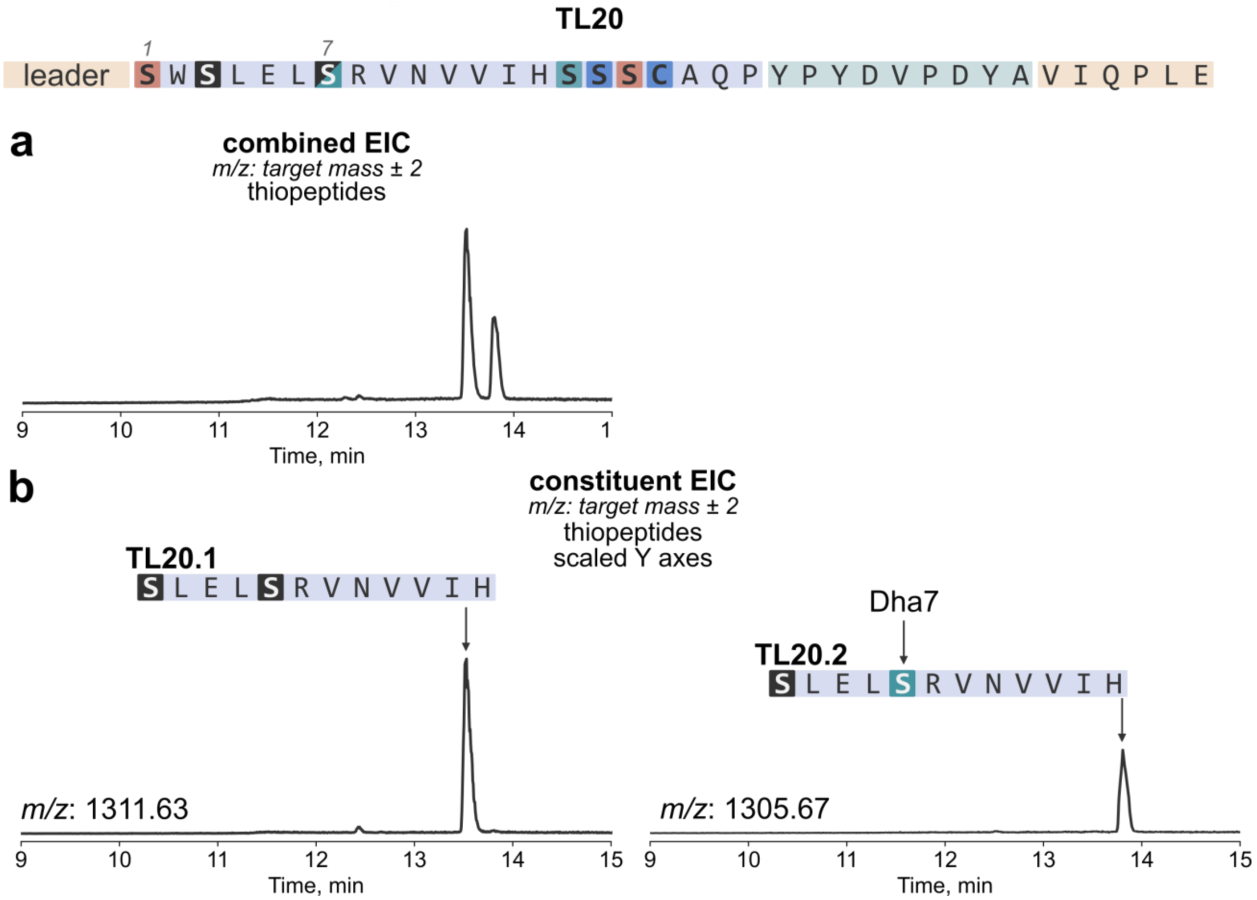

Figure S23. Formation of thiopeptide mixtures from TL20. FIT-system derived peptide was treated with Laz enzymes as described in section 2.8 and the outcomes were analyzed by LC/MS. a) A composite EIC for all detected TP products (as in Fig. S15). b) EIC for the individual products and their annotations.

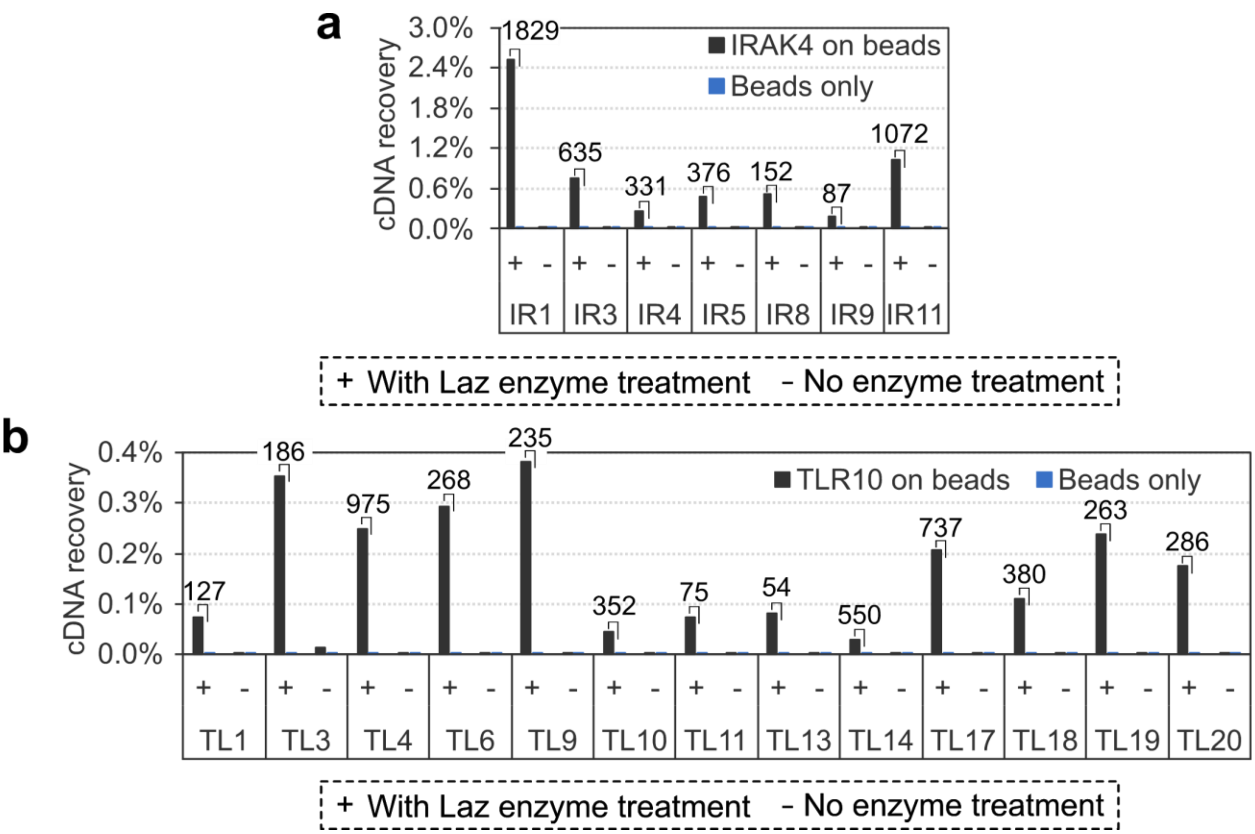

Figure S24. Qualitative analysis of the thiopeptide precursors binding to its target protein of interest using an mRNA display assay. A single round of selection (section 2.8) was carried out using the corresponding mRNA-displayed precursor peptides, and the amounts of pulled down cDNA were quantified by qPCR. To test whether linear random inserts are responsible for binding to target protein of interest, an analogous assay was conducted while omitting the enzymes (buffer only treatment). Every tested peptide preferentially bound to the target-immobilized beads after the incubation with the enzymes, which suggests that macrocyclic thiopeptides, and not their linear epitopes, are responsible for binding.

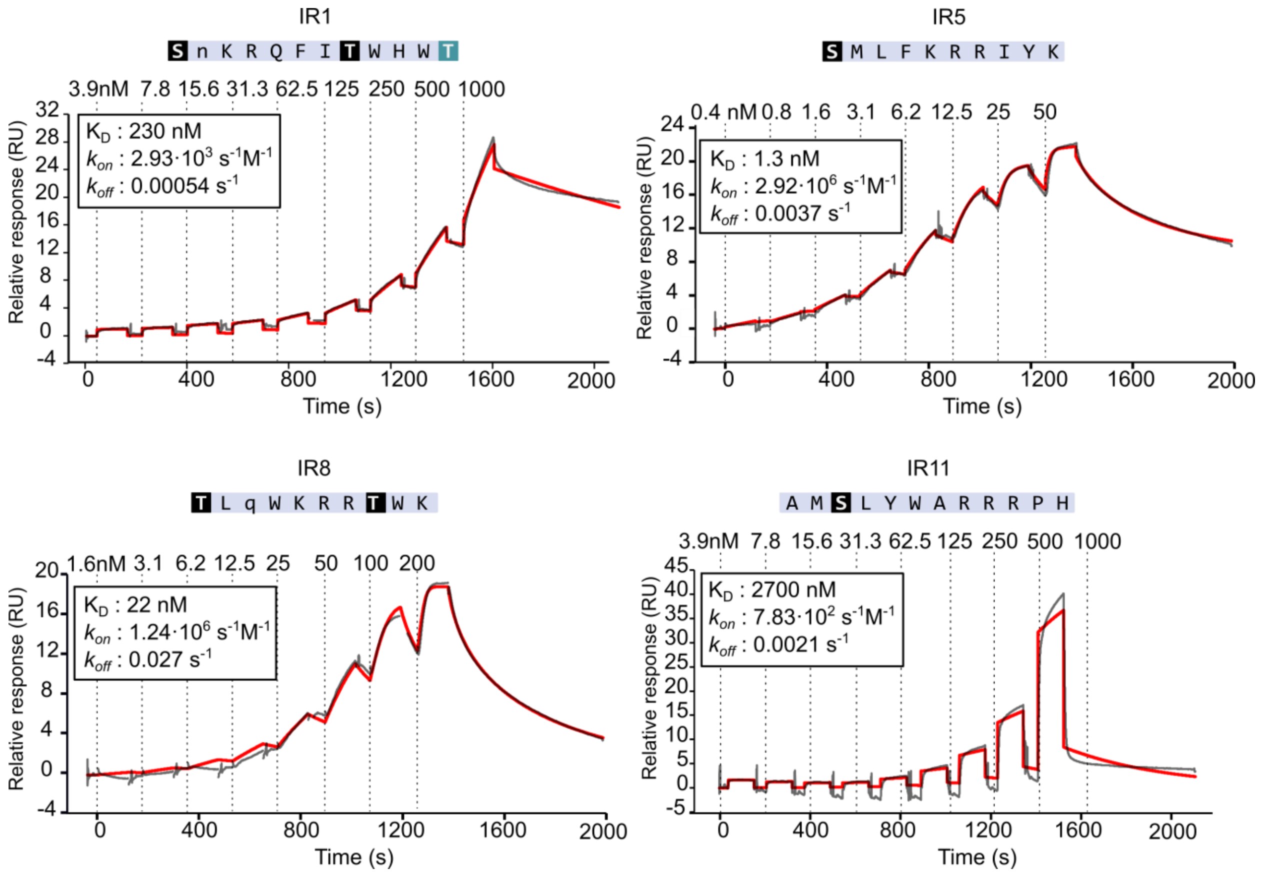

Figure S25. SPR sensorgram curves for all the synthesized compounds against IRAK4. See section 2.10 for experimental details. Experimental data are shown as black lines, and best fit in red. T highlighted in green indicates a Dhb residue; n represents ^{L}norleucine (Nle); q represents ^{L}homoglutamine (^{h}Gln).

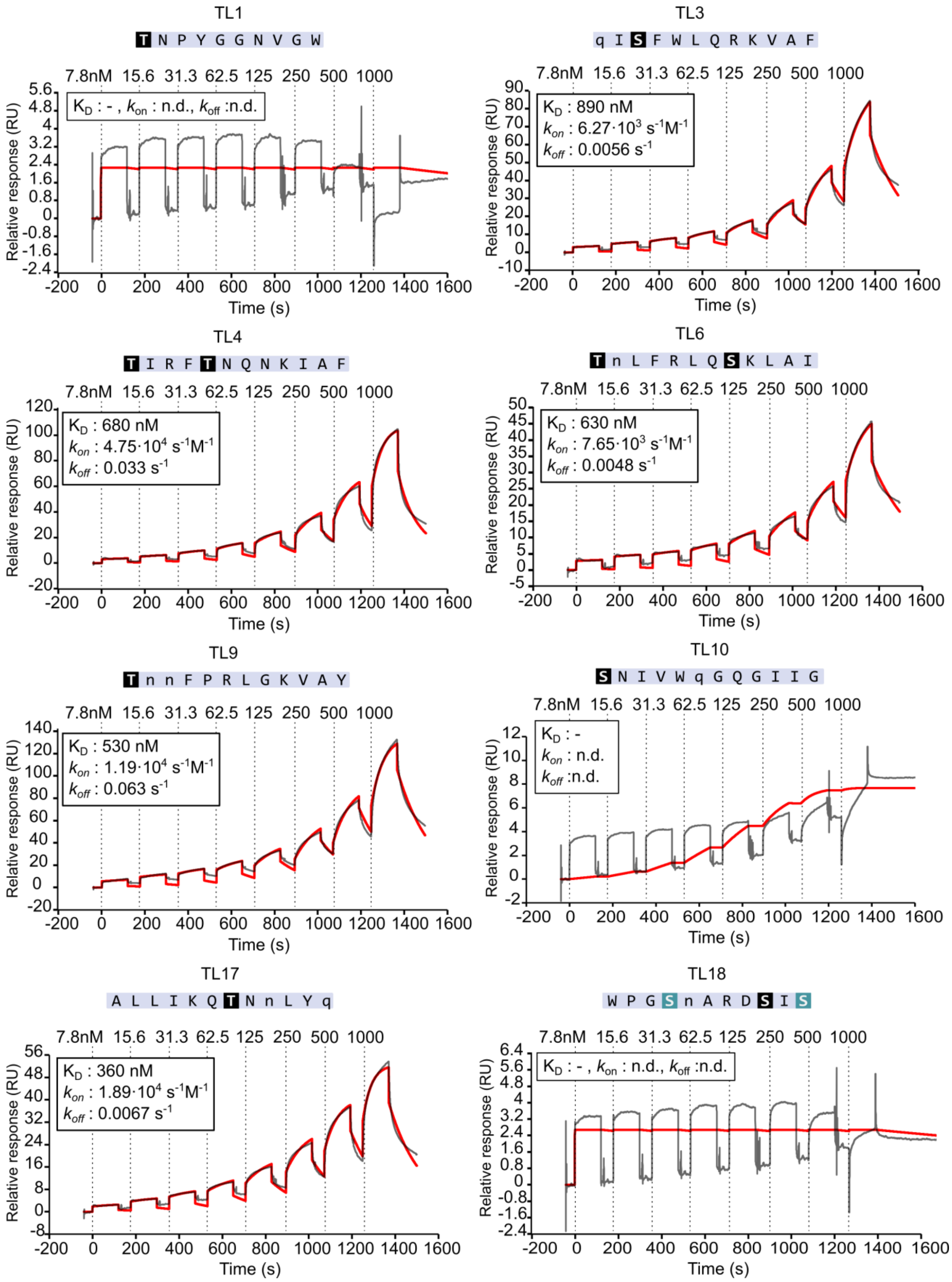
TL1
T N P Y G G N V G W
7.8nM 15.6 31.3 62.5 125 250 500 1000
K_D : - , k_on : n.d., k_off :n.d.
Relative response (RU)
Time (s)
TL3
q I S F W L Q R K V A F
7.8nM 15.6 31.3 62.5 125 250 500 1000
K_D : 890 nM
k_on : 6.27·10^3 s^-1M^-1
k_off : 0.0056 s^-1
TL4
T I R F T N Q N K I A F
K_D : 680 nM
k_on : 4.75·10^4 s^-1M^-1
k_off : 0.033 s^-1
TL6
T n L F R L Q S K L A I
K_D : 630 nM
k_on : 7.65·10^3 s^-1M^-1
k_off : 0.0048 s^-1
TL9
T n n F P R L G K V A Y
K_D : 530 nM
k_on : 1.19·10^4 s^-1M^-1
k_off : 0.063 s^-1
TL10
S N I V W q G Q G I I G
K_D : -
k_on : n.d.
k_off :n.d.
TL17
A L L I K Q T N n L Y q
K_D : 360 nM
k_on : 1.89·10^4 s^-1M^-1
k_off : 0.0067 s^-1
TL18
W P G S n A R D S I S
K_D : - , k_on : n.d., k_off :n.d.

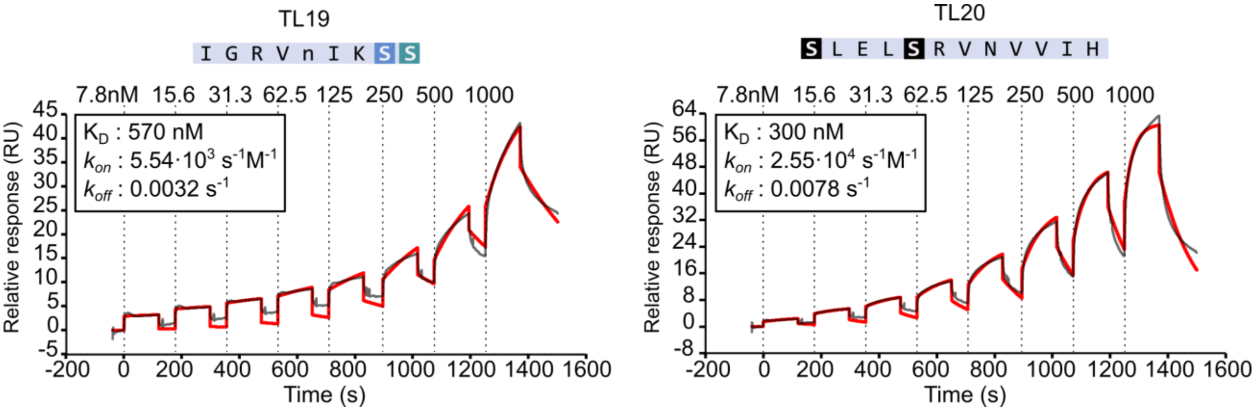

Figure S26. SPR sensorgram curves for all the synthesized compounds against TLR10. See section 2.10 for experimental details. Experimental data are shown as black lines, and best fit in red. An S highlighted in green indicates a Dha residue; S in blue – Oxz; n represents ^{L}norleucine (Nle); q represents ^{L}homoglutamine (^{h}Gln).

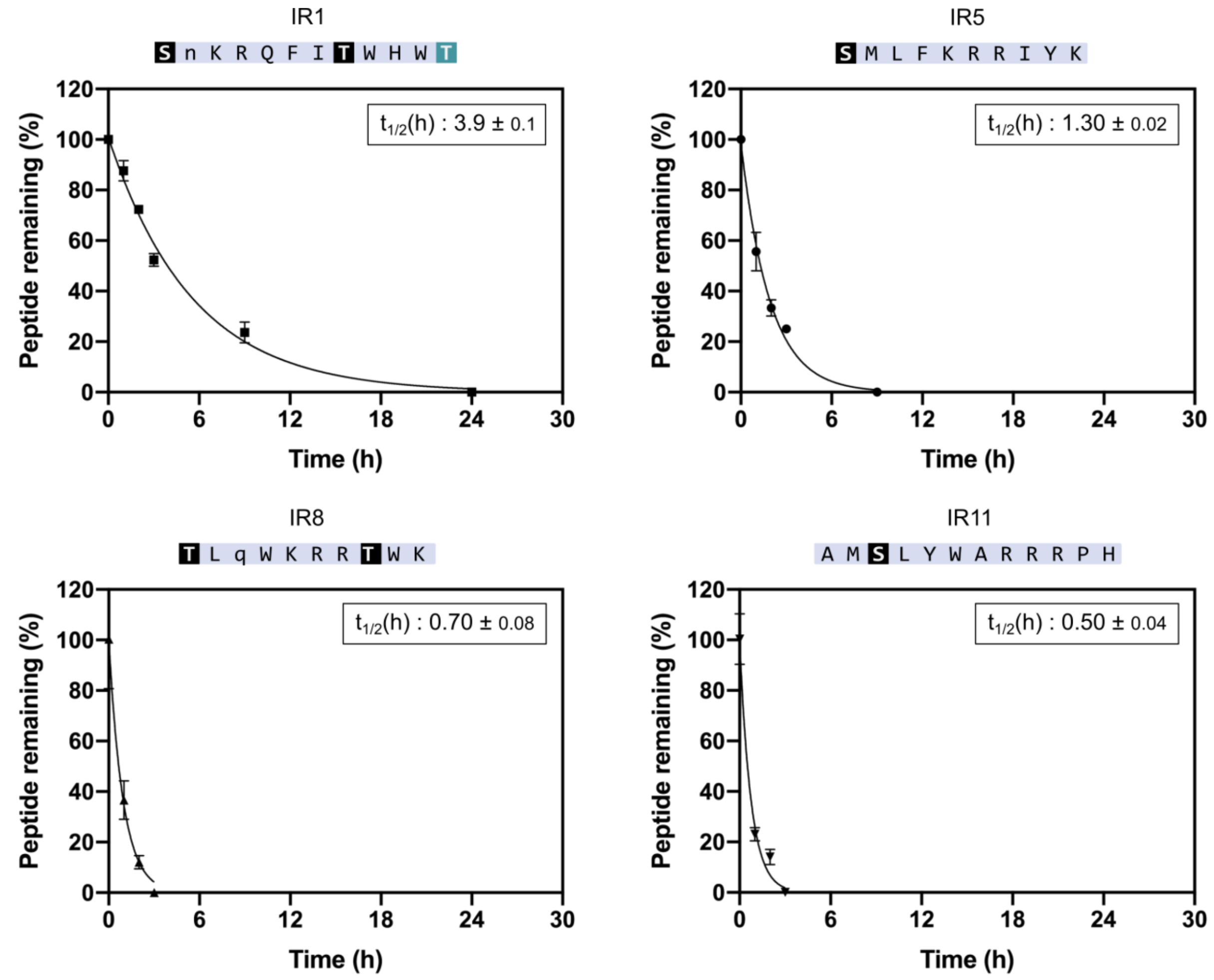

Figure S27. Serum stability of IR1, IR5, IR8, IR11. See section 2.12 for experimental details. Displayed are experimental data (points) and best fit (lines). T highlighted in green indicates a Dhb residue; n represents ^{L}norleucine (Nle); q represents ^{L}homoglutamine (^{h}Gln).

TL1

T N P Y G G N V G W

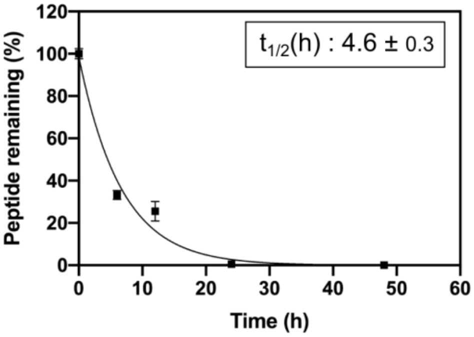

TL3

q I S F W L Q R K V A F

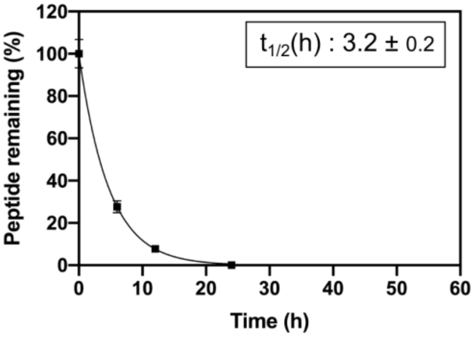

TL4

T I R F T N Q N K I A F

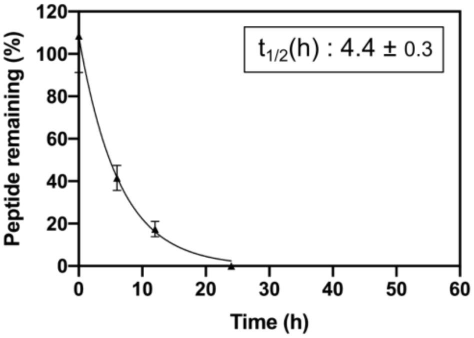

TL6

T n L F R L Q S K L A I

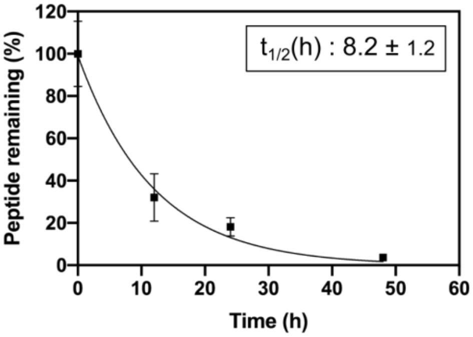

TL9

T n n F P R L G K V A Y

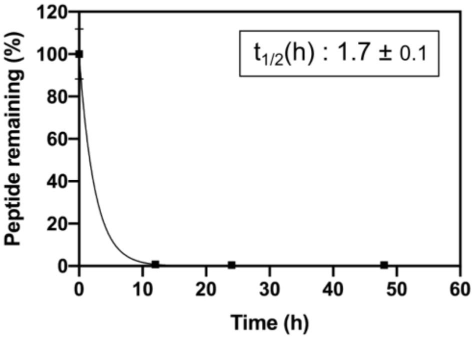

TL10

S N I V W q G Q G I I G

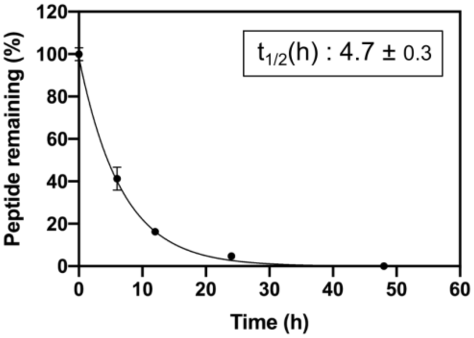

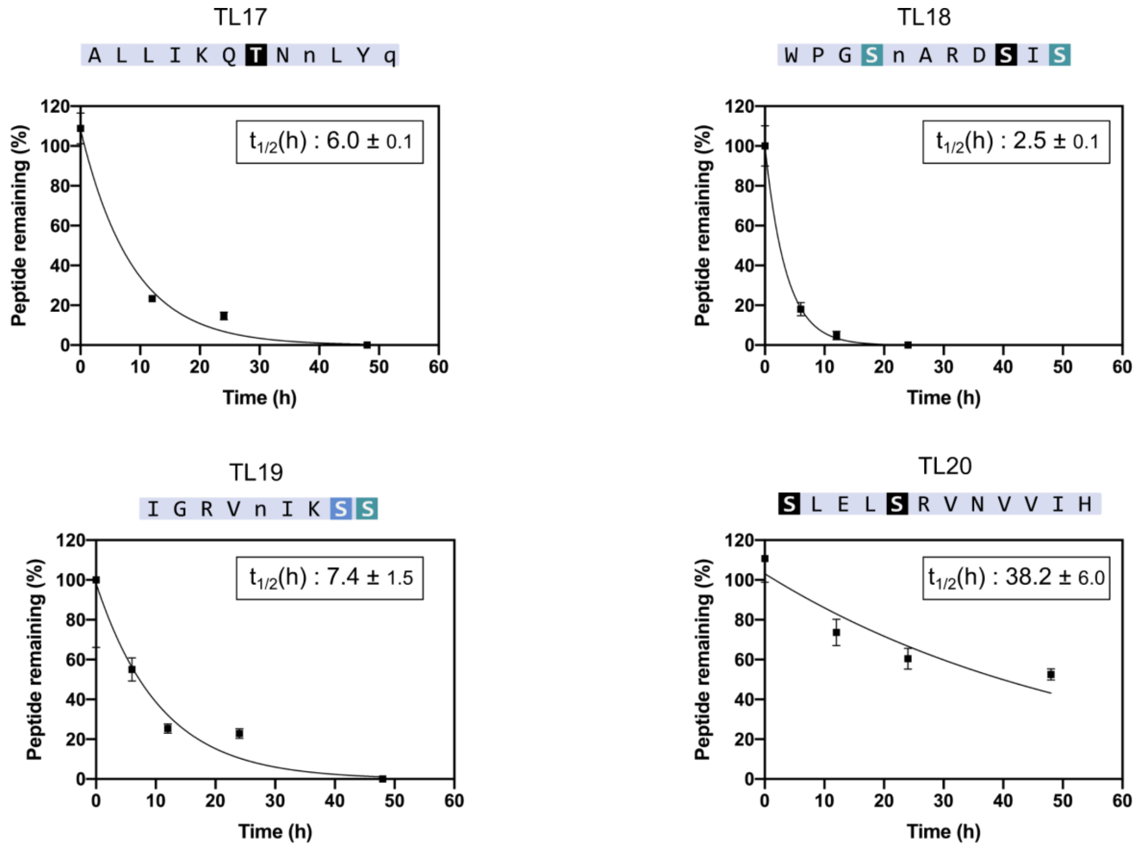

Figure S28. Serum stability of TLR10-targeting peptides. See section 2.12 for experimental details. Displayed are experimental data (points) and best fit (lines). An S highlighted in green indicates a Dha residue; S in blue – Oxz; n represents ^{L}norleucine (Nle); q represents ^{L}homoglutamine (^{h}Gln).

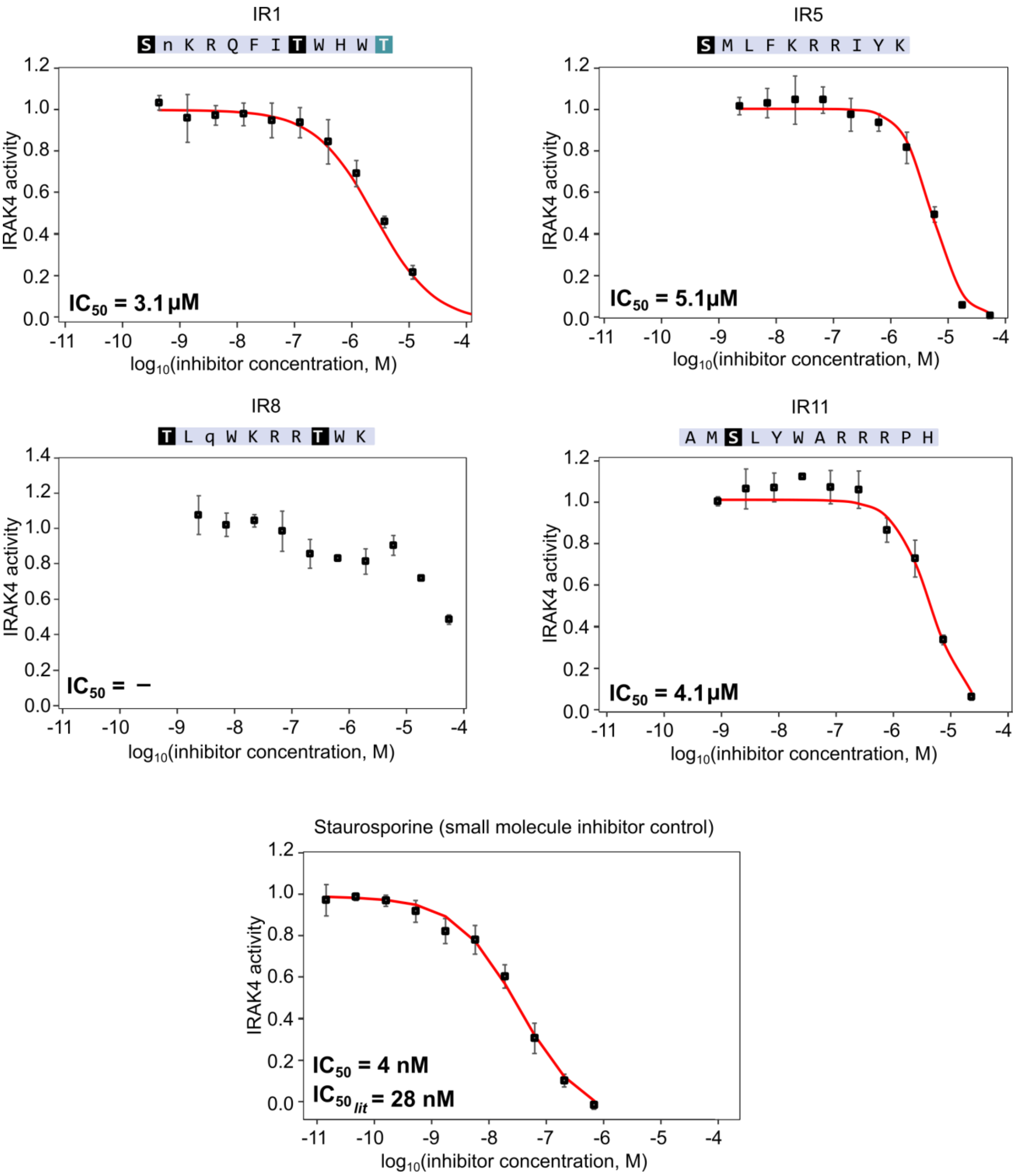

Figure S29. IRAK4 ADP-Glo inhibition curves for all synthesized compounds and Staurosporine small molecule inhibitor control.[17] See section 2.11 for experimental details. Experimental data are shown in black, and best fit in red. T highlighted in green indicates a Dhb residue; n represents ^{L}norleucine (Nle); q represents ^{L}homoglutamine (^{h}Gln).

.

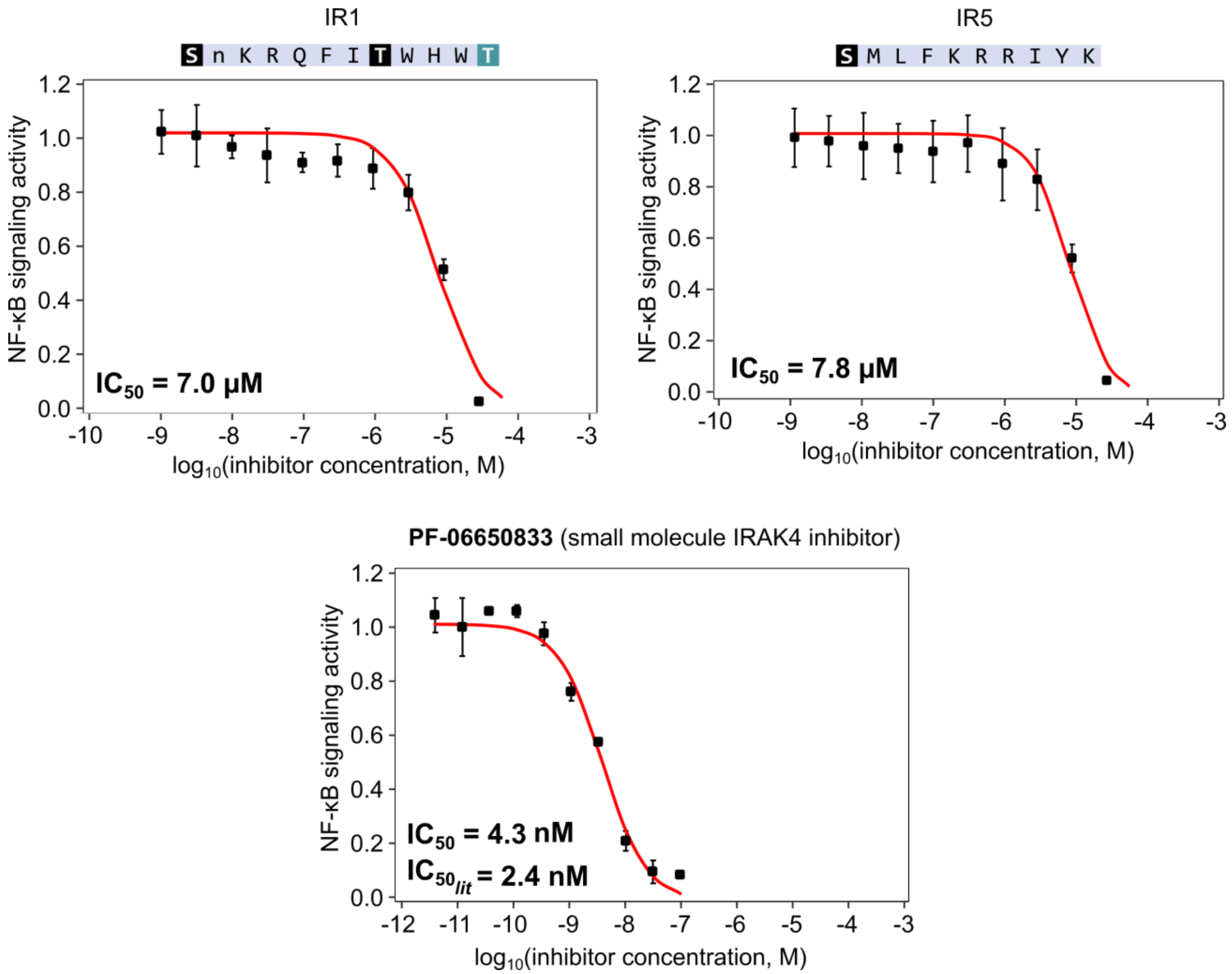

Figure S30. NF-kB signaling inhibition curves for IR1, IR5 and PF-06650833 a small molecule inhibitor control.[13] See section 2.13 for experimental details. Experimental data are shown in black, and best fit in red.

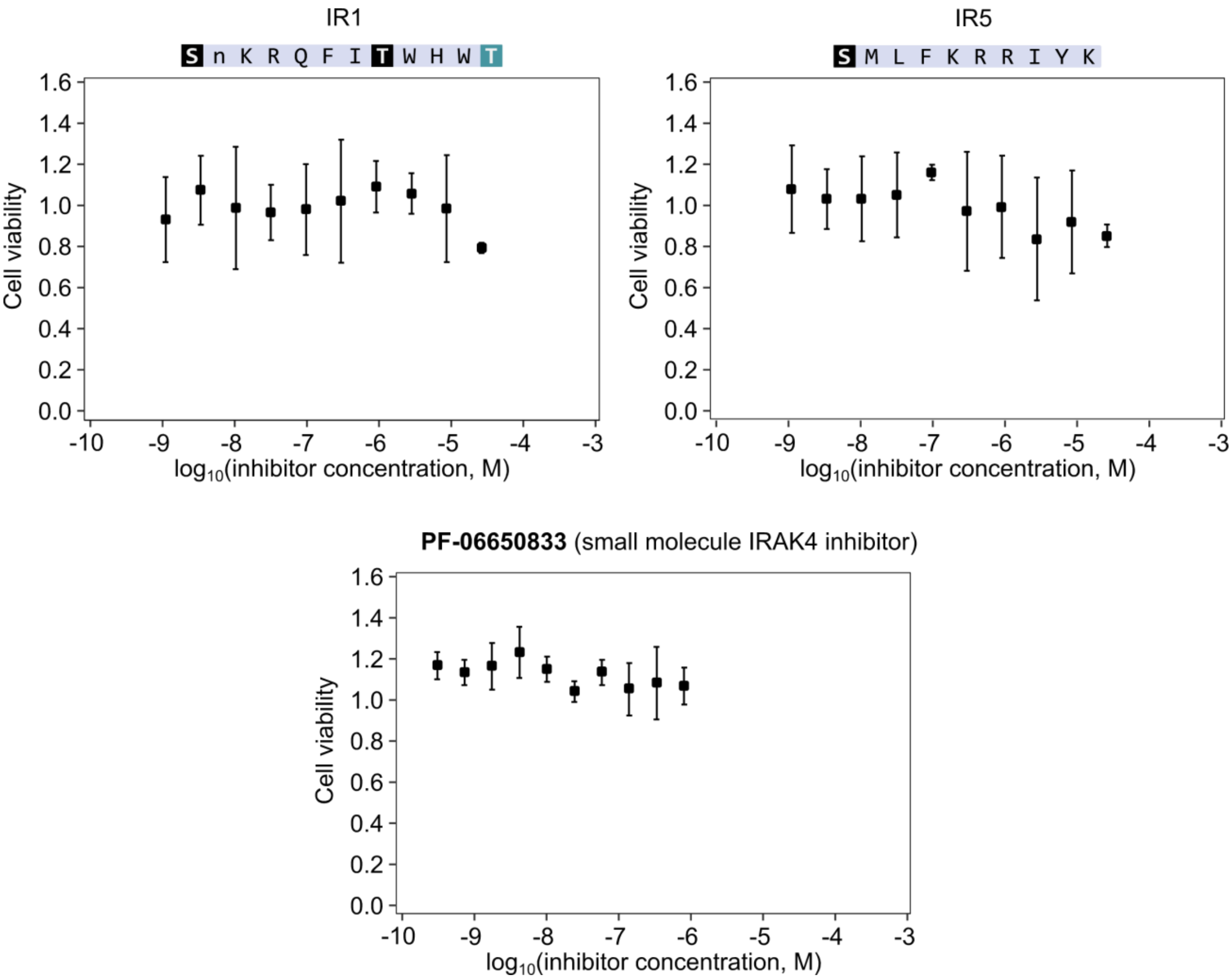

Figure S31. Cell viability curves for IR1, IR5 and PF-06650833 using Cell Counting Kit-8.[18] Experimental data are shown in black, and best fit in red. All the compounds are not cytotoxic at the tested concentrations.

4. Chemical synthesis of Thiopeptide Building Blocks

4.1. General Remarks and Experimental Details

All commercially available materials were used without further purification. Amino acids, coupling reagents, and resins were obtained from Sigma-Aldrich and TCI chemicals. All solvents were of reagent grade or HPLC grade (FUJIFILM Wako Pure Chemical Corporation). All anhydrous (H_2O ≤ 10 ppm) solvents were also obtained from FUJIFILM Wako Pure Chemical Corporation. Normal phase column chromatography was performed on Biotage Isolera™ Spektra flash purification system equipped with Biotage SNAP Ultra columns. ^{1}H and ^{13}C NMR spectra were recorded on JEOL ECS400 MHz spectrometers at 25°C. Chemical shifts for ^{1}H NMR are reported in parts per million (δ) relative to tetramethylsilane in deuterated solvent as internal standard. Chemical shifts for ^{13}C NMR are reported in parts per million (δ) relative to the solvent (e.g., $CDCl_3$, δ = 77.16). The following abbreviations are used for spin multiplicity: s = singlet, d = doublet, t = triplet, q = quadruplet, m = multiplet. High-resolution mass was performed on a Bruker MicrOTOF II TOF-MS mass spectrometer.

Reverse phase (RP) preparative HPLC separations utilized a mobile phase consisting of 0.1% trifluoroacetic acid (TFA) (v/v) in acetonitrile (Solvent B′) and 0.1% TFA (v/v) in water (Solvent A′) and were performed on a Prominence HPLC (Shimadzu) equipped with a Chromolith Prep RP-18 column (Merck) at a flow rate of 25 mL/min. Purification was performed using the method listed below.

HPLC method

0 – 3.5 min	10% B′
3.5 – 63.5 min	10 – 55% B′
63.5 – 65 min	55 – 95% B′
65 – 70 min	95% B′
70 – 75 min	10% B′

For LC/MS analysis, all separations utilized a mobile phase consisting of 0.1% formic acid (v/v) in acetonitrile (Solvent B) and 0.1% formic acid (v/v) in water (Solvent A). Analysis was performed on a Waters® SELECT SERIES H class/Cyclic IMS instrument equipped with ACQUITY UPLC® Peptide BEH C18 column (1.7 µm, 300 Å) at a flow rate of 0.3 mL/min and at 60°C with one of the two methods listed below.

Method 1

0 – 2 min	1% B
2 – 17 min	1 – 81% B
17 – 18.5 min	95% B
18.5 – 22 min	1% B

Method 2

0 – 1 min	1% B
1 – 9 min	1 – 81% B
9 – 10 min	95% B
10 – 12 min	1% B

For analytical UPLC analysis, all separations utilized a mobile phase consisting of Solvent B′ and Solvent A′. The analysis was performed on a Shimadzu Nexera X2 UHPLC System equipped with ACQUITY UPLC® BEH C18 1.7 µm column at a flow rate of 0.5 mL/min using the following method:

UPLC analysis method

0 – 2 min	5% B′
2 – 18 min	5 – 80% B′
18 – 20 min	80 – 95% B′
20 – 22 min	95% B′
22 – 25 min	5% B′

4.2. Synthesis of dehydrobutyrine building block

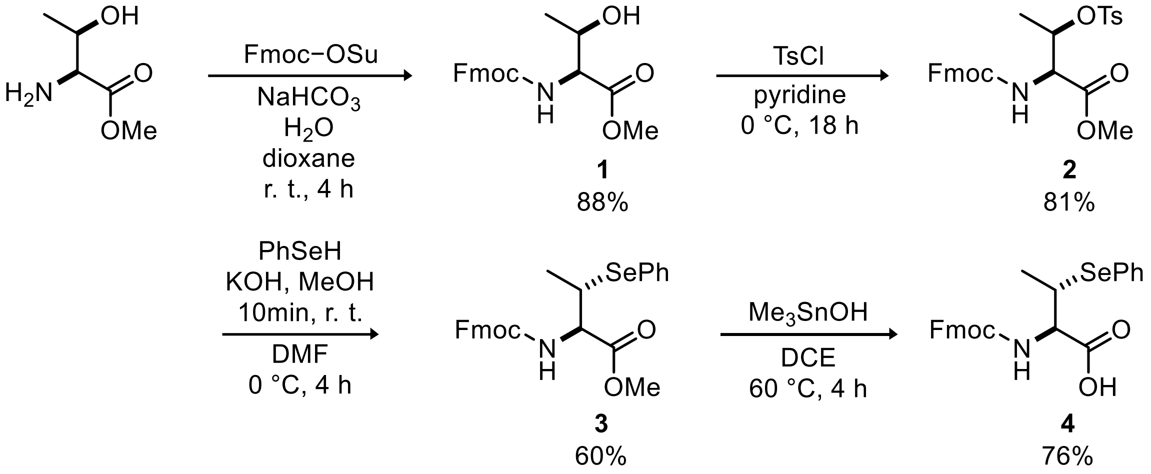

Compound **1 (Fmoc-^{L}Thr -OMe)** [methyl (((9*H*-fluoren-9-yl)methoxy)carbonyl)-D-threoninate]

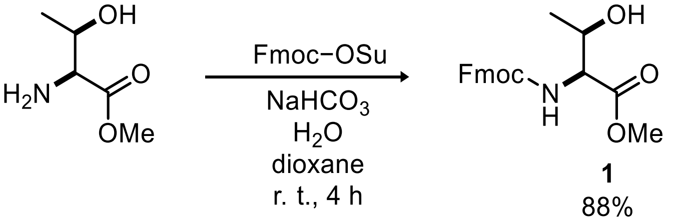

In a round bottom flask equipped with a magnetic stirring bar L-Threonine methyl ester hydrochloride (5.07 g; 30 mmol) was dissolved in water (50 mL), to which a solution of Fmoc-succinimide (Fmoc-OSu; 9.91 g; 29 mmol) in dioxane (75 mL) was added. To the resulting mixture was added solid $NaHCO_3$ (3.55 g; 42 mmol), and the suspension was vigorously stirred at room temperature for 4 h, after which more water (100 mL) was added. The product was extracted with ethyl acetate (100 mL x 3), and the combined organic layer was washed with brine (100 mL) and dried over Na_2SO_4. The compound was purified with silica gel chromatography using EA/*n*-hexane as the solvent system. The product **1** was isolated as a white solid (9.06 g; 25.5 mmol; 88%).

^{1}H NMR (400 MHz, DMSO-*d6*) δ 7.86 (d, *J* = 7.2 Hz, 2H), 7.72 (dd, *J* = 7.6, 2.4 Hz, 2H), 7.39 (t, *J* = 7.6 Hz, 2H), 7.30 (t, *J* = 7.2 Hz, 2H), 4.80 (d, *J* = 6.8 Hz, 1H), 4.30 (d, *J* = 7.2 Hz, 2H), 4.21 (t, *J* = 7.2 Hz, 1H), 4.09-4.06 (m, 2H), 3.61 (s, 3H), 1.08 (d, *J* = 6.4 Hz, 3H).

^{13}C NMR (101 MHz, DMSO-*d6*) δ 171.4, 156.4, 143.8, 140.7, 127.7, 127.1, 125.3, 120.1, 66.4, 65.9, 60.2, 51.9, 46.7, 40.1, 39.9, 39.7, 39.5, 39.3, 39.1, 38.9, 20.1.

ESI-TOF MS *m/z* for $[M+Na]^+$ ($C_{20}H_{21}NO_5$); calculated: 378.1317; found: 378.1316.

Compound **2 (Fmoc-^{L}Thr(OTs)-OMe)** [methyl N-(((9*H*-fluoren-9-yl)methoxy)carbonyl)-O-tosyl-D-threoninate]

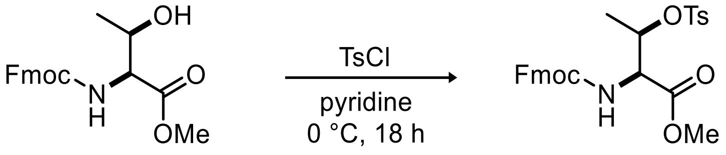

1 **2**
81%

A flame-dried round bottom flask equipped with a magnetic stirring bar was cooled to 0 °C and sequentially charged with Fmoc-^{L}Thr-OMe (9.06 g; 25.5 mmol), 4-toluenesulfonyl chloride (24.3 g; 127.5 mmol), and pyridine (45 mL). The mixture was stirred at this temperature for 18 h, after which ethyl acetate (300 mL) and water (300 mL) were added. The organic layer was separated and washed with 1M $KHSO_4$ (100 mL x 4), sat. $NaHCO_3$ (100 mL), brine (100 mL), and dried over Na_2SO_4. The compound was purified with silica gel chromatography using EA/*n*-hexane as the solvent system. The product **2** was isolated as a white solid (10.5 g; 20.7 mmol; 81%).

^{1}H NMR (400 MHz, $CDCl_3$) δ 7.79-7.76 (m, 4H), 7.61 (t, *J* = 6.8 Hz, 2H), 7.43-7.30 (m, 6H), 5.58 (d, *J* = 9.6 Hz, 1H), 5.19-5.14 (m, 1H), 4.50 (dd, *J* = 10, 2 Hz, 1H), 4.41 (d, *J* = 7.2 Hz, 2H), 4.23 (t, *J* = 7.2 Hz, 1H), 3.59 (s, 3H), 2.44 (s, 3H), 1.36 (d, *J* = 6.4 Hz, 3H).

^{13}C-NMR (101 MHz, $CDCl_3$) δ 169.3, 156.6, 145.2, 143.8, 143.7, 141.4, 133.6, 130.0, 127.9, 127.2, 125.2, 120.1, 78.5, 77.5, 77.4, 77.2, 76.9, 67.6, 58.1, 52.9, 47.2, 21.7, 18.2.

ESI-TOF MS *m/z* for $[M+Na]^+$ ($C_{27}H_{27}NO_7S$); calculated: 532.1406; found: 532.1403

Compound **3 (Fmoc-^{L}Thr(SePh)-OMe)** [methyl (2*S*,3*R*)-2-((((9*H*-fluoren-9-yl)methoxy)carbonyl)amino)-3-(phenylselanyl)butanoate]

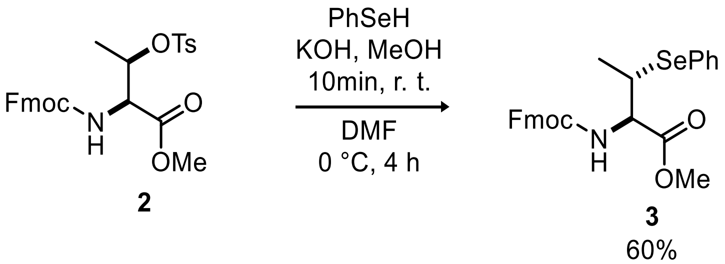

2 **3**
60%

In a flame-dried round bottom flask equipped with a magnetic stirring bar, KOH (2.1 g; 37.4 mmol) was dissolved in dry methanol (33 mL), and the flask was filled with nitrogen. To the resulting solution, phenylselenol (4.68 mL; 43.8 mmol) was added via syringe, and the mixture was stirred at room temperature for 10 min. The solvent was then evaporated under reduced pressure yielding potassium phenyl selenide as a yellow solid. The flask was immediately filled with nitrogen, and cooled to 0 °C. A cool (0 °C) solution of Fmoc-^{L}Thr(OTs)-OMe (9.69 g; 19 mmol) in DMF (83 mL) was then added to the flask via syringe, and the resulting bright orange solution was left to warm up to room temperate over a period of 4 h with constant stirring. The reaction was quenched with cold aqueous 1 M $KHSO_4$ (150 mL), and the product was extracted with ethyl acetate (400 mL). The organic layer was washed with sat. $NaHCO_3$ (100 mL), brine (100 mL), and

dried over Na_2SO_4. The compound was purified with silica gel chromatography using EA/*n*-hexane as the solvent system. The product **3** was isolated as a clear oil (5.64 g; 11.4 mmol; 60%).

^{1}H NMR (400 MHz, $CDCl_3$) δ 7.78 (d, *J* = 8 Hz, 2H), 7.64-7.631 (m, 4H), 7.42 (t, *J* = 7.2 Hz, 2H), 7.36-7.25 (m, 5H), 5.73 (d, *J* = 8.8 Hz, 1H), 4.69 (q, *J* = 4.1 Hz, 1H), 4.47-4.38 (m, 2H), 4.26 (t, *J* = 6.8 Hz, 1H), 3.74-3.70 (m, 4H), 1.50 (d, *J* = 6.8 Hz, 3H).

^{13}C NMR (101 MHz, $CDCl_3$) δ 171.1, 156.1, 144.0, 143.9, 141.4, 135.6, 129.3, 128.4, 127.9, 127.3, 125.3, 120.2, 77.7, 77.4, 77.1, 67.4, 58.7, 52.6, 47.3, 41.1, 18.4.

ESI-TOF MS *m/z* for $[M+Na]^+$ ($C_{26}H_{25}NO_4Se$); calculated: 518.0846; found: 518.0845.

Compound **4 (Fmoc-^{L}Thr(SePh)-OH)** [(2*S*,3*R*)-2-((((9*H*-fluoren-9-yl)methoxy)carbonyl)amino)-3-(phenylselanyl)butanoic acid]

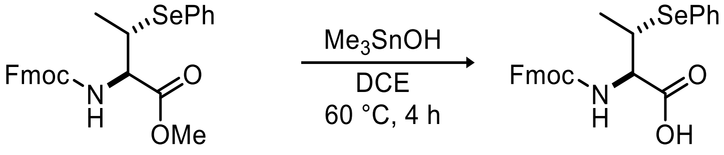

3 **4** 76%

A flame-dried two-necked round bottom flask equipped with a magnetic stirring bar and a reflux condenser was charged with Fmoc-^{L}Thr(SePh)-OMe (4.86 g; 9.8 mmol) and trimethyltin hydroxide (7.1 g; 39 mmol), and filled with nitrogen. Anhydrous dichloroethane (122 mL) was added via syringe and the mixture was heated to 60 °C. After 4 h of stirring at this temperature, TLC indicated full consumption of the starting material. The mixture was cooled down to room temperature and the solvent was evaporated under reduced pressure. The residue was redissolved in ethyl acetate (150 mL), extracted with 1 M $KHSO_4$ (200 mL x 4), washed with brine (100 mL) and dried over Na_2SO_4. The compound was purified with silica gel chromatography using DCM/MeOH as the solvent system. The product **4** was isolated as a white solid (3.56 g; 7.4 mmol; 76%).

^{1}H NMR (400 MHz, DMSO-*d$_6$*) δ 7.91 (d, *J* = 7.2 Hz, 2H), 7.79 (d, *J* = 7.2 Hz, 2H), 7.59-7.57 (m, 2H), 7.44 (t, *J* = 7.2 Hz, 2H), 7.37-7.33 (m, 5H), 4.38-4.24 (m, 4H), 3.77-3.72 (1H), 1.38 (d, *J* = 7.2 Hz, 3H)

^{13}C NMR (101 MHz, DMSO-*d$_6$*) δ 172.2, 157.0, 144.3, 141.2, 134.7, 129.8, 129.2, 128.3, 128.2, 127.6, 126.0, 125.9, 120.6, 66.5, 58.7, 47.2, 40.7, 40.5, 40.3, 40.1, 39.9, 39.6, 39.4, 17.8

ESI-TOF MS *m/z* for $[M+Na]^+$ ($C_{25}H_{23}NO_4Se$); calculated: 504.0690; found: 504.0694.

4.3. Synthesis of dehydroalanine-oxazole building block

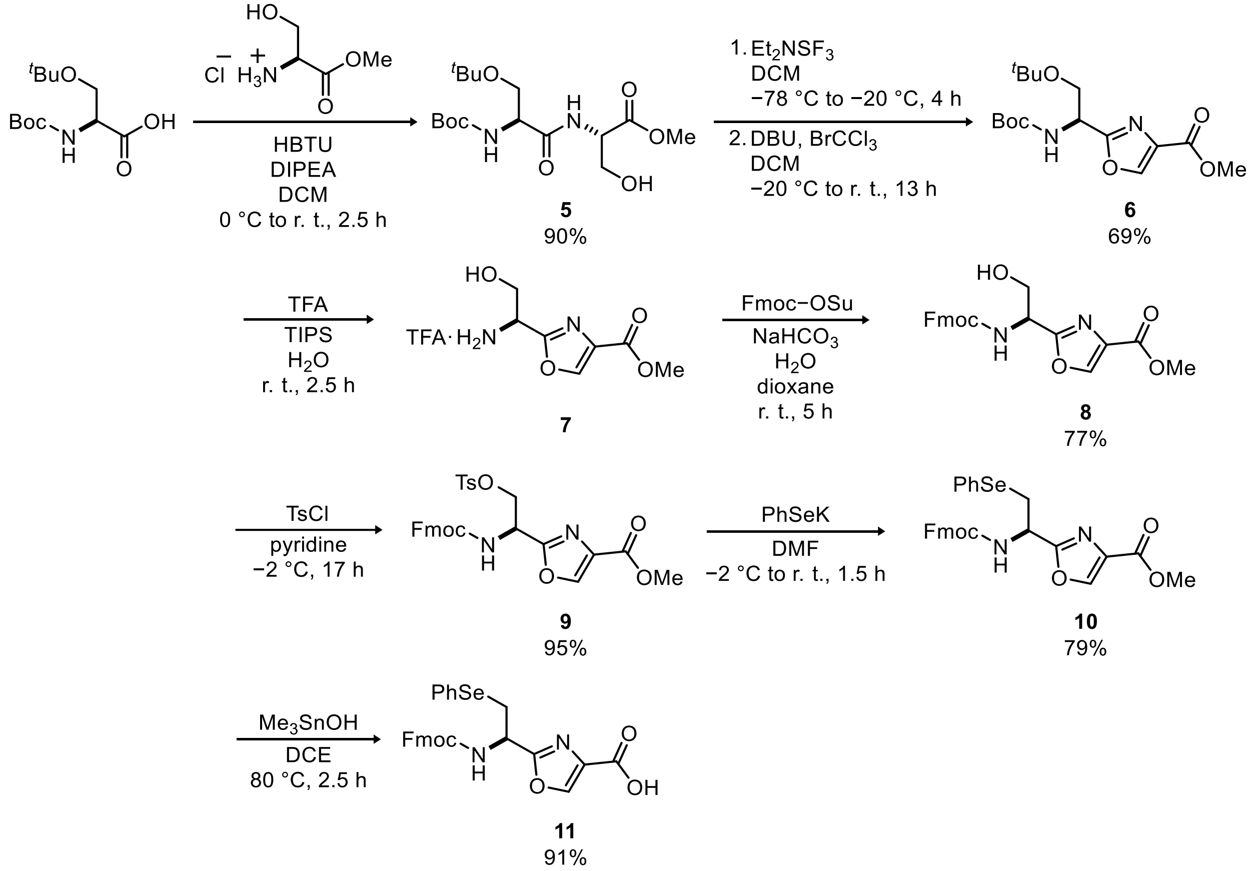

Compound **5, 6,** and **7** was synthesized according to reported synthetic procedures.[19] The detailed synthesis and isolation of the product is proceeded in the same manner and the characterization of the compounds agreed with the reported literature.

Compound **8** **(Fmoc-^{L}Ser(OH)-Oxz-OMe)** [methyl (*S*)-2-(1-((((9*H*-fluoren-9-yl)methoxy)carbonyl)amino)-2-hydroxyethyl)oxazole-4-carboxylate]

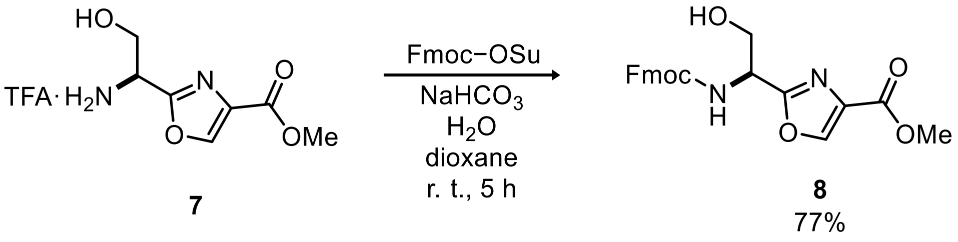

In a round bottom flask equipped with a magnetic stirring bar ^{L}Ser(OH)-Oxz-OMe trifluoroacetate (3.62 g; 12 mmol) was dissolved in water (35 mL), to which a solution of Fmoc-succinimide (Fmoc-OSu; 4.72 g; 14 mmol) in dioxane (25 mL) was added. To the resulting mixture was added solid $NaHCO_3$ (2.52 g; 30 mmol), and the suspension was vigorously stirred at room temperature for 5

h, after which more water (100 mL) was added. The product was extracted with ethyl acetate (75 mL x 3), and the combined organic layer was washed with brine (50 mL) and dried over Na_2SO_4. The compound was purified with silica gel chromatography using EA/*n*-hexane as the solvent system. The product **8** was isolated as a white solid (3.80 g; 9.3 mmol; 77%).

^{1}H NMR (400 MHz, DMSO-*d$_6$*) δ 8.82 (s, 1H), 8.01 (d, *J* = 8.4 Hz, 1H), 7.89 (d, *J* = 7.2 Hz, 2H), 7.72 (dd, *J* = 6.8, 4.4 Hz, 2H), 7.41 (t, *J* = 7.2 Hz, 2H), 7.34-7.31 (m, 2H), 5.10 (t, *J* = 5.6 Hz, 1H), 4.79 (q, *J* = 6.4 Hz, 1H), 4.32 (d, *J* = 7.2 Hz, 2H), 4.23 (d, *J* = 6.8 Hz, 1H), 3.83-3.72 (m, 4H)

^{13}C NMR (101 MHz, DMSO-*d$_6$*) δ 164.1, 161.7, 156.5, 146.1, 144.3, 141.3, 132.8, 128.2, 127.6, 125.8, 120.6, 66.4, 62.2, 52.3, 52.1, 47.1, 40.7, 40.4, 40.2, 40.0, 39.8, 39.6, 39.4

ESI-TOF MS *m/z* for $[M+Na]^+$ ($C_{22}H_{21}N_2O_6$); calculated: 431.1216; found: 431.1219.

Compound **9** **(Fmoc-^{L}Ser(OTs)-Oxz-OMe)** [methyl (*S*)-2-(1-((((9*H*-fluoren-9-yl)methoxy)carbonyl)amino)-2-(tosyloxy)ethyl)oxazole-4-carboxylate]

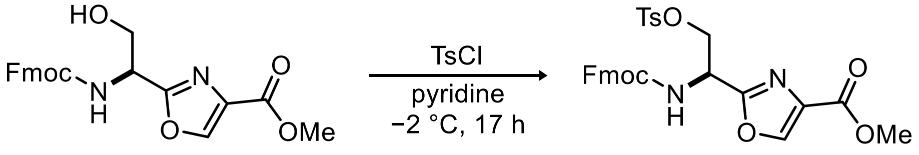

8 **9**
95%

A flame-dried round bottom flask equipped with a magnetic stirring bar was cooled to –2 °C and sequentially charged with Fmoc-^{L}Ser(OH)-Oxz-OMe (3.2 g; 7.8 mmol), 4-toluenesulfonyl chloride (7.47 g; 39.2 mmol), and pyridine (21 mL). The mixture was stirred at this temperature for 16 h, after which ethyl acetate (150 mL) and water (160 mL) were added. The organic layer was separated and washed with 1M $KHSO_4$ (100 mL x 4), 0.2 M $NaHCO_3$ (80 mL), brine (100 mL), and dried over Na_2SO_4. The compound was purified with silica gel chromatography using EA/*n*-hexane as the solvent system. The product **9** was isolated as a white solid (4.19 g; 7.44 mmol; 95%).

^{1}H-NMR (400 MHz, DMSO-*d$_6$*) δ 8.82 (s, 1H), 8.33 (d, *J* = 8.4 Hz, 1H), 7.90 (d, *J* = 7.6 Hz, 2H), 7.75-7.69 (m, 4H), 7.44-7.31 (m, 6H), 5.11 (q, *J* = 6.8 Hz, 1H), 4.40-4.19 (m, 5H), 3.81 (s, 3H), 2.34 (s, 3H)

^{13}C-NMR (101 MHz, DMSO-*d$_6$*) δ 161.4, 161.4, 156.1, 146.5, 145.7, 144.2, 144.2, 141.3, 132.8, 132.2, 130.7, 128.2, 127.6, 125.8, 120.7, 68.8, 66.6, 52.4, 48.6, 47.0, 40.7, 40.5, 40.3, 40.1, 39.9, 39.6, 39.4, 21.6

ESI-TOF MS *m/z* for $[M+Na]^+$ ($C_{29}H_{27}N_2O_8S$); calculated: 585.1300; found: 585.1304.

Compound **10 (Fmoc-^{L}Sec(Ph)-Oxz-OMe)** [methyl (*R*)-2-(1-((((9*H*-fluoren-9-yl)methoxy)carbonyl)amino)-2-(phenylselanyl)ethyl)oxazole-4-carboxylate]

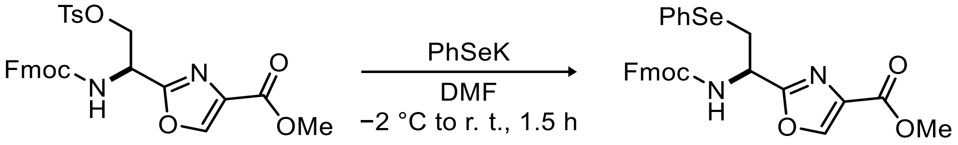

9 **10** 79%

In a flame-dried round bottom flask equipped with a magnetic stirring bar, KOH (718 mg; 12.8 mmol) was dissolved in dry methanol (10.2 mL), and the flask was filled with nitrogen. To the resulting solution, phenylselenol (1.56 mL; 14.7 mmol) was added via syringe, and the mixture was stirred at room temperature for 10 min. The solvent was then evaporated under reduced pressure yielding potassium phenyl selenide as a yellow solid. The flask was immediately filled with nitrogen, and cooled to –2 °C. A cool (–2 °C) solution of Fmoc-^{L}Ser(OTs)-Oxz-OMe (3.6 g; 6.4 mmol) in DMF (12.8 mL) was then added to the flask via syringe, and the resulting bright orange solution was left to warm up to room temperate over a period of 1.5 h with constant stirring. The reaction was quenched with 1 M $KHSO_4$ (140 mL), and the product was extracted with ethyl acetate (200 mL). The organic layer was washed with saturated $NaHCO_3$ (80 mL), brine (50 mL), and dried over Na_2SO_4. The compound was purified with silica gel chromatography using EA/*n*-hexane as the solvent system. The product **10** was isolated as a white solid (2.80 g; 5.1 mmol; 79%).

^{1}H NMR (400 MHz, $CDCl_3$) δ 8.01 (s, 1H), 7.77 (d, *J* = 7.6 Hz, 2H), 7.57-7.57 (m, 2H), 7.48-7.46 (m, 2H), 7.41 (t, *J* = 7.2 Hz, 2H), 7.34-7.29 (m, 2H), 7.22-7.2 (m, 3H), 5.76 (d, *J* = 8.4 Hz, 1H), 5.30-5.27 (m, 1H), 4.38 (d, *J* = 6.8 Hz, 2H), 4.19 (t, *J* = 6.8 Hz, 1H), 3.91 (s, 3H), 3.45 (d, *J* = 5.2 Hz, 2H)

^{13}C NMR (101 MHz, $CDCl_3$) δ 163.3, 161.3, 155.5, 144.2, 143.8, 143.7, 141.4, 133.7, 133.4, 129.3, 127.9, 127.7, 127.2, 125.2, 120.1, 77.5, 77.4, 77.2, 76.9, 67.4, 52.3, 49.7, 47.2, 31.8

ESI-TOF MS *m/z* for $[M+Na]^+$ ($C_{28}H_{25}N_2O_5Se$); calculated: 571.0745; found: 571.0748.

Compound **11 (Fmoc-^{L}Sec(Ph)-Oxz-OH)** [(*R*)-2-(1-((((9*H*-fluoren-9-yl)methoxy)carbonyl)amino)-2-(phenyl-selanyl)ethyl)oxazole-4-carboxylic acid]

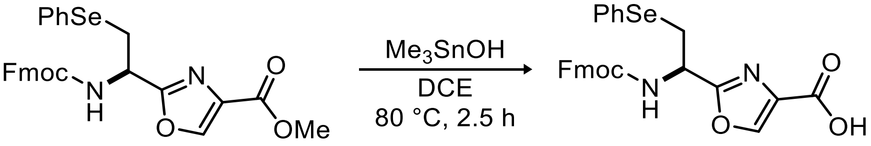

10 **11** 91%

A flame-dried two-necked round bottom flask equipped with a magnetic stirring bar and a reflux condenser was charged with Fmoc-^{L}Sec(Ph)-Oxz-OMe (2.75 g; 5 mmol) and trimethyltin hydroxide (2.71 g; 15 mmol), and filled with nitrogen. Anhydrous dichloroethane (50 mL) was added via syringe and the mixture was heated to 80 °C. After 2.5 h of stirring at this temperature,

TLC indicated full consumption of the starting material. The mixture was cooled down to room temperature and the solvent was evaporated under reduced pressure. The residue was redissolved in ethyl acetate (150 mL), extracted with 1 M $KHSO_4$ (200 mL x 4), washed with brine (80 mL) and dried over Na_2SO_4. After filtration and evaporation of the solvent, the solid was triturated with a mixture of diethyl ether and *n*-hexane (1 : 9, v/v; 50 mL x 3). Upon drying under reduced pressure, title compound **11** was obtained as a crystalline white solid (2.44 g; 4.57 mmol; 91%).

^{1}H NMR (400 MHz, DMSO-d_6) δ 8.68 (s, 1H), 8.27 (d, *J* = 8.0 Hz, 1H), 7.89 (d, *J* = 7.2 Hz, 2H), 7.70 (d, *J* = 7.2 Hz, 2H), 7.52-7.50 (m, 2H), 7.41 (t, *J* = 7.6 Hz, 2H), 7.34-7.25 (m, 5H), 4.88 (q, *J* = 8.0 Hz, 1H), 4.37-4.21 (m, 3H), 3.48-3.32 (m, 2H).

^{13}C NMR (101 MHz, DMSO-d_6) δ 163.8, 162.4, 156.2, 145.9, 144.2, 141.3, 133.8, 132.6, 129.9, 129.7, 128.2, 127.7, 127.6, 125.8, 120.7, 66.4, 49.8, 47.1, 40.7, 40.5, 40.3, 40.1, 39.9, 39.6, 39.4, 29.6

ESI-TOF MS *m/z* for $[M+Na]^+$ ($C_{27}H_{23}N_2O_5Se$); calculated: 557.0588; found: 557.0592.

5. Total synthesis of lactazole-like thiopeptides

5.1. General procedure for thiopeptide synthesis

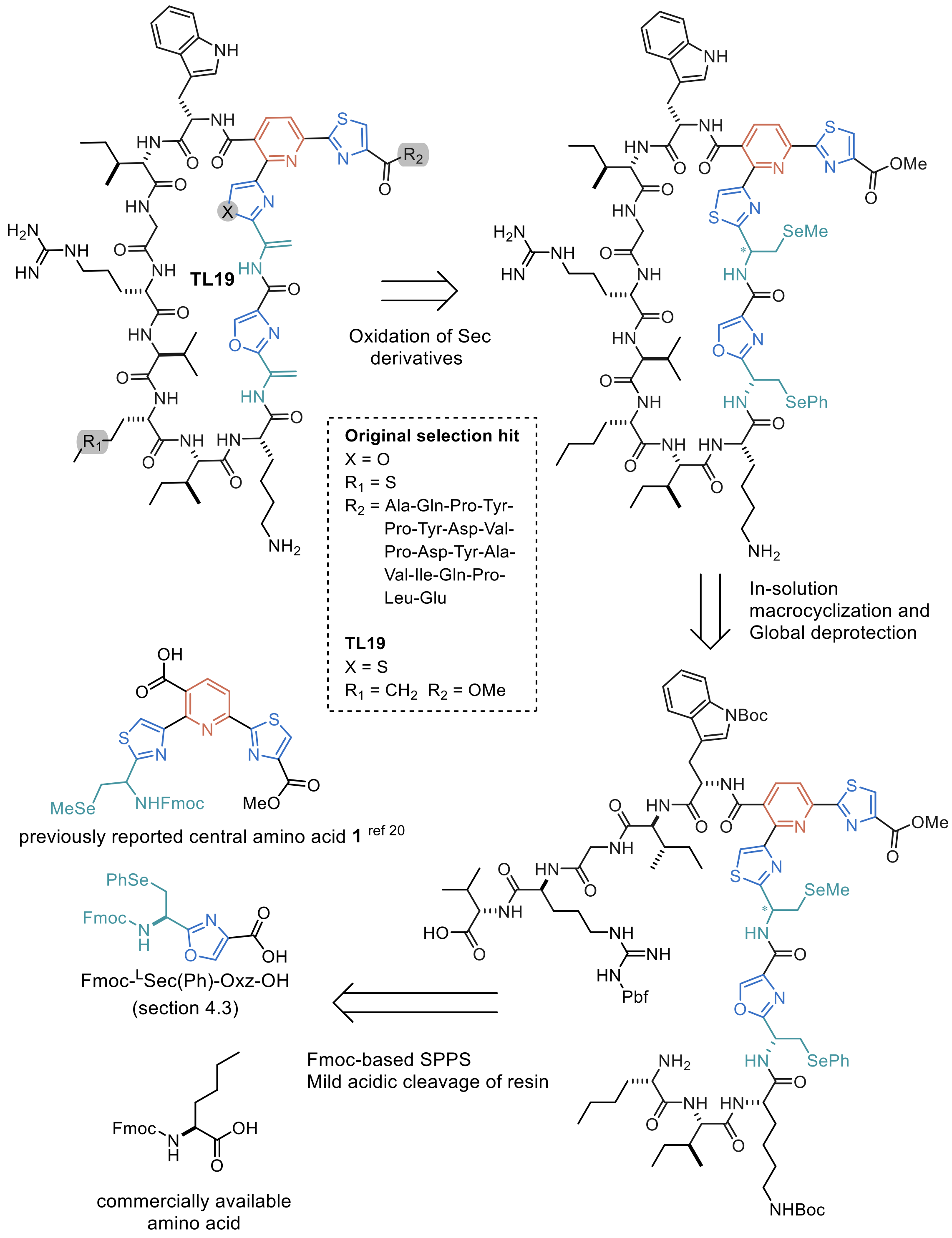

Figure S32. A general procedure for the total synthesis of lactazole-like thiopeptides analog. Exemplified here is TL19, a de novo discovered lactazole-like binder of TLR10 and its retrosynthesis.

All the lactazole-like thiopeptide analogs was synthesized according to the general procedure as reported before.[20] This approach utilized the essential amino acid building block (as described in section 4 and Fig. S32) for solid phase peptide synthesis (SPPS) chain assembly, and the linear SPPS products were macrocyclized in solution. Lastly, the Se-alkylated selenocysteine derivatives were oxidized to yield the Dha/Dhb containing thiopeptides. To simplify the synthesis, there were three major modifications, (i) truncation of tail amino acids to -OMe, (ii) mutation of internal oxazole (Oxz) to thiazole (Thz), and (iii) mutation of Met and Cys-IAA to its carbon analogs (Nle and ^{h}Gln respectively) to mitigate the sulfoxide formation during the final oxidation step. Combined, this total chemical synthesis approach enables us to prepare all candidate compounds with most in multi-mg quantities as detailed below.

5.1.1. General procedure 1: Loading of first amino acid to 2-chlorotrityl chloride resin

Synthesis was performed manually on 2-chlorotrityl chloride resin (100¬ – 200 mesh, 1% DVB; Novabiochem®, Merck; loading: 1.00 – 1.80 mmol/g). First, anhydrous DCM (1.5 mL) was used to swell the resin (25 mg) for 15 min. To the swollen resin, a solution of the first amino acid (4 eq. to the initial resin loading) and *N,N*-diisopropylethylamine (DIPEA; 8 eq.) in anhydrous DCM (1 – 1.5 mL) was added. The reaction mixture was shaken at room temperature for 2 h. After aspiration, the resin was then washed with DCM (3 × 2 mL) and DMF (3 × 2 mL). The resin was subsequently submitted to iterative peptide assembly via the Fmoc-based SPPS protocol described below.

5.1.2. General procedure 2: Fmoc-based solid-phase peptide synthesis (SPPS) of lactazole-like thiopeptides

The iterative amino acid coupling to the pre-loaded resin (25 mg) was performed using a mixture of an Fmoc-amino acid (4.5 eq.), 1-[bis(dimethylamino)methylene]-1*H*-1,2,3-triazolo[4,5-b]pyridinium 3-oxide hexafluorophosphate (HATU; 4.0 equiv.) and *N,N*-diisopropylethylamine (DIPEA; 8.0 equiv.) in DMF (1 mL) for 1 h at room temperature. The following side chain protecting scheme was utilized for proteinogenic amino acids: Arg(Pbf), Asn(Trt), Asp(O^{t}Bu), Gln(Trt), Glu(O^{t}Bu), His(Boc), Lys(Boc), Ser(^{t}Bu), Thr(^{t}Bu), Trp(Boc), and Tyr(^{t}Bu). Compound **4** was utilized for the synthesis of IR1; compound **11** was utilized for the synthesis of TL19. After coupling, the resin was then washed with DMF (3 × 2 mL), DCM (3 × 2 mL) and DMF (3 × 2 mL). Fmoc deprotection was carried out using 1 mL of 20% piperidine in DMF at room temperature for 15 min, and the resin was then washed with DMF (3 × 2 mL), DCM (3 × 2 mL) and DMF (3 × 2 mL). (*Note: The above procedure was used for SPPS prior to the coupling of* central amino acid **1**).

For the coupling of central amino acid **1**, a solution of **1** (1.2 eq. to the resin loading), benzotriazol-1-yloxytripyrrolidinophosphonium hexafluorophosphate (PyBOP) (1.2 eq.), DIPEA (2.4 eq.) in anhydrous DMF (0.4 – 0.6 mL) was gently agitated on shaker with the resin at room temperature for 12 h. The resin was then washed with DMF (3 × 2 mL) and DCM (2 mL). Residual unreacted amine was capped using the DCM : pyridine : acetic anhydride (1 mL; 2 : 1 : 1, v : v : v) mixture for 30 min at room temperature, after which the solution was aspirated and resin was

washed by DMF (5 × 2 mL). Fmoc deprotection after the coupling of pyridine building block **1** was shortened to 2 × 3 min treatments (20% piperidine in DMF, 2 mL each time). After Fmoc deprotection, the resin was washed with DMF (3 × 2 mL), DCM (3 × 2 mL) and DMF (3 × 2 mL), and the next amino acid was coupled as described above.

5.1.3. General procedure 3: C-terminal cleavage of the side-chain protected peptide from the resin

The resin was washed with DCM and subjected to a mild acidic cleavage cocktail consisting of DCM / AcOH / TFE (8 : 1 : 1, v : v : v) for 2 h at room temperature. Following filtration, the cleavage solution was collected, and the resin was washed with DCM twice. The combined solution was reduced on a rotary evaporator. The residue was co-evaporated with *n*-hexane to remove residual acetic acid, resulting in the crude protected peptide bearing the free carboxylic acid at the C-terminus. The compound was further dried under vacuum overnight prior to macrocyclization.

5.1.4. General procedure 4: In-solution macrocyclization

In a 25 mL round bottom flask, crude protected linear peptide (1 eq.) was dissolved in anhydrous DCM (0.2 mM) in an ice bath. A mixture of *N,N*-diisopropylethylamine (DIPEA; 12 eq.), 1-hydroxy-7-azabenzotriazole (HOAt; 6 eq.) and ethyl cyano(hydroxyimino)acetate (OxymaPure; 6 eq.) in anhydrous DCM / DMF (10 : 1; v : v) was added to the reaction solution, and the contents were stirred at 0 °C for 10 min. Then, 1-[bis(dimethylamino)methylene]-1*H*-1,2,3-triazolo[4,5-b]pyridinium 3-oxide hexafluorophosphate (HATU; 10 eq.) was added, and the solution was slowly warmed to the room temperature and stirred for another 12 h. After, the solvent was removed on a rotary evaporator and the cyclic peptide was further dried under vacuum. The crude cyclic peptide was used directly in the next step.

5.1.5. General procedure 5: Global deprotection

Global cleavage cocktail (TFA : TIPS : H_2O = 95 : 2.5 : 2.5, v : v : v) was freshly prepared and added to the crude cyclic peptide residue, and the resulting mixture was stirred at room temperature for 2 h. For peptides containing arginine and methionine, thioanisole was added to the deprotection reagent (TFA : TIPS : H_2O : thioanisole = 92.5 : 2.5 : 2.5 : 2.5; v : v : v : v). The deprotection solution was concentrated and the crude peptide was precipitated with and then washed by ice cold diethyl ether. The crude product was dried under vacuum to remove residual solvent.

5.1.6. General procedure 6: Oxidative elimination

Mild oxidation of the selenocysteine derivatives and elimination of the selenoxide furnished dehydroalanine-containing thiopeptides. Unprotected macrocyclic thiopeptide precursor was dissolved in DMSO and diluted with 30% ACN in phosphate buffer (pH 8; 1 mM). Then *tert*-butyl hydroperoxide (70 wt. in water) was added to (100 mM; 200 mM was used for TL18 (contains three Sec residues); 30 mM – 50 mM were used for peptides containing Met) and the reaction

was shaken at room temperature until completion as determined by RP-LCMS. (*Note: the initial reaction solution could be cloudy, but the precipitate will gradually dissolve during the reaction*) Upon full conversion, the reaction solution was acidified by TFA (0.1%), purified by preparative RP-HPLC, and lyophilized to give the final lactazole-like thiopeptide as a white solid.

The oxidative elimination procedure was modified for thiopeptides containing Dhb amino acid building block because *tert*-butyl hydroperoxide oxidative elimination procedures were too mild. Unprotected macrocyclic thiopeptide precursor was dissolved in DMSO and diluted with 50% ACN in phosphate buffer (pH 8; 1 mM). Then $NaIO_4$ (f.c. 4 mM dissolved in phosphate buffer) was added and the reaction solution was incubated at 25 °C. The reaction was completed in 10 min as confirmed by LC/MS, quenched with 1 volume of 20 mM hydrazine, purified by preparative RP-HPLC, and lyophilized to give the final lactazole-like thiopeptide as a white solid.

5.1.7. General procedure 7: Selective hydrolysis of thiazole tail methyl ester on fully protected thiopeptide

Upon completion of the macrocyclization reaction, the mixture was diluted with DCM (50 –100 mL) and washed with 0.1 M HCl (3 × 50 mL) and brine (30 mL), and then dried over anhydrous sodium sulfate. The residue was concentrated on a rotary evaporator, passed through a silica plug using DCM : MeOH = 10 : 1 (v : v) as the eluent to remove excess coupling reagents, and dried under vacuum. In a 25 mL round bottle flask, protected cyclic peptide was dissolved in anhydrous 1,2-dichloroethane (0.016 M) at room temperature. Trimethyltin hydroxide (Me_3SnOH; 9.5 eq.) was added, and the mixture was stirred for 1 h at 80 °C. After, another 18 eq. of Me_3SnOH were added, and the reaction was stirred at 80 °C for overnight. Upon full conversion as indicated by RP-LCMS analysis (deprotected sample), the reaction was cooled to room temperature and diluted with DCM (100 mL). The organic phase was washed with aqueous solution of potassium hydrogen sulfate (0.01 M, 3 × 20 mL) and brine (50 mL), and then dried over anhydrous sodium sulfate. The solvent was removed under vacuum. The crude protected cyclic peptide with free tail carboxylic acid was obtained as light-yellow to off-white solid and used directly in next step.

5.1.8. General procedure 8: Functionalization of thiopeptides in the carboxylate tail

The protected cyclic thiopeptide bearing free carboxylate tail was dissolved in anhydrous DMF (0.01 M). An amine coupling partner (e.g., ct-NH_2; 5 eq.), 1-ethyl-3-(3-dimethylaminopropyl)carbodiimide (EDCI; 4.5 eq.), 1-hydroxy-7-azabenzotriazole (HOAt; 4.5 eq.) and *N,N*-diisopropylethylamine (DIPEA; 10 eq.) were added sequentially at 0 °C. The mixture was slowly warmed to the room temperature and stirred for 12 h. After full conversion, the reaction was diluted with DCM, washed with 0.1 M HCl solution (2 × 20 mL), aqueous lithium chloride (5 % w/v, 3 × 20 mL) and brine (50 mL), dried over anhydrous sodium sulfate. The solvent was removed under vacuum, and the residue was ready for the following global deprotection (general procedure 5).

5.2. Total synthesis of lactazole-like thiopeptide

5.2.1 Synthesis of thiopeptide IR1

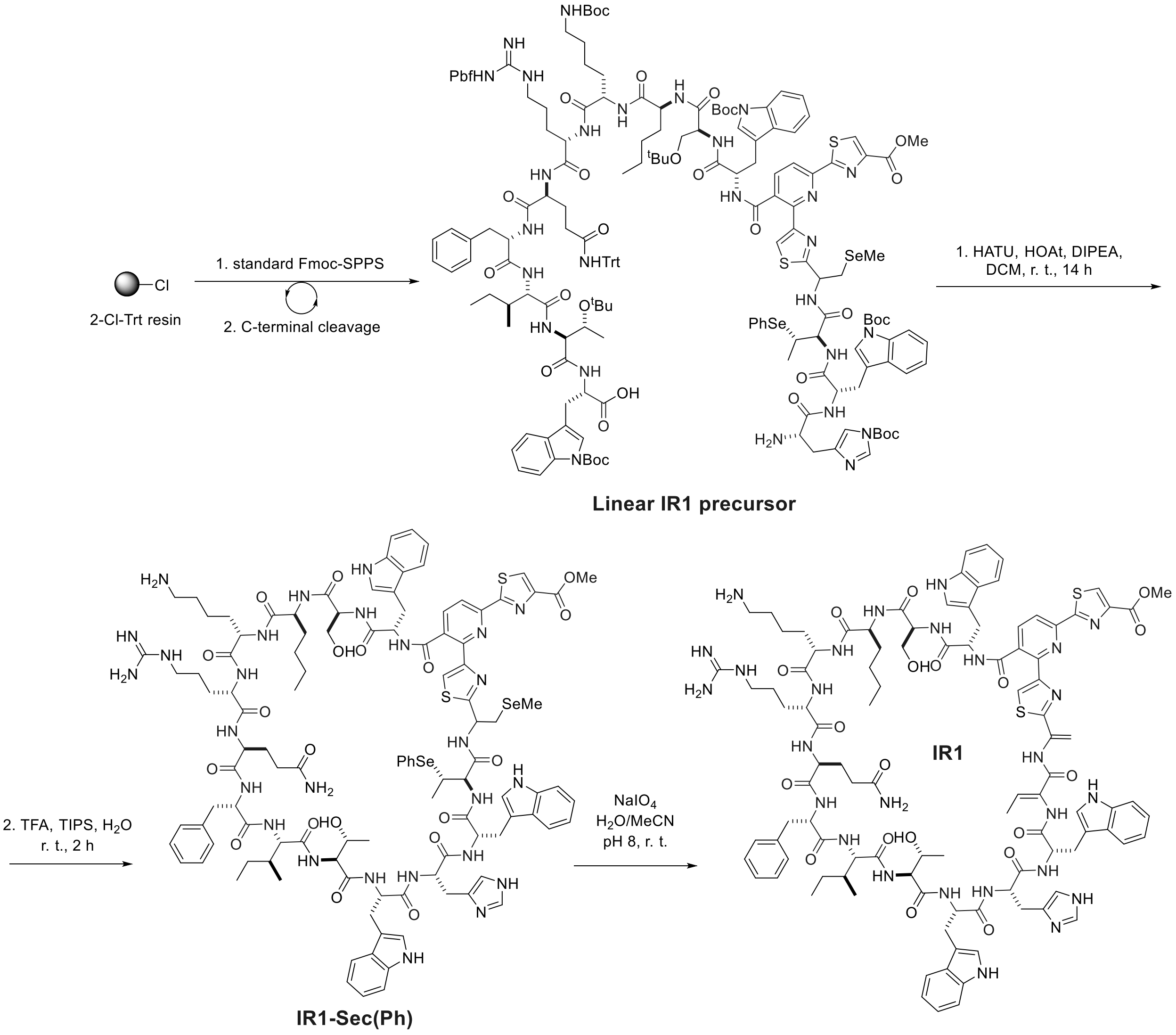

IR1 was synthesized according to general procedures as reported before.[20] Oxidative elimination was performed as explained in 5.1.6. The product was purified by RP-HPLC using HPLC method as described in general remarks 5.1 and lyophilized as a white solid (4.1 mg, 7% yield based on the resin loading), the purity was determined to be 96% by analytical UPLC analysis.

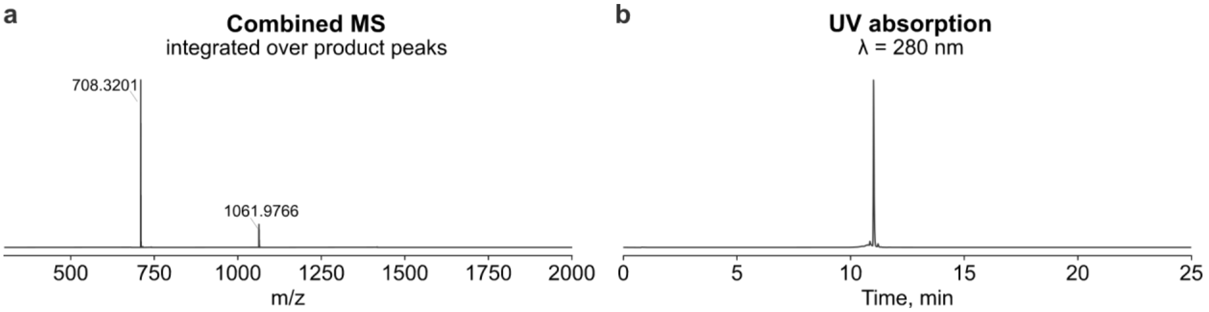

Figure S33. a) Mass spectra from LC/MS analysis of purified IR1 using method 1. ESI-MS (m/z): calcd for $C_{104}H_{127}N_{27}O_{19}S_2$ $[M+2H]^{2+}$ m/z = 1061.9695, found 1061.9766 ; $[M+3H]^{3+}$ m/z = 708.3154, found 708.3201. b) UV chromatogram of IR1 (acetate salt, λ = 280 nm) analyzed by the UPLC analysis method.

5.2.2 Synthesis of thiopeptide IR5

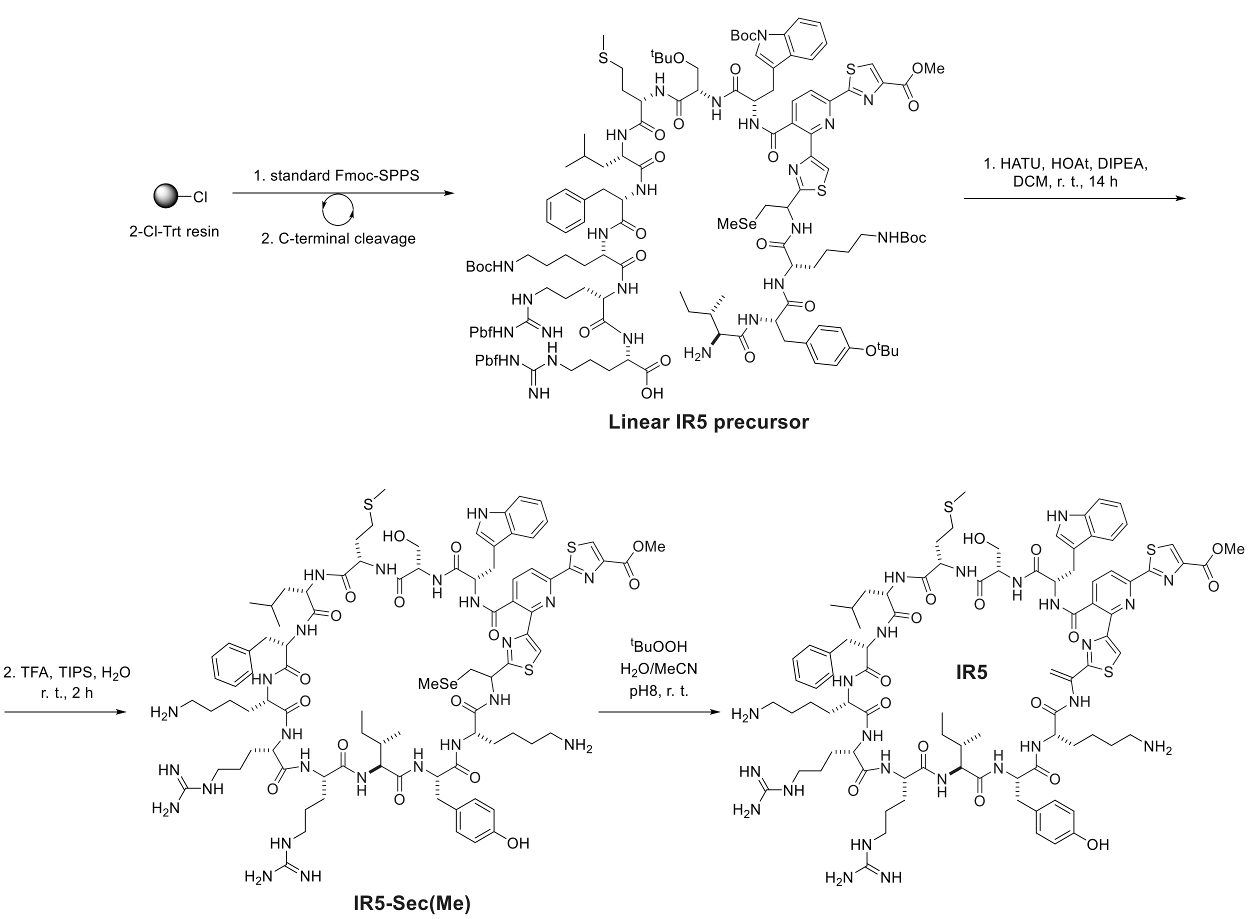

IR5 was synthesized according to general procedures as reported before.[20] The product was purified by RP-HPLC using HPLC method as described in general remarks 5.1 and lyophilized as a white solid (7.6 mg, 10% yield based on the resin loading), the purity was determined to be > 95% by analytical UPLC analysis.

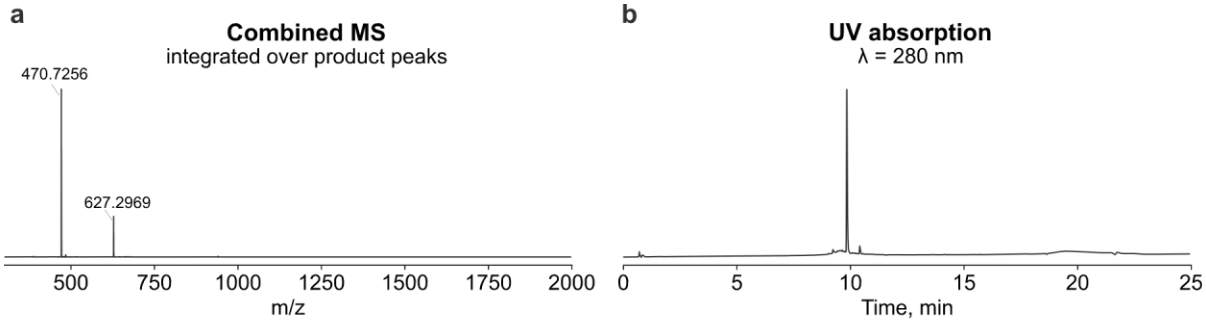

Figure S34. a) Mass spectra from LC/MS analysis of purified IR5 using method 1. ESI-MS (m/z): calcd for $C_{89}H_{122}N_{24}O_{16}S_3$ $[M+3H]^{3+}$ m/z = 627.2951, found 627.2969; $[M+4H]^{4+}$ m/z = 470.7231, found 470.7256. b) UV chromatogram of IR5 (acetate salt, λ = 280 nm) analyzed by the UPLC analysis method.

5.2.3 Synthesis of thiopeptide IR8

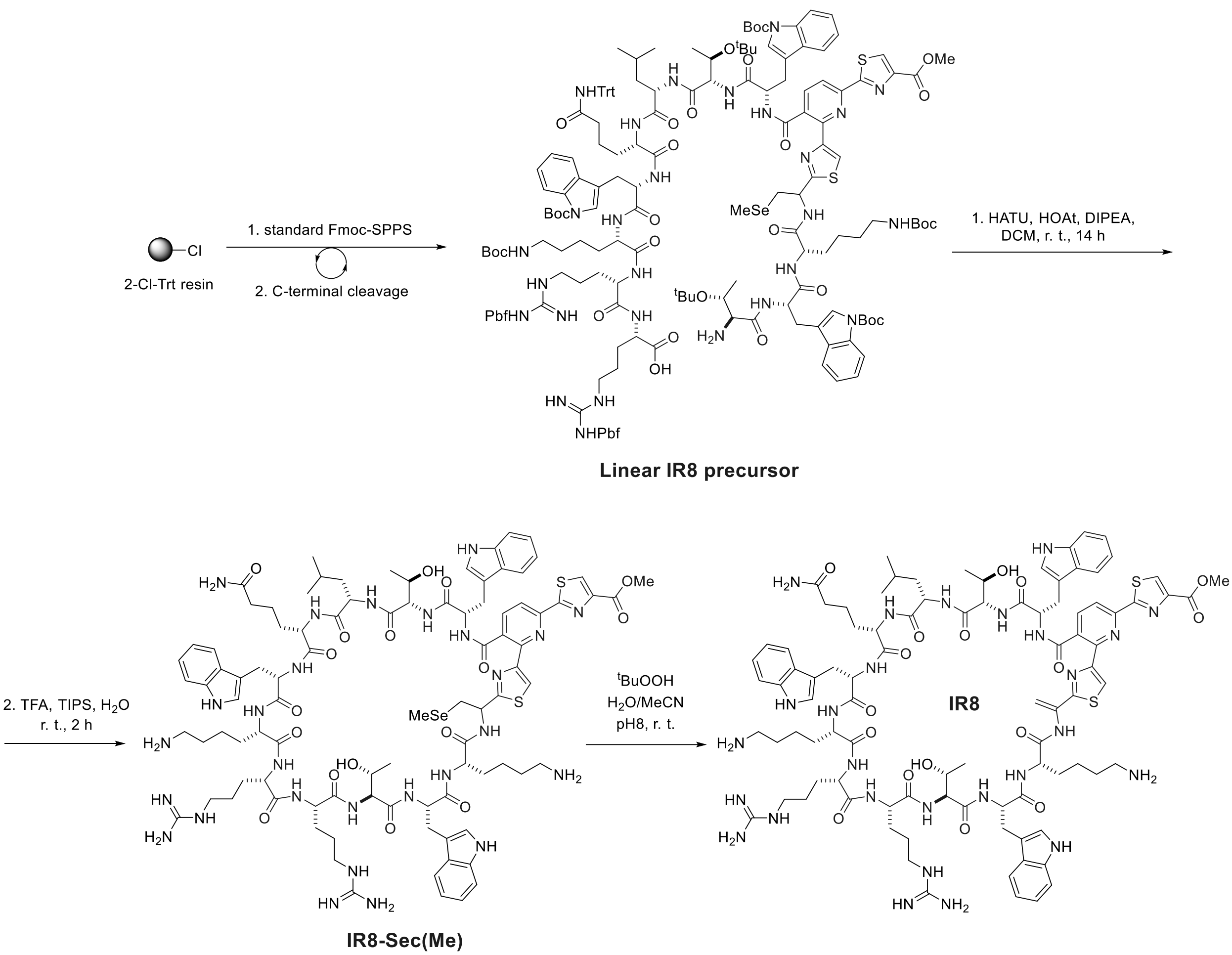

IR8 was synthesized according to general procedures as reported before.[20] The product was purified by RP-HPLC using HPLC method as described in general remarks 5.1 and lyophilized as a white solid (11.8 mg, 15% yield based on the resin loading), the purity was determined to be 99% by analytical UPLC analysis.

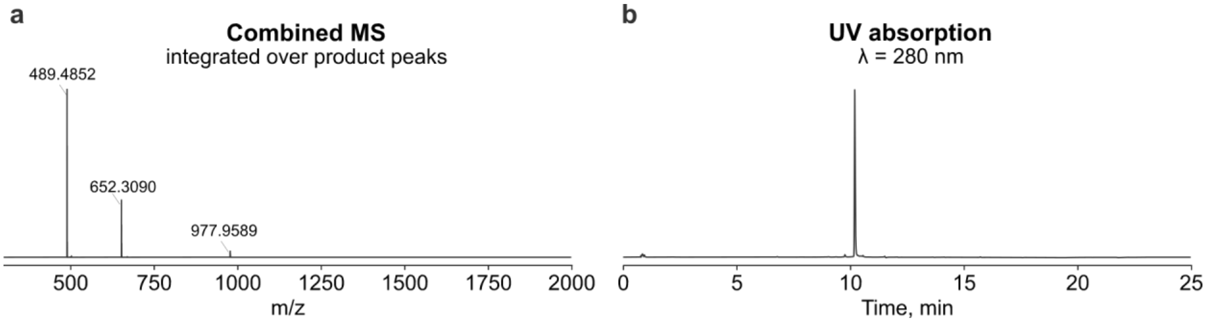

Figure S35. a) Mass spectra from LC/MS analysis of purified IR8 using method 1. ESI-MS (m/z): calcd for $C_{93}H_{123}N_{27}O_{17}S_2$ $[M+2H]^{2+}$ m/z = 977.9589, found 977.9589; $[M+3H]^{3+}$ m/z = 652.3084, found 652.3090; $[M+4H]^{4+}$ m/z = 489.4831, found 489.4852. b) UV chromatogram of IR8 (acetate salt, λ = 280 nm) analyzed by the UPLC analysis method.

5.2.4 Synthesis of thiopeptide IR11

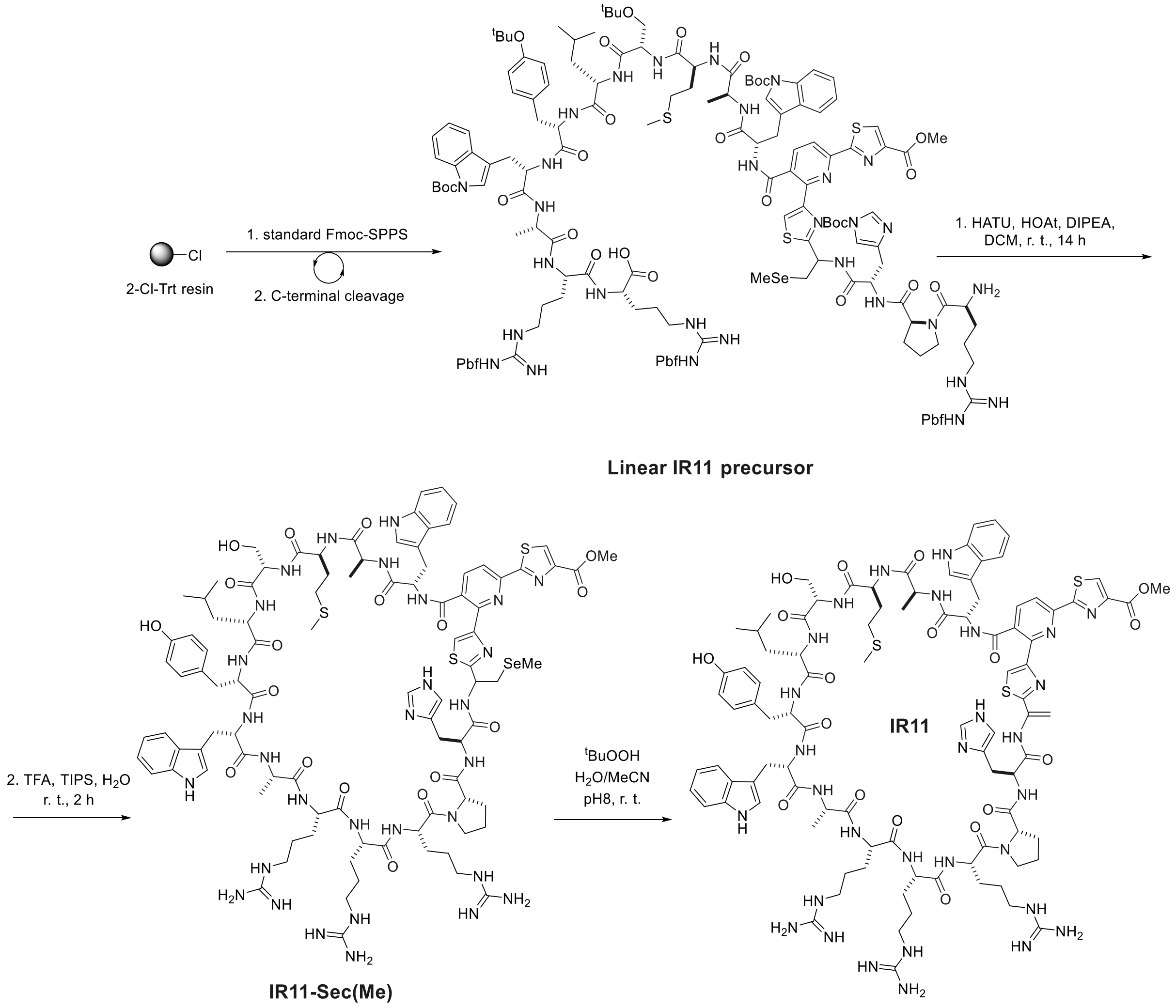

IR11 was synthesized according to general procedures as reported before.[20] The product was purified by RP-HPLC using HPLC method as described in general remarks 5.1 and lyophilized as a white solid (1 mg, 3% yield based on the resin loading), the purity was determined to be > 95% by analytical UPLC analysis.

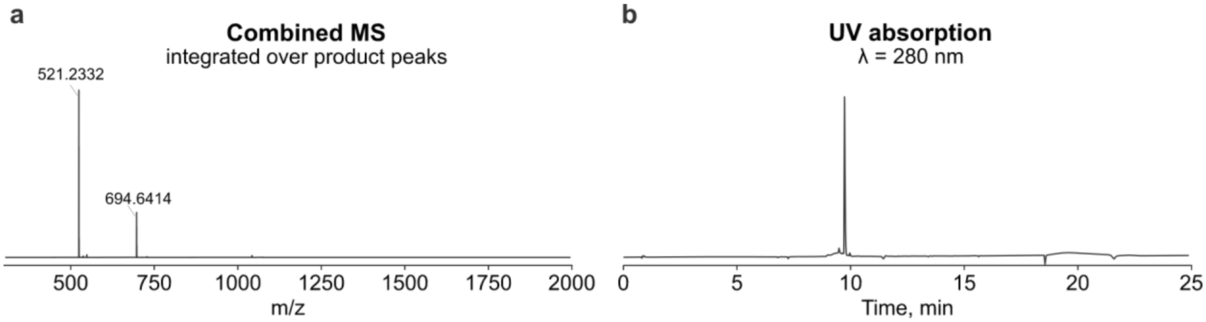

Figure S36. a) Mass spectra from LC/MS analysis of purified IR11 using method 1. ESI-MS (m/z): calcd for $C_{96}H_{124}N_{30}O_{18}S_3$ $[M+3H]^{3+}$ m/z = 694.6364, found 694.6414; $[M+4H]^{4+}$ m/z = 521.2219, found 521.2332. b) UV chromatogram of IR11 (acetate salt, λ = 280 nm) analyzed by the UPLC analysis method.

5.2.5 Synthesis of thiopeptide TL1

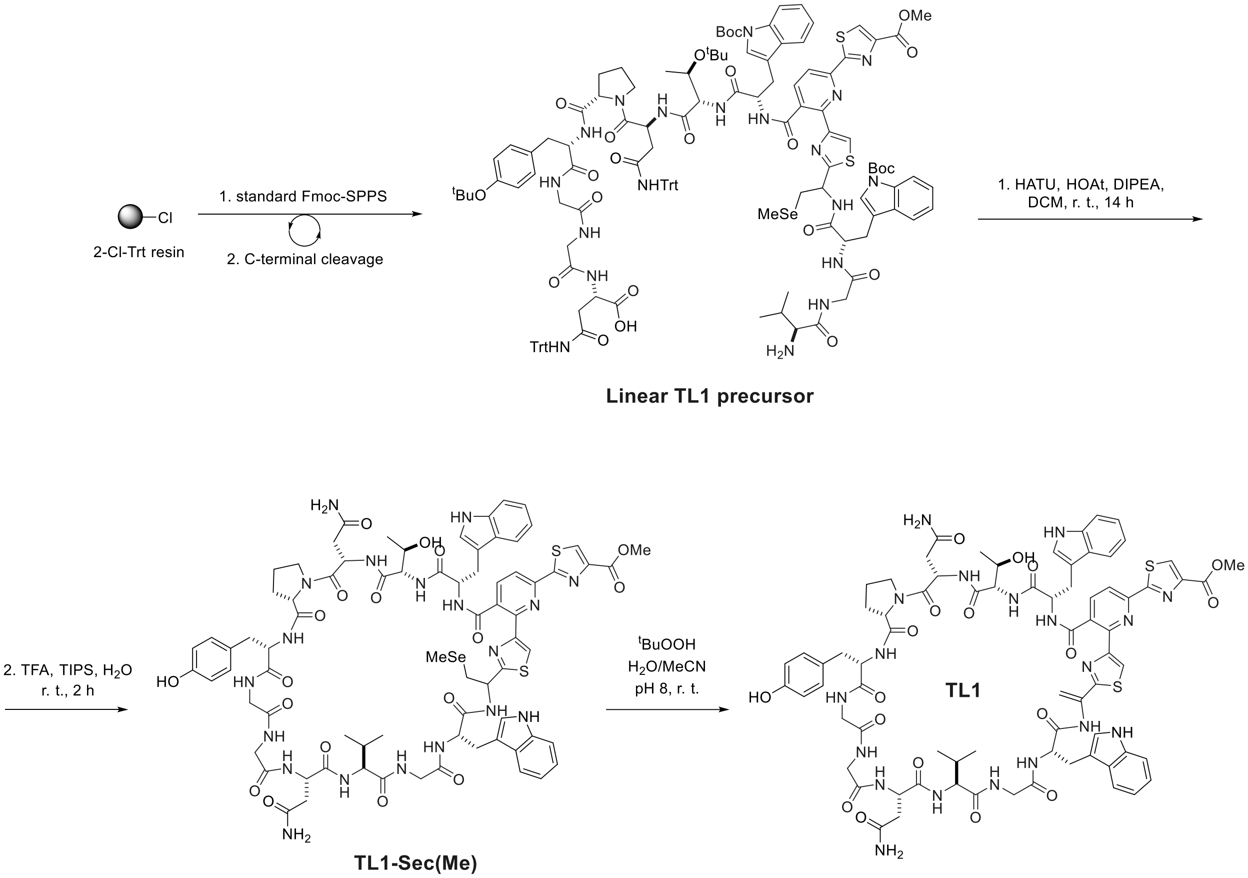

TL1 was synthesized according to general procedures as reported before.[20] The product was purified by RP-HPLC using HPLC method as described in general remarks 5.1 and lyophilized as a white solid (6.7 mg, 16% yield based on the resin loading), the purity was determined to be > 95% by analytical UPLC analysis.

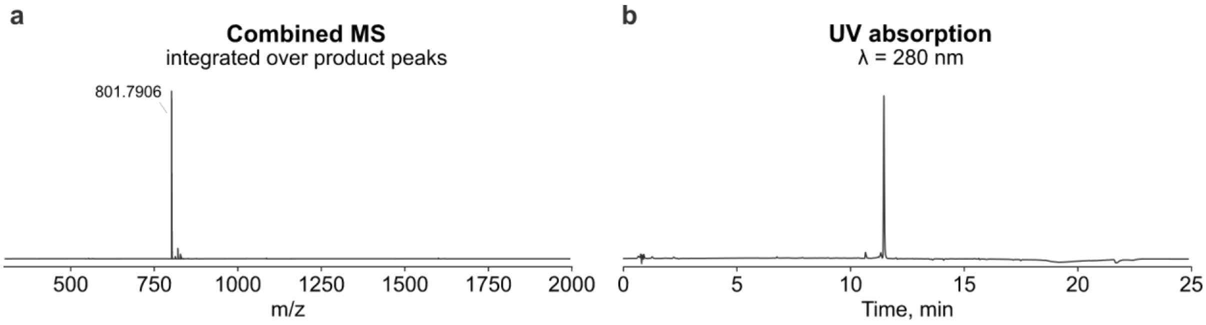

Figure S37. a) Mass spectra from LC/MS analysis of purified TL1 using method 1. ESI-MS (m/z): calcd for $C_{75}H_{83}N_{19}O_{18}S_2$ $[M+2H]^{2+}$ m/z = 801.7876, found 801.7906. b) UV chromatogram of TL1 (acetate salt, λ = 280 nm) analyzed by the UPLC analysis method.

5.2.6 Synthesis of thiopeptide TL3

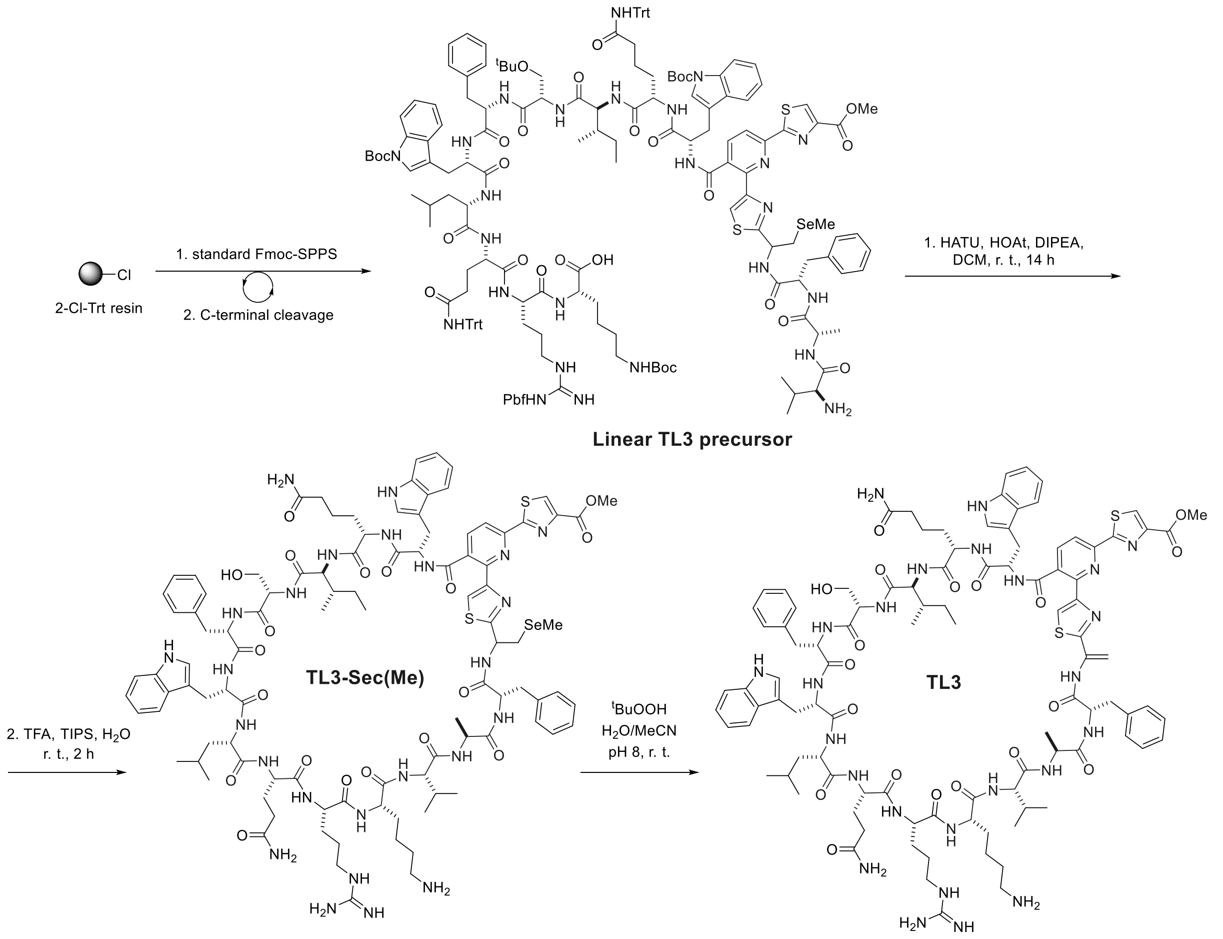

TL3 was synthesized according to general procedures as reported before.[20] The product was purified by RP-HPLC using HPLC method as described in general remarks 5.1 and lyophilized as a white solid (1.0 mg, 2% yield based on the resin loading), the purity was determined to be > 95% by analytical UPLC analysis.

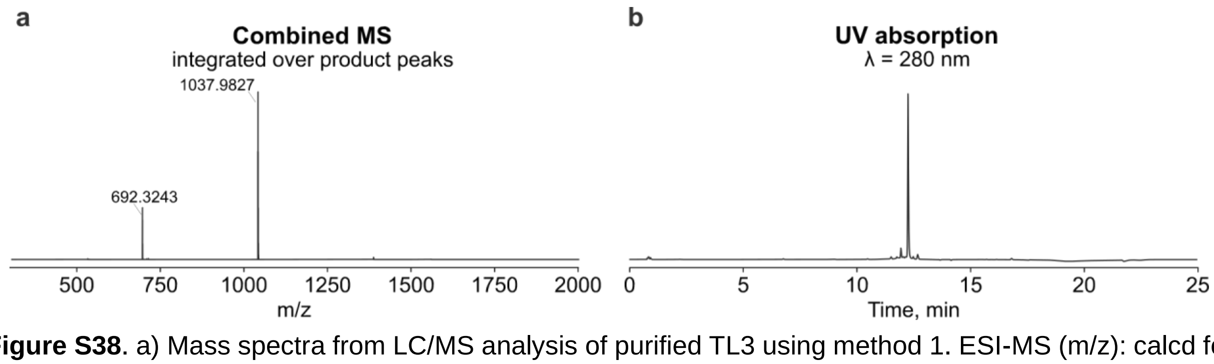

Figure S38. a) Mass spectra from LC/MS analysis of purified TL3 using method 1. ESI-MS (m/z): calcd for $C_{102}H_{131}N_{25}O_{19}S_2$ $[M+2H]^{2+}$ m/z = 1037.9820, found 1037.9827; $[M+3H]^{3+}$ m/z = 692.3238, found 692.3243. b) UV chromatogram of TL3 (acetate salt, λ = 280 nm) analyzed by the UPLC analysis method.

5.2.7 Synthesis of thiopeptide TL4

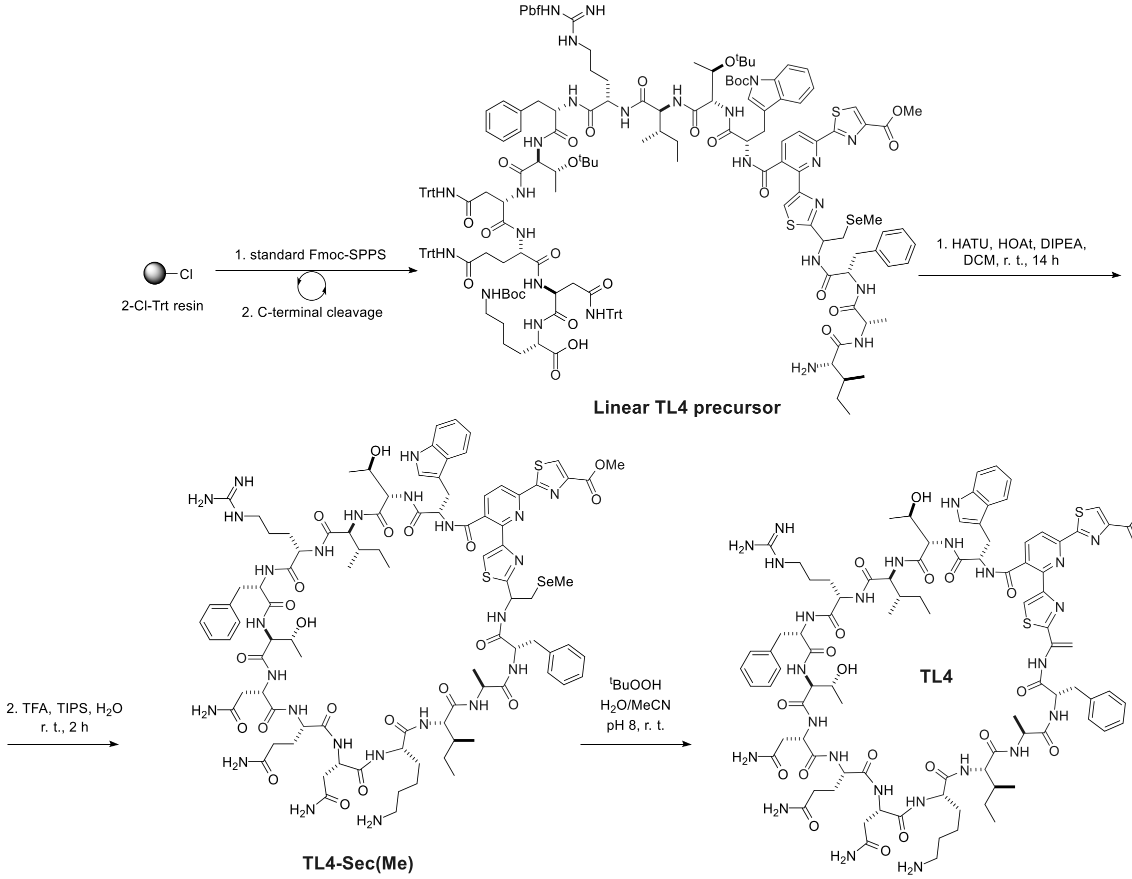

TL4 was synthesized according to general procedures as reported before.[20] The product was purified by RP-HPLC using HPLC method as described in general remarks 5.1 and lyophilized as a white solid (7.8 mg, 15% yield based on the resin loading), the purity was determined to be > 95% by analytical UPLC analysis.

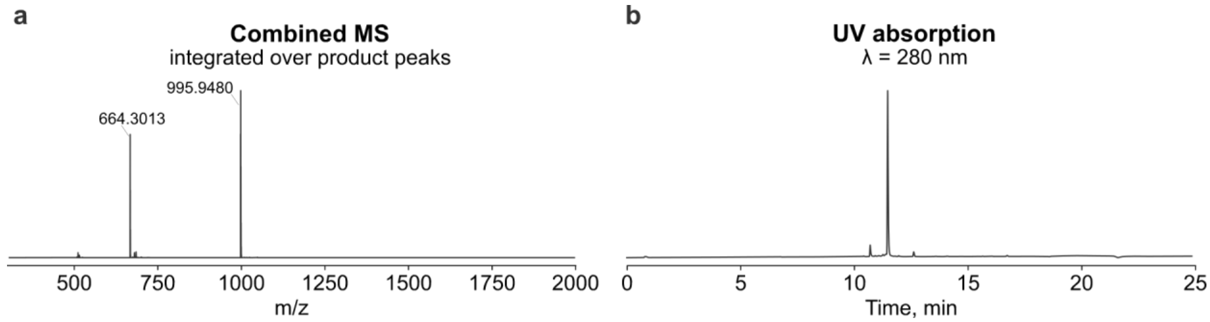

Figure S39. a) Mass spectra from LC/MS analysis of purified TL4 using method 1. ESI-MS (m/z): calcd for $C_{93}H_{123}N_{25}O_{21}S_2$ $[M+2H]^{2+}$ m/z = 995.9457, found 995.9480; $[M+3H]^{3+}$ m/z = 664.2995, found 664.3013. b) UV chromatogram of TL4 (acetate salt, λ = 280 nm) analyzed by the UPLC analysis method.

5.2.8 Synthesis of thiopeptide TL6

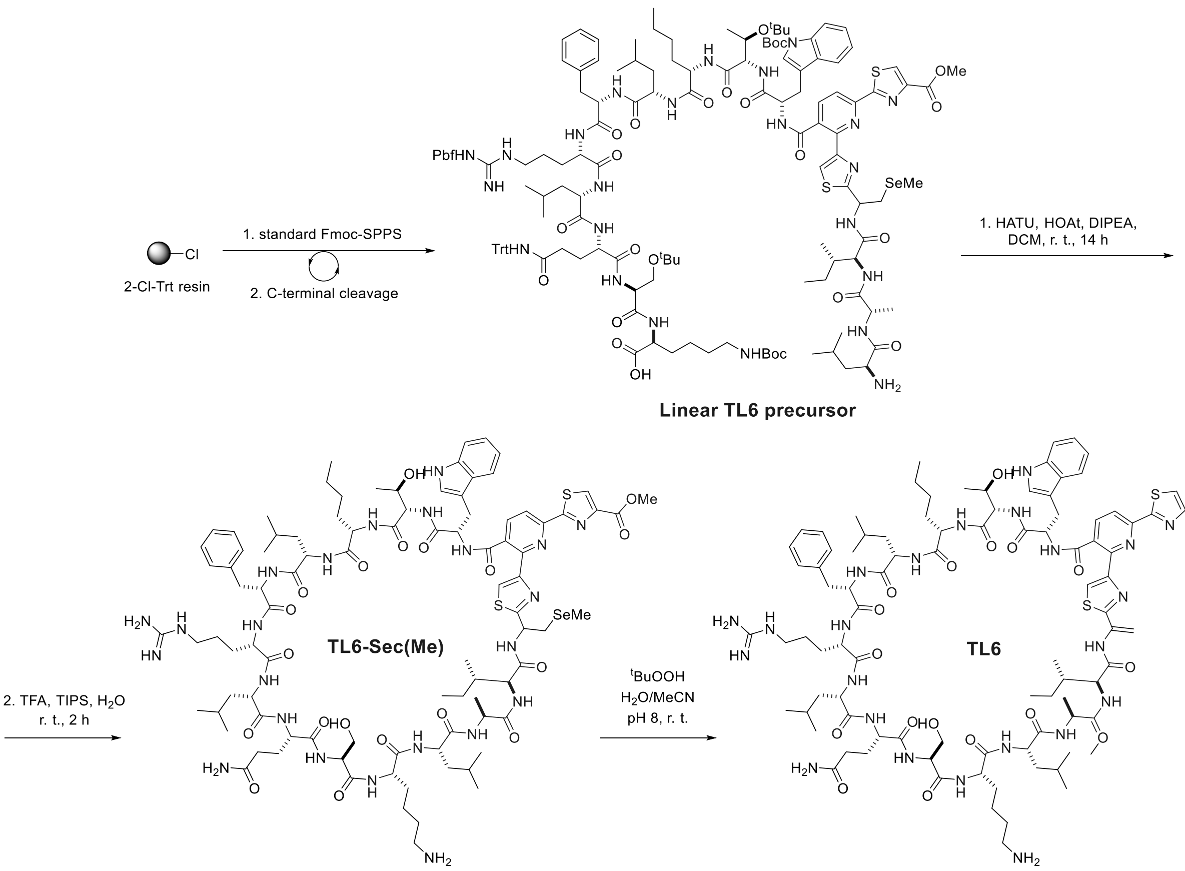

TL6 was synthesized according to general procedures as reported before.[20] The product was purified by RP-HPLC using HPLC method as described in general remarks 5.1 and lyophilized as a white solid (1.5 mg, 3% yield based on the resin loading), the purity was determined to be 98% by analytical UPLC analysis.

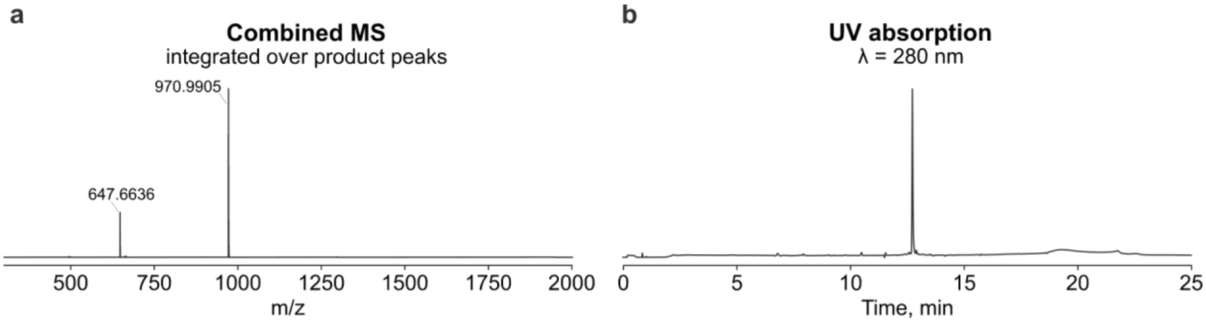

Figure S40. a) Mass spectra from LC/MS analysis of purified TL6 using method 1. ESI-MS (m/z): calcd for $C_{93}H_{133}N_{23}O_{19}S_2$ $[M+2H]^{2+}$ m/z = 970.9868, found 970.9905; $[M+3H]^{3+}$ m/z = 647.6603, found 647.6636. b) UV chromatogram of TL6 (acetate salt, λ = 280 nm) analyzed by the UPLC analysis method.

5.2.9 Synthesis of thiopeptide TL9

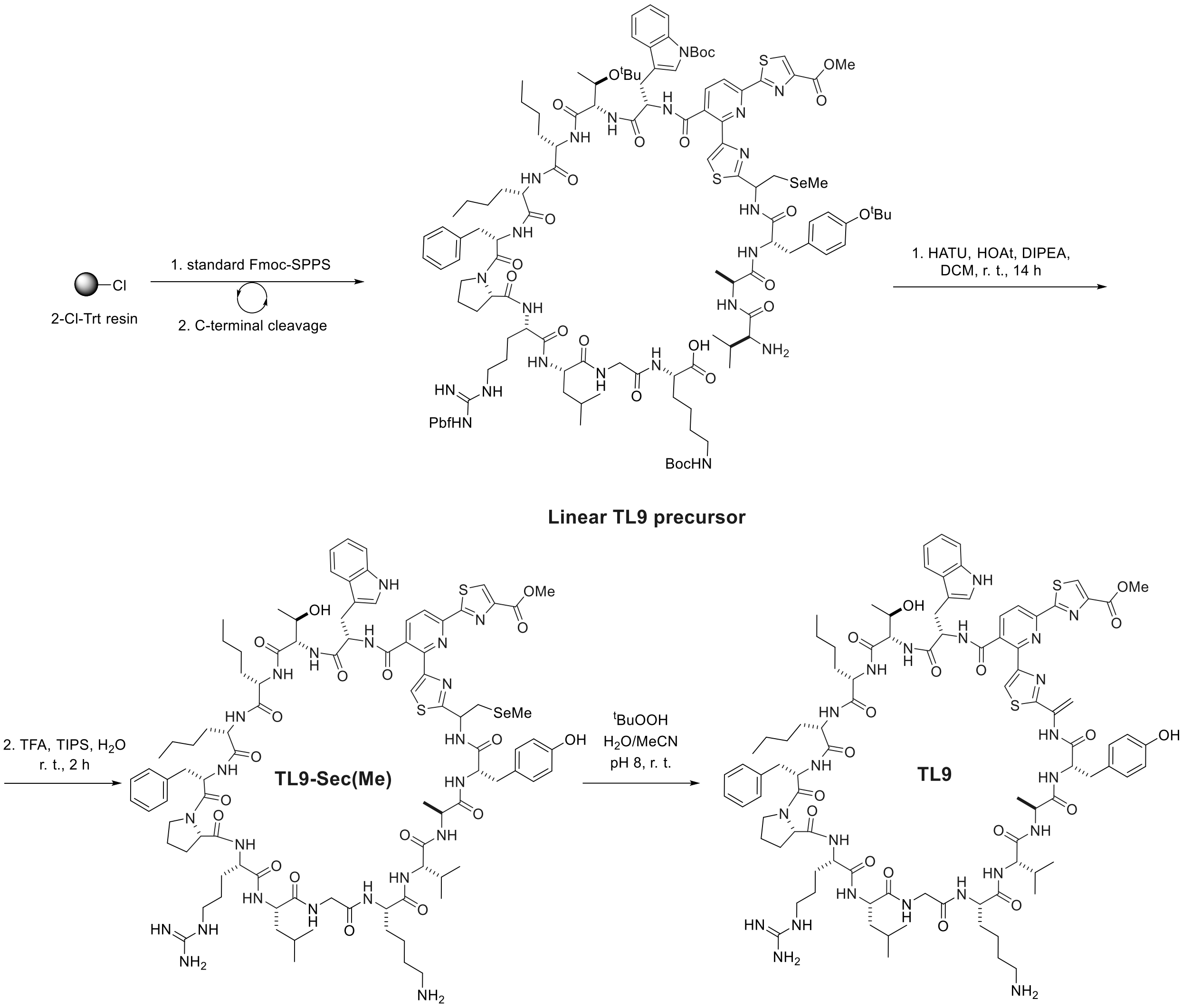

TL9 was synthesized according to general procedures as reported before.[20] The product was purified by RP-HPLC using HPLC method as described in general remarks 5.1 and lyophilized as a white solid (6.2 mg, 13% yield based on the resin loading), the purity was determined to be 99% by analytical UPLC analysis.

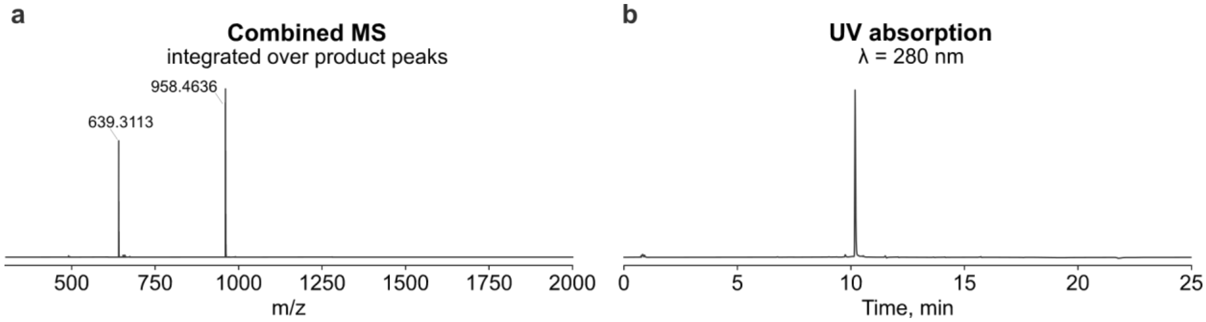

Figure S41. a) Mass spectra from LC/MS analysis of purified TL9 using method 1. ESI-MS (m/z): calcd for $C_{94}H_{126}N_{22}O_{18}S_2$ $[M+2H]^{2+}$ m/z = 958.4604, found 958.4636; $[M+3H]^{3+}$ m/z = 639.3094, found 639.3113. b) UV chromatogram of TL9 (acetate salt, λ = 280 nm) was analyzed by the UPLC analysis method.

5.2.10 Synthesis of thiopeptide TL10

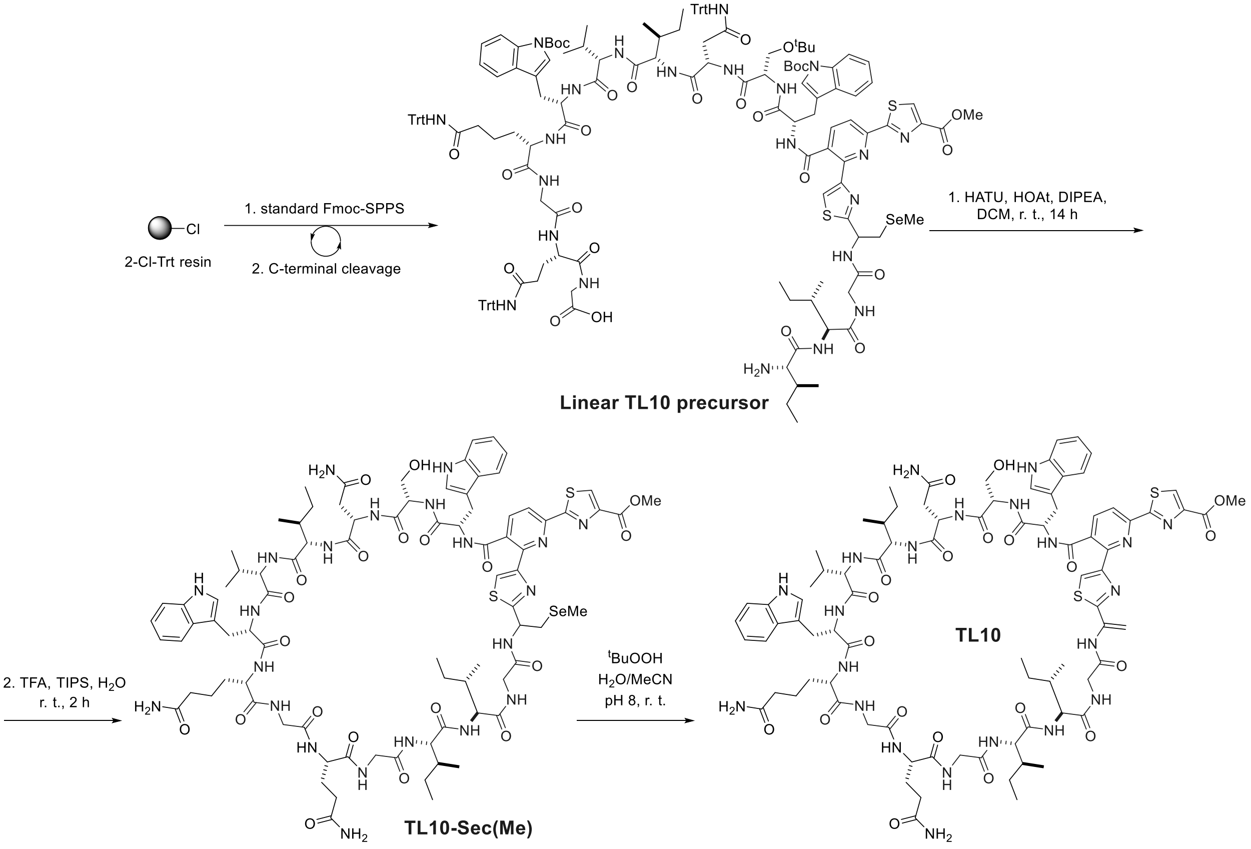

TL10 was synthesized according to general procedures as reported before.[20] The product was purified by RP-HPLC using HPLC method as described in general remarks 5.1 and lyophilized as a white solid (1 mg, 2% yield based on the resin loading), the purity was determined to be > 95% by analytical UPLC analysis.

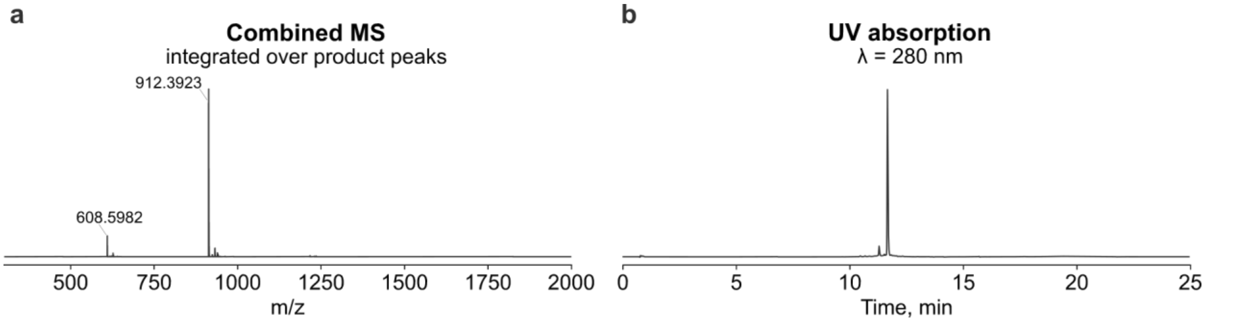

Figure S42. a) Mass spectra from LC/MS analysis of purified TL10 using method 1. ESI-MS (m/z): calcd for $C_{85}H_{110}N_{22}O_{20}S_2$ $[M+2H]^{2+}$ m/z = 912.3927, found 912.3923; $[M+3H]^{3+}$ m/z = 608.5976, found 608.5982. b) UV chromatogram of TL10 (acetate salt, λ = 280 nm) analyzed by the UPLC analysis method.

5.2.11 Synthesis of thiopeptide TL17

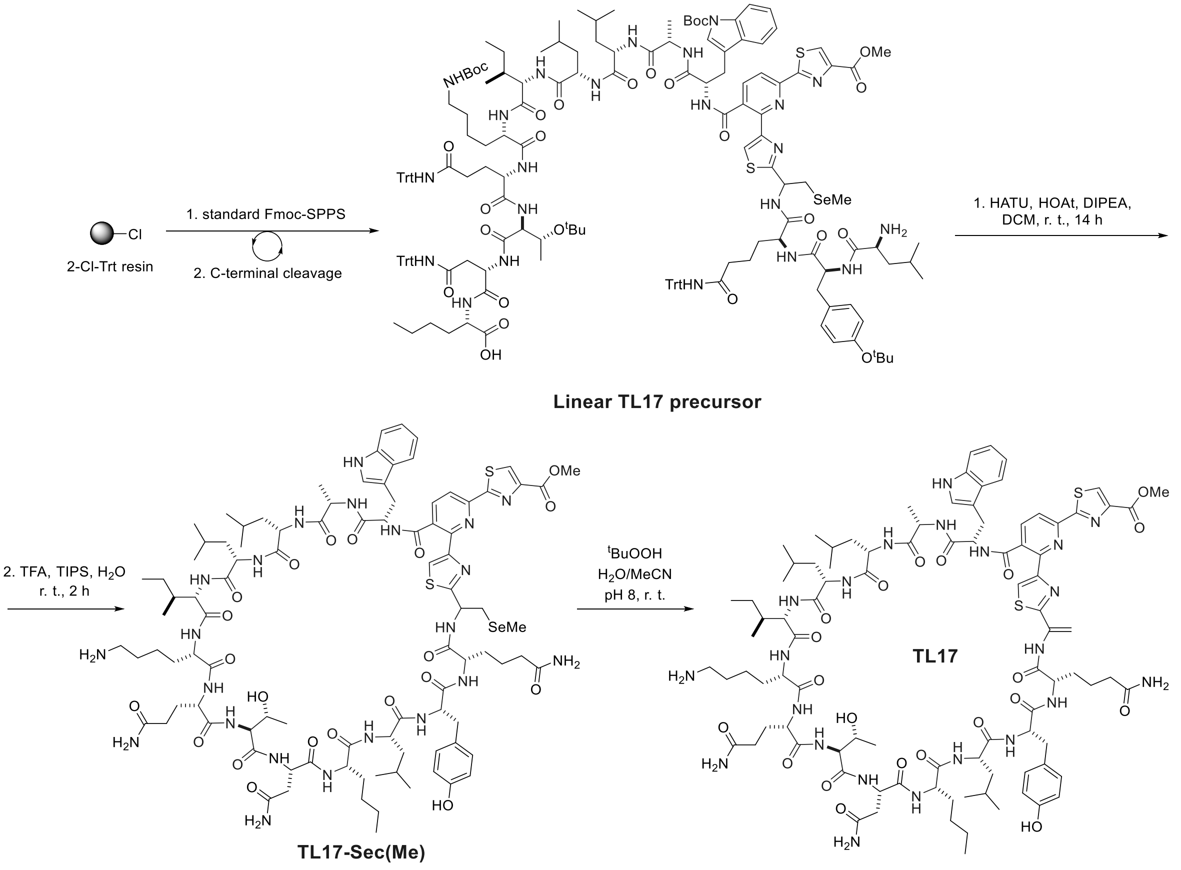

TL17 was synthesized according to general procedures as reported before.[20] The product was purified by RP-HPLC using HPLC method as described in general remarks 5.1 and lyophilized as a white solid (2.6 mg, 5% yield based on the resin loading), the purity was determined to be 99% by analytical UPLC analysis.

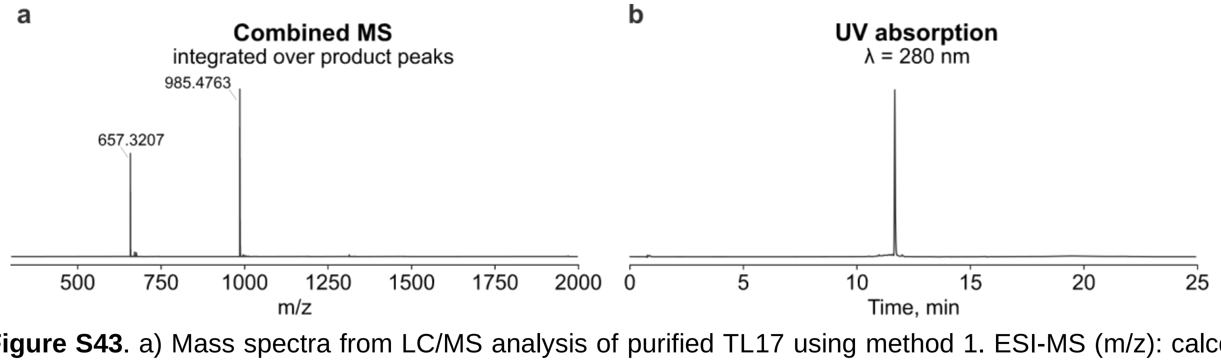

Figure S43. a) Mass spectra from LC/MS analysis of purified TL17 using method 1. ESI-MS (m/z): calcd for $C_{87}H_{122}N_{24}O_{21}S_2$ $[M+2H]^{2+}$ m/z = 985.4763, found 985.4795; $[M+3H]^{3+}$ m/z = 657.3199, found 657.3207. b) UV chromatogram of TL17 (acetate salt, λ = 280 nm) analyzed by the UPLC analysis method.

5.2.12 Synthesis of thiopeptide TL18

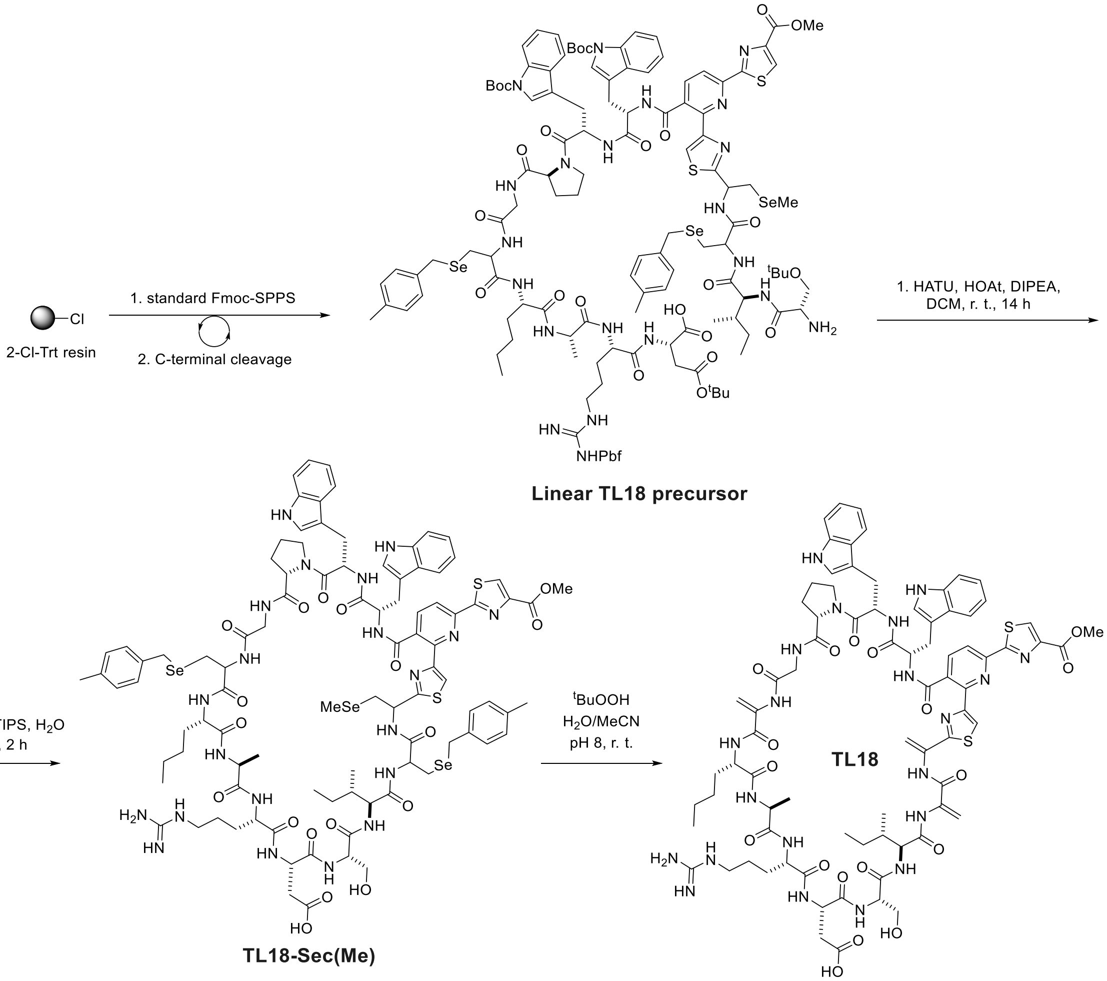

TL18 was synthesized according to general procedures as reported before.[20] The product was purified by RP-HPLC using HPLC method as described in general remarks 5.1 and lyophilized as a white solid (4.2 mg, 11% yield based on the resin loading), the purity was determined to be > 95% by analytical UPLC analysis.

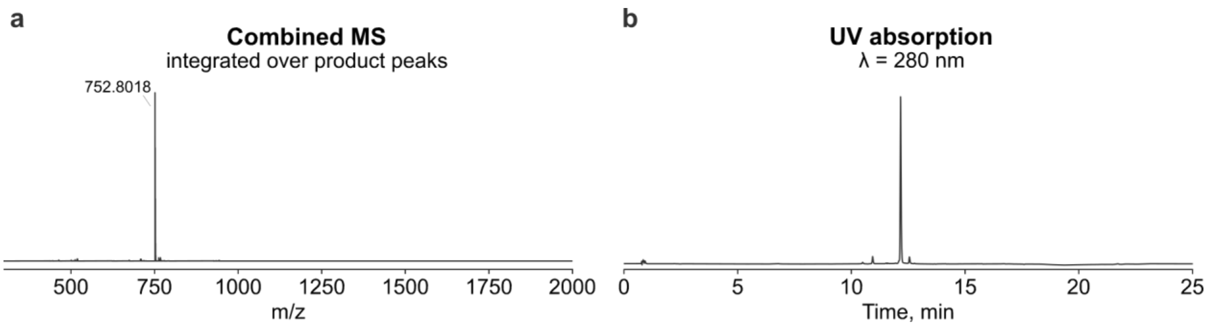

Figure S44. a) Mass spectra from LC/MS analysis of purified TL18 using method 1. ESI-MS (m/z): calcd for $C_{68}H_{85}N_{19}O_{17}S_2$ $[M+2H]^{2+}$ m/z = 752.7979, found 752.8018. b) UV chromatogram of TL18 (acetate salt, λ = 280 nm) analyzed by the UPLC analysis method.

5.2.13 Synthesis of thiopeptide TL19

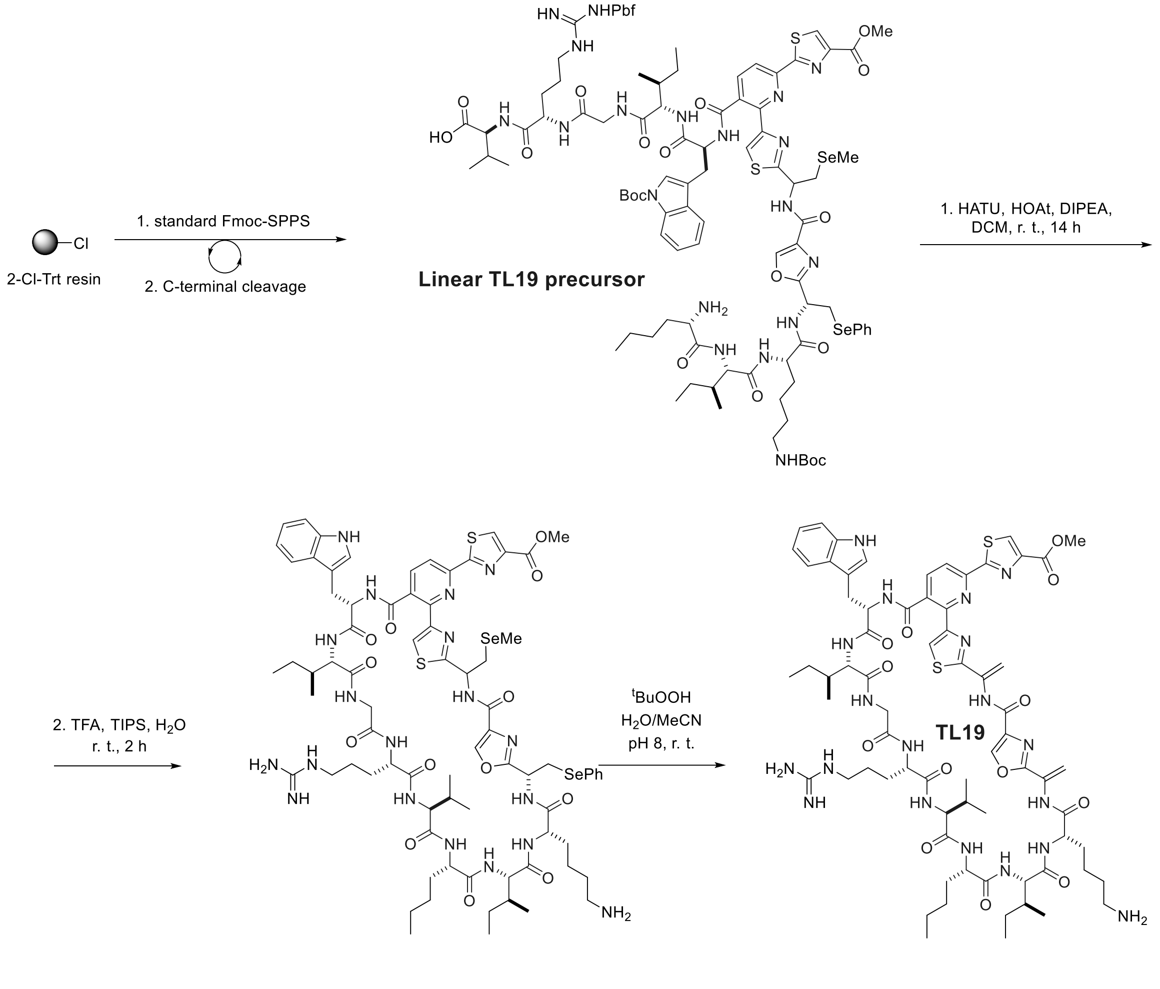

TL19-Sec(Me)

TL19 was synthesized according to general procedures as reported before.[20] The product was purified by RP-HPLC using HPLC method as described in general remarks 5.1 and lyophilized as a white solid (0.5 mg, 1% yield based on the resin loading), the purity was determined to be 99% by analytical UPLC analysis.

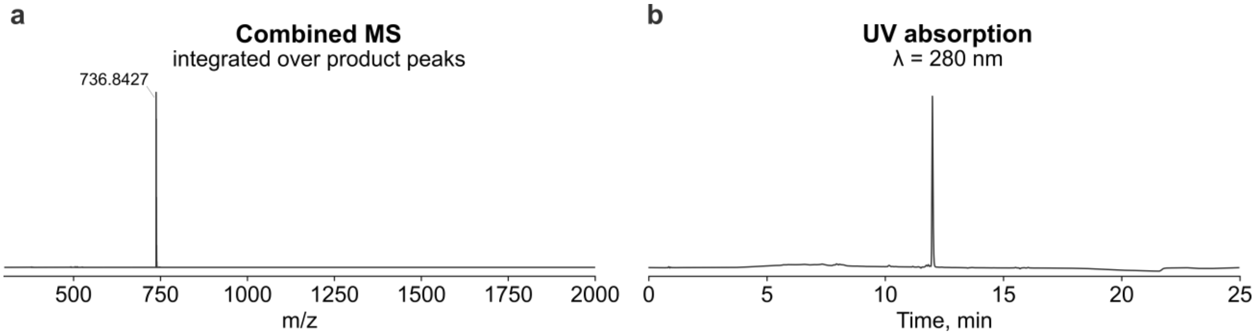

Figure S45. a) Mass spectra from LC/MS analysis of purified TL19 using method 1. ESI-MS (m/z): calcd for $C_{70}H_{93}N_{19}O_{13}S_2$ $[M+2H]^{2+}$ m/z = 736.8394, found 736.8427. b) UV chromatogram of TL19 (acetate salt, λ = 280 nm) analyzed by the UPLC analysis method.

5.2.14 Synthesis of thiopeptide TL20

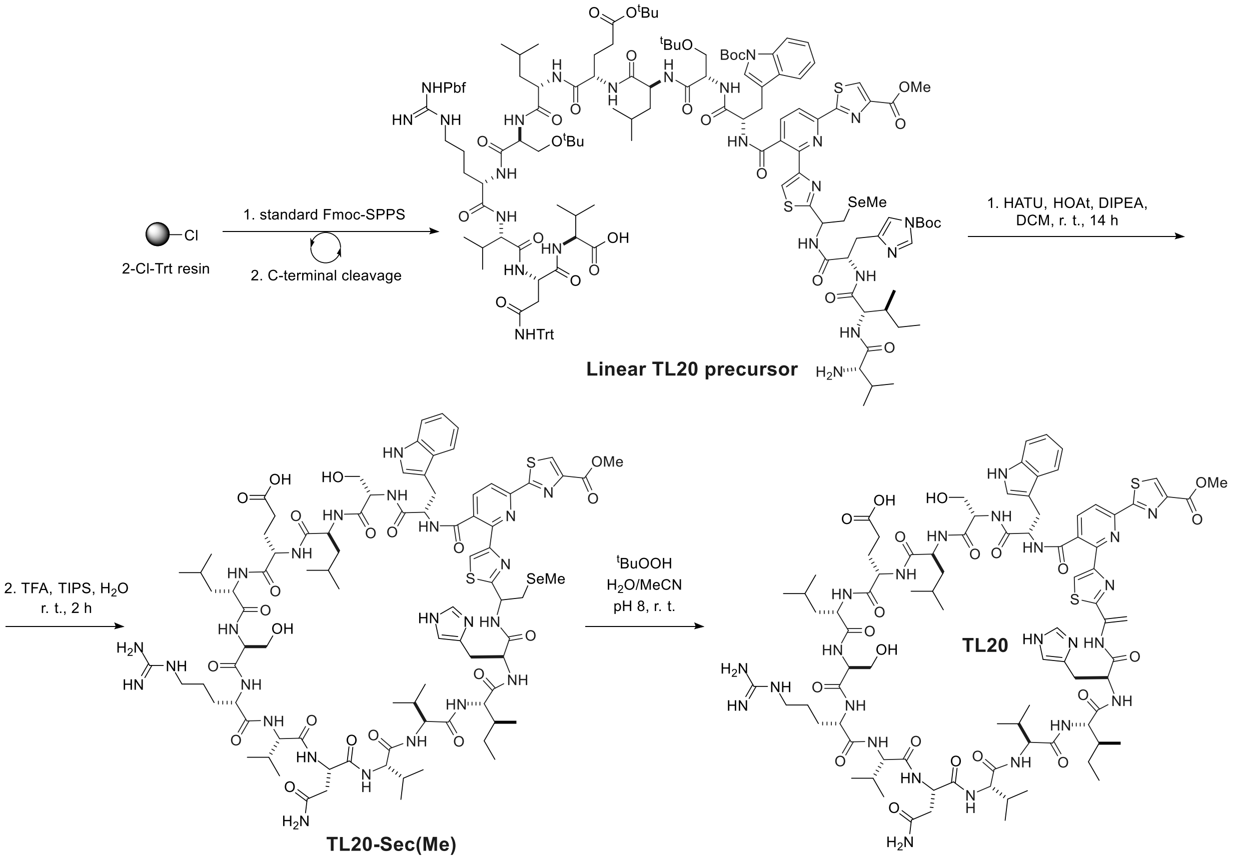

TL20 was synthesized according to general procedures as reported before.[20] The product was purified by RP-HPLC using HPLC method as described in general remarks 5.1 and lyophilized as a white solid (1.1 mg, 6% yield based on the resin loading), the purity was determined to be > 95% by analytical UPLC analysis.

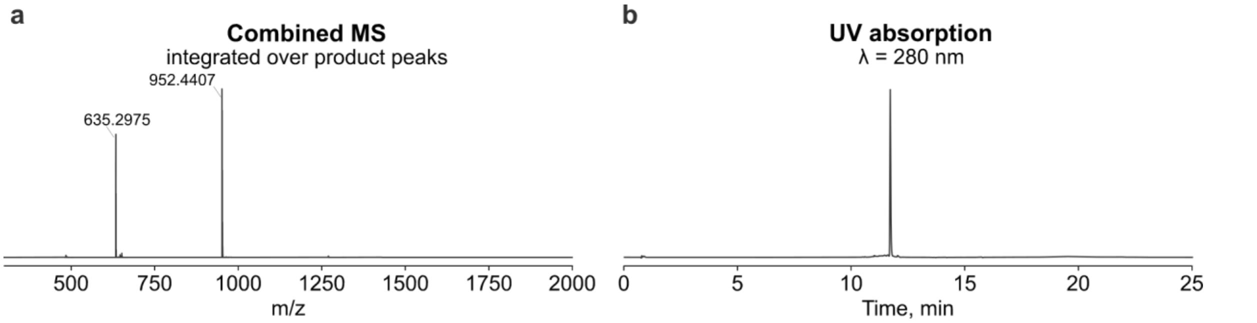

Figure S46. a) Mass spectra from LC/MS analysis of purified TL20 using method 1. ESI-MS (m/z): calcd for $C_{87}H_{122}N_{24}O_{21}S_2$ $[M+2H]^{2+}$ m/z = 952.4329, found 952.4407; $[M+3H]^{3+}$ m/z = 635.2886, found 635.2975. b) UV chromatogram of TL20 (acetate salt, λ = 280 nm) analyzed by the UPLC analysis method.

5.3. Late-stage Lactazole-like Thiopeptide Functionalization

5.3.1 Synthesis of IR1^{ct}

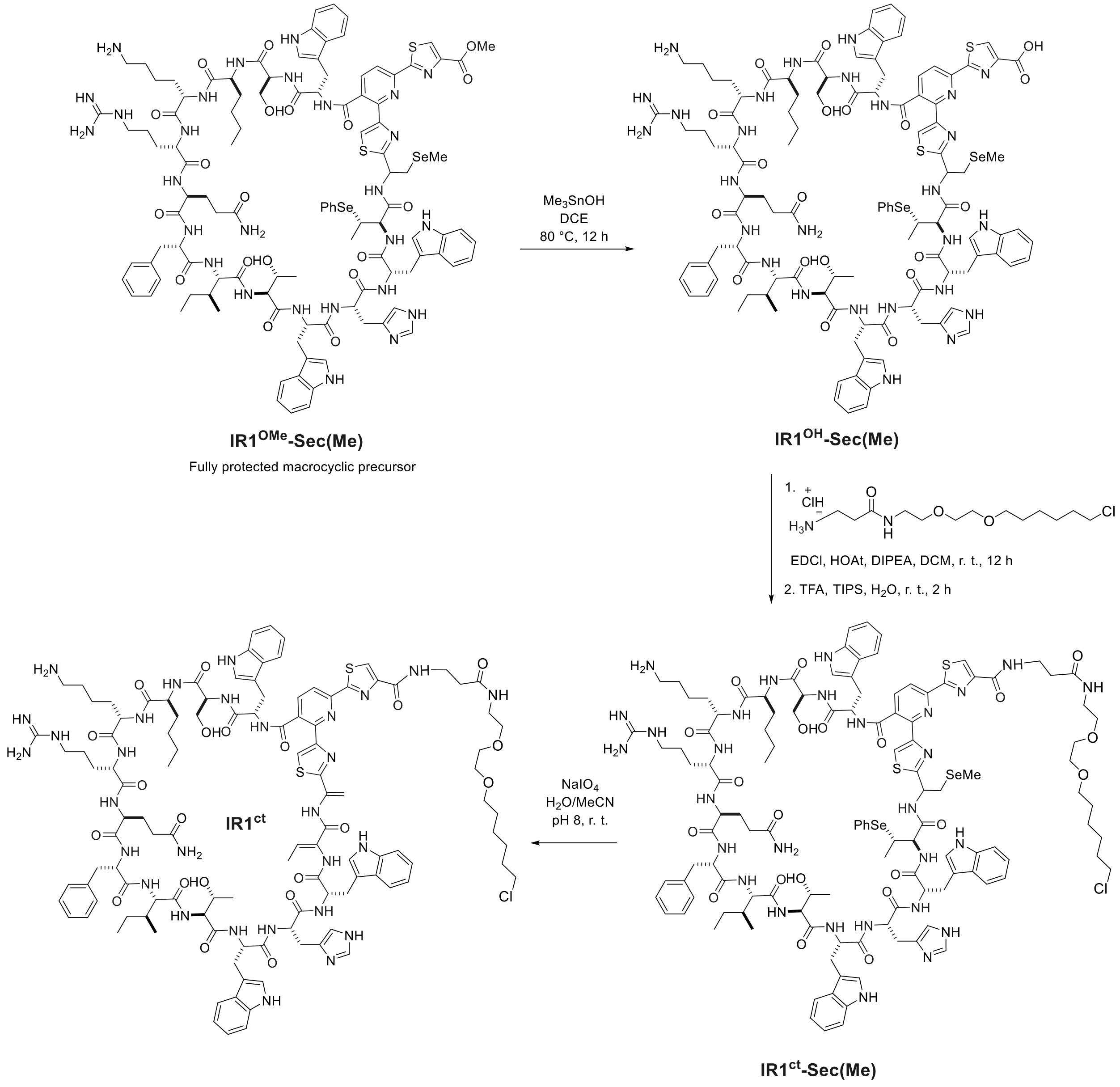

IR1^{ct} was synthesized according to general procedures as reported before.[20] The product was purified by RP-HPLC using HPLC method as described in general remarks 5.1 and lyophilized as a white solid (0.9 mg, 2% yield based on the resin loading), the purity was determined to be 96% by analytical UPLC analysis.

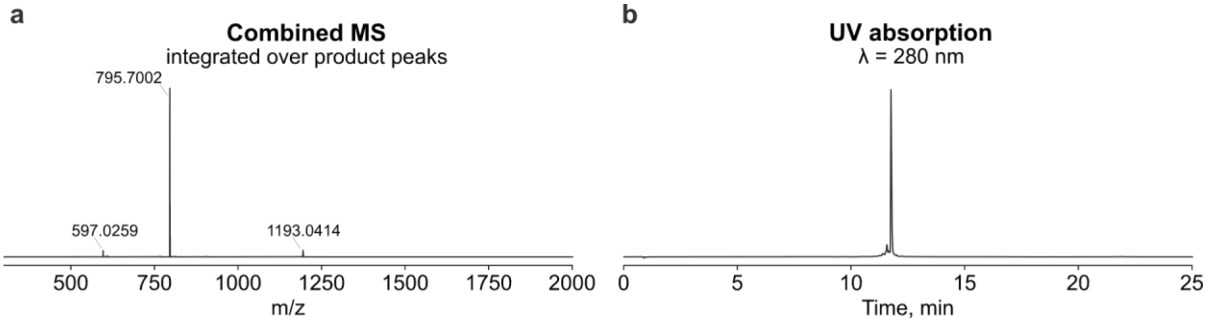

Figure S47. a) Mass spectra from LC/MS analysis of purified IR1^{ct} using method 1. ESI-MS (m/z): calcd for $C_{116}H_{150}ClN_{29}O_{21}S_2$ $[M+2H]^{2+}$ m/z = 1193.0419, found 1193.0414; $[M+3H]^{3+}$ m/z = 795.6970, found 795.7002; $[M+4H]^{4+}$ m/z = 597.0246, found 597.0259. b) UV chromatogram of IR1^{ct} (acetate salt, λ = 280 nm) analyzed by the UPLC analysis method.

5.3.2 Synthesis of IR5^{ct}

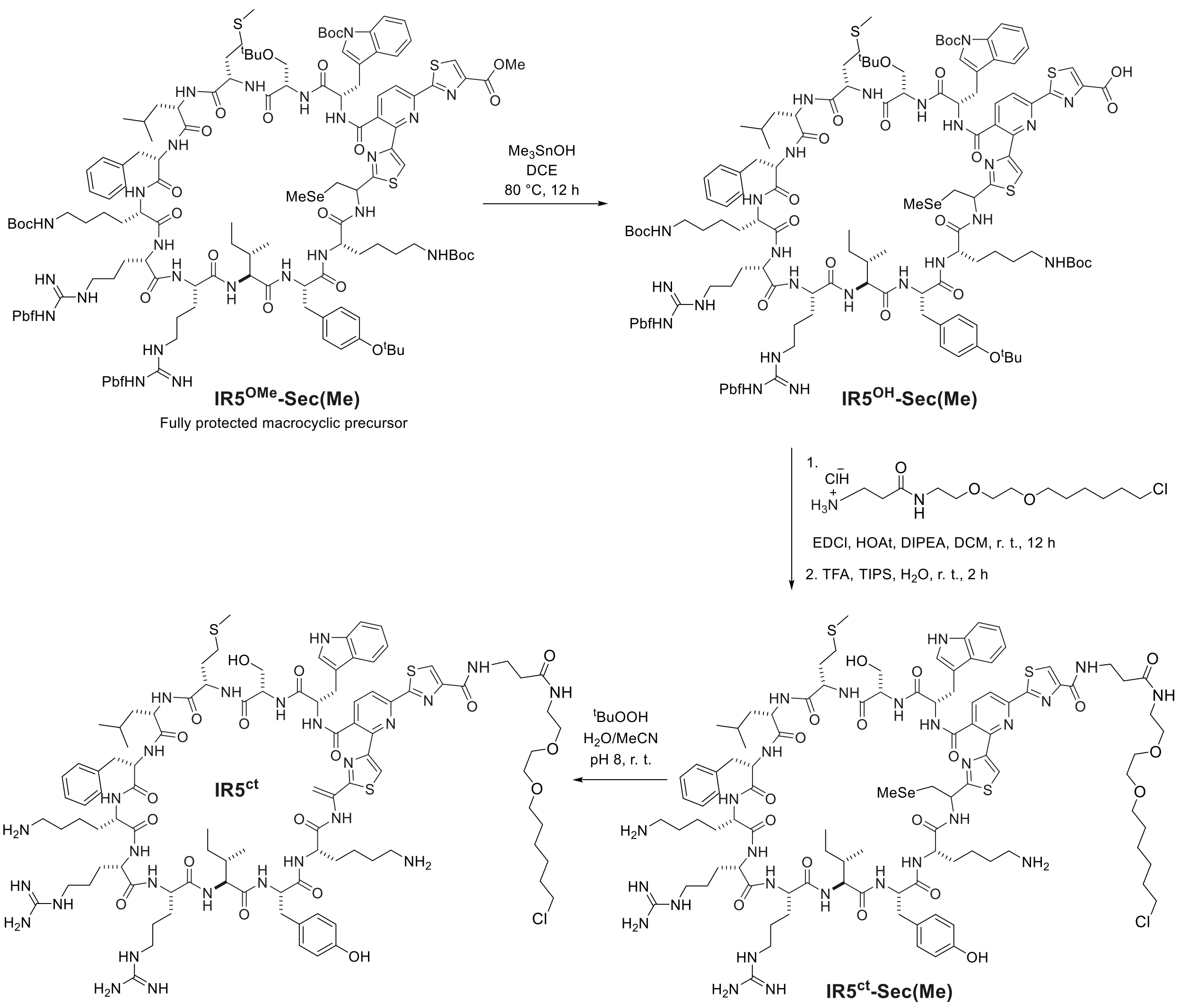

IR5^{ct} was synthesized according to general procedures as reported before.[20] The product was purified by RP-HPLC using HPLC method as described in general remarks 5.1 and lyophilized as a white solid (4.2 mg, 7% yield based on the resin loading), the purity was determined to be > 95% by analytical UPLC analysis.

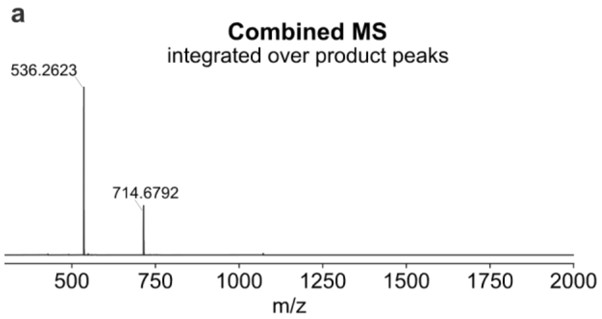

Figure S48. a) Mass spectra from LC/MS analysis of purified IR5^{ct} using method 1. ESI-MS (m/z): calcd for $C_{101}H_{145}ClN_{26}O_{18}S_3$ $[M+3H]^{3+}$ m/z = 714.6767, found 714.6792; $[M+4H]^{4+}$ m/z = 536.2593, found 536.2623. b) UV chromatogram of IR5^{ct} (acetate salt, λ = 280 nm) analyzed by the UPLC analysis method.

6. ^{1}H and ^{13}C NMR spectra

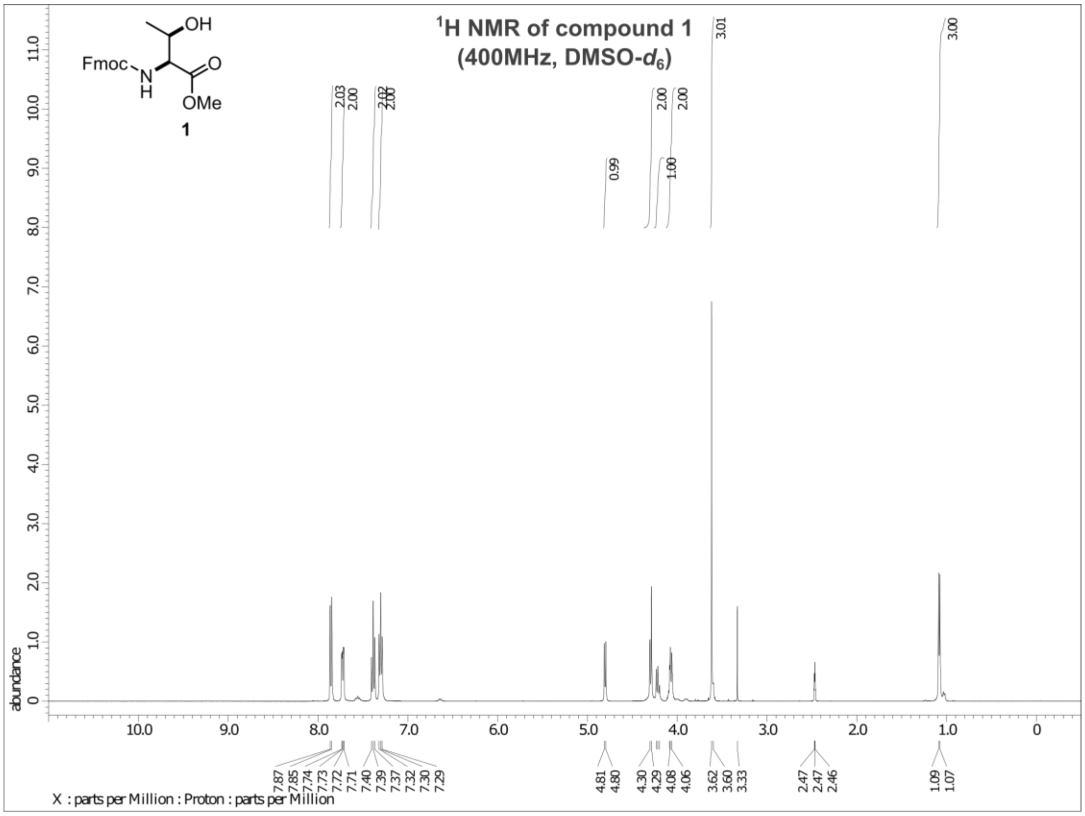

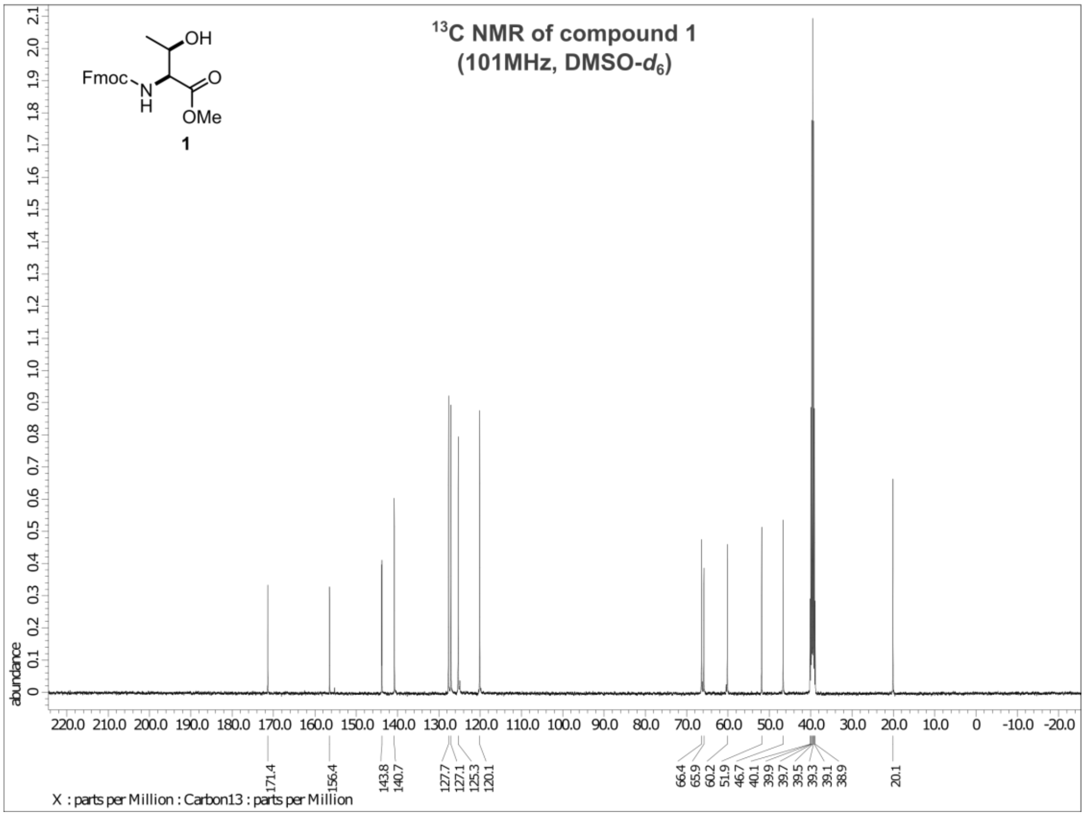

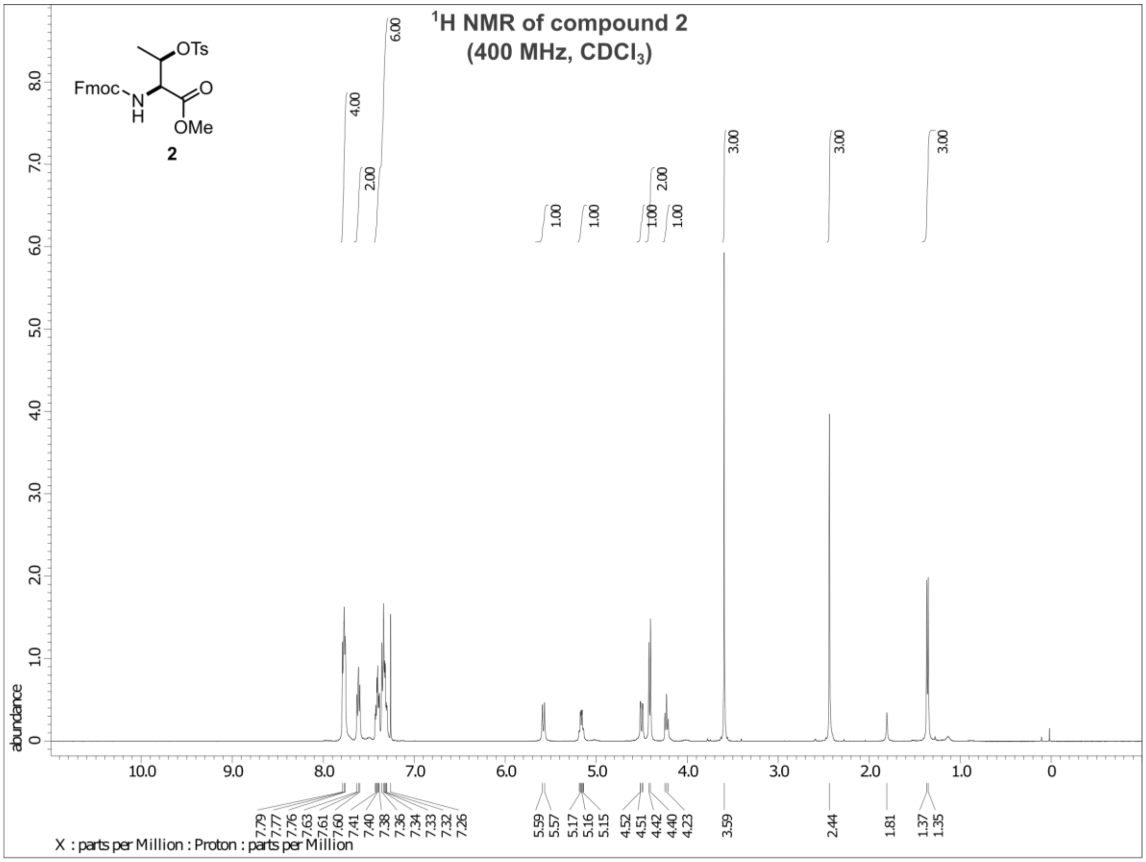

¹H NMR of compound 2
(400 MHz, CDCl₃)
OTs
Fmoc
N
H
O
OMe
2
abundance
X : parts per Million : Proton : parts per Million

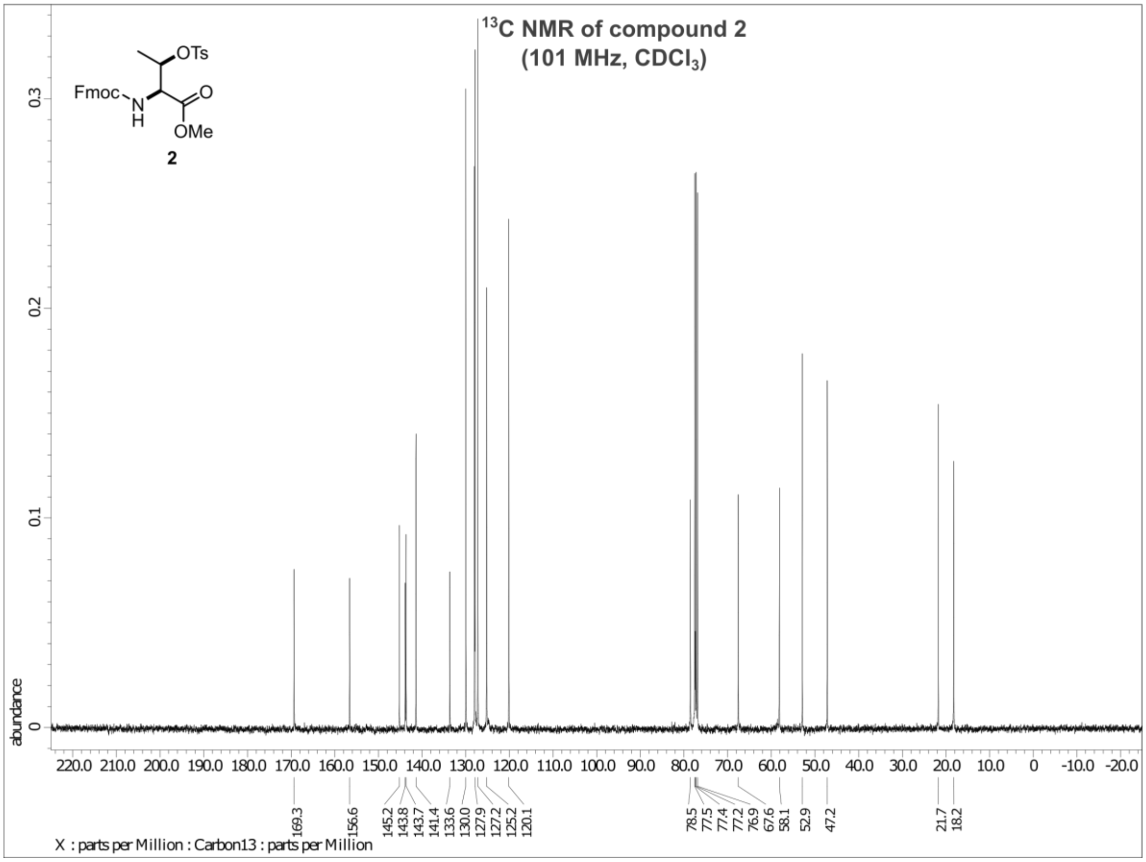

¹³C NMR of compound 2
(101 MHz, CDCl₃)
OTs
Fmoc
N
H
O
OMe
2
abundance
169.3
156.6
145.2
143.8
143.7
141.4
133.6
130.0
127.9
127.2
125.2
120.1
78.5
77.5
77.4
77.2
76.9
67.6
58.1
52.9
47.2
21.7
18.2
X : parts per Million : Carbon13 : parts per Million

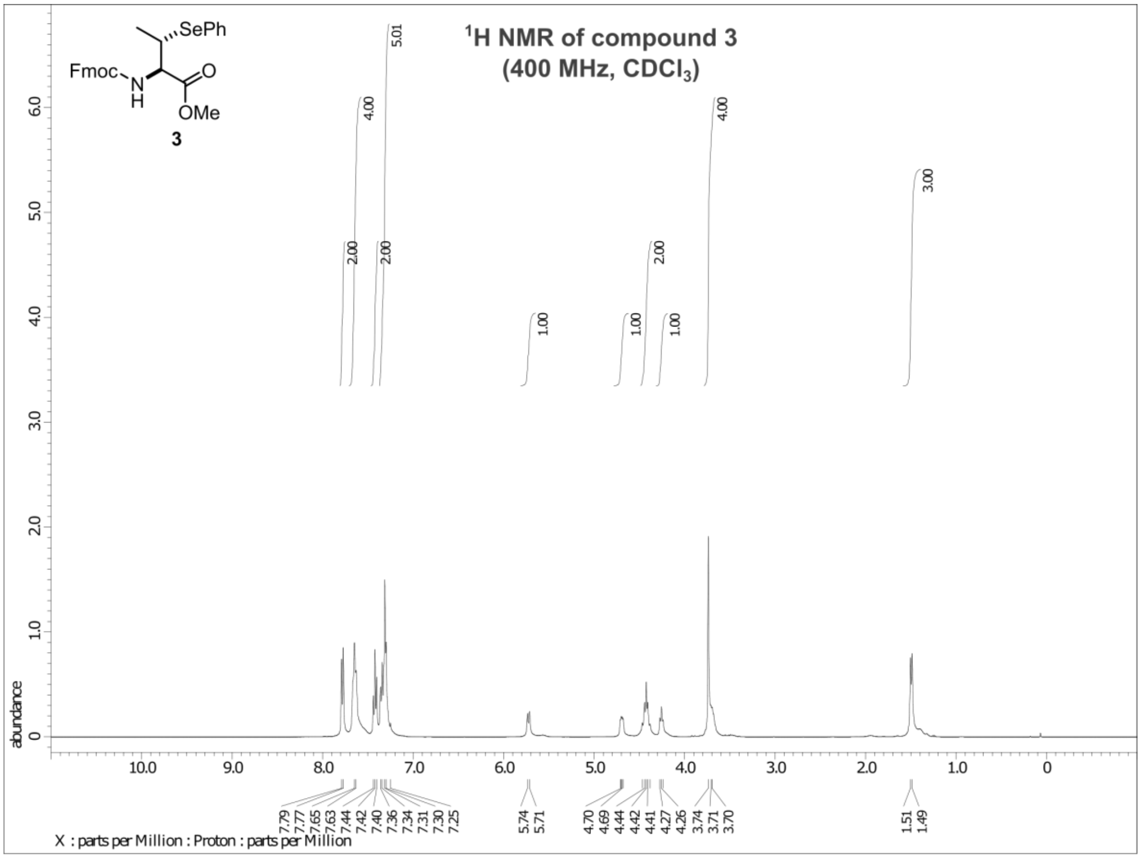
SePh
Fmoc
N
H
O
OMe
3
1H NMR of compound 3
(400 MHz, CDCl3)
abundance
X : parts per Million : Proton : parts per Million
7.79
7.77
7.65
7.63
7.44
7.42
7.40
7.36
7.34
7.31
7.30
7.25
5.74
5.71
4.70
4.69
4.44
4.42
4.41
4.27
4.26
3.74
3.71
3.70
1.51
1.49
2.00
4.00
2.00
5.01
1.00
1.00
2.00
1.00
4.00
3.00

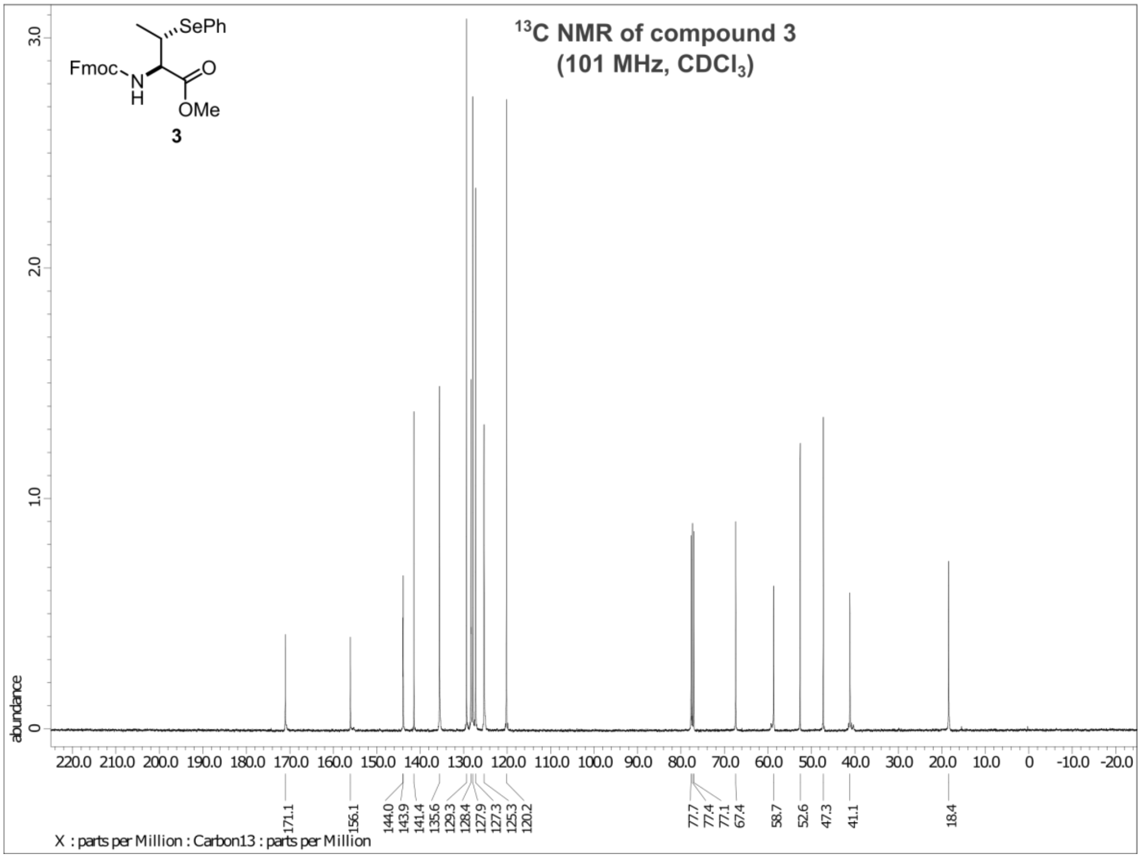
SePh
Fmoc
N
H
O
OMe
3
13C NMR of compound 3
(101 MHz, CDCl3)
abundance
X : parts per Million : Carbon13 : parts per Million
171.1
156.1
144.0
143.9
141.4
135.6
129.3
128.4
127.9
127.3
125.3
120.2
77.7
77.4
77.1
67.4
58.7
52.6
47.3
41.1
18.4

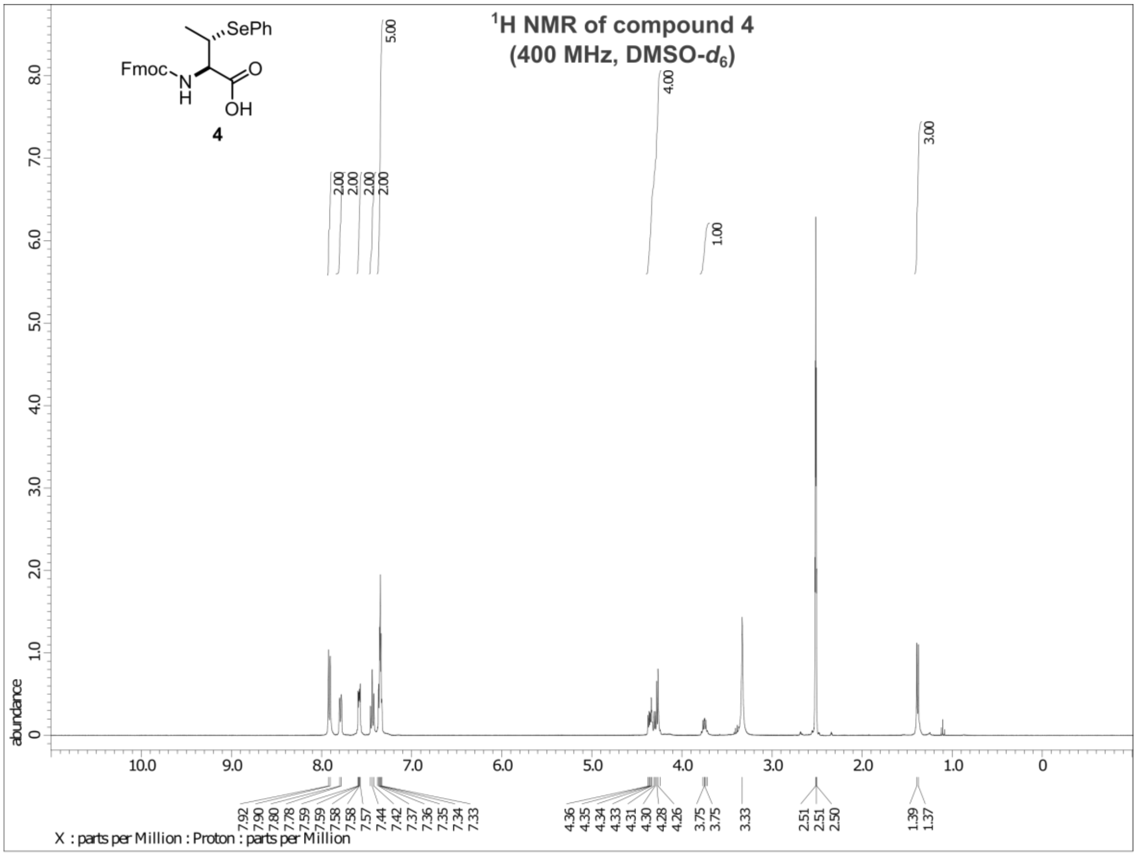

^{1}H NMR of compound 4
(400 MHz, DMSO-d_6)
SePh
Fmoc
N
H
O
OH
4
abundance
X : parts per Million : Proton : parts per Million

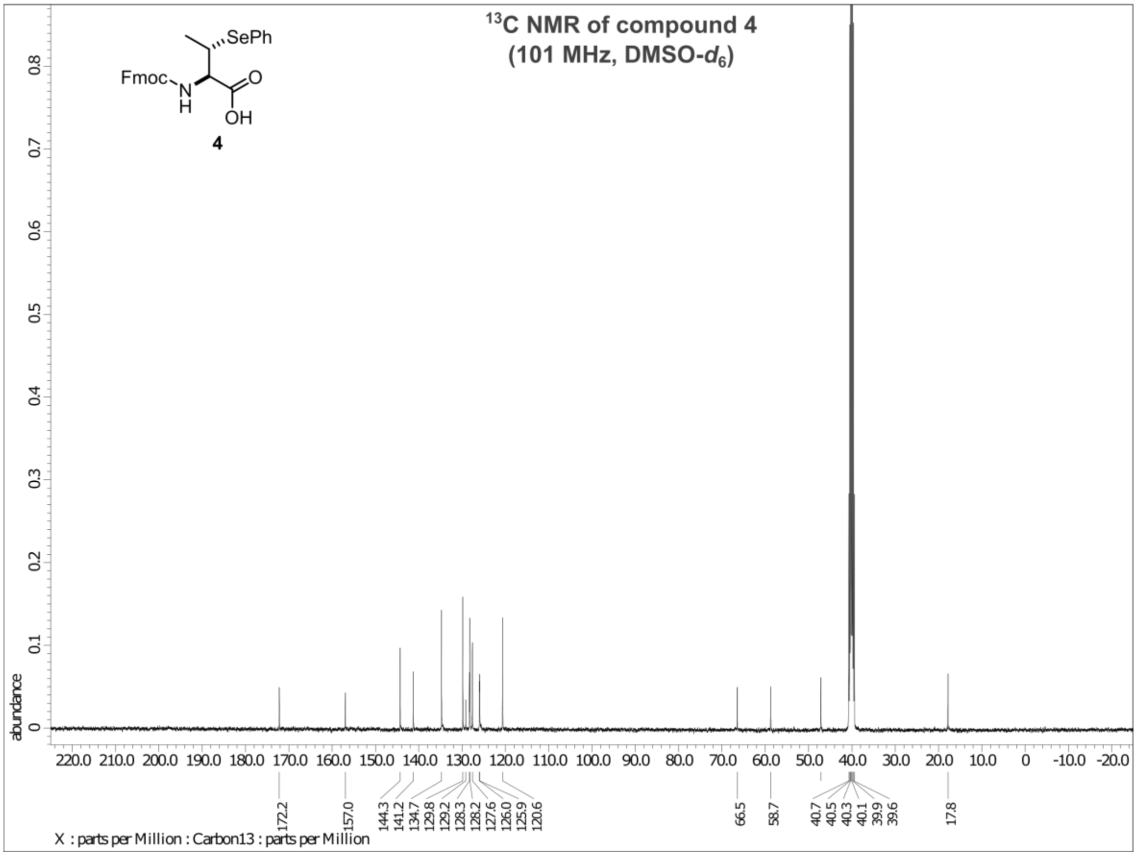

^{13}C NMR of compound 4
(101 MHz, DMSO-d_6)
SePh
Fmoc
N
H
O
OH
4
abundance
X : parts per Million : Carbon13 : parts per Million

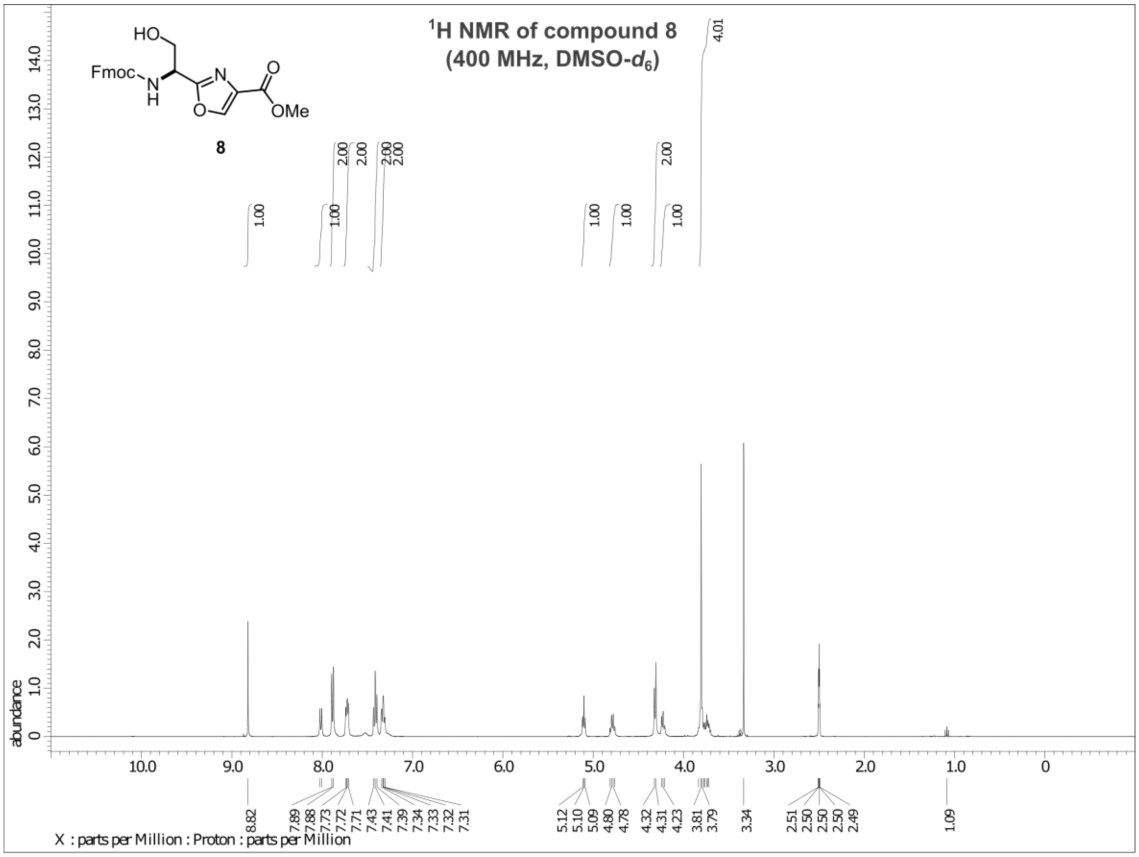

¹H NMR of compound 8
(400 MHz, DMSO-d6)
HO
Fmoc
N
H
N
O
O
OMe
8
abundance
X : parts per Million : Proton : parts per Million
8.82
7.89
7.88
7.73
7.72
7.71
7.43
7.41
7.39
7.34
7.33
7.32
7.31
5.12
5.10
5.09
4.80
4.78
4.32
4.31
4.23
3.81
3.79
3.34
2.51
2.50
2.50
2.50
2.49
1.09

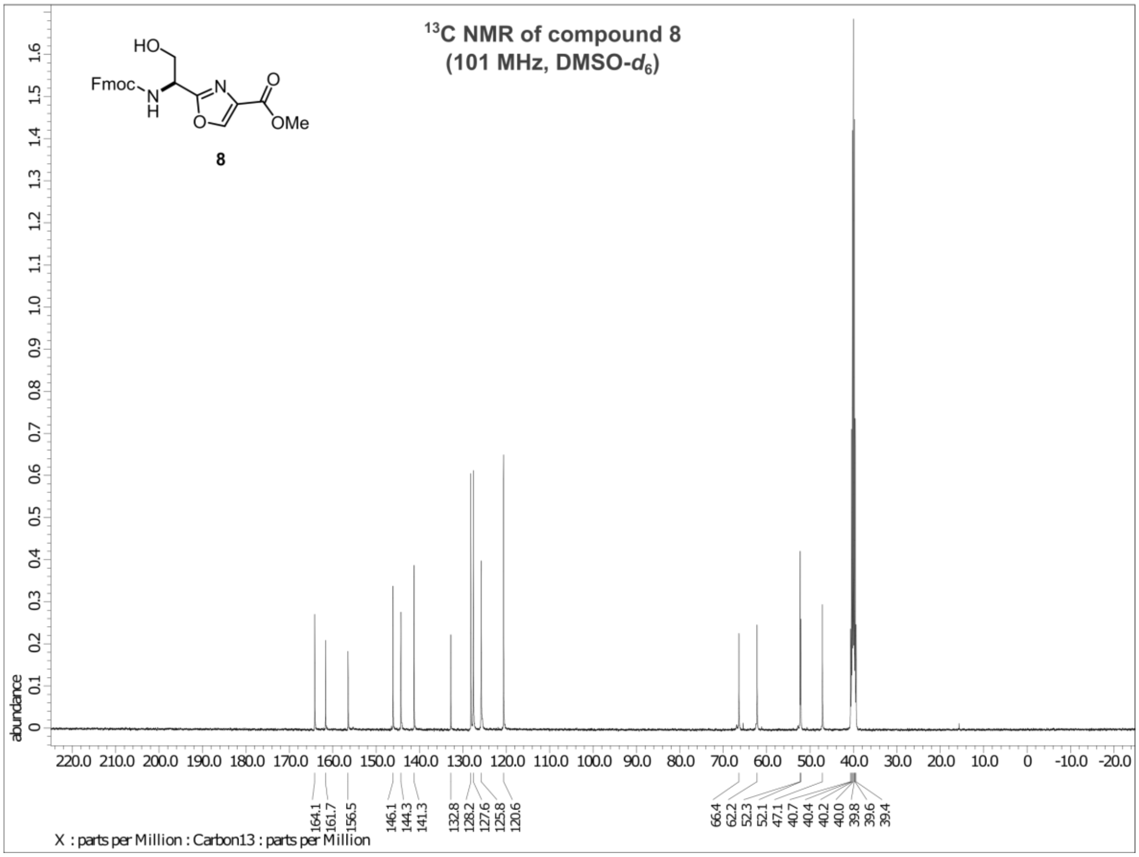

¹³C NMR of compound 8
(101 MHz, DMSO-d6)
HO
Fmoc
N
H
N
O
O
OMe
8
abundance
X : parts per Million : Carbon13 : parts per Million
164.1
161.7
156.5
146.1
144.3
141.3
132.8
128.2
127.6
125.8
120.6
66.4
62.2
52.3
52.1
47.1
40.7
40.4
40.2
40.0
39.8
39.6
39.4

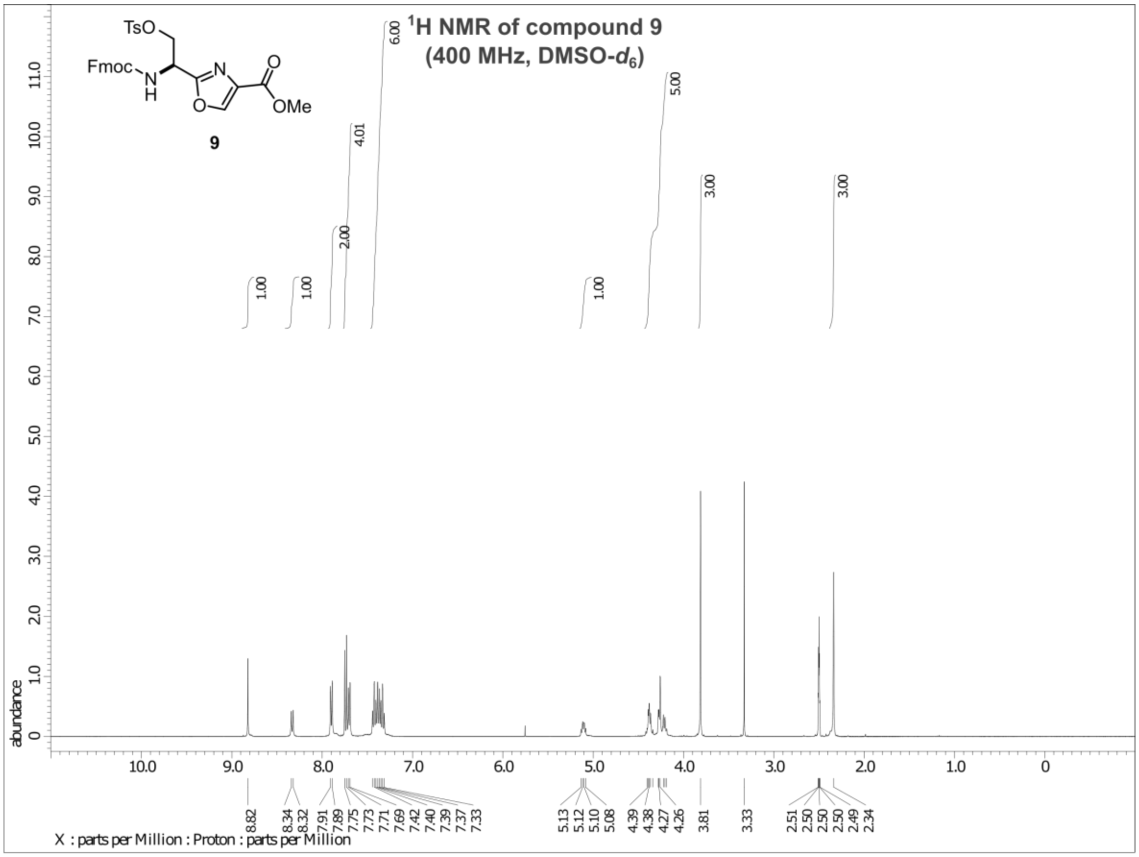

^{1}H NMR of compound 9
(400 MHz, DMSO-d_6)
TsO
Fmoc
N
H
N
O
O
OMe
9
X : parts per Million : Proton : parts per Million
abundance

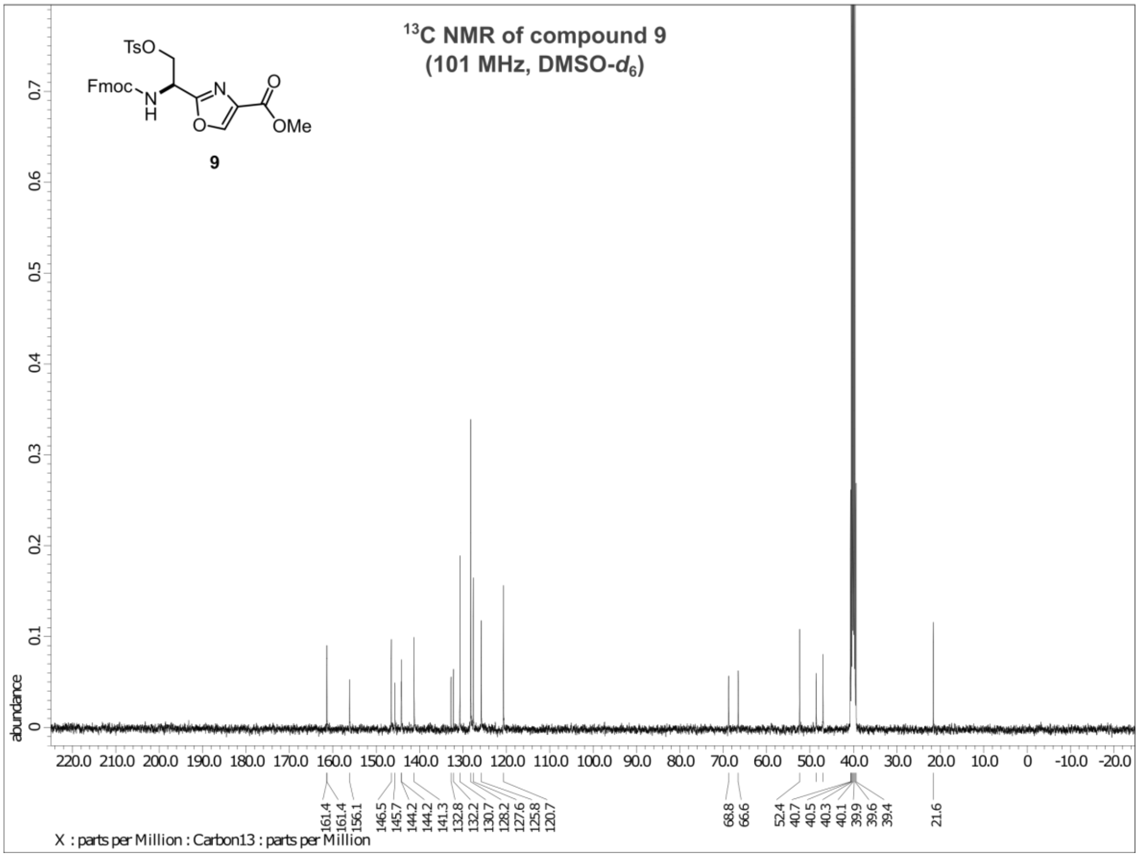

^{13}C NMR of compound 9
(101 MHz, DMSO-d_6)
TsO
Fmoc
N
H
N
O
O
OMe
9
X : parts per Million : Carbon13 : parts per Million
abundance

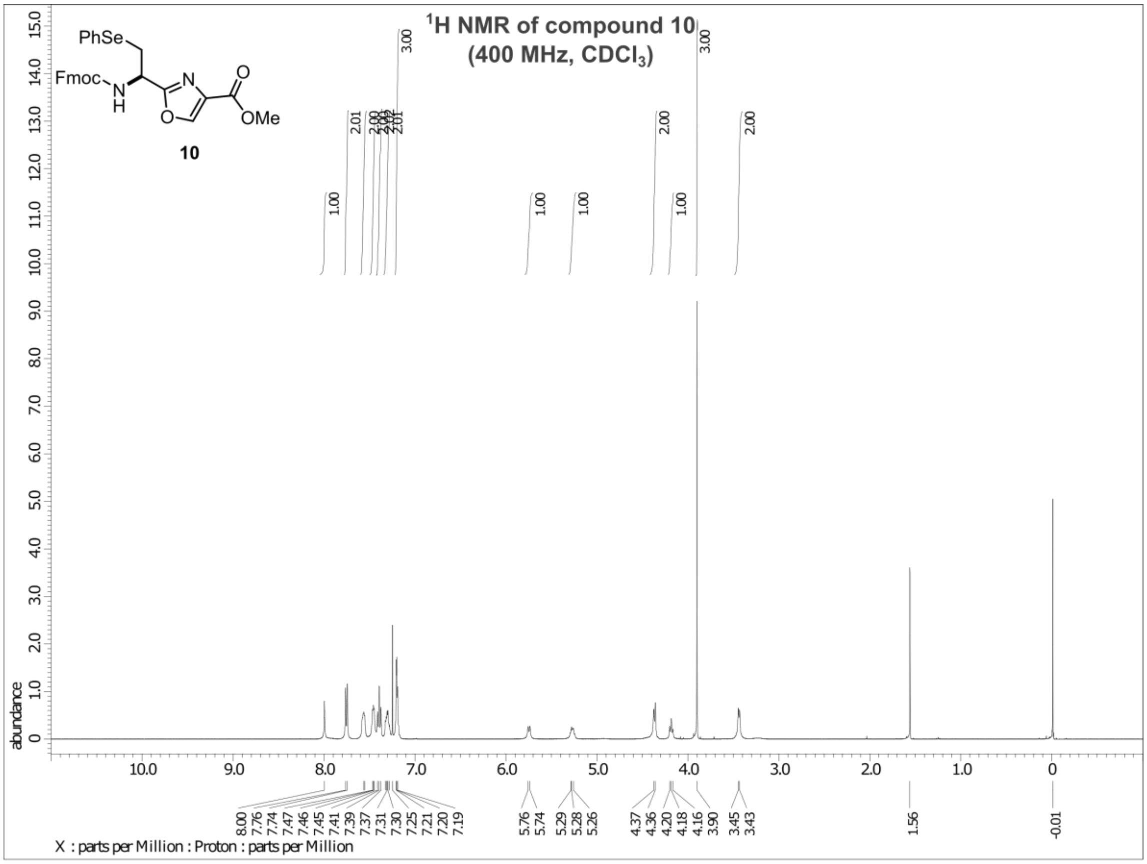

¹H NMR of compound 10
(400 MHz, CDCl₃)
PhSe
Fmoc
N
H
N
O
O
OMe
10
abundance
X : parts per Million : Proton : parts per Million

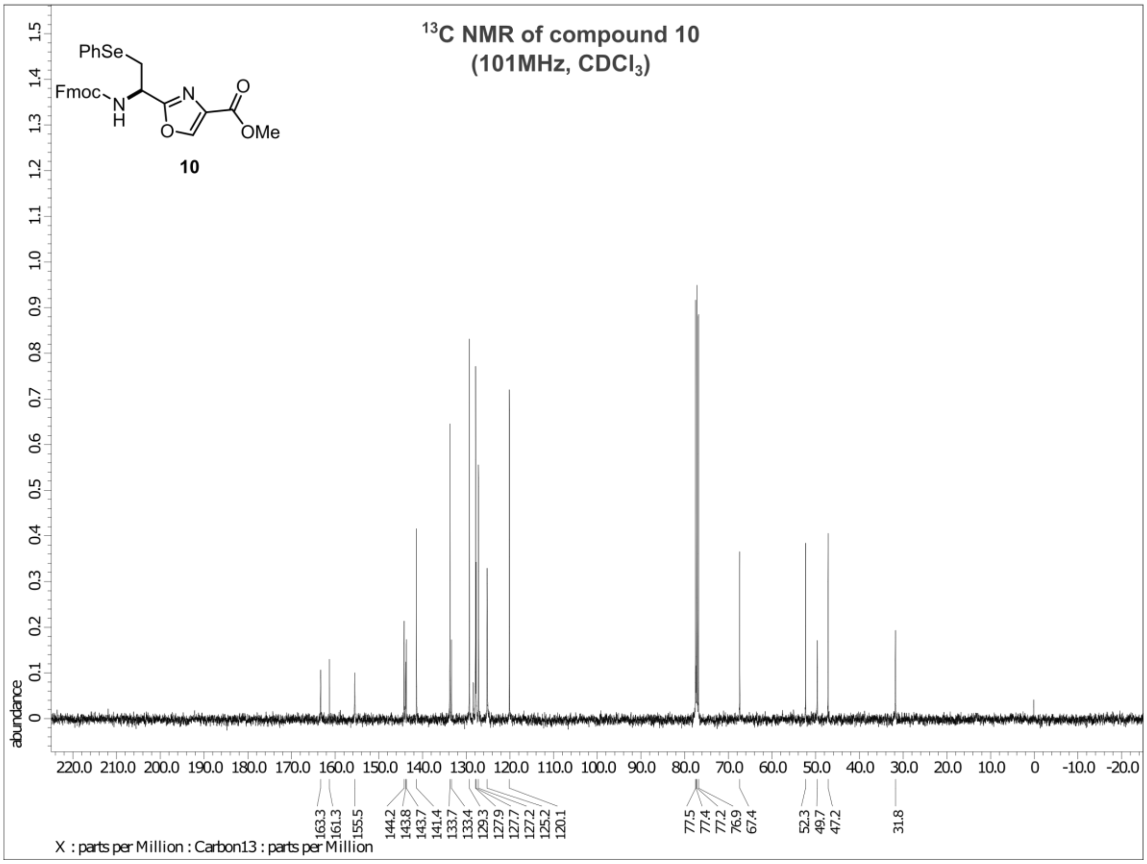

¹³C NMR of compound 10
(101MHz, CDCl₃)
PhSe
Fmoc
N
H
N
O
O
OMe
10
abundance
X : parts per Million : Carbon13 : parts per Million

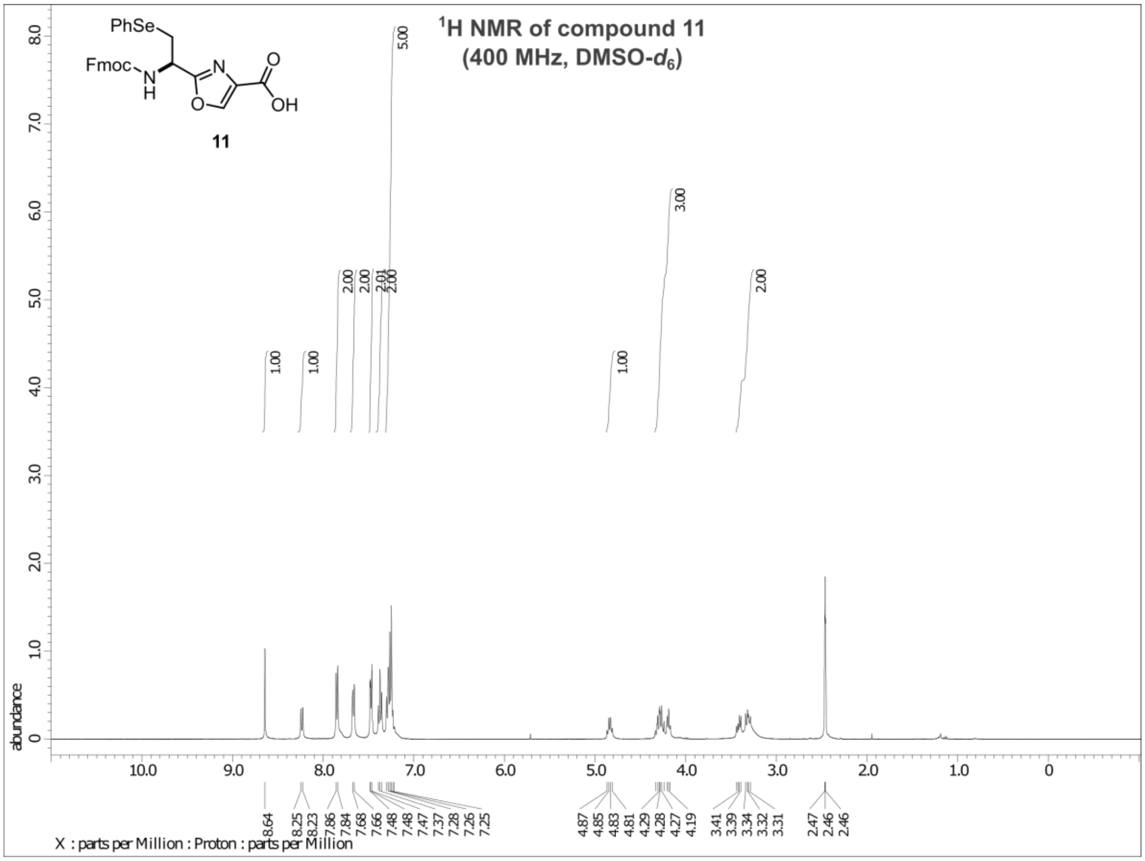

PhSe
Fmoc
N
H
N
O
O
OH
11
^{1}H NMR of compound 11
(400 MHz, DMSO-d_6)
X : parts per Million : Proton : parts per Million
abundance

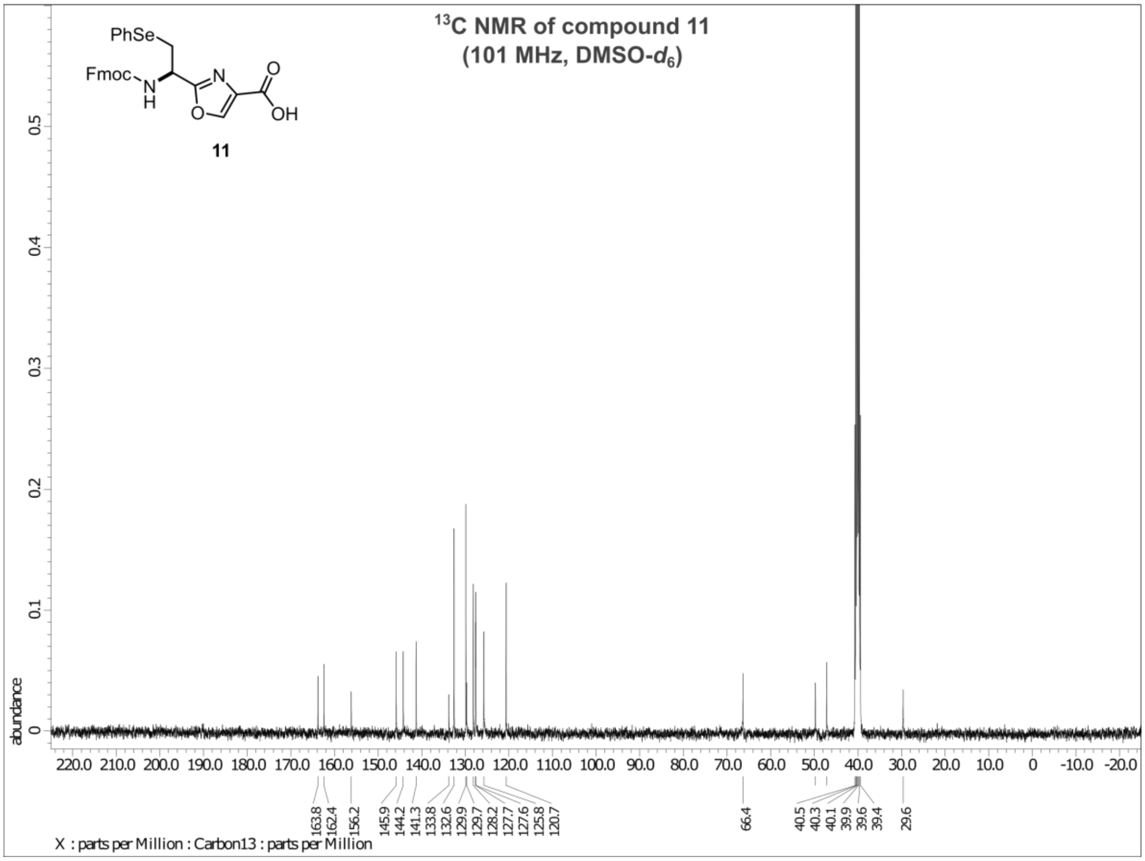

PhSe
Fmoc
N
H
N
O
O
OH
11
^{13}C NMR of compound 11
(101 MHz, DMSO-d_6)
X : parts per Million : Carbon13 : parts per Million
abundance
163.8
162.4
156.2
145.9
144.2
141.3
133.8
132.6
129.9
129.7
128.2
127.7
127.6
125.8
120.7
66.4
40.5
40.3
40.1
39.9
39.6
39.4
29.6

7. References

1) Vinogradov, A. A.; Shimomura, M.; Goto, Y.; Sugai, Y.; Suga, H.; Onaka, H. Minimal Lactazole Scaffold for *in vitro* Thiopeptide Bioengineering. *Nat. Commun.* **2020**, 11, 2272. DOI: 10.1038/s41467-020-16145-4
2) Vinogradov, A. A.; Nagai, E.; Chang, J. S.; Narumi, K.; Onaka, H.; Goto, Y.; Suga, H. Accurate Broadcasting of Substrate Fitness for Lactazole Biosynthetic Pathway from Reactivity Profiling MRNA Display. *J. Am. Chem. Soc.* **2020**, 142, 20329–20334. DOI: 10.1021/jacs.0c10374
3) Vinogradov, A. A.; Nagano, M.; Goto, Y.; Suga, H. Site-Specific Nonenzymatic Peptide S/OGlutamylation Reveals the Extent of Substrate Promiscuity in Glutamate Elimination Domains. *J. Am. Chem. Soc*. **2021**, 143, 13358–13369. DOI: 10.1021/jacs.1c06470
4) Vinogradov, A. A.; Zhang, Y.; Hamada, K.; Chang, J.S.; Okada, C.; Nishimura, H.; Terasaka, N.; Goto, Y.; Ogata, K.; Sengoku, T. et al. De Novo Discovery of Thiopeptide Pseudo-natural Products Acting as Potent and Selective TNIK Kinase Inhibitors. J. Am. Chem. Soc. 2022, 144, 20332−20341. DOI: 10.1021/jacs.2c07937
5) Shimizu, Y.; Inoue, A.; Tomari, Y.; Suzuki, T.; Yokogawa, T.; Nishikawa, K.; Ueda, T. Cell-Free Translation Reconstituted with Purified Components. *Nat. Biotechnol.* **2001**, 19, 751–755. DOI: 10.1038/90802
6) Goto, Y.; Katoh, T.; Suga, H. Flexizymes for Genetic Code Reprogramming. *Nat. Protoc.* **2011**, 6, 779–790. DOI: 10.1038/nprot.2011.331
7) Rogers, D.; Hahn, M. Extended-Connectivity Fingerprints. *J. Chem. Inf. Model*. **2010**, 50, 742– 754. DOI: 10.1021/ci100050t
8) Vinogradov, A. A.; Chang, J. S.; Onaka, H.; Goto, Y.; Suga, H. Accurate Models of Substrate Preferences of Post-Translational Modification Enzymes from a Combination of mRNA Display and Deep Learning. ACS Cent. Sci. 2022, 8, 814-824. DOI: 10.1021/acscentsci.2c00223
9) Kingma, D. P.; Ba, J. L. Adam: A Method for Stochastic Optimization. *arXiv Prepr. arXiv* **2014**, 1412.6980. DOI: 10.48550/arXiv.1412.6980
10) Vaswani, A.; Shazeer, N.; Parmar, N.; Uszkoreit, J.; Jones, L.; Gomez, A. N.; Kaiser, Ł.; Polosukhin, I. Attention Is All You Need. *Adv. Neural Inf. Process. Syst*. **2017**, 5998–6008. DOI: 10.48550/arXiv.1706.03762
11) Srivastava, N.; Hinton, G.; Krizhevsky, A.; Sutskever, I.; Salakhutdinov, R. Dropout: A Simple Way to Prevent Neural Networks from Overfittin. *J. Mach. Learn. Res*. **2014**, 15, 1929–1958. http://jmlr.org/papers/v15/srivastava14a.html
12) McInnes, L.; Healy, J.; Melville, J. Umap: Uniform manifold approximation and projection for dimension reduction. *arXiv preprint arXiv* **2018**, 1802.03426. DOI: 10.48550/arXiv.1802.03426
13) Lee, K. L. ; Ambler, C. M. ; Anderson, D. R. ; Boscoe, B. P. ; Bree, A. G. ; Brodfuehrer, J. I. ; Chang, J. S. ; Choi, C. ; Chung, S. ; Curran, K. J. et al. Discovery of Clinical Candidate 1-{[(2S,3S,4S)-3-Ethyl-4-fluoro-5-oxopyrrolidin-2-yl]methoxy}-7-methoxyisoquinoline-6-carboxamide (PF-06650833), a Potent, Selective Inhibitor of Interleukin-1 Receptor Associated Kinase 4 (IRAK4), by Fragment-Based Drug Design. *J. Med. Chem.* **2017** 60 (13), 5521-5542. DOI: 10.1021/acs.jmedchem.7b00231
14) Virtanen, P.; Gommers, R.; Oliphant, T. E.; Haberland, M.; Reddy, T.; Cournapeau, D.; Burovski, E.; Peterson, P.; Weckesser, W.; Bright, J. et al. SciPy 1.0: Fundamental Algorithms for Scientific Computing in Python. *Nat. Methods* **2020**, 17, 261–272. DOI: 10.1038/s41592-019-0686-2

15) Katoh, T.; Sengoku, T.; Hirata, K.; Ogata, K.; Suga, H. Ribosomal Synthesis and de Novo Discovery of Bioactive Foldamer Peptides Containing Cyclic β-Amino Acids. *Nat. Chem.* **2020**, 12, 1081–1088. DOI: 10.1038/s41557-020-0525-1
16) Peraro, L.; Deprey, K. L.; Moser, M. K.; Zou, Z.; Ball, H. L.; Levine, B.; Kritzer, J. A. Cell Penetration Profiling Using the Chloroalkane Penetration Assay. *J. Am. Chem. Soc*. **2018**, 140, 11360–11369. DOI: 10.1021/jacs.8b06144
17) Kuglstatter, A.; Villasenor, A. G.; Shaw, D.; Lee, S. W.; Tsing, L. S.; Niu, L.; Song, K. W.; Barnett, J. W.; Browner, M. F. Cutting Edge: IL-1 Receptor-Associated Kinase 4 Structures Reveal Novel Features and Multiple Conformations. *J. Immunol.* **2017**, 178, 5, 2641-2645. DOI: 10.4049/jimmunol.178.5.2641
18) Tominaga, H.; Ishiyama, M.; Ohseto, F.; Sasamoto, K.; Hamamoto, T.; Suzuki, K.; Watanabe, M. A water-soluble tetrazolium salt useful for colorimetric cell viability assay. *Anal. Commun*., **1999**, 36, 47. DOI: 10.1039/A809656B
19) Banala, S.; Ensle, P.; Sussmuth, R. D. Total synthesis of the Ribosomally Synthesized Linear Azole-Containing Peptide Plantazolicin A from Bacillus amyloliquefaciens. *Angew. Chem. Int. Ed.* **2013**, 52, 9518-9523. DOI: 10.1002/anie.201302266
20) Zhang, Y.; Vinogradov. A. A.; Chang, J. S.; Goto, Y.; Suga, H. Solid phase-based synthesis of lactazole-like thiopeptides. *Org. Lett*. **2022**, 24, 43, 7894–7899. DOI: 10.1021/acs.orglett.2c02870
