# Supplementary material for: Deep Learning-Driven Library Design for the De Novo Discovery of Bioactive Thiopeptides
Source: ACS Cent Sci. 2023 Nov 7;9(11):2150–60. doi: 10.1021/acscentsci.3c00957 (PMC10683472; doi:10.1021/acscentsci.3c00957)
Supplement: Supplementary file 3 — oc3c00957_si_003.pdf [file oc3c00957_si_003.pdf]

oc-2023-00957v.R1

Name: Peer Review Information for "Deep learning-driven library design for de novo discovery of bioactive thiopeptides"

First Round of Reviewer Comments

Reviewer: 1

Comments to the Author

This manuscript is the latest in the excellent series of papers from the Suga lab focused on the twin goals of 1) understanding the substrate tolerance of the enzymes that produce the thiopeptide lactazole and 2) leveraging engineered lactazoles as ligands that bind therapeutically relevant targets. The paper builds on approach published by many of these same authors in ACS Central Science last year in which a combination of mRNA display with DNA barcoding and next gen sequencing is used to study substrate tolerance of the LazBF and LazDEF enzymes. The current work differs because the entire pathway, comprised of the LazA precursor and LazBCDEF maturation enzymes, are included, resulting in cyclic peptides. This switch also required a change in the screening protocol; given that cyclization occurs concomitant with leader peptide cleavage, the authors screen their libraries via loss of the leader peptide. From these screens the authors train a deep convolutional neural network classifier to predict substrate tolerance. The authors next use the model to design libraries of LazA with reduced codon sets at the positions adjacent to the cyclization point of the peptide. The model predicts that 36% of a fully random library would be modified and cyclized efficiently vs. 53% of the reduced library. Using the reduced codon sets does come at cost of ~4-fold in terms of library size. The authors next screen their library against two targets, IRAK4, a kinase, and TLR10, a receptor. Both are targets for autoimmune diseases. Both screens were successful, and the authors synthesized the lead cyclic peptides using solid-phase synthesis with some bespoke building blocks. For the IRAK4 ligands, the best peptide binds with a Kd of 1.3 nM while the best TLR10 ligand binds with a Kd of 300 nM. In an enzyme assay the IRAK4 ligands can inhibit the kinase with an IC50 in the single micromolar range, and impressively, these IRAK4 ligands are inherently cell-penetrating, as evidenced by a cell-based assay. This manuscript represents a huge amount of work, starting with library design and deep learning and going all the way to high affinity engineered peptide ligands that function inside cells. One potential criticism is that this group has published on similar aspects of this work before; there is an ACS Central Science paper in 2022 on the use NGS and deep learning to study substrate specificity, and there is a JACS paper from 2022 using lactazole libraries to find an inhibitor of the TNIK kinase. Given the new insights into substrate specificity here and the discovery of novel potent peptide ligands, it is my opinion that the work should still be published in a top venue like ACS Central Science. Below are some minor clarifications and suggestions for the paper.

- In the introduction the authors should consider citing Thokkadam et al. ACS Central Science 2023 as a further example of RiPP engineering and library screening, especially since this work included a deep learning approach

- Line 146: the text about the deep learning model should include references to the model

- Line 148: can the authors share how many peptides the model was trained on in order to predict 1.7 x 10^5 peptides? This information may be in the SI, but discussing it in the main text is warranted here.

- Line 186-188: there is a tradeoff between library size and library quality. Based on the numbers in this section, one would expect 7.2 x 10^13 peptides that can be matured in the full library vs. 2.9 x 10^13 in the reduced codon set library. However, based on the discussion in lines 204-207, that “only” 4 x 10^12 members will be screened. (Only is in quotes because this is still an enormous number!) I would suggest that the authors talk about how many peptides can actually be screened experimentally from a given library along with the discussion of library size in the vicinity of lines 184-186 to make the point that not every possible peptide member of the library will get screened.

- The Figure 2 caption should define DADL so that the reader can understand this without going back to the text

- SI, top of page 7: this section discusses the fact that some fraction of data from the NGS gets thrown away. Can the authors give a sense of the fraction of data that is triaged?

- SI page 7, data preprocessing: similar question to above: how much data gets thrown away when comparing the selection and antiselection datasets

- SI page 7: after “hamming distance” there is an incorrect symbol.

- SI page 7: define ECFPs

- SI page 7: more incorrect symbols in the paragraph discussing the Adam optimizer. Either that or the symbols need to be defined.

- SI page 8: In the section titled validation experiment, I am confused by the statement “unlike the previously reported data.” Is this a typo in which unlike should be like? If not, please explain why this data is unlike the previous data.

- Figure S14 caption: the Ser residue in IR17 is shaded green and black, presumably because the authors see a mix of Ser and dehydroAla? The two color shading (which appears in subsequent figures too) should be explained in the caption.

Reviewer: 2

Comments to the Author

I really enjoyed reading this paper and think it will be of great interest to the peptide community. Cyclic peptides are a powerful modality for targeting challenging drug targets, but the clinical development of de novo peptides, rather than those based on natural products, has been hampered by their poor ADME properties. However, recent examples of de novo cyclic peptides entering clinical trials (e.g. 10.1021/jacs.3c03886 from Chugai Pharmaceutical and 10.1021/acs.jmedchem.1c01599 from Merck) suggest that improved peptide library design and optimisation of peptide hits may yet allow this modality to achieve its full potential.

However, design of natural product-like cyclic peptides libraries, that tend to have better ADME properties, can be complex. To address this, by combining their earlier work 1) using machine learning to predict single enzyme substrate preferences (10.1021/jacs.3c03886) and 2) producing pseudo-natural thiopeptide RiPPs (10.1021/jacs.2c07937), Chang and coworkers present a new approach to designing enzymatically modified display libraries that balance modification efficiency with library diversity. Following thorough testing, optimised library designs are applied in peptide selections against two targets, TLR10 and IRAK4. This results in potent RiPP-like ligands that in the case of IRAK4 ligands are cell permeable (although still with relatively modest permeability) and cell active. Overall, the findings of the paper are thoroughly tested and the paper itself well written. Detailed experimental details are provided in the SI alongside a high number of supplementary figures that support the main findings. I found the experimental work convincing and have no important suggestions for modifications. However, I do think the paper would benefit from a more extended discussion that considers their new method in a wider context. Can they give examples of other pathways where they think this approach will be powerful? Are their restrictions to what pathways can be explored that they envisage? Related to this, despite the involvement of 5 enzymes in the RiPP pathway tested here, the authors find that only the first and last amino acids are important – how frequently do they think this will be the case for RiPP pathways, and if infrequently, how straightforward do they think it will be to design libraries with restrictions in multiple positions in the cyclic peptide sequence?

Overall I believe this paper will be of high interest to the broad readership of ACS Central Science, particularly scientists interested in cyclic peptide drug discovery and the drugging of difficult protein targets, and would fully support its publication following minor modification.

Reviewer: 3

Comments to the Author

The manuscript from Chang et al., describe exploiting deep learning tools to understand and exploit the promiscuity of RiPP biosynthetic pathway for the purposes of scaffold design. Although similar efforts have been carried out by the same group, prior instances focused on single enzymes rather than entire biosynthetic pathways. Thus, the work does represent a significant leap forward and is worthy of consideration for ACS Central Science.

That said, there are many instances where technical shortcomings are either minimized or glossed over in light of the success. A more detailed explanation of these drawbacks are necessary to provide suitable context to the reader. Given the broad readership of ACS Central Science, there ought to be more details provided on the items described below.

1. The authors need to detail how the biosynthetic timing is set for the Laz enzymes as the heterocyclases and dehydrates could potentially compete for the same substrate. More experimental details are warranted to explain this to non-specialists.

2. In instances where the cyclodehydrations reactions are stalled, are the subsequent modifications still viable? In other words, how would the promiscuity of the pathway provide data that is significantly different from the promiscuity of the first modification? More details are needed here.

3. There is far too little discussion on the kinetics of binding of the peptides selected against the two targets. The authors should add a few sentences to describe the on and off rates of their selected peptides.

4. There should be some control data to show that the selected peptides are true binders and associate specifically with their targets. The authors should demonstrate lack of binding to a non-cognate protein of choice.

5. TLR10 is an extracellular target, while IRAK4 is intracellular. Presumably full-length proteins were used for the pulldown experiments but I could not find this detail in the SI. Please add more information about the targets.

These clarifications should strengthen the quality of the final manuscript and make it appeal to a broader readership.

Author's Response to Peer Review Comments:

Response to the reviewers of the manuscript "Deep learning-driven library design for de novo discovery of bioactive thiopeptides"

We would like to thank the referees for taking the time to evaluate the manuscript and for their insightful suggestions. Below, please find our line-by-line responses to the specific comments.

Reviewer #1

> This manuscript is the latest in the excellent series of papers from the Suga lab focused on the twin goals of 1) understanding the substrate tolerance of the enzymes that produce the thiopeptide lactazole and 2) leveraging engineered lactazoles as ligands that bind therapeutically relevant targets. The paper builds on approach published by many of these same authors in ACS Central Science last year in which a combination of mRNA display with DNA barcoding and next gen sequencing is used to study substrate tolerance of the LazBF and LazDEF enzymes. The current work differs because the entire pathway, comprised of the LazA precursor and LazBCDEF maturation enzymes, are included, resulting in cyclic peptides. This switch also required a change in the screening protocol; given that cyclization occurs concomitant with leader peptide cleavage, the authors screen their libraries via loss of the leader peptide. From these screens the authors train a deep convolutional neural network classifier to predict substrate tolerance. The authors next use the model to design libraries of LazA with reduced codon sets at the positions adjacent to the cyclization point of the peptide. The model predicts that 36% of a fully random library would be modified and cyclized efficiently vs. 53% of the reduced library. Using the reduced codon sets does come at cost of ~4-fold in terms of library size. The authors next screen their library against two targets, IRAK4, a kinase, and TLR10, a receptor. Both are targets for autoimmune diseases. Both screens were successful, and the authors synthesized the lead cyclic peptides using solid-phase synthesis with some bespoke building blocks. For the IRAK4 ligands, the best peptide binds with a Kd of 1.3 nM while the best TLR10 ligand binds with a Kd of 300 nM. In an enzyme assay the IRAK4 ligands can inhibit the kinase with an IC50 in the single micromolar range, and impressively, these IRAK4 ligands are inherently cell-penetrating, as evidenced by a cell-based assay. This manuscript represents a huge amount of work, starting with library design and deep learning and going all the way to high affinity engineered peptide ligands that function inside cells. One potential criticism is that this group has published on similar aspects of this work before; there is an ACS Central Science paper in 2022 on the use NGS and deep learning to study substrate specificity, and there is a JACS paper from 2022 using lactazole libraries to find an inhibitor of the TNIK kinase. Given the new insights into substrate specificity here and the discovery of novel potent peptide ligands, it is my opinion that the work should still be published in a top venue like ACS Central Science.

Thank you for your positive evaluation of the work!

In the introduction the authors should consider citing Thokkadam et al. ACS Central Science 2023 as a further example of RiPP engineering and library screening, especially since this work included a deep learning approach

The publication in question (ACS Cent. Sci. **2023**, 9, 540–550) is now cited in the introduction (ref. 3).

> • Line 146: the text about the deep learning model should include references to the model

The relevant Supporting information section (S.I. 2.4) is now referenced at the beginning of the paragraph describing data preprocessing and training.

> Line 148: can the authors share how many peptides the model was trained on in order to predict 1.7 x 10^5 peptides? This information may be in the SI, but discussing it in the main text is warranted here.

The preceding sentence now specifies the number of peptides used for training. The sentence reads: “The datasets (8.47 × 10^6 unique peptides) were then used to train a deep convolutional neural network classifier (12 convolutional layers, 1.2 × 10^7 parameters) of substrate fitness for LazA variants.”

> • Line 186-188: there is a tradeoff between library size and library quality. Based on the numbers in this section, one would expect 7.2 x 10^13 peptides that can be matured in the full library vs. 2.9 x 10^13 in the reduced codon set library. However, based on the discussion in lines 204-207, that “only” 4 x 10^12 members will be screened. (Only is in quotes because this is still an enormous number!) I would suggest that the authors talk about how many peptides can actually be screened experimentally from a given library along with the discussion of library size in the vicinity of lines 184-186 to make the point that not every possible peptide member of the library will get screened.

After lines 204–207, a sentence was added to address this suggestion. It reads: “This result also indicates that during the first round of affinity selection, 11- and 12-mer insert libraries are sparsely sampled, i. e., a small fraction of all encoded thiopeptides is produced, and to a first approximation, every compound is unique.”

For the 12-mer library [nbk-$(nnk)_9$-nbk design], taking an $n = 4 \times 10^{12}$ sample from a much larger pool m which contains 5.7×10^{14} unique sequences can be modelled with a hypergeometric distribution. Since $n << m$, the binomial distribution is a good approximation for the sampling process. Thus, the number of unique peptides in the sample can be estimated at 3.988×10^{12} sequences, i. e., nearly every thiopeptide is expected to be unique.

• The Figure 2 caption should define DADL so that the reader can understand this without going back to the text

Figure 2 caption was revised accordingly: “Maturation of model validation peptides (MVP1– 10; see also Table S4 and Fig. S1–10) was ascertained experimentally using a single-round DADL (data acquisition for deep learning applications) assay (S.I. 2.3) or by LC/MS (semiquantitative monitoring of leader-NH2 formation; S.I. 2.7).”

> • SI, top of page 7: this section discusses the fact that some fraction of data from the NGS gets thrown away. Can the authors give a sense of the fraction of data that is triaged

Roughly 50% of NGS reads were triaged as the result of the preprocessing, mostly due to insufficiently high Q scores. Table S3 that lists the dataset sizes at every step of the process for several NGS files was added to the Supporting Tables.

> •SI page 7, data preprocessing: similar question to above: how much data gets thrown away when comparing the selection and antiselection datasets

Likewise, the dataset sizes at every step of preprocessing are contained in the newly added Table S3. Data preprocessing removed only 1.2% of the data.

> • SI page 7: after “hamming distance” there is an incorrect symbol.

The symbol was “less or equal to” (≤) typeset in a non-standard font. The font type was changed to Arial.

> • SI page 7: define ECFPs

The relevant sentence now reads: “Finally, the peptides were represented as matrices of extended-connectivity fingerprints (ECFPs) for model training.”

> SI page 7: more incorrect symbols in the paragraph discussing the Adam optimizer. Either that or the symbols need to be defined.

Similar to the comment above, the font type was changed to Arial. The symbols (β_1, β_2, and ε) are the standard Adam optimizer parameters as defined in the original publication and implemented in common deep learning libraries such as tensorflow and pytorch.

• SI page 8: In the section titled validation experiment, I am confused by the statement “unlike the previously reported data.” Is this a typo in which unlike should be like? If not, please explain why this data is unlike the previous data.

The comparison to the previously reported data was removed altogether as it was not helpful.

> • Figure S14 caption: the Ser residue in IR17 is shaded green and black, presumably because the authors see a mix of Ser and dehydroAla? The two color shading (which appears in subsequent figures too) should be explained in the caption.

The following sentence was added to the caption of Fig. S14 and S15: “Simultaneous green and black highlighting indicates the sites of partial dehydration of Ser/Thr residues which leads to the formation of product mixtures.”

Reviewer #2

> I really enjoyed reading this paper and think it will be of great interest to the peptide community. Cyclic peptides are a powerful modality for targeting challenging drug targets, but the clinical development of de novo peptides, rather than those based on natural products, has been hampered by their poor ADME properties. However, recent examples of de novo cyclic peptides entering clinical trials (e.g. 10.1021/jacs.3c03886 from Chugai Pharmaceutical and 10.1021/acs.jmedchem.1c01599 from Merck) suggest that improved peptide library design and optimisation of peptide hits may yet allow this modality to achieve its full potential.

> However, design of natural product-like cyclic peptides libraries, that tend to have better ADME properties, can be complex. To address this, by combining their earlier work 1) using machine learning to predict single enzyme substrate preferences (10.1021/jacs.3c03886) and 2) producing pseudo-natural thiopeptide RiPPs (10.1021/jacs.2c07937), Chang and coworkers present a new approach to designing enzymatically modified display libraries that balance modification efficiency with library diversity. Following thorough testing, optimised library designs are applied in peptide selections against two targets, TLR10 and IRAK4. This results in potent RiPP-like ligands that in the case of IRAK4 ligands are cell permeable (although still with relatively modest permeability) and cell active. Overall, the findings of the paper are thoroughly tested and the paper itself well written. Detailed experimental details are provided in the SI alongside a high number of supplementary figures that support the main findings. I found the experimental work convincing and have no important suggestions for modifications. <…>

> Overall I believe this paper will be of high interest to the broad readership of ACS Central Science, particularly scientists interested in cyclic peptide drug discovery and the drugging of difficult protein targets, and would fully support its publication following minor modification.

Thank you for your enthusiastic appraisal of the work!

> However, I do think the paper would benefit from a more extended discussion that considers their new method in a wider context. Can they give examples of other pathways where they think this approach will be powerful? Are their restrictions to what pathways can be explored that they envisage?

The conclusion section was expanded accordingly. The additional discussion reads: "In principle, analogous pipelines can be constructed for other RiPP biosynthetic pathways so long as the enzymatic reaction products can be chemically differentiated from the starting materials and intermediates. Many RiPP pathways amenable to combinatorial workflows (lanthipeptides, lasso peptides, cyanobactins, graspetides, and spliceotides among others) may benefit from a comprehensive substrate profiling in an mRNA display format. Modelguided library design may be particularly useful for the pathways with enigmatic substrate preferences (e. g., prochlorosins) or highly cooperative biosynthesis (thiopeptides, polytheonamides), where the substrate fitness landscapes become too complex for the manual construction of optimal library designs."

> Related to this, despite the involvement of 5 enzymes in the RiPP pathway tested here, the authors find that only the first and last amino acids are important – how frequently do they think this will be the case for RiPP pathways, and if infrequently, how straightforward do they think it will be to design libraries with restrictions in multiple positions in the cyclic peptide sequence?

For many RiPP enzymes, the substrate preferences are primarily determined by the amino acids surrounding the modification site (notably, azoline-forming YcaO enzymes), but there are also examples where the recognition requirements are more extensive (for example, see J. Am. Chem. Soc. **2022**, 144, 25, 11263–11269). Our model-guided design approach can be utilized in both cases (arguably, it would be more beneficial in the latter case, because the manual library design becomes more challenging). As long as a scoring function (or a reward function; M^{long} and M^{short} in our case) can be constructed, and an unambiguous mapping between the composition of a library and its M score exists (the model serves as the mapping), it should be possible to optimize the library composition to maximize the scoring function. In our case, the relationship between the library composition and the scoring function turned out relatively straightforward, and thus, we opted for a semiautomated design process. In a general case, modern mathematical optimization techniques (e. g., evolutionary/genetic algorithms and reinforcement learning) can fully automate the process even for highly nuanced biosynthesis pathways.

Reviewer #3

> The manuscript from Chang et al., describe exploiting deep learning tools to understand and exploit the promiscuity of RiPP biosynthetic pathway for the purposes of scaffold design. Although similar efforts have been carried out by the same group, prior instances focused on single enzymes rather than entire biosynthetic pathways. Thus, the work does represent a significant leap forward and is worthy of consideration for ACS Central Science.

Thank you for your supportive comments!

> 1. The authors need to detail how the biosynthetic timing is set for the Laz enzymes as the heterocyclases and dehydrates could potentially compete for the same substrate. More experimental details are warranted to explain this to non-specialists.

The order of post-translational modifications in lactazole biosynthesis is unusual compared to other thiopeptides. Although a complete description of the PTM installation order (studied previously in J. Am. Chem. Soc. **2020**, 142, 13886–13897), would detract from the present work's thesis, we agree that elaborating on this aspect is important. A sentence at the beginning of the Results and Discussion section was added to strengthen the discussion: "Substrate preferences of the full pathway are obfuscated by the fact that the enzymes extensively cooperate and compete with each other during biosynthesis.[18] Cyclodehydration of Ser and Cys residues, azoline dehydrogenation, and dehydroalanine formation events are intertwined during the maturation process to maintain the integrity of the biosynthesis and minimize the formation of shunt products. <…>"

> In instances where the cyclodehydrations reactions are stalled, are the subsequent modifications still viable? In other words, how would the promiscuity of the pathway provide data that is significantly different from the promiscuity of the first modification? More details are needed here.

A sentence that qualifies various contributions to the overall substrate fitness of the lactazole biosynthesis pathway was added to the Results and Discussion section: "Cyclodehydration of Ser and Cys residues, azoline dehydrogenation, and dehydroalanine formation events are intertwined during the maturation process to maintain the integrity of the biosynthesis and minimize the formation of shunt

products. The substrate preferences of the whole pathway are comprised of the preferences of the individual enzymes and numerous second order effects stemming from the fact that the installation of post-translational modification (**PTM**s) influence each other."

Cyclodehydration alone does not explain the observed specificity profile; LazBF (Ser dehydratase) and LazC (pyridine synthase) have their own substrate preferences which i) do not match those of LazDEF (ACS Cent. Sci. **2022**, 8, 814–824) and ii) pose additional constraints on the amino acids permissible in the random region. The easiest place to see these restrictions is the strong selection for certain amino acids in position 3, which is adjacent to the site of Ser1 dehydration and pyridine formation, but is far from the action of LazDEF (positions 15 and 17 in library n11).

> There is far too little discussion on the kinetics of binding of the peptides selected against the two targets. The authors should add a few sentences to describe the on and off rates of their selected peptides.

A Table S7 and a sentence describing the kinetics of binding was added to the relevant text which now reads: "For TLR10, out of the 10 synthesized thiopeptides, seven bound to the extracellular domain of TLR10 with sub-µM affinities. The ligand association rates varied between 5×10^3 and 5×10^4 $M^{-1}s^{-1}$, and in the five out of seven cases, the dissociation rates were below 10^{-2} s^{-1} (Table S7)."

> There should be some control data to show that the selected peptides are true binders and associate specifically with their targets. The authors should demonstrate lack of binding to a non-cognate protein of choice.

The mRNA display-based evaluation of the thiopeptides' affinity toward IRAK4 and TLR10 (the last part of the "Selections against IRAK4 and TLR10" section) indicated that the discovered compounds associated selectively with their respective target proteins. Note that the "beads only" control (Fig. S24) is somewhat of a misnomer, as in both cases the beads bore an immobilized protein: in the case of IRAK4, streptavidin-immobilized beads were used, and for TLR10, the beads displayed Protein G. Although the qPCR results are semiquantitative, the high recovery ratios (>50 in every case for the pulldown against the target vs. an unrelated protein), support the notion that the discovered ligands are selective for their target proteins.

> TLR10 is an extracellular target, while IRAK4 is intracellular. Presumably full-length proteins were used for the pulldown experiments but I could not find this detail in the SI. Please add more information about the targets.

This information is provided in S. I. section 1: "Biotinylated IRAK4 (kinase domain, amino acids 1-460; NP_057207.2) was purchased from Carna Biosciences (Kobe, Japan). Recombinant human TLR10 Fc Chimera protein (extracellular domain, amino acid 20-576;
Q9BXR5) was purchased from R&D system (#6619-TR-050)."
